# Radical Replacement Process for Ligated Boryl Radical-Mediated Activation of Unactivated Alkyl Chlorides for C(sp^3^)–C(sp^3^) Bond Formation

**DOI:** 10.1021/jacs.4c10915

**Published:** 2024-09-12

**Authors:** Chang-Zhen Fang, Bei-Bei Zhang, Yong-Liang Tu, Qiang Liu, Zhi-Xiang Wang, Xiang-Yu Chen

**Affiliations:** † School of Chemical Sciences, University of Chinese Academy of Sciences, Beijing National Laboratory for Molecular Sciences, Beijing 100049, China; ‡ Binzhou Institute of Technology, Weiqiao-UCAS Science and Technology Park, Binzhou, Shandong Province 256606, China

## Abstract

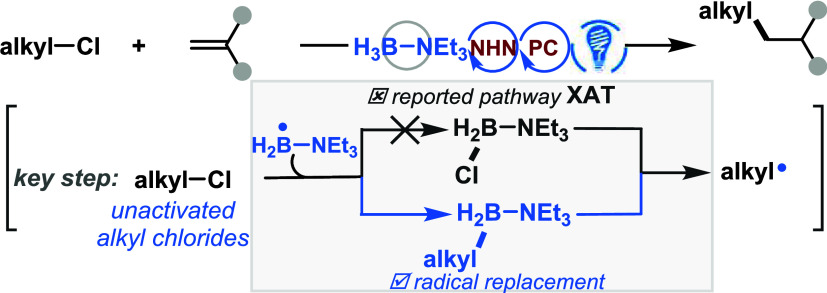

The ligated boryl radical (LBR) has emerged as a potent tool for activating alkyl halides in radical transformations through halogen-atom transfer (XAT). However, unactivated alkyl chlorides still present an open challenge for this strategy. We herein describe a new activation mode of the LBR for the activation of unactivated alkyl chlorides to construct a C(sp^3^)–C(sp^3^) bond. Mechanistic studies reveal that the success of the protocol relies on a radical replacement process between the LBR and unactivated alkyl chloride, forming an alkyl borane intermediate as the alkyl radical precursor. Aided with the additive K_3_PO_4_, the alkyl borane then undergoes one-electron oxidation, generating an alkyl radical. The incorporation of the radical replacement activation model to activate unactivated alkyl chlorides significantly enriches LBR chemistry, which has been applied to activate alkyl iodides, alkyl bromides, and activated alkyl chlorides via XAT.

## Introduction

**Figure 1:**
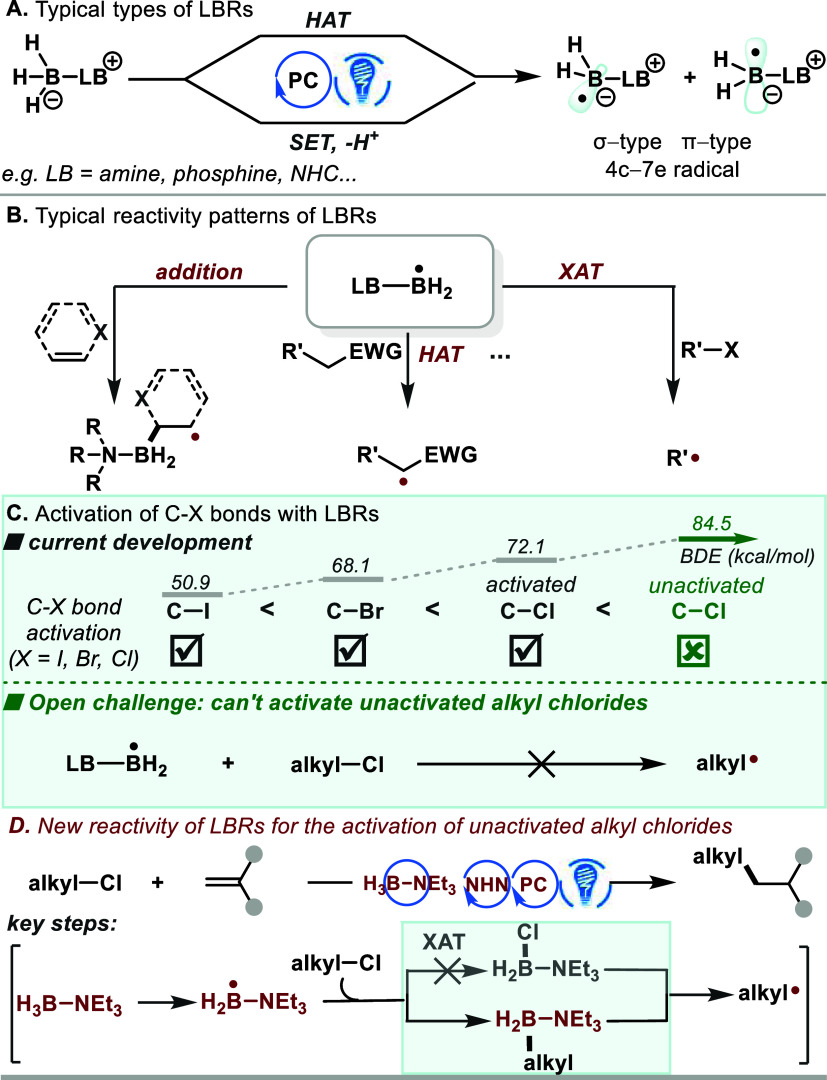
Current reactivity patterns of LBRs and the development of their new activation mode for the activation of unactivated alkyl chlorides.

Boryl radicals ligated with amine, phosphine, or N-heterocyclic carbene, known as ligated boryl radicals (LBRs), are valuable additions to synthetic chemistry.^1^ LBRs are characterized as 4-center-7-electron (4c-7e) radicals, offering a desirable balance among reactivity, stability, and accessibility (Figure 1A). These radicals exhibit diverse reactivity patterns, enabling a variety of transformations, such as additions to unsaturated C–C, C–N/O bonds and (hetero)arenes,^2^ hydrogen-atom transfer (HAT) from protic positions^3^ and others^4^ (Figure 1B). Additionally, LBRs can act as halogen-atom transfer (XAT) agents,^1a,5^ effectively activating alkyl iodides, alkyl bromides, and activated alkyl chlorides to generate alkyl radicals. Recent work by Zhang, Fu, Wang, and co-workers demonstrated that LBRs could selectively cleave activated trichloromethyl group, facilitating the construction of diversely substituted all-carbon quaternary centers.^6^ Another significant advancement involves the combination of LBRs with organophotoredox catalysis for activating alkyl iodides and activated alkyl chlorides, as shown by the research groups of Ma, Zhang, Wu,^7^ and Noël.^8^ Despite these advances, particularly when paired with photocatalysis, a notable challenge still persists for LBRs: their inability to activate unactivated alkyl chlorides (Figure 1C). This substrate class poses a challenge for the generation of alkyl radicals, although they are among the most convenient starting materials in chemical synthesis.^9^

If realized, such a process would not only uncover new potential applications of LBRs but also introduce a new activation mode for generating alkyl radicals from unactivated alkyl chlorides. However, developing a catalytic methodology for forming C(sp^3^)-C(sp^3^) bonds with unactivated alkyl chlorides via radical intermediates remains one of the most formidable challenges in synthetic chemistry. The main difficulties in utilizing these substrates in radical chemistry are their highly negative reduction potentials and strong bond dissociation energies.^10,11^ Several examples in the activation of unactivated alkyl chlorides to alkyl radicals highlight the challenge of developing a robust, transition metal-free photocatalytic system for constructing intermolecular C(sp^3^)-C(sp^3^) bonds.^11^ Existing studies primarily focus on hydrodechlorination^12^ and the formation of C–X (X=O, B) bonds.^13^ Intermolecular C(sp^3^)-C(sp^3^) bond formations typically necessitate the use of UV light and transition metals.^14^

It has been established that LBRs cannot activate unactivated alkyl chlorides through the XAT process,^5−8,15^ but alkyl boranes can be converted to alkyl radicals.^16^ In light of these facts, we envisioned an alternative approach for LBRs to activate unactivated alkyl chloride. As outlined in Figure 1D, the in situ generated potent nucleophilic LBR (specifically, the amine-ligated boryl radical)^17^ could potentially engage in a radical replacement process with unactivated alkyl chlorides, rather than an XAT process, leading to an alkyl borane intermediate. The alkyl borane then undergoes one-electron oxidation process to yield the desired alkyl radical. These two sequential processes effectively bypass the limitations of LBRs in activating unactivated alkyl chlorides, converting them into alkyl radicals. To our knowledge, the radical replacement activation of C-X bonds has not been previously demonstrated with LBRs. As our ongoing interest in photoinduced C–C bond formation,^18^ we herein demonstrate that the radical replacement activation mode of LBRs provides a general platform for forming C(sp^3^)-C(sp^3^) bonds using unactivated alkyl chlorides as alkylating agents under transition-metal-free conditions. This new activation mode significantly expands the utility of LBRs in synthetic chemistry.

## Results and Discussion

We interrogated our perception by investigating the model reaction of *N*-methyl-*N*-phenylmethacrylamide (**1**) with (3-chloropropyl)benzene (**2**). Pleasingly, we discovered that employing N-heterocyclic nitrenium^19^ (NHN) **A1** as a photoreductant,^20,21^ along with BH_3_–NEt_3_ as the ligated boryl radical precursor, 10-phenyl-10H-phenothiazine (PTH) as the photooxidant, and K_3_PO_4_ as a base, yielded the desired product (**3**) in 84% yield (Table 1, entry 1). Omitting any of these components (NHN **A1**, BH_3_–NEt_3_, PTH, and K_3_PO_4_) from the reaction mixture resulted in significantly reduced yields, highlighting their crucial roles (entries 2–6). The control experiment confirmed the indispensability of visible light (entry 7).

**Table 1:**
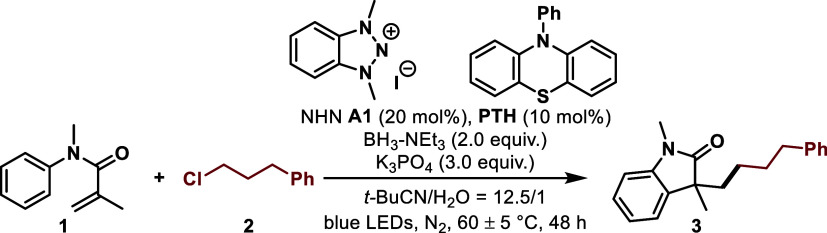
Reaction Condition Optimization^a^

entry	variations from standard conditions	yield (%)^b^
1	none	84
2	without NHN **A1**	trace
3	NaI instead of NHN **A1**	trace
4	without BH_3_–NEt_3_	8
5	without PTH	17
6	without K_3_PO_4_	trace
7	without irradiation, 60 °C	NR

a Reaction condition: **1** (0.2 mmol), **2** (1.0 mmol), NHN **A1** (20 mol %), PTH (10 mol %), BH_3_–NEt_3_ (0.4 mmol), K_3_PO_4_ (0.6 mmol), H_2_O (80 μL) and *t*-BuCN (1.0 mL).

b Yields of isolated products after chromatography. See Table S1 for more reaction condition optimization results.

**Scheme 1:**
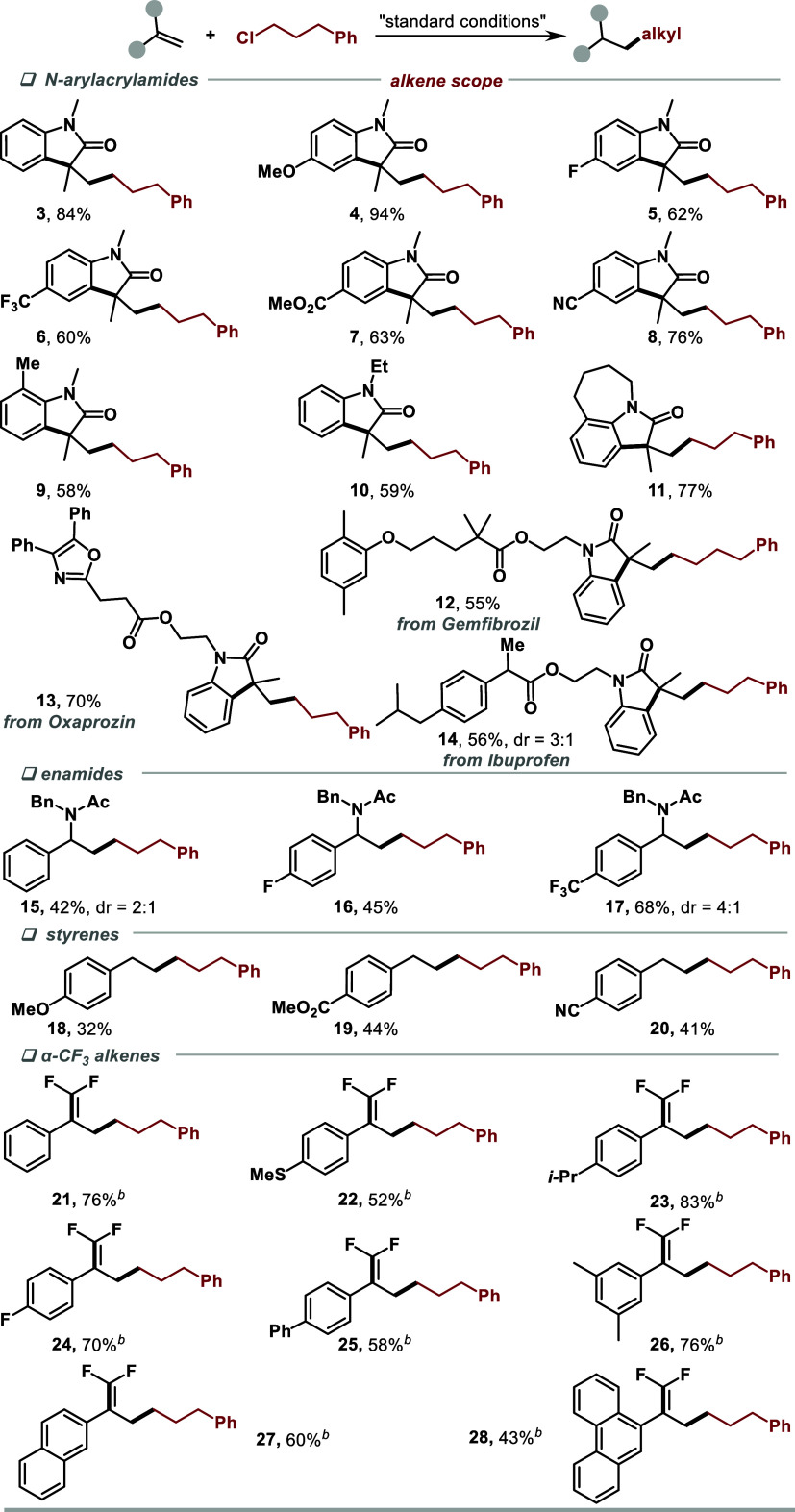
Substrate Scope of Alkenes Isolated yields, 0.2 mmol scale. KI (20 mol %) was added. The dr values were determined by ^1^H NMR analysis.

Having the optimized conditions in hand, we investigated the reaction scope (Scheme 1). Various alkenes were first tested with (3-chloropropyl)benzene **2** as the reaction partner and we found that the mild reaction conditions accommodated a broad scope of alkenes. A variety of substituted N-arylacrylamides bearing both electron-donating (-OMe) and electron-withdrawing (–F, −CF_3_, −CO_2_Me, and −CN) groups at the para- position proceeded well, providing the corresponding products **4**–**8** in 60–94% yields. N-arylacrylamides with a methyl group at the ortho-position gave the desired product **9** in 58% yield. Ethyl substitution at the N atom of the substrate also worked well to afford **10** in 59% yield. That was also true for the cyclic N-acylamide (**11**). Moreover, substrates derived from Gemfibrozil, Oxaprozin, and Ibuprofen were converted to the corresponding products **12**–**14** in 55–70% yields. Additionally, both enamides and styrenes were successful, giving the target products **15**–**20** in moderate yields. Various α-CF_3_ alkenes reacted as well and afforded the corresponding gem-difluoroalkenes **21**–**28** in 43–83% yields.

**Scheme 2:**
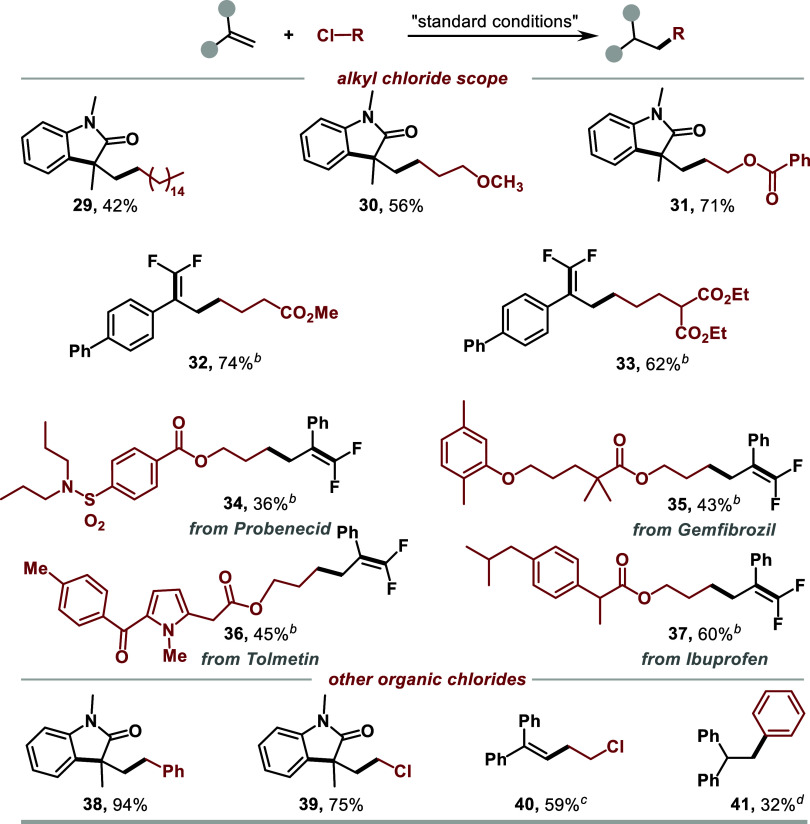
Substrate Scope of Organic Chloridesb Isolated yields, 0.2 mmol scale. KI (20 mol %) was added. 1,2-dichloroethane (0.5 mL). 72 h.

To demonstrate the synthetic utility of this strategy, we then examined the scope of unactivated alkyl chlorides (Scheme 2). Remarkably, the method worked with a variety of unactivated alkyl chlorides bearing different functional groups, such as alkyl chains, ether, and esters (**29**–**33**). Furthermore, modifications of the unactivated alkyl chloride derivatives of Probenecid, Gemfibrozil, Tolmetin, and Ibuprofen were also successful, affording the desired products **34**–**37** in 36–60% yields. Furthermore, reactions of benzyl chloride, dichloromethane, and 1,2-dichloroethane gave the desired products **38**–**40** in 54–94% yields. Aryl chloride also worked in the reaction, albeit with a decreased yield (**41**). When allylic chloride was used as the substrate, the dimerization product was observed via GCMS. Unfortunately, when secondary and tertiary alkyl chlorides were employed, only trace amounts of products were observed under the current conditions.

**Figure 2:**
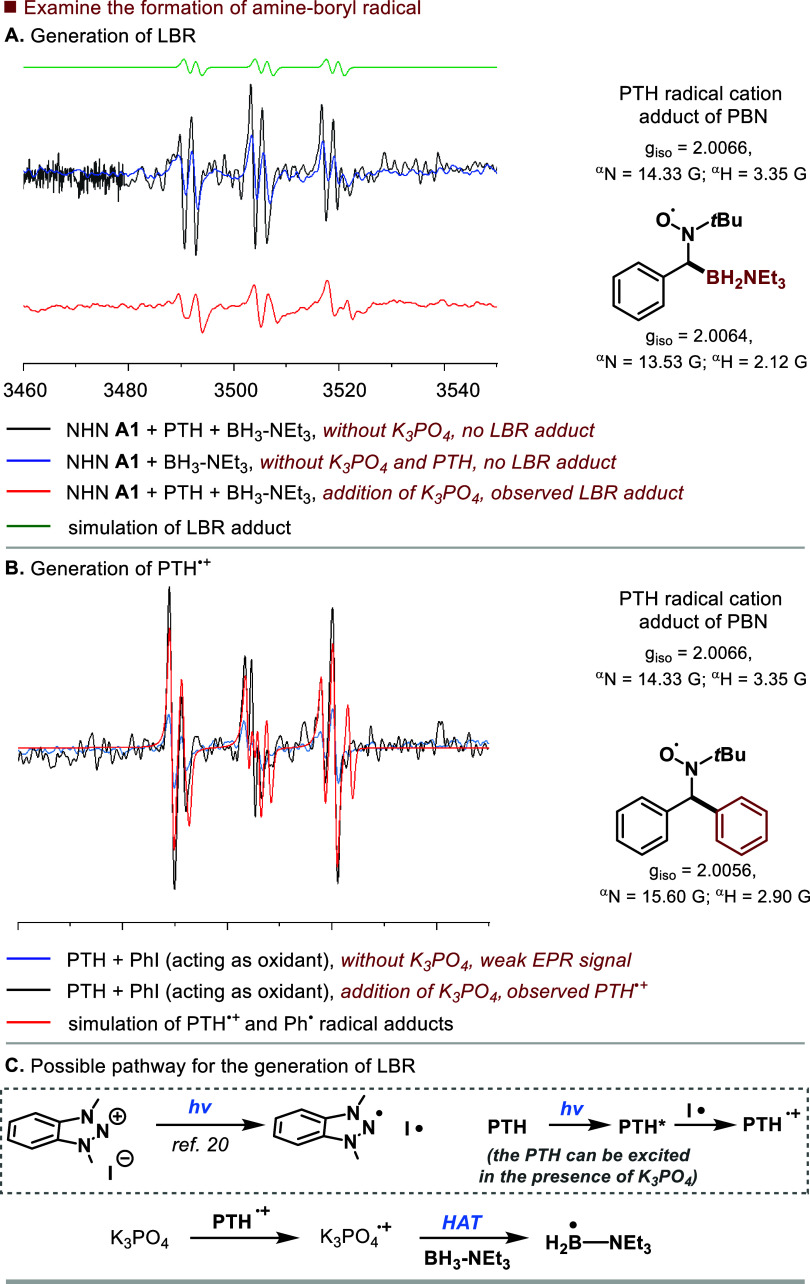
Mechanistic studies to examine the formation of amine-boryl radical.

To elucidate the reaction mechanism, we first investigated the potential generation of the amine-ligated boryl radical under standard reaction conditions. In our system, the photogenerated I^•^ (*E*_ox_ = +0.30 V vs SCE)^22^ and PTH^•+^ (*E*_ox_ = +0.67 V vs SCE)^23^ radicals cannot oxidize BH_3_-NEt_3_ (*E*_ox_ = +2.05 V vs SCE),^7^ making oxidation and deprotonation processes for LBR generation unlikely. Electron paramagnetic resonance (EPR) studies revealed the crucial role of K_3_PO_4_ in generating LBR within this system. A solution of PTH, NHN **A1**, BH_3_-NEt_3_, and N-*tert*-butyl-α-phenylnitrone (PBN) monitored by EPR spectroscopy showed no LBR adduct signal. However, upon the addition of K_3_PO_4_, an LBR adduct signal was observed (Figure 2A; see Figures S5–S10 for details). Additionally, the PTH^•+^ radical cation was also detected.^24^ As PTH does not absorb visible light alone, we surmised that the addition of K_3_PO_4_ enhanced the photoactivity of PTH, as shown in UV–vis studies (see Figure S2). Furthermore, EPR studies indicated that PTH could be excited by blue light irradiation in the presence of K_3_PO_4_. A solution of PTH, PhI (acting as an oxidant), and PBN monitored by EPR spectroscopy showed obvious PTH^•+^ and Ph^•^ radical adduct signals when K_3_PO_4_ was added (Figure 2B). These results suggest that PTH can be excited by blue light irradiation in the presence of K_3_PO_4_, leading to the reduction of PhI to the Ph^•^ radical by PTH*. To further support our proposal that PTH could be excited by blue light in our catalytic system, we replaced PTH with the visible light-active photocatalysts 4CzIPN or Ir[(dF(CF_3_)ppy)_2_(CF_3_bpy)]PF_6_ to run the model reaction in Table 1. The comparable yields (67% and 63%, respectively, Table S1) indicated that PTH in our catalytic system acted similarly to the two catalysts. Based on these findings, we propose that LBR is generated from BH_3_-NEt_3_ via HAT with K_3_PO_4_^•+^, which is formed through one-electron oxidation of K_3_PO_4_ with PTH^•+^ (Figure 2C).

**Figure 3:**
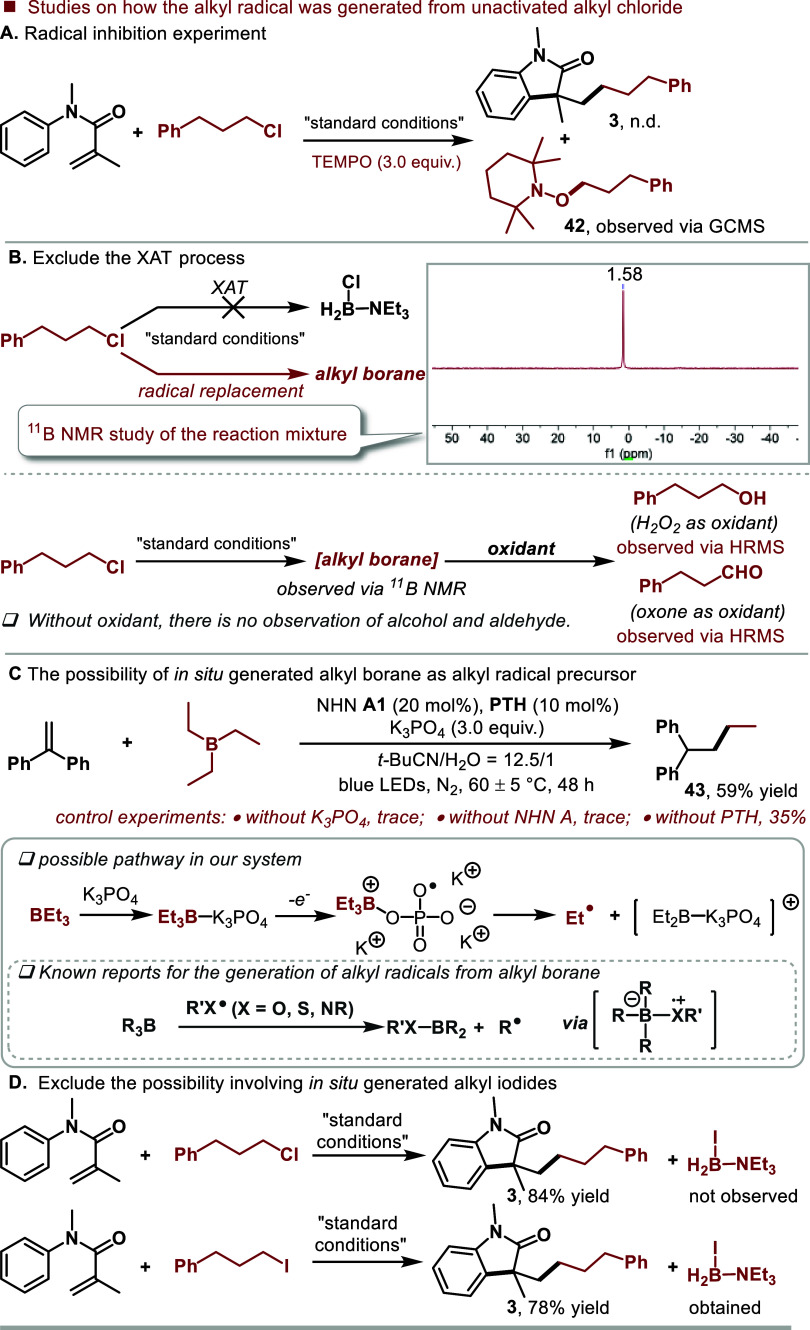
Mechanistic studies on how the alkyl radical was generated from unactivated alkyl chloride.

Subsequent inquiries focused on the potential formation of the alkyl radical from unactivated alkyl chlorides. The radical inhibition experiment suggested the involvement of an alkyl radical (Figure 3A). According to known studies,^7,8^ LBRs could not undergo XAT process with unactivated alkyl chlorides. Consistently, we could not observe the B–Cl peak at around −4.20 ppm via ^11^B NMR,^25^ indicating that the amine-boryl radical did not undergo XAT with the unactivated alkyl chloride **2**. The ^11^B NMR analysis of the reaction mixture showed a peak at 1.58 ppm (Figure 3B, top), which could be assigned to alkyl borane. Additionally, we performed a hydroboration/oxidation sequence using H_2_O_2_ or oxone^2e^ as oxidants, revealing primary alcohol formation with H_2_O_2_ and aldehyde formation with oxone (Figure 3B, bottom). These results further support the potential formation of alkyl borane. According to these experimental results, we hypothesized that the alkyl boranes were generated via radical replacement reaction of amine-ligated boryl radical with unactivated alkyl chlorides and the alkyl boranes served as in situ generated alkyl radical precursors. To support our hypothesis, we employed triethyl borane to replace BH_3_–NEt_3_ and ran the radical trapping experiment with 1,1-diphenyl alkene as the radical scavenger. The trapping product **43** could be obtained in 59% yield, indicating the generation of ethyl radical from triethyl borane. Control experiments further confirmed the more important role of NHN **A1** and K_3_PO_4_ than PTH in this step (Figure 3C, top). Accordingly, we proposed that the generated Et_3_B–K_3_PO_4_ could undergo one-electron oxidation with PTH^•+^ or I^•^ for the generation of ethyl radical (Figure 3C, bottom). Supportively, it was reported that an alkyl radical could be also generated from alkyl borane via a radical replacement process with heteroatom-centered radicals.^16a−16c^ Moreover, a possible pathway via in situ generated alkyl iodide from NHN **A1** could be excluded, as evidenced by the absence of Et_3_N-BH_2_I (Figure 3D, top). In contrast, significant amounts of Et_3_N-BH_2_I could be obtained when alkyl iodide was employed as the substrate (Figure 3D, bottom).

**Figure 4:**
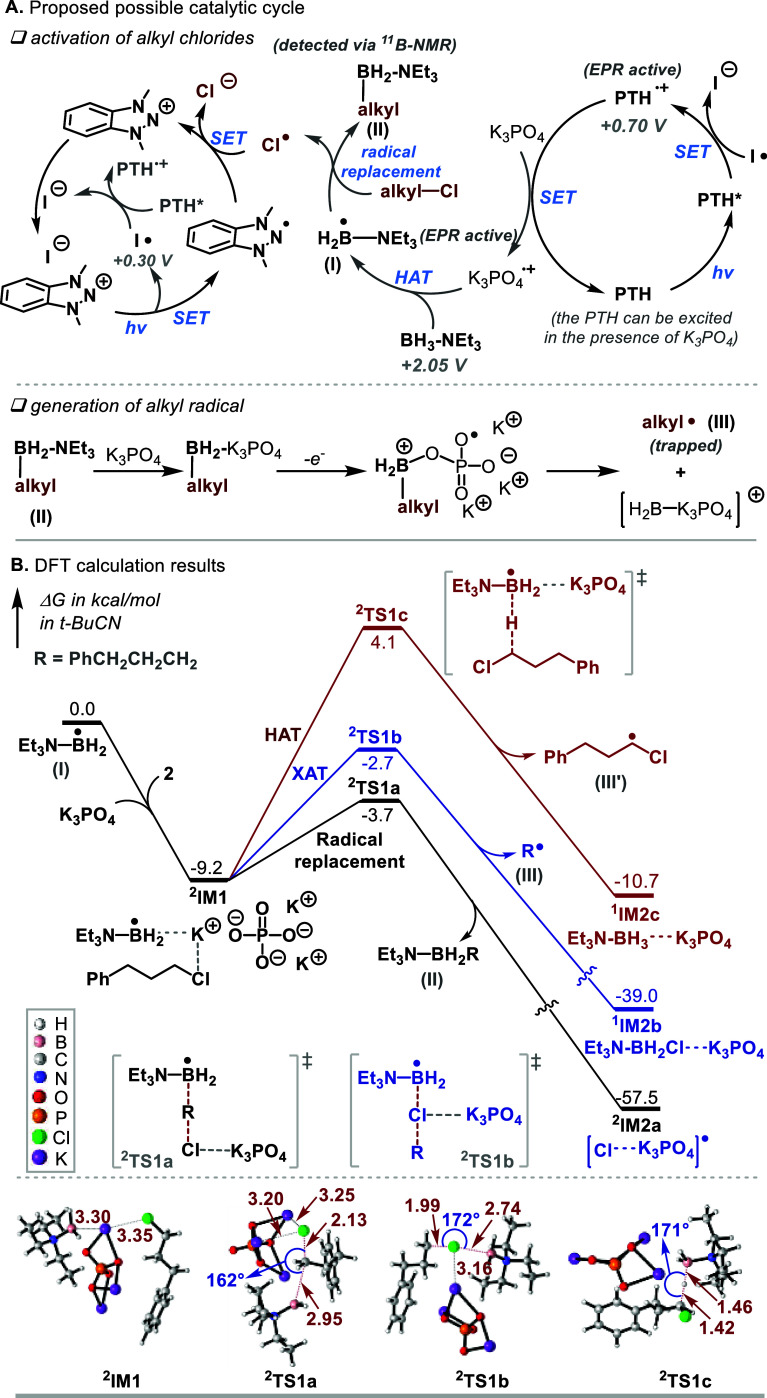
(A) Proposed possible catalytic cycle. (B) Comparing the energetics of the three processes of the LBR. The bond lengths are given in angstroms and bond angles in degrees. Conformational searches were conducted for ^**2**^**TS1a** and ^**2**^**TS1b**, respectively, each of which is the lowest among the ten conformers located (Figure S21).

Based on these mechanistic studies and existing reports, we propose a plausible mechanism in Figure 4A. The NHN **A1** salt is excited by blue light, promoting a SET process to generate NHN^•^ and I^•^ radicals. The I^•^ radical (*E*_ox_ = +0.30 V vs SCE) could oxidize the excited PTH* to its oxidative state PTH^•+^ (*E*_ox_ = +0.67 V vs SCE). Then, PTH^•+^ oxidizes K_3_PO_4_ to K_3_PO_4_^•+^, which then undergoes HAT with BH_3_–NEt_3_ to generate the corresponding LBR **I**.^26^ Subsequent radical replacement process between LBR **I** with unactivated alkyl chloride produces alkyl borane **II**. Subsequently, the generated alkyl-BH_2_–K_3_PO_4_ from alkyl borane **II** undergoes one-electron oxidation process for the generation of alkyl radical (see Figure S19 for DFT calculation results to support the process). The generated alkyl radical then reacts with an alkene, leading to an alkyl radical and subsequent redox-neutral radical cascade cyclization or anionic processes (protonation and E1cB),^20b^ yielding the desired products (See Figure S20 for the details).

There has been no precedent for LBR undergoing radical replacement process with alkyl halides. To corroborate the feasibility of the process, we carried out density functional theory (DFT) calculations to study the reaction of LBR **I** with unactivated alkyl chloride **2** (see Supporting Information (SI) for computational details). As depicted in Figure 4B, the process starts with the formation of a ternary complex ^**2**^**IM1** among LBR **I**, K_3_PO_4_, and **2**. Subsequently, ^**2**^**IM1** could undergo radical replacement reaction (^**2**^**TS1a**), XAT (^**2**^**TS1b**), or HAT (^**2**^**TS1c**) process. The energetic results indicate that the radical replacement process is 1.0 and 7.8 kcal/mol more favorable than the XAT and HAT, respectively. Considering the simplified molecular model used for K_3_PO_4_, the energetic results reasonably account for the preference of the radical replacement mechanism in our system. Supportively, no XAT product (Et_3_N-BH_2_Cl) could be observed in our experimental study.

The formation of a strong B-X bond is the driving force for LBRs to exhibit XAT reactivity.^15^ To understand the driving force for forming alkyl borane in our catalytic system, we further computed the three processes in the absence of K_3_PO_4_ (Figure S22). The calculations show that the barriers for HAT, XAT, and radical replacement are 17.5, 10.3, and 10.4 kcal/mol and the three processes are exergonic by 2.1, 31.2, and 20.1 kcal/mol, respectively. Thus, the presence of K_3_PO_4_ reverses the favorability of the radical replacement compared to XAT in terms of both kinetics and thermodynamics. Consistently, the experimental study demonstrated the crucial role of K_3_PO_4_ for our reactions. Comparing the structures of ^**2**^**TS1a** and ^**2**^**TS1b**, it could be observed the transition state ^**2**^**TS1a** favors the interaction of K_3_PO_4_ with the leaving Cl atom, compared to the XAT transition state ^**2**^**TS1b**.

To further consolidate our proposed mechanism, we used another inorganic base Cs_2_CO_3_, which delivered the desired product in 45% yield (Table S1), to conduct the mechanistic studies. In agreement with the decreased yield, the results indicate that Cs_2_CO_3_ could still play similar roles of K_3_PO_4_ but with inferior energetics in LBR generation and radical replacement (see Figures S4, S11, S15, S16, S18, S23, S24 for details). In addition, because amine-borane BH_3_–NEt_3_ was used as LBR precursor in our reactions, we exclude a possible mechanism involving XAT of the excited LBR.^5k^

## Conclusions

In conclusion, we have developed a new activation mode of LBRs for generating alkyl radicals from unactivated alkyl chlorides. Unlike the XAT mechanism commonly employed by LBRs to activate alkyl iodides, alkyl bromides, and activated alkyl chlorides, this new approach involves radical replacement process to form alkyl borane serving as the intermediate. By addressing the challenge of activating unactivated alkyl chlorides, which has previously impeded progress in LBR chemistry, our study presents an efficient photochemical strategy for forming C(sp^3^)-C(sp^3^) bonds under transition metal-free conditions. This advancement not only expands the synthetic versatility of LBRs but also furnishes organic chemists with a valuable tool for accessing alkyl radicals from readily available and accessible substrates-unactivated alkyl chlorides.

## Supporting Information Available

Experimental details, characterization data, DFT studies, and spectra (PDF)
