# Supplementary material for: Radical Replacement Process for Ligated Boryl Radical-Mediated Activation of Unactivated Alkyl Chlorides for C(sp3)–C(sp3) Bond Formation
Source: J Am Chem Soc. 2024 Sep 12;146(38):26574–84. doi: 10.1021/jacs.4c10915 (PMC11694241; doi:10.1021/jacs.4c10915)
Supplement: Supplementary file 1 — ja4c10915_si_001.pdf [file ja4c10915_si_001.pdf]

Supporting Information

Radical Replacement Process for Ligated Boryl Radical-Mediated Activation of Unactivated Alkyl Chlorides for C(sp^3)-C(sp^3) Bond Formation

Chang-Zhen Fang[a+], Bei-Bei Zhang[a+], Yong-Liang Tu[a], Qiang Liu[a], Zhi-Xiang Wang[a,b*], Xiang-Yu Chen[a,b*]

[a]School of Chemical Sciences, University of Chinese Academy of Sciences (UCAS), Beijing National Laboratory for Molecular Sciences, Beijing 100049, China.

[b]Binzhou Institute of Technology, Weiqiao-UCAS Science and Technology Park, Binzhou, Shandong Province 256606, China.

[+]These authors contributed equally to this work.

*E-mail: zxwang@ucas.ac.cn; chenxiangyu20@ucas.ac.cn;

Table of contents

1. General Information....S3

2. Synthesis of N-Heterocyclic Nitrenium Salts....S6

3. General Procedure and the Optimization of the Reaction Conditions....S7

4. UV/vis Absorption Spectra Experiments....S10

5. EPR Spectroscopy Experiments....S12

6. Alkyl borane as an alkyl radical precursor....S17

7. Radical Trapping Experiments....S18

8. ^{11}B NMR spectra studies....S20

9. Conversion of the alkyl borane by oxidation....S22

10. Compound Characterization Data....S24

11. NMR Spectra....S40

12. Computational details and additional results....S98

13. References....S142

1. General Information

- Chemicals were purchased from Heowns, Innochem, or Bidepharm and used without further purification unless otherwise noted. Solvents were purified using a solvent-purification system (VSPS-8, Vigor) that contained activated alumina and molecular sieves. N-arylacrylamides,[1,2] enamides,[3] α-CF_3 alkenes,[4] and nitrenium salts[5,6] were prepared according to literature methods.
- Analytical thin layer chromatography was carried out with silica gel pre-coated glass plates (TLC-Silica gel GF254, coating thickness: 0.25 mm) purchased from Xinnuo Chemical (Yantai, China). Chromatographic purification of the products was performed on silica gel 200-300 mesh. Visualization of the developed TLC plates was performed with ultraviolet irradiation (254 nm) or by staining with a basic potassium permanganate solution.
- High-resolution mass spectra (HRMS) were obtained with the mass analyzer of an orbitrap. The calculated values are based on the most abundant isotope.
- IR spectra were taken on a Vertex 70 spectrophotometer and reported as wave numbers (cm^{-1}).
- The WRS-2 microcomputer melting point meter was used to measure the melting point of solids.
- The GC-MS TQ8040 was used in the detection of the reaction mixture.
- UV-vis absorption spectra were acquired on a UV-2600 spectrophotometer (Shimadzu, Japan).
- Cyclic voltammetry studies were carried out on a Shanghai Chen Hua CHI660E electrochemical workstation.
- EPR spectra were recorded at room temperature, using a Bruker EMXplus spectrometer.
- ^{1}H, ^{13}C NMR, ^{19}F NMR, and ^{11}B NMR spectra were recorded at ambient temperature on a JNM-ECZ-400S/500S/600R Spectrometer. The chemical shifts are reported in ppm downfield of tetramethylsilane (TMS) and referenced to residual solvent peaks resonance as an internal standard. The order of citation in parentheses is a) multiplicity (s = singlet, d = doublet, t = triplet, q = quartet, dd= doublet of doublet, ddd= doublet of doublet of doublet, td = triplet of doublet, m = multiplet, bs = broad signal), b) coupling constants, c) number of protons. Coupling constants (*J*) are reported in Hertz (Hz).

- Photochemical experiments were performed magnetically stirred in 10 mL glass tubes, sealed with a rubber septum. The tubes were irradiated with blue light (Kelo-AO100S (450nm) or PLS-LED100C(465nm)) using a LED lamp with a power output of 100 W (see below pictures). The distance from the light source to the irradiation vessel is 2.0 cm to keep the reaction temperature at 60 ± 5 °C.
- (The purchase link of Kelo-AO100S blue LED is https://item.taobao.com/item.htm?spm=a230r.1.14.90.19013f311BIQEz&id=548675812368&ns=1&abbucket=0#detail. The purchase link of PLS-LED100C blue LED (465 nm) is https://www.perfectlight.cn/Product/detail/id/32.html).

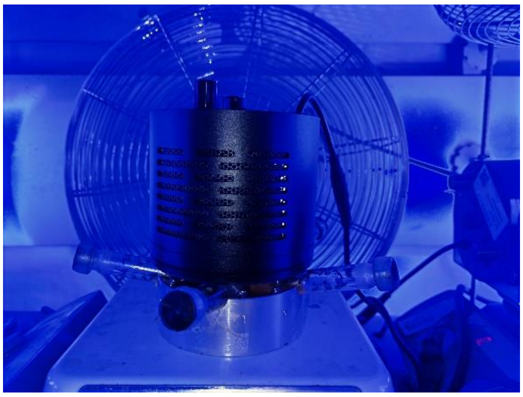

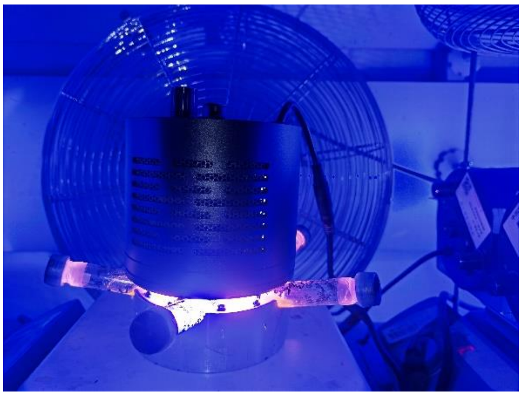

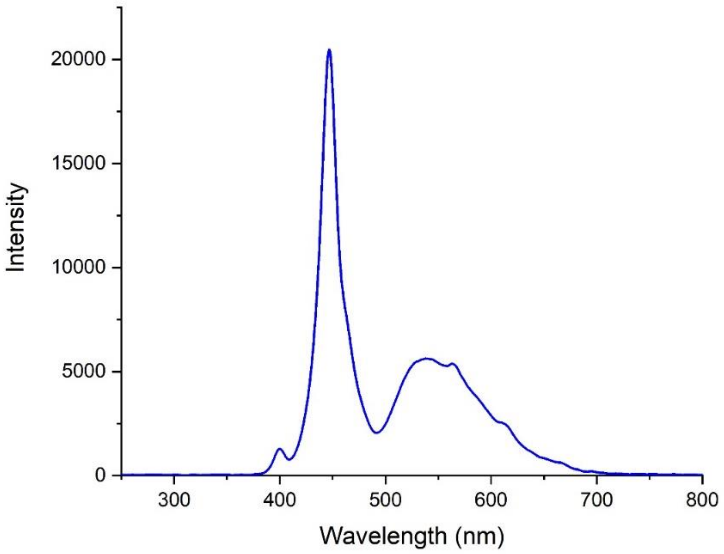

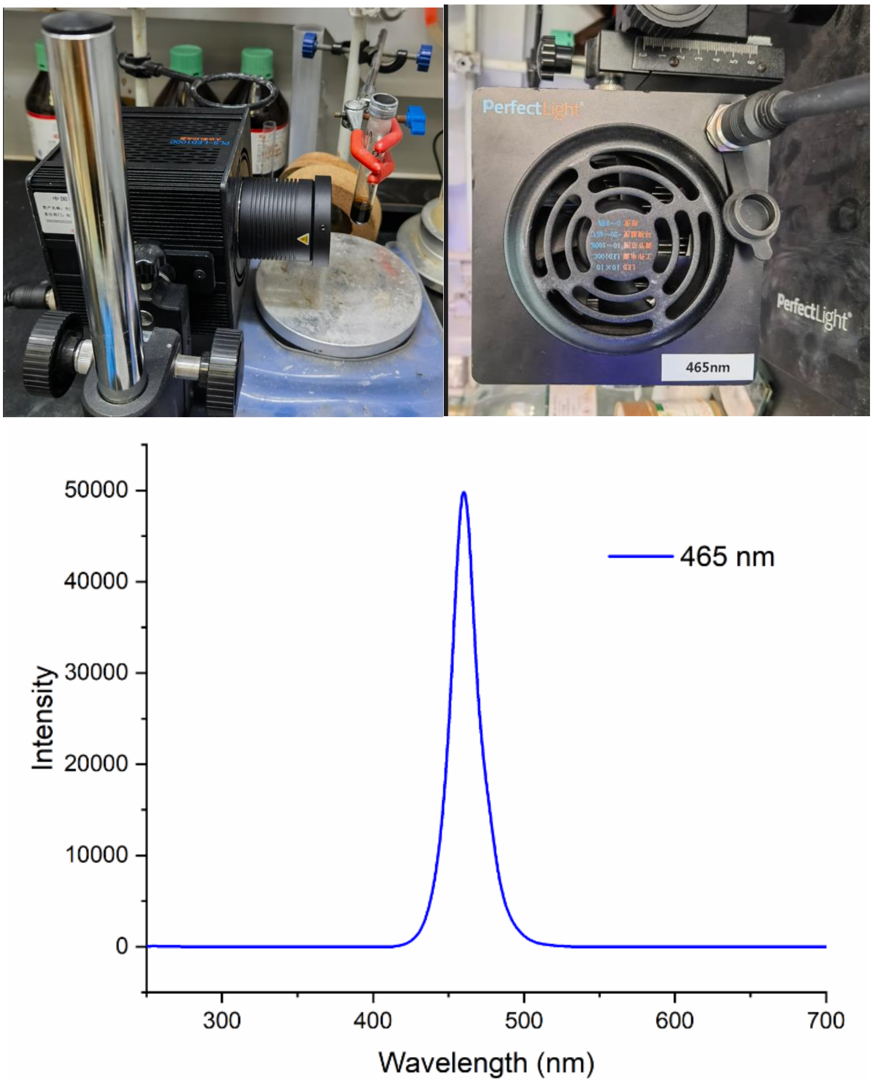

Figure S1. The spectrum of blue LEDs (Kelo-AO100S (450nm) and PLS-LED100C(465nm)) employed in the reaction

2. Synthesis of N-Heterocyclic Nitrenium Salts

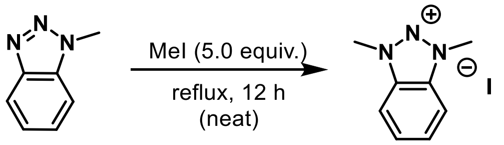

The preparation of nitrenium salt **A1-A3** was performed according to the previously reported procedure[5]. 1-methylbenzotriazole (266.0 mg, 2.0 mmol, 1.0 equiv.) was dissolved in methylating reagent (Me-X, 10.0 mmol, 5.0 equiv.) which was warmed to reflux for 12 h. After cooling to room temperature, the removal of alkyl iodide and recrystallization of the crude crystals from ethanol led to nitrenium salt.

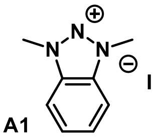

A1

^{1}H NMR (400 MHz, Methanol-*d*$_4$) δ 8.30 – 8.25 (m, 2H), 8.04 – 7.94 (m, 2H), 4.65 (s, 6H). **^{13}C NMR** (101 MHz, Methanol-*d*$_4$) δ 135.6, 131.1, 113.5, 37.4. **IR** (ATR): 2981, 1603, 1437, 1313, 1012, 774 cm^{-1} **HRMS** (ESI)**:** *m/z* [M]$^+$ calcd for $C_8H_{10}N_3^+$:148.0869; found 148.0866. **Melting Point:** 184 - 186 °C. These data are in agreement with those reported previously in the literature[5].

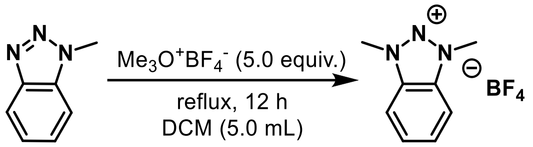

A3

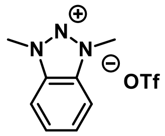

A2

^{1}H NMR (400 MHz, Methanol-*d*$_4$) δ 8.24 – 8.18 (m, 2H), 7.99 – 7.93 (m, 2H), 4.61 (s, 6H). **^{13}C NMR** (101 MHz, Methanol-*d*$_4$) δ 135.5, 131.0, 120.5 (q, *J* = 323.2 Hz), 113.3, 36.9. **^{19}F NMR** (471 MHz, Methanol-*d*$_4$) δ -79.99. **IR** (ATR): 2988, 1439, 1323, 1265, 1023, 775 cm^{-1}

HRMS (ESI)**:** *m/z* [M]$^+$ calcd for $C_8H_{10}N_3^+$:148.0869; found 148.0867. **Melting Point:** 125 - 127 °C.

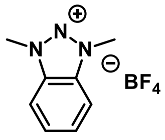

A3

^{1}H NMR (400 MHz, D_2O) δ 7.99 (dd, J = 6.6, 3.1 Hz, 1H), 7.84 (dd, J = 6.6, 3.1 Hz, 1H), 4.47 (s, 3H). **^{13}C NMR** (101 MHz, Methanol-*d*$_4$) δ 135.3, 131.2, 113.1, 37.2. **^{19}F NMR** (471 MHz, Methanol-*d*$_4$) δ -150.6 (major), -150.5 (minor). **IR** (ATR): 2987, 2911, 1092, 1039, 1017, 779, 521 cm^{-1}.

HRMS (ESI)**:** *m/z* [M]$^+$ calcd for $C_8H_{10}N_3^+$:148.0869; found 148.0867. **Melting Point:** 169 - 170 °C.

3. General Procedure and the Optimization of the Reaction Conditions

Table S1. Optimization of the reaction conditions

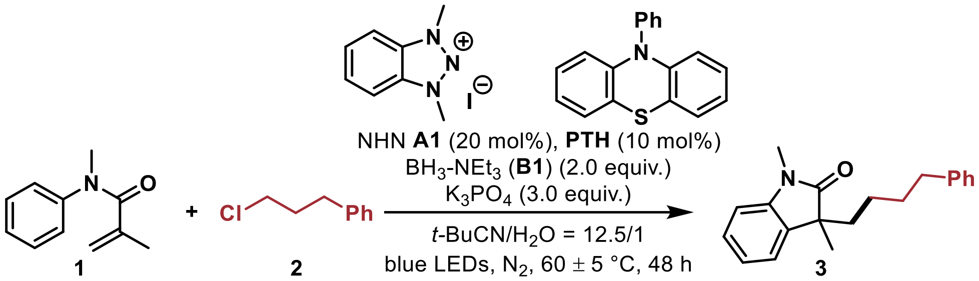

Entry	Variations from standard conditions	Yield (%)[a]
1	none	84
2	without NHN **A1**	trace
3	NaI instead of NHN **A1**	trace
4	without BH_3-NEt_3	8
5	without PTH	17
6	without K_3PO_4	trace
7	without H_2O	trace
8	without BH_3-NEt_3 and K_3PO_4	trace
9	without BH_3-NEt_3 and PTH	trace
10	without K_3PO_4 and PTH	trace
11	without K_3PO_4 and H_2O	trace
12	without PTH and H_2O	trace
13	without BH_3-NEt_3, PTH, K_3PO_4, and H_2O	NR
14	without BH_3-NEt_3, PTH, and K_3PO_4	trace
15	without BH_3-NEt_3, PTH, and H_2O	trace
16	without PTH, K_3PO_4, and H_2O	trace
17	without BH_3-NEt_3, K_3PO_4, and H_2O	trace
18	without irradiation, 60 ^{o}C	NR

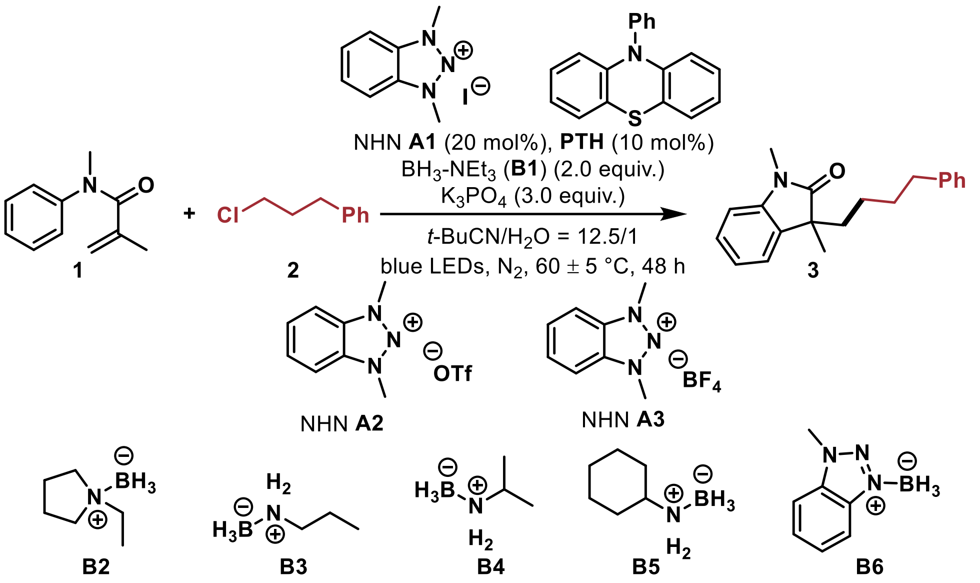

Entry	Variations from standard conditions	Yield (%)[a]
1	none	84
2	NHN **A2** instead of **A1**	trace
3	NHN **A3** instead of **A1**	trace
4	4CzIPN instead of **PTH**	67
5	Ir[(dF(CF$_3$)ppy)$_2$(CF$_3$bpy)]PF$_6$ instead of **PTH**	63
6	DMF instead of TMA	52
7	DMA instead of TMA	54
8	DMSO instead of TMA	54
9	NMP instead of TMA	50
11	CH$_3$CN instead of TMA	9
12	THF instead of TMA	trace
13	EtOH instead of TMA	trace
14	**B2** instead of BH$_3$-NEt$_3$	trace
15	**B3** instead of BH$_3$-NEt$_3$	trace
16	**B4** instead of BH$_3$-NEt$_3$	trace
17	**B5** instead of BH$_3$-NEt$_3$	14
18	**B6** instead of BH$_3$-NEt$_3$	15
19	NEt$_3$ instead of K$_3$PO$_4$	8
20	TMEDA instead of K$_3$PO$_4$	4
21	DIPEA instead of K$_3$PO$_4$	6
22	DBU instead of K$_3$PO$_4$	trace
23	DMAP instead of K$_3$PO$_4$	trace
24	DABCO instead of K$_3$PO$_4$	trace
25	Na$_2$CO$_3$ instead of K$_3$PO$_4$	30
26	K$_2$CO$_3$ instead of K$_3$PO$_4$	51
27	Cs$_2$CO$_3$ instead of K$_3$PO$_4$	45
28	KOAc instead of K$_3$PO$_4$	38

Reaction condition: **1** (0.2 mmol), **2** (1.0 mmol), NHN **A1** (20 mol%), PTH (10 mol%), BH$_3$-NEt$_3$ (0.4 mmol), K$_3$PO$_4$ (0.6 mmol), H$_2$O (80 μL) and *t*-BuCN (TMA) (1.0 mL). [a]Yields of isolated product after chromatography.

General procedure A:

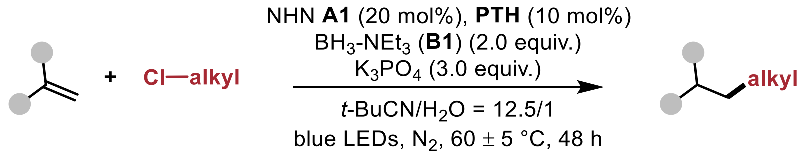

In a nitrogen-filled glove box, the alkenes (0.2 mmol, 1 equiv.), NHN **A1** (0.04 mmol, 10.5 mg, 20 mol%), PTH (0.02 mmol, 5.5 mg, 10 mol%), and K_3PO_4 (0.6 mmol, 127.2 mg, 3 equiv.) were added sequentially into a 10 mL dry tube, then *t*-BuCN (1.0 mL), Et_3N-BH_3 (0.4 mmol, 46 mg, 2 equiv.) and alkyl chloride (1 mmol, 5 equiv.) were added by syringe. The vial was closed and removed from the glove box, then H_2O (80 μL) was added by syringe, and the resulting mixture was allowed to stir under blue LED irradiation for 48 h, where the distance from the light source to the irradiation vessel is 1.5 cm to keep the reaction temperature at 60 ± 5 °C. Upon completion, the solvent was removed under vacuum and the residue was subjected to silica gel chromatography using petroleum ether and ethyl acetate as eluent to afford the desired product.

General procedure B

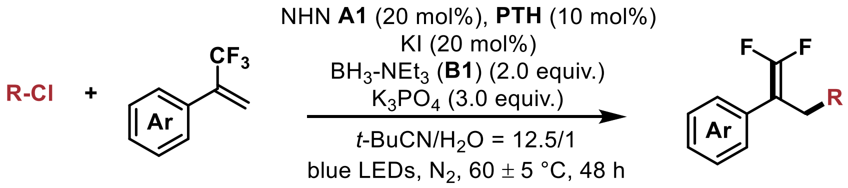

In a nitrogen-filled glove box, the NHN **A1** (0.04 mmol, 10.5 mg, 20 mol%), PTH (0.02 mmol, 5.5 mg, 10 mol%), K_3PO_4 (0.6 mmol, 127.2 mg, 3 equiv.) and KI (0.04 mmol, 6.6 mg, 20 mol%) were added sequentially into a 10 mL dry tube, then the *t*-BuCN (1.0 mL), alkenes (0.2 mmol, 1 equiv.), Et_3N-BH_3 (0.4 mmol, 46 mg, 2 equiv.) and alkyl chloride (1 mmol, 5 equiv.) were added by syringe. The vial was closed and removed from the glove box, then H_2O (80 μL) was added by syringe, and the resulting mixture was allowed to stir under blue LED irradiation for 48 h, where the distance from the light source to the irradiation vessel is 1.5 cm to keep the reaction temperature at 60 ± 5 °C. Upon completion, the solvent was removed under vacuum and the residue was subjected to silica gel chromatography using petroleum ether and ethyl acetate as eluent to afford the desired product.

4. UV/vis Absorption Spectra Experiments

UV/vis absorption spectra were recorded using *t*-BuCN as solvent in 1 cm path quartz cuvettes using a UV-2600 UV/Vis spectrometer.

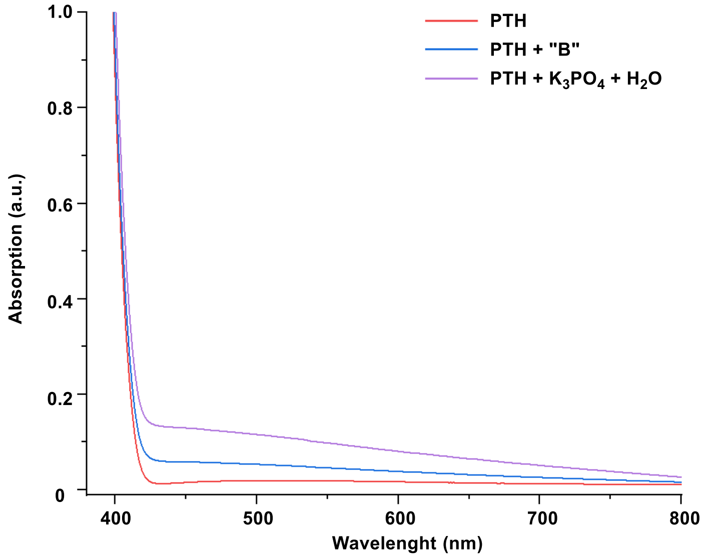

Figure S2. The UV/vis spectra of PTH (0.02 M in *t*-BuCN), and its mixtures with Et_3N-BH_3 (0.4 M in *t*-BuCN), K_3PO_4 (0.6 M in *t*-BuCN) and H_2O (8% (v/v) in *t*-BuCN).

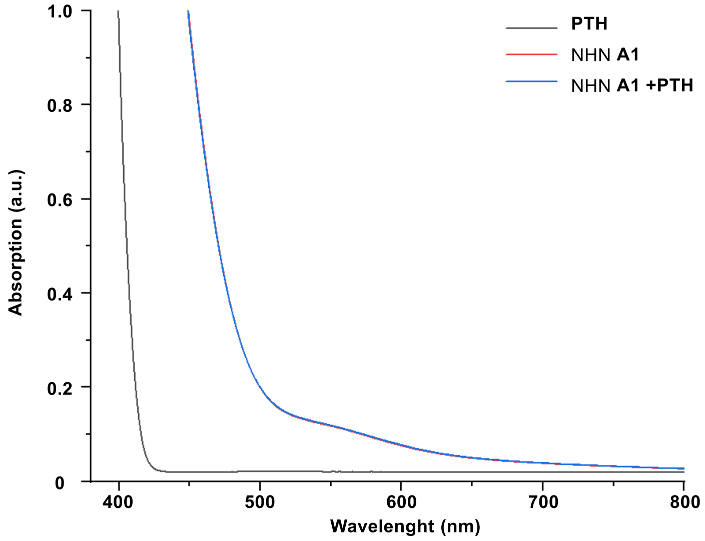

Figure S3. The UV/vis spectra of PTH (0.02 M in *t*-BuCN), NHN **A1** (0.04 M in *t*-BuCN), and their mixture was tested.

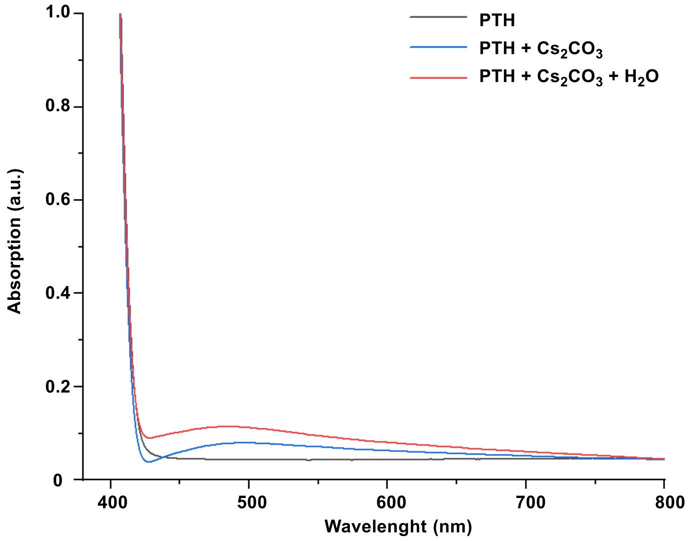

Figure S4. The UV/vis spectra of PTH (0.02 M in *t*-BuCN), and its mixtures with Cs_2CO_3 (0.6 M in *t*-BuCN) and H_2O (8% (v/v)).

5. EPR Spectroscopy Experiments

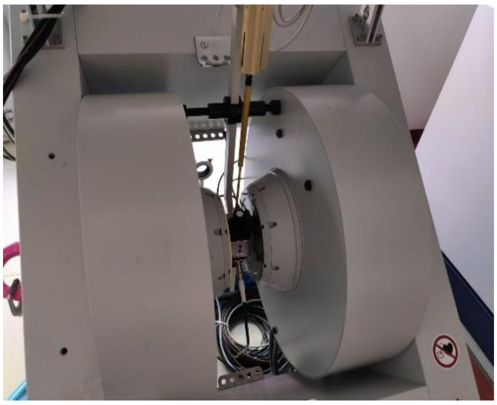

Continuous-wave (CW) electron paramagnetic resonance (EPR) measurements were performed on acetonitrile solution in capillary tubes at ambient temperature with a Bruker EMXplus spectrometer at microwave frequencies of about 9.85 GHz. A Bruker super-high Q resonator (ER 4119HS) with slits in the cavity wall for optical excitation was used. As a light source, a 100 W blue LED (λ = 465 nm, PLS-LED100C) was placed outside the magnet 40 cm from the cavity wall. CW EPR spectra were measured with a microwave power of 2.518 mW and a modulation amplitude of 0.1 mT in order to avoid line broadening by saturation or overmodulation.

Characterization of the PTH radical adduct

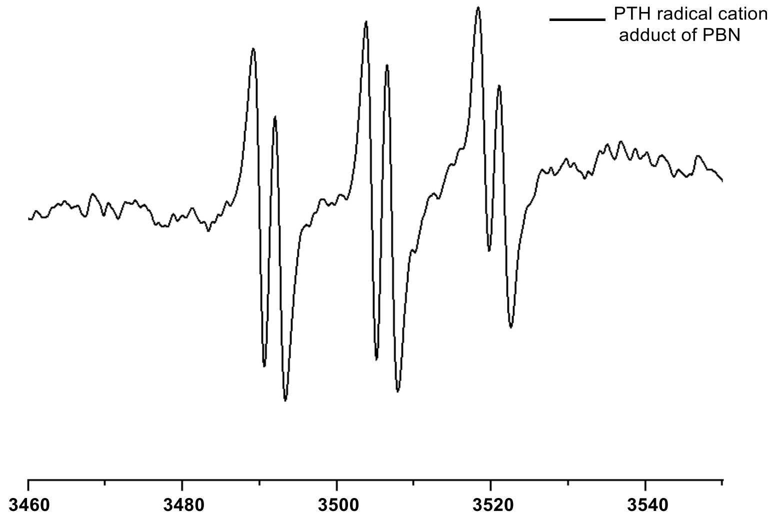

Figure S5. EPR spectrum of the prepared PTH radical adduct.

The solution of phenothiazine (0.02 mmol, 5.5 mg, 1 equiv.) with dibenzoyl peroxide (0.06 mmol 14.5 mg, 3 equiv.) in *t*-BuCN was degassed under N_2, then PBN (n-benzylidene-tert-butylamineoxide, 1 equiv.) was added under N_2. The PTH radical adduct was detected from the solution.[7] The EPR spectrum was shown in Figure S5.

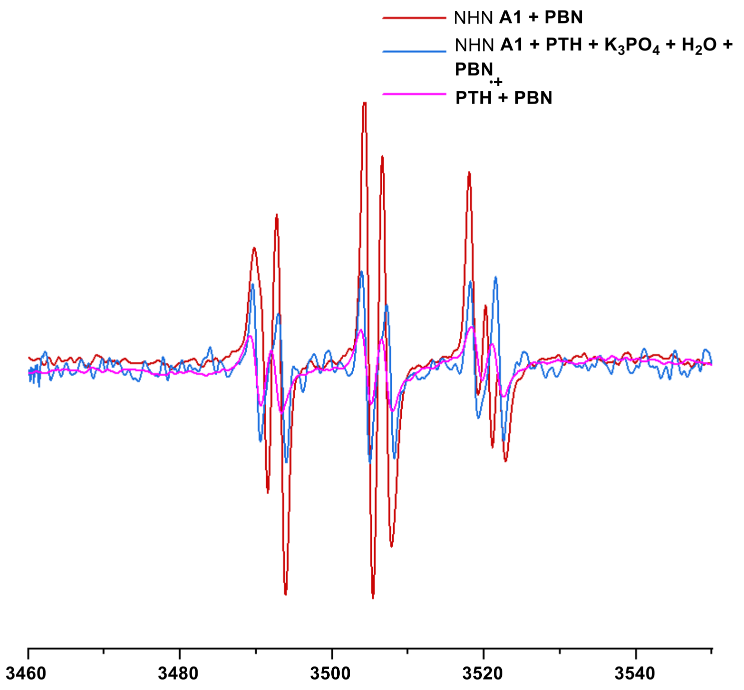

Figure S6. EPR spectrum of the PTH radical adduct in the reaction system.

In a nitrogen-filled glove box, NHN **A1** (0.04 mmol, 10.5 mg, 20 mol%), PTH (0.02 mmol, 5.5 mg, 10 mol%), and PBN (0.6 mmol, 106.3 mg, 3 equiv.) were added to dry *t*-BuCN (1 mL). Then the solution was added to a 3 mm EPR tube and sealed. The PTH radical adduct was detected upon the irradiation of blue light (λ = 465 nm, PLS-LED100C). The EPR spectrum was shown in Figure S6.

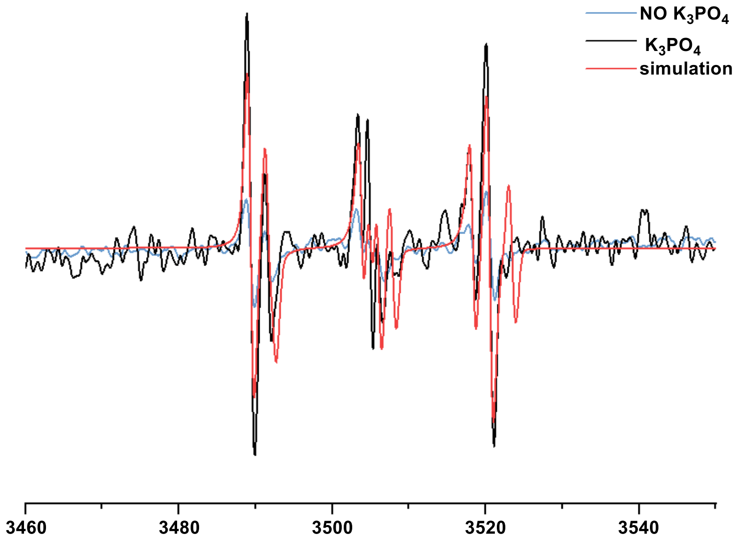

Figure S7. EPR spectrum of the Ph• radical adduct in the reaction system.

In a nitrogen-filled glove box, PTH (0.02 mmol, 5.5 mg, 10 mol%), PhI (0.2 mmol, 22.3 μL), K_3PO_4

(0.6 mmol, 127.2 mg, 3 equiv.) and PBN (0.6 mmol, 53.2 mg, 3 equiv.) were added sequentially into a 10 mL dry tube, then *t*-BuCN (1 mL) were added by syringe. The vial was closed and removed from the glove box, and then H_2O (80 μL) was added by syringe. Then the solution was added to a 3 mm EPR tube and sealed. The Ph• radical adduct was detected upon the irradiation of blue light (λ = 465 nm, PLS-LED100C). The EPR spectrum was shown in Figure S7. One signal was PTH radical adduct, and the other was Ph• radical adduct. Their g-values are 2.0066 and 2.0056[8].

The hyperfine couplings of the PTH radical adduct are $^{\alpha}N$ = 14.33 G and $^{\alpha}H$ = 3.35 G (Figure S7) and the hyperfine couplings of the PBN radical adduct are $^{\alpha}N$ = 15.60 G and $^{\alpha}H$ = 2.90 G (Figure S7)[8].

Characterization of the amine-borane radical adduct.

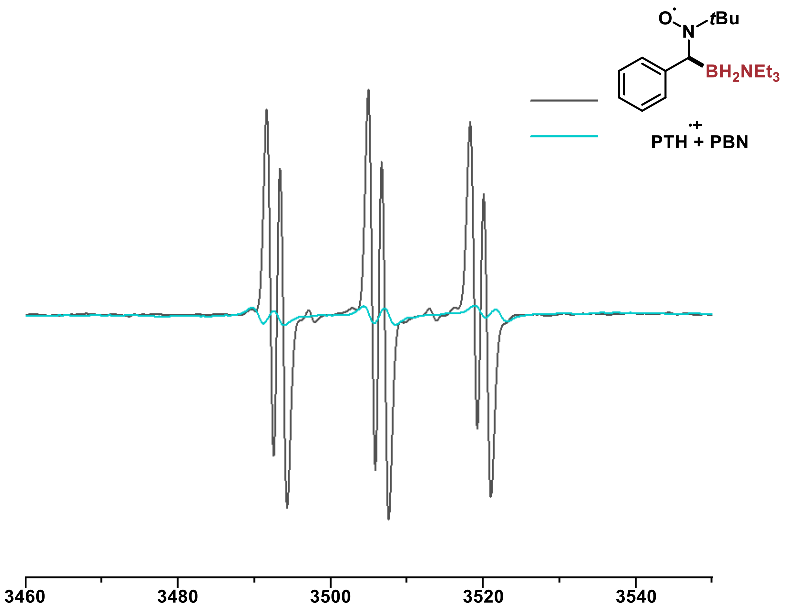

Figure S8. EPR spectrum of the PTH radical adduct and amine-borane radical adduct.

The amine-borane radical adduct was detected from the mixture of dibenzoyl peroxide (0.1 mmol, 24.2 mg, 1 equiv.), BH_3-NEt_3 (0.1 mmol, 12.5 mg, 1 equiv.) and PBN (1 equiv.) without the presence of blue light. The EPR spectrum was shown in Figure S8.

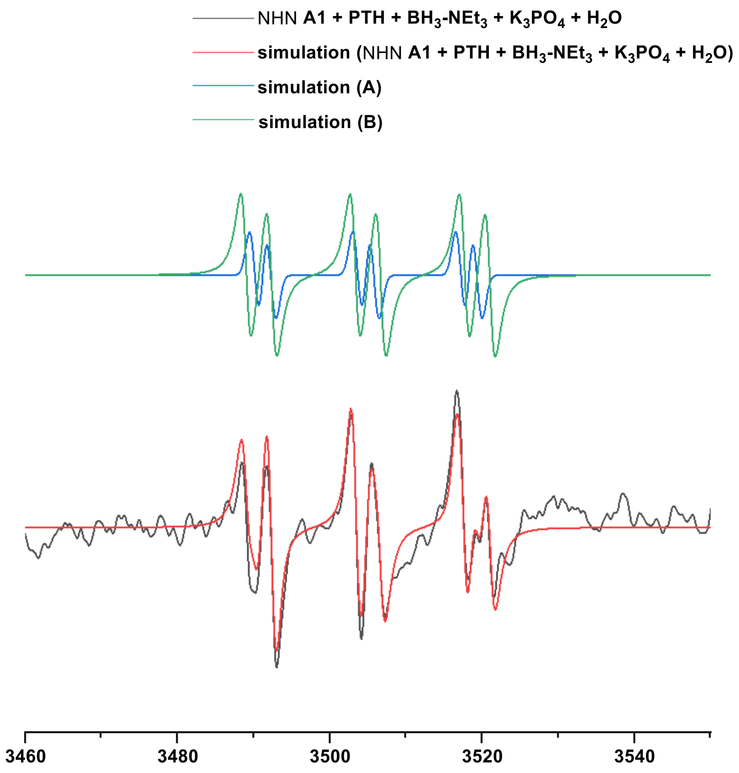

Figure S9. EPR spectrum of the PTH radical adduct and BH_3-NEt_3 radical adduct in the reaction system.

In a nitrogen-filled glove box, NHN **A1** (0.04 mmol, 10.5 mg, 20 mol%), PTH (0.02 mmol, 5.5 mg, 10 mol%), K_3PO_4 (0.6 mmol, 127.2 mg, 3 equiv.), and PBN (0.6 mmol, 53.2 mg, 3 equiv.) were added sequentially into a 10 mL dry tube, then *t*-BuCN (1 mL), and BH_3-NEt_3 (0.4 mmol, 46 mg, 2 equiv.) were added by syringe. The vial was closed and removed from the glove box, and then H_2O (80 μL) was added by syringe. The solution was added to a 3 mm EPR tube and sealed. The radical adducts were detected from the solution upon the irradiation of blue light (λ = 465 nm, PLS-LED100C). The EPR spectrum was shown in Figure S9. One signal was PTH radical adduct, and the other was amine-borane radical adduct. Their g-values are 2.0066 and 2.0064.

The hyperfine couplings of the PTH radical adduct are $^{\alpha}N$ = 14.33 G and $^{\alpha}H$ = 3.35 G (Figure S9) and the hyperfine couplings of the PBN radical adduct are $^{\alpha}N$ = 13.53 G and $^{\alpha}H$ = 2.12 G (Figure S9). After the light was switched off, a weak signal was still detectable in the first scan, but not thereafter.

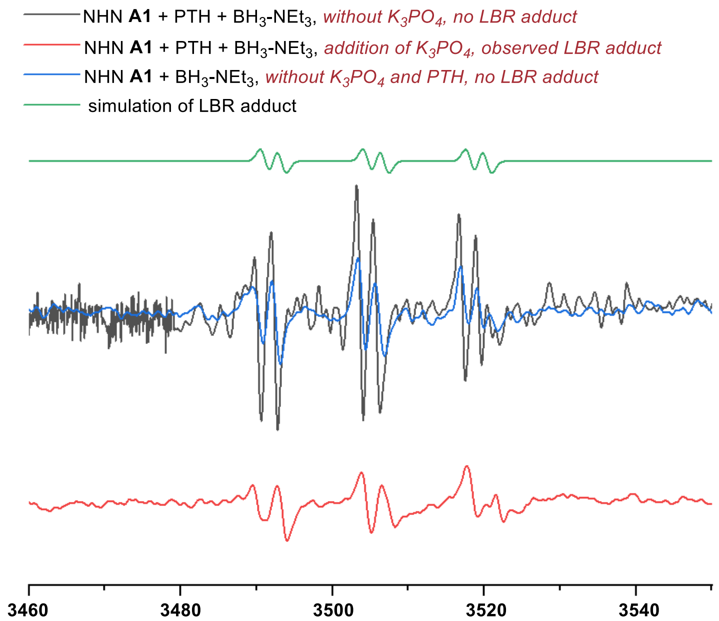

Figure S10. EPR spectrum of the PTH radical adduct and BH_3-NEt_3 radical adduct in the reaction system.

In a nitrogen-filled glove box, NHN **A1** (0.04 mmol, 10.5 mg, 20 mol%), PTH (0.02 mmol, 5.5 mg, 10 mol%), K_3PO_4 (0.6 mmol, 127.2 mg, 3 equiv.), and PBN (0.6 mmol, 53.2 mg, 3 equiv.) were added sequentially into a 10 mL dry tube, then *t*-BuCN (1 mL), and BH_3-NEt_3 (0.4 mmol, 46 mg, 2 equiv.) were added by syringe. The vial was closed and removed from the glove box, and then H_2O (80 μL) was added by syringe. The solution was added to a 3 mm EPR tube and sealed. The radical adduct was detected from the solution upon the irradiation of blue light (λ = 465 nm, PLS-LED100C). The EPR spectrum was shown in Figure S10.

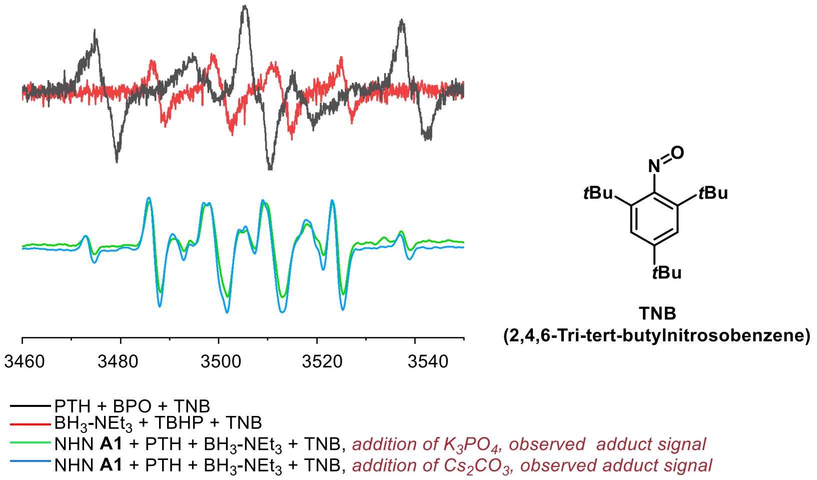

Figure S11. EPR spectrum of the PTH radical adduct and BH_3-NEt_3 radical adduct in the reaction system.

The solution of phenothiazine (0.02 mmol, 5.5 mg, 1 equiv.) with dibenzoyl peroxide (0.06 mmol 14.5 mg, 3 equiv.) in *t*-BuCN was degassed under N_2, then TNB (2,4,6-Tri-tert-butylnitrosobenzene, 1 equiv.) was added under N_2. The PTH radical adduct was detected from the solution.[7] The EPR spectrum was shown in Figure S11.

The amine-borane radical adduct was detected from the mixture of tert-Butyl hydroperoxide (70% in H_2O) (0.3 mmol, 3 equiv.), BH_3-NEt_3 (0.1 mmol, 12.5 mg, 1 equiv.) and TNB (1 equiv.) without the presence of blue light. The EPR spectrum was shown in Figure S11.

In a nitrogen-filled glove box, NHN **A1** (0.04 mmol, 10.5 mg, 20 mol%), PTH (0.02 mmol, 5.5 mg, 10 mol%), Cs_2CO_3 (0.6 mmol, 195.5 mg, 3 equiv.), and TNB (0.6 mmol, 165.3 mg, 3 equiv.) were added sequentially into a 10 mL dry tube, then *t*-BuCN (1 mL), and BH_3-NEt_3 (0.4 mmol, 46 mg, 2 equiv.) were added by syringe. The vial was closed and removed from the glove box, and then H_2O (80 μL) was added by syringe. The solution was added to a 3 mm EPR tube and sealed. The radical adduct was detected from the solution upon the irradiation of blue light (λ = 465 nm, PLS-LED100C). The EPR spectrum was shown in Figure S11.

6. Alkyl borane as an alkyl radical precursor

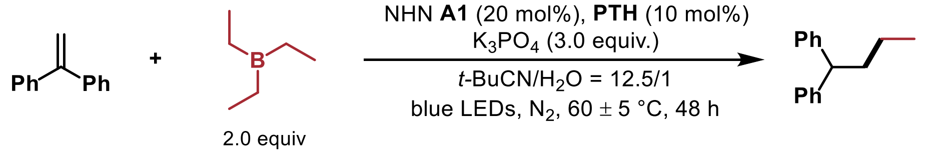

Entry	Variations from standard conditions	Yield
1	none	59%
2	Cs_2CO_3 instead of K_3PO_4	21%
3	without PTH	35%
4	without NHN **A1**	trace
5	without K_3PO_4	trace

Procedure C: In a nitrogen-filled glove box, the NHN **A1** (0.04 mmol, 10.5 mg, 20 mol%), PTH (0.02 mmol, 5.5 mg, 10 mol%), and K_3PO_4 (0.6 mmol, 127.2 mg, 3 equiv.) were added sequentially into a 10 mL dry tube, then the *t*-BuCN (1 mL), 1,1-diphenyl alkene (0.2 mmol, 36.1 mg, 1 equiv.),

and triethyl borane (1 M solution in tetrahydrofuran) (0.4 mmol, 0.4 mL, 2 equiv.) were added by syringe. The vial was closed and removed from the glove box, then H_2O (80 μL) was added by syringe, and the resulting mixture was allowed to stir under blue LED irradiation for 48 h, where the distance from the light source to the irradiation vessel is 1.5 cm to keep the reaction temperature at 60 ± 5 °C. Upon completion, the solvent was removed under vacuum and the residue was subjected to silica gel chromatography using petroleum as eluent to afford the desired product.

7. Radical Trapping Experiments

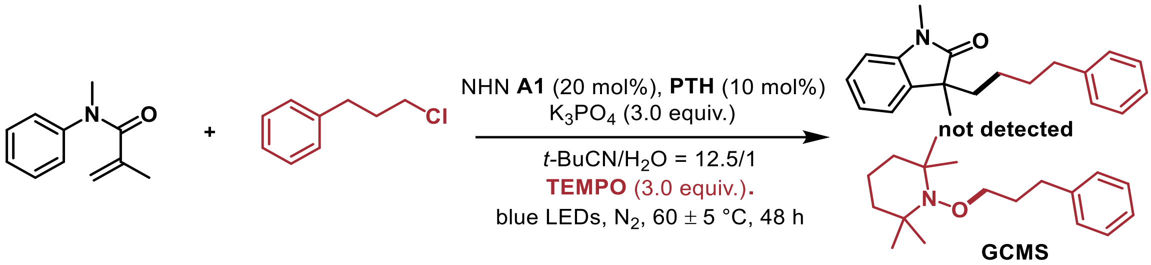

In a nitrogen-filled glove box, the N-arylacrylamide (0.2 mmol, 35.0 mg, 1 equiv.), NHN **A1** (0.04 mmol, 10.5 mg, 20 mol%), PTH (0.02 mmol, 5.5 mg, 10 mol%), K_3PO_4 (0.6 mmol, 127.2 mg, 3 equiv.), and TEMPO (0.6 mmol, 93.6 mg, 3 equiv.) were added sequentially into a 10 mL dry tube, then *t*-BuCN (1 mL), EtN_3BH_3 (0.4 mmol, 46 mg, 2 equiv.) and alkyl chloride (1 mmol, 5 equiv.) were added by syringe. The vial was closed and removed from the glove box, and then H_2O (80 μL) was added by syringe, and the resulting mixture was allowed to stir under blue LED irradiation for 48 h, where the distance from the light source to the irradiation vessel was 1.5 cm to keep the reaction temperature at 60 ± 5 °C. Upon completion, the product of alkyl radical trapped by TEMPO was detected by GCMS.

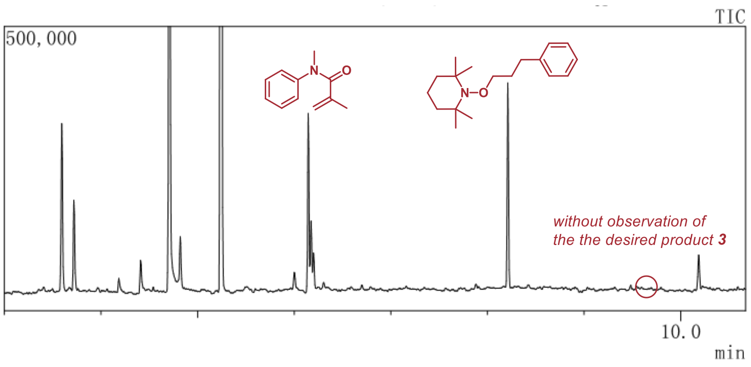

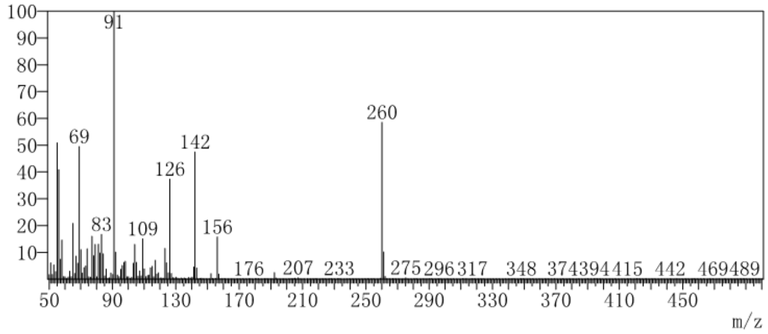

Figure S12. The GCMS result of alkyl radical adduct.

8. ^{11}B NMR spectra studies

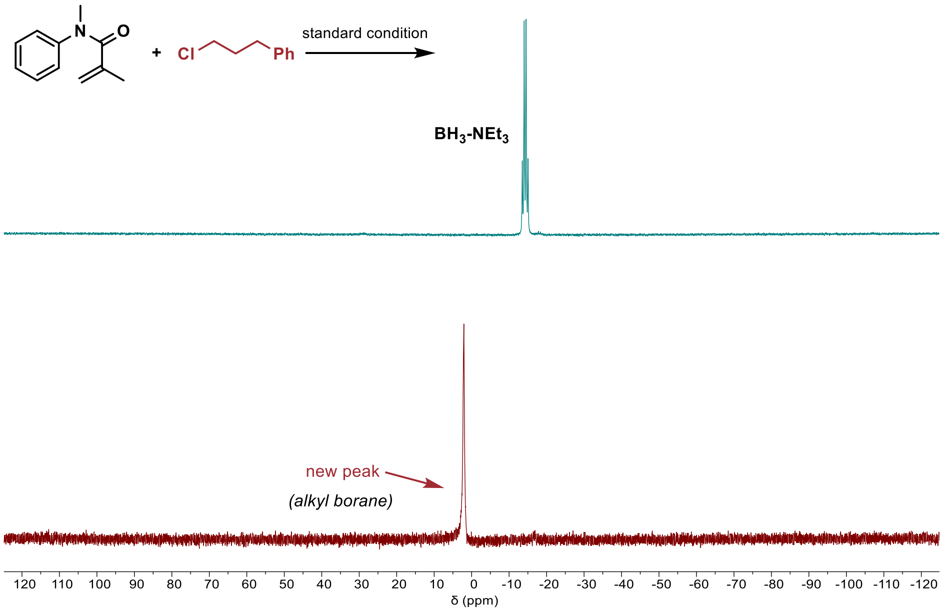

Figure S13. ^{11}B NMR (193 MHz, $CDCl_3$ (top) and D_2O (bottom)) spectra of reaction mixtures were recorded after 48 h.

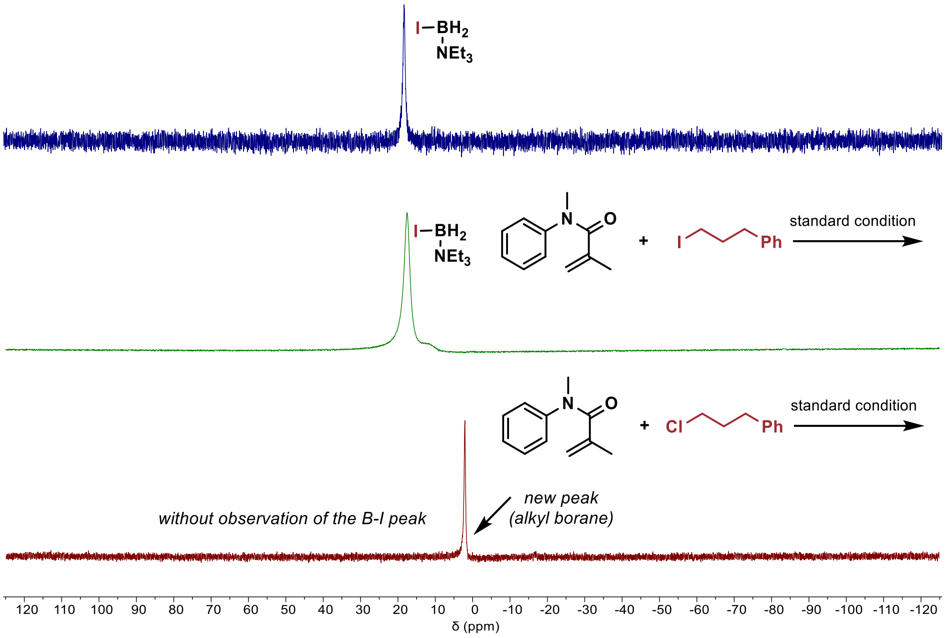

Figure S14. ^{11}B NMR (193 MHz, D_2O) spectra of aqueous phase mixtures were recorded after 48 h.

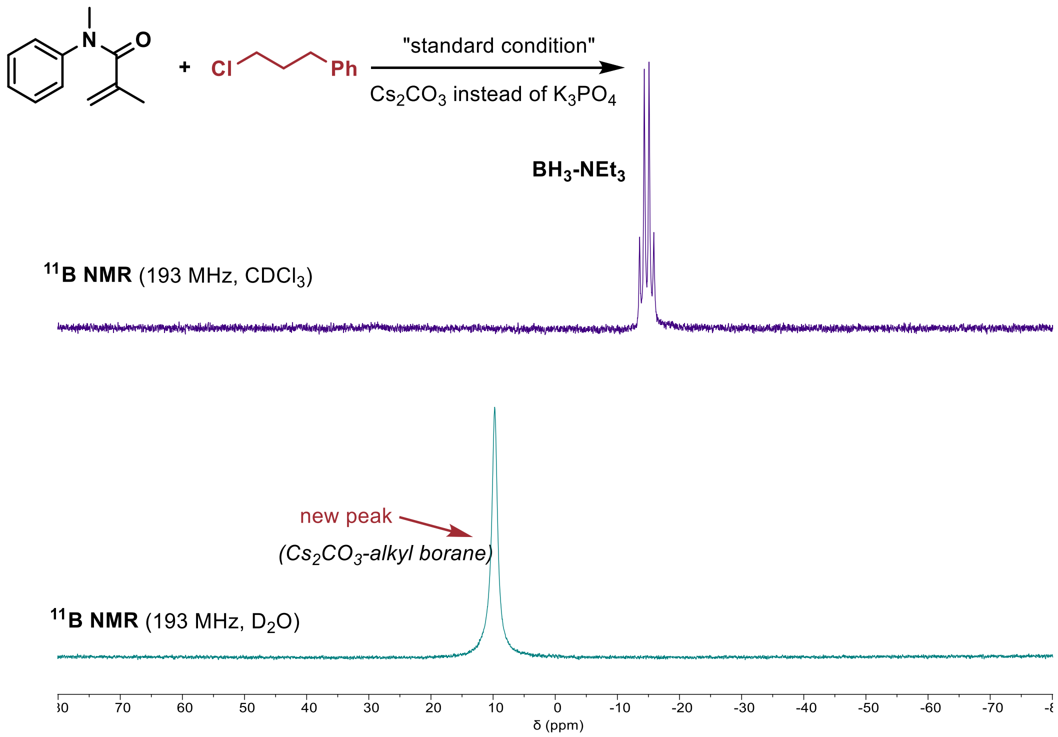

Figure S15. **^{11}B NMR** analysis of the reaction mixture with Cs_2CO_3 as the base.

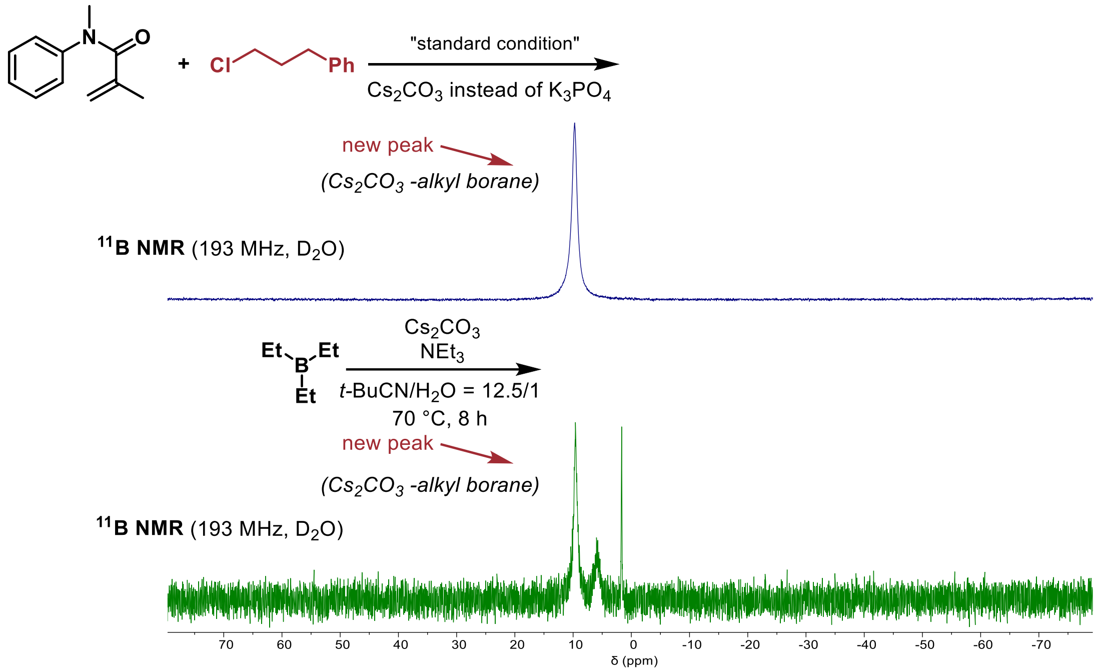

Figure S16. **^{11}B NMR** analysis of the reaction mixture compared with the mixture of BEt_3, Cs_2CO_3, and NEt_3.

9. Conversion of the alkyl borane by oxidation

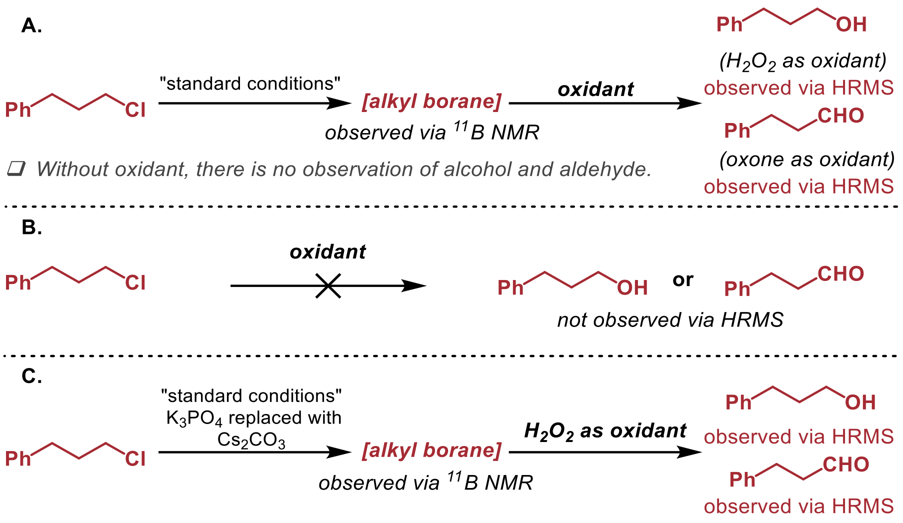

In a nitrogen-filled glove box, NHN **A1** (0.04 mmol, 10.5 mg, 20 mol%), PTH (0.02 mmol, 5.5 mg, 10 mol%), and K_3PO_4 (0.6 mmol, 127.2 mg, 3 equiv.) were added sequentially into a 10 mL dry tube, then *t*-BuCN (1 mL), Et_3N-BH_3 (0.4 mmol, 46 mg, 2 equiv.) and alkyl chloride (0,2 mmol, 30 μL, 1 equiv.) were added by syringe. The tube was closed and removed from the glove box, then H_2O (80 μL) was added by syringe, and the resulting mixture was allowed to stir under blue LED irradiation for 24 h, where the distance from the light source to the irradiation vessel is 1.5 cm to keep the reaction temperature at 60 ± 5 °C.

A1: Upon completion, H_2O_2 (30%) (100 μL, 3 equiv.) were sequentially added. The reaction was warmed to 60 °C and stirred overnight. The mixture was cooled to r.t. and detected by HRMS.

A2: Upon completion, oxone (242 mg, 1.6 mmol, 8 equiv.). THF (1.0 mL) and H_2SO_4 (98%) (38 μL) were sequentially added. The reaction was warmed to 40 °C and stirred overnight. The mixture was cooled to r.t. and detected by HRMS.

B: In a nitrogen-filled glove box, alkyl chloride (0,2 mmol, 30 μL, 1 equiv.) was added by syringe into a 10 mL dry tube. The tube was closed and removed from the glove box, then H_2O (80 μL) was added by syringe, and H_2O_2 (30%) (100 μL, 3 equiv.) was sequentially added. The reaction was warmed to 60 °C and stirred overnight. The mixture was cooled to r.t. and detected by HRMS.

C: Upon completion (Cs_2CO_3 instead of K_3PO_4), H_2O_2 (30%) (100 μL, 3 equiv.) were sequentially added. The reaction was warmed to 60 °C and stirred overnight. The mixture was cooled to r.t. and

detected by HRMS.

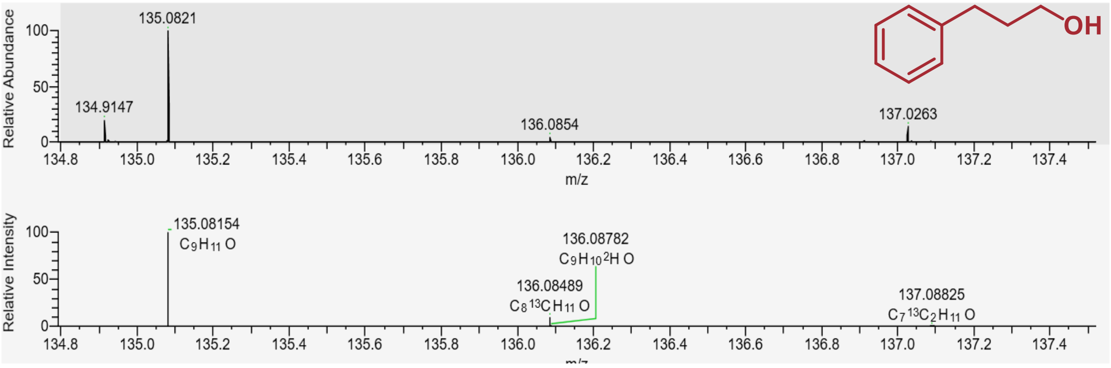

HRMS (ESI)**:** *m/z* [M-H]$^-$ calcd for $C_9H_{11}O^-$: 135.0815; found 135.0821.

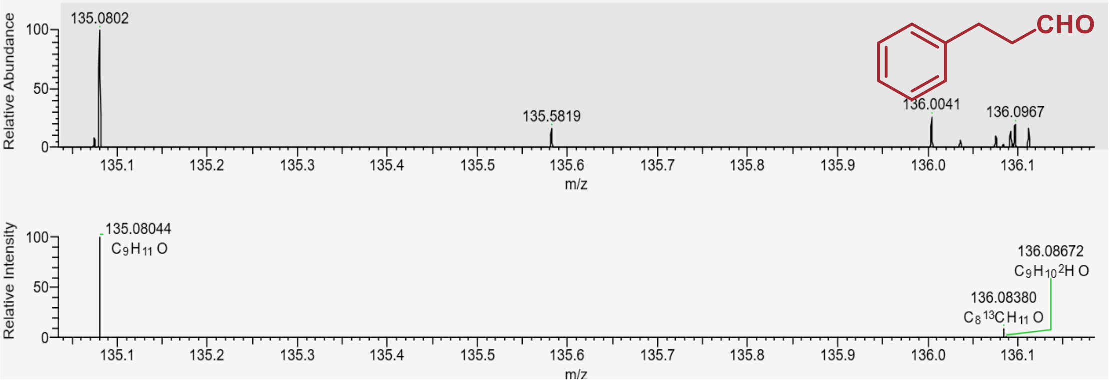

HRMS (ESI)**:** *m/z* [M+H]$^+$ calcd for $C_9H_{11}O^+$: 135.0804; found 135.0802.

Figure S17. HRMS analysis of the reaction mixture with K_3PO_4 as the base.

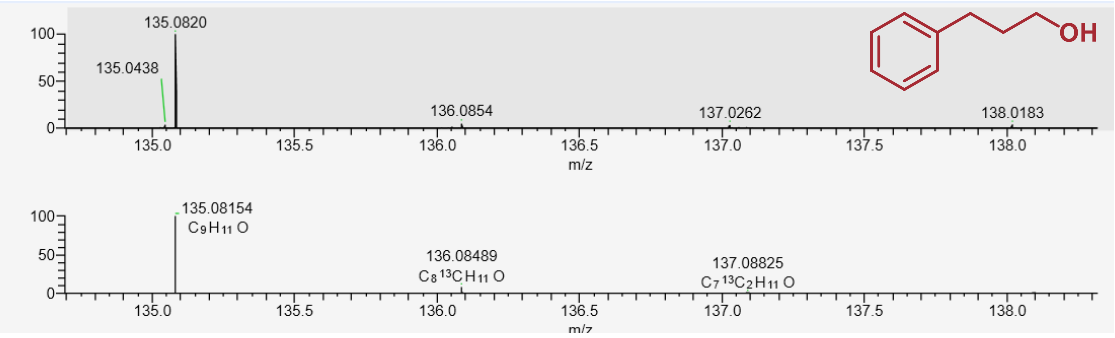

HRMS (ESI)**:** *m/z* [M-H]$^-$ calcd for $C_9H_{11}O^-$: 135.0815; found 135.0820. (Cs_2CO_3 as the base)

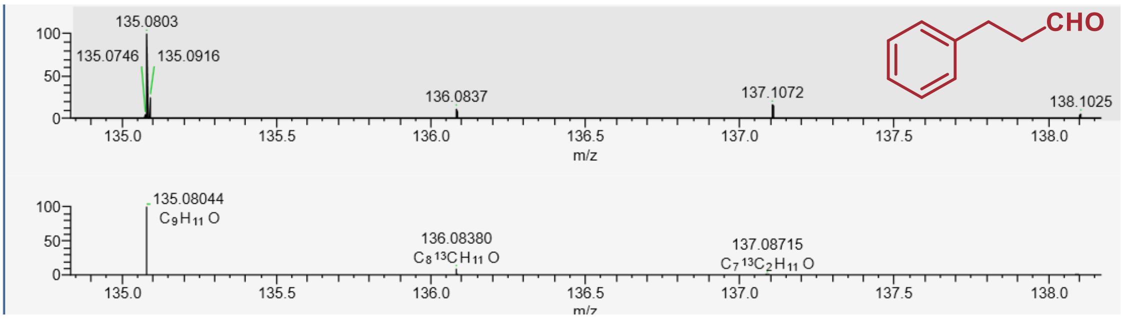

HRMS (ESI)**:** *m/z* [M+H]$^+$ calcd for $C_9H_{11}O^+$: 135.0804; found 135.0803. (Cs_2CO_3 as the base)

Figure S18. HRMS analysis of the reaction mixture with Cs_2CO_3 as the base.

10. Compound Characterization Data

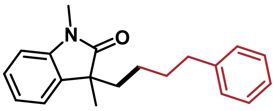

1,3-dimethyl-3-(4-phenylbutyl)indolin-2-one (3) Following the general procedure A, the title product was obtained after purification by column chromatography (PE/EA = 20:1) as a colorless oil (49.2 mg, 0.168 mmol, 84%). **^{1}H NMR** (500 MHz, $CDCl_3$) δ 7.29 – 7.26 (m, 1H), 7.25 – 7.20 (m, 2H), 7.19 – 7.11 (m, 2H), 7.09 – 7.04 (m, 3H), 6.84 (d, *J* = 7.7 Hz, 1H), 3.21 (s, 3H), 2.46 (t, *J* = 8.0 Hz, 2H), 1.93 (td, *J* = 12.7, 4.7 Hz, 1H), 1.77 (td, *J* = 12.8, 4.4 Hz, 1H), 1.56 – 1.40 (m, 2H), 1.35 (s, 3H), 1.14 – 1.02 (m, 1H), 0.99 – 0.86 (m, 1H). **^{13}C NMR** (126 MHz, $CDCl_3$) δ 181.0, 143.5, 142.7, 134.3, 128.41, 128.36, 127.8, 125.7, 122.6, 122.6, 108.0, 48.5, 38.4, 35.8, 31.8, 26.2, 24.4, 23.9. These data are in agreement with those reported previously in the literature.[9]

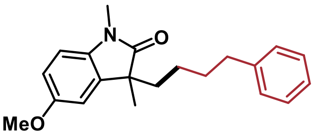

5-methoxy-1,3-dimethyl-3-(4-phenylbutyl)indolin-2-one (4) Following the general procedure A, the title product was obtained after purification by column chromatography (PE/EA = 20:1) as a yellow oil (60.7 mg, 0.188 mmol, 94%). **^{1}H NMR** (500 MHz, $CDCl_3$) δ 7.23 – 7.20 (m, 2H), 7.14 – 7.12 (m, 1H), 7.08 – 7.06 (m, 2H), 6.79 – 6.77 (m, 2H), 6.74 – 6.72 (m, 1H), 3.80 (s, 3H), 3.18 (s, 3H),

2.46 (t, *J* = 8.0 Hz, 2H), 1.93 (td, *J* = 12.6, 4.7 Hz, 1H), 1.74 (td, *J* = 12.6, 4.7 Hz, 1H), 1.55 – 1.41 (m, 2H), 1.34 (s, 3H), 1.13 – 1.00 (m, 1H), 0.99 – 0.86 (m, 1H). **^{13}C NMR** (126 MHz, $CDCl_3$) δ 180.5, 156.2, 142.6, 137.0, 135.8, 128.4, 128.3, 125.7, 111.6, 110.5, 108.2, 55.9, 49.0, 38.4, 35.7, 31.8, 26.3, 24.4, 23.9. **IR** (ATR): 2930, 2857, 1701, 1598, 1493, 1286, 1030, 743, 698 cm^{-1}. **HRMS** (ESI): *m/z* $[M+H]^+$ calcd for $C_{21}H_{26}NO_2^+$: 324.1958; found 324.1954.

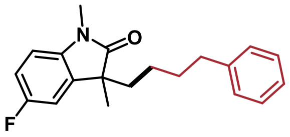

1,3-dimethyl-3-(4-phenylbutyl)-5-(trifluoromethyl)indolin-2-one (5) Following the general procedure A, the title product was obtained after purification by column chromatography (PE/EA = 20:1) as yellow oil (35.6 mg, 0.124 mmol, 62%). **^{1}H NMR** (600 MHz, $CDCl_3$) δ 7.25 – 7.21 (m, 2H), 7.16 – 7.12 (m, 1H), 7.09 – 7.05 (m, 2H), 6.99 – 6.93 (m, 1H), 6.92 – 6.88 (m, 1H), 6.76 – 6.73 (m, 1H), 3.19 (s, 3H), 2.47 (t, *J* = 8.0 Hz, 2H), 1.97 – 1.90 (m, 1H), 1.77 – 1.70 (m, 1H), 1.56 – 1.42 (m, 2H), 1.34 (s, 3H), 1.10 – 1.00 (m, 1H), 0.95 – 0.86 (m, 1H). **^{13}C NMR** (151 MHz, $CDCl_3$) δ 180.5, 159.5 (d, *J* = 240.3 Hz), 142.5, 139.3, 136.0 (d, *J* = 7.7 Hz), 128.40, 128.37, 125.8, 113.9 (d, *J* = 23.4 Hz), 110.8 (d, *J* = 24.5 Hz), 108.4 (d, *J* = 8.2 Hz), 49.0 (d, *J* = 1.8 Hz), 38.3, 35.7, 31.7, 26.4, 24.3, 23.8. **^{19}F NMR** (376 MHz, $CDCl_3$) δ -120.74 (s, 1F). **IR** (ATR): 2928, 2857, 1707, 1492, 1441, 1274, 1112, 749, 697, 560 cm^{-1}. **HRMS** (ESI): *m/z* $[M+H]^+$ calcd for $C_{20}H_{23}FNO^+$: 312.1758; found 312.1754.

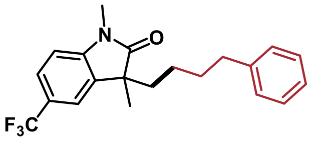

1,3-dimethyl-3-(4-phenylbutyl)-5-(trifluoromethyl)indolin-2-one (6) Following the general procedure A, the title product was obtained after purification by column chromatography (PE/EA = 20:1) as yellow oil (43.3 mg, 0.120 mmol, 60%). **^{1}H NMR** (500 MHz, $CDCl_3$) δ 7.58 – 7.50 (m, 1H), 7.37 (d, *J* = 1.8 Hz, 1H), 7.24 – 7.19 (m, 2H), 7.15 – 7.10 (m, 1H), 7.08 – 7.01 (m, 2H), 6.88 (d, *J* = 8.1 Hz, 1H), 3.22 (s, 3H), 2.46 (t, *J* = 7.9 Hz, 2H), 2.00 – 1.90 (m, 1H), 1.84 – 1.73 (m, 1H), 1.56 – 1.42 (m, 2H), 1.37 (s, 3H), 1.09 – 0.98 (m, 1H), 0.95 – 0.82 (m, 1H). **^{13}C NMR** (126 MHz, $CDCl_3$) δ 180.8, 146.4, 142.4, 134.9, 128.40, 128.37, 125.8, 125.7 (q, *J* = 4.4 Hz), 124.9 (q, *J* = 32.4 Hz), 122.5 (q, *J* = 272.2 Hz), 119.6 (q, *J* = 3.4 Hz), 107.8, 48.6, 38.2, 35.6, 31.6, 26.4, 24.2, 23.7. **^{19}F NMR** (376 MHz, $CDCl_3$) δ -61.12 (s, 3F). **IR** (ATR): 2967, 2931, 1711, 1621, 1326, 1080, 821, 749, 536 cm^{-1}. **HRMS** (ESI): *m/z* $[M+H]^+$ calcd for $C_{21}H_{23}F_3NO^+$: 362.1726; found 362.1721.

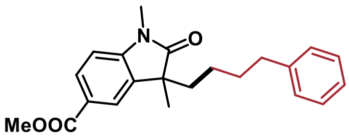

methyl 1,3-dimethyl-2-oxo-3-(4-phenylbutyl)indoline-5-carboxylate (7) Following the general procedure A, the title product was obtained after purification by column chromatography (PE/EA = 20:1) as yellow oil (44.3 mg, 0.126 mmol, 63%). **^{1}H NMR** (500 MHz, $CDCl_3$) δ 8.03 (dd, *J* = 8.2, 1.7 Hz, 1H), 7.84 (d, *J* = 1.7 Hz, 1H), 7.24 – 7.19 (m, 2H), 7.16 – 7.10 (m, 1H), 7.08 – 7.04 (m, 2H), 6.87 (d, *J* = 8.2 Hz, 1H), 3.93 (s, 3H), 3.24 (s, 3H), 2.45 (t, *J* = 8.0 Hz, 2H), 2.03 – 1.91 (m, 1H), 1.88 – 1.75 (m, 1H), 1.54 – 1.43 (m, 2H), 1.38 (s, 3H), 1.10 – 0.98 (m, 1H), 0.95 – 0.81 (m, 1H). **^{13}C NMR** (126 MHz, $CDCl_3$) δ 181.2, 167.2, 147.6, 142.5, 134.2, 130.7, 128.39, 128.36, 125.8, 124.5, 123.9, 107.6, 52.2, 48.4, 38.3, 35.7, 31.7, 26.4, 24.3, 23.8. **IR** (ATR): 2930, 2857, 1708, 1613, 1496 1237, 1100, 767, 749, 699 cm^{-1}. **HRMS** (ESI)**:** *m/z* $[M+H]^+$ calcd for $C_{22}H_{25}NO_3^+$: 352.1907; found 352.1903.

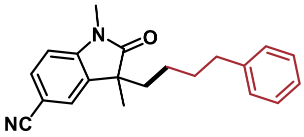

1,3-dimethyl-2-oxo-3-(4-phenylbutyl)indoline-5-carbonitrile (8) Following the general procedure A, the title product was obtained after purification by column chromatography (PE/EA = 20:1) as yellow oil (48.3 mg, 0.151 mmol, 76%). **^{1}H NMR** (500 MHz, $CDCl_3$) δ 7.61 – 7.59 (m 1H), 7.38 (s 1H), 7.25 – 7.22 (m 2H), 7.18 – 7.12 (m, 1H), 7.09 – 7.03 (m, 2H), 6.90 – 6.88 (m, 1H), 3.22 (s, 3H), 2.48 (t, *J* = 7.9 Hz, 2H), 1.98 – 1.92 (m, 1H), 1.80 – 1.74 (m, 1H), 1.57 – 1.43 (m, 2H), 1.36 (s, 3H), 1.08 – 0.97 (m, 1H), 0.92 – 0.83 (m, 1H). **^{13}C NMR** (126 MHz, $CDCl_3$) δ 180.5, 147.3, 142.2, 135.3, 133.3, 128.39, 128.38, 126.0, 125.9, 119.4, 108.4, 105.7, 48.4, 38.1, 35.5, 31.4, 26.4, 24.1, 23.6. **IR** (ATR): 2928, 2856, 2221, 1717, 1612, 1494, 1453, 1339, 750, 700 cm^{-1}. **HRMS** (ESI)**:** *m/z* $[M+H]^+$ calcd for $C_{21}H_{23}N_2O^+$: 319.1805; found 319.1804.

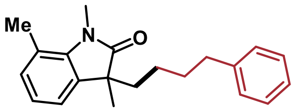

1,3,7-trimethyl-3-(4-phenylbutyl)indolin-2-one (9) Following the general procedure A, the title product was obtained after purification by column chromatography (PE/EA = 20:1) as a yellow oil (35.6 mg, 0.116 mmol, 58%).**^{1}H NMR** (500 MHz, $CDCl_3$) δ 7.24 – 7.21 (m, 2H), 7.16 – 7.11 (m, 1H), 7.09 – 7.05 (m, 2H), 7.01 – 6.92 (m, 3H), 3.48 (s, 3H), 2.58 (s, 3H), 2.46 (t, *J* = 8.0 Hz, 2H), 1.96 – 1.90 (m, 1H),

1.76 – 1.70 (m, 1H), 1.55 – 1.39 (m, 2H), 1.3 (s, 3H), 1.09 – 1.00 (m, 1H), 0.96 – 0.84 (m, 1H). **^{13}C NMR** (126 MHz, $CDCl_3$) δ 181.6, 142.7, 141.2, 134.9, 131.4, 128.4, 128.3, 125.7, 122.4, 120.5, 119.6, 47.8, 38.6, 35.7, 31.8, 29.5, 24.4, 24.3, 19.2. **IR** (ATR): 2967, 2856, 1703, 1601, 1454, 1363, 1337, 1073, 746, 699 cm^{-1}. **HRMS** (ESI): *m/z* [M+H]$^+$ calcd for $C_{21}H_{26}NO^+$: 308.2009; found 308.2005.

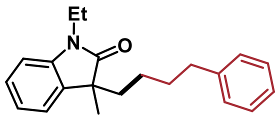

1-ethyl-3-methyl-3-(4-phenylbutyl)indolin-2-one (10) Following the general procedure A, the title product was obtained after purification by column chromatography (PE/EA = 20:1) as yellow oil (36.2 mg, 0.118 mmol, 59%). **^{1}H NMR** (500 MHz, $CDCl_3$) δ 7.26 – 7.18 (m, 3H), 7.18 – 7.09 (m, 2H), 7.09 – 7.02 (m, 3H), 6.85 (d, *J* = 7.8 Hz, 1H), 3.87 – 3.76 (m, 1H), 3.74 – 3.64 (m, 1H), 2.51 – 2.39 (m, 2H), 1.98 – 1.89 (m, 1H), 1.81 – 1.72 (m, 1H), 1.55 – 1.40 (m, 2H), 1.34 (s, 3H), 1.23 (t, *J* = 7.2 Hz, 3H), 1.12 – 0.99 (m, 1H), 0.95 – 0.81 (m, 1H). **^{13}C NMR** (126 MHz, $CDCl_3$) δ 180.4, 142.6, 142.5, 134.5, 128.4, 128.3, 127.7, 125.7, 122.8, 122.3, 108.2, 48.4, 38.5, 35.7, 34.6, 31.7, 24.3, 23.9, 12.8. **IR** (ATR): 2972, 2932, 2857, 1708, 1612, 1489, 1356, 749, 699 cm^{-1}. **HRMS** (ESI): *m/z* [M+H]$^+$ calcd for $C_{21}H_{26}NO^+$: 308.2009; found 308.2007.

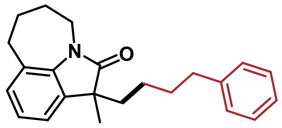

7-methyl-7-(4-phenylbutyl)-1,2,3,4-tetrahydroazepino[3,2,1-hi]indol-6(7H)-one (11) Following the general procedure A, the title product was obtained after purification by column chromatography (PE/EA = 20:1) as a yellow oil (51.3 mg, 0.154 mmol, 77%). **^{1}H NMR** (500 MHz, $CDCl_3$) δ 7.23 – 7.20 (m, 2H), 7.16 – 7.10 (m, 1H), 7.10 – 7.04 (m, 2H), 7.01 – 6.90 (m, 3H), 3.98 – 3.87 (m, 2H), 3.07 – 2.87 (m, 2H), 2.51 – 2.42 (m, 2H), 2.12 – 1.86 (m, 5H), 1.79 – 1.69 (m, 1H), 1.55 – 1.39 (m, 2H), 1.33 (s, 3H), 1.13 – 1.00 (m, 1H), 0.99 – 0.86 (m, 1H). **^{13}C NMR** (126 MHz, $CDCl_3$) δ 181.3, 142.7, 142.1, 134.5, 129.1, 128.4, 128.3, 125.7, 125.2, 122.4, 120.5, 48.5, 40.7, 38.7, 35.8, 31.8, 30.8, 26.62, 26.56, 24.4, 24.1. **IR** (ATR): 2926, 2855, 1704, 1651, 1600, 1453, 1357, 1157, 749, 698 cm^{-1}. **HRMS** (ESI): *m/z* [M+H]$^+$ calcd for $C_{23}H_{28}NO^+$: 334.2165; found 334.2165

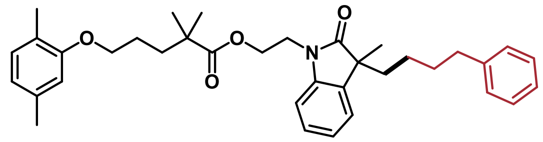

2-(3-methyl-2-oxo-3-(4-phenylbutyl)indolin-1-yl)ethyl 5-(2,5-dimethylphenoxy)-2,2-dimethylpentanoate (12) Following the general procedure A, the title product was obtained after purification by column chromatography (PE/EA = 5:1) as a brown oil (61.1 mg, 0.110 mmol, 55%). **^{1}H NMR** (500 MHz, $CDCl_3$) δ 7.25 – 7.18 (m, 3H), 7.16 – 7.10 (m, 2H), 7.08 – 7.02 (m, 3H), 6.99 (d, *J* = 7.5 Hz, 1H), 6.92 (d, *J* = 7.8 Hz, 1H), 6.65 (d, *J* = 6.0 Hz, 1H), 6.57 (s, 1H), 4.29 (t, *J* = 5.8 Hz, 2H), 4.01 – 3.90 (m, 2H), 3.85 – 3.77 (m, 2H), 2.45 (t, *J* = 8.0 Hz, 2H), 2.30 (s, 3H), 2.15 (s, 3H), 1.98 – 1.88 (m, 1H), 1.80 – 1.71 (m, 1H), 1.65 – 1.59 (m, 4H), 1.52 – 1.41 (m, 2H), 1.34 (s, 3H), 1.12 (s, 6H), 1.10 – 1.03 (m, 1H), 0.95 – 0.86 (m, 1H). **^{13}C NMR** (151 MHz, $CDCl_3$) δ 180.8, 177.8, 157.0, 142.6, 142.5, 136.5, 134.2, 130.4, 128.4, 128.3, 127.7, 125.7, 123.6, 122.8, 122.6, 120.8, 112.0, 108.4, 67.8, 61.6, 48.3, 42.1, 38.8, 38.4, 37.0, 35.7, 31.7, 25.18, 25.17, 25.1, 24.3, 24.0, 21.5, 15.9. **IR** (ATR): 2926, 2858, 1713, 1612, 1585, 1353, 1263, 1130, 749, 699 cm^{-1} **HRMS** (ESI): *m/z* [M+H]$^+$ calcd for $C_{36}H_{46}NO_4^+$: 556.3421; found 556.3419.

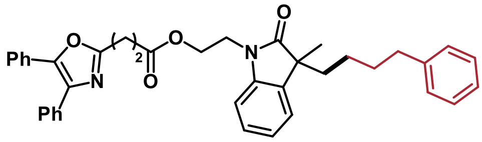

2-(3-methyl-2-oxo-3-(4-phenylbutyl)indolin-1-yl)ethyl 3-(4,5-diphenyloxazol-2-yl)propanoate (13) Following the general procedure A, the title product was obtained after purification by column chromatography (PE/EA = 5:1) as a yellow oil (83.8 mg, 0.140 mmol, 70%). **^{1}H NMR** (400 MHz, $CDCl_3$) δ 7.63 – 7.58 (m, 2H), 7.57 – 7.53 (m, 2H), 7.37 – 7.30 (m, 6H), 7.25 – 7.15 (m, 4H), 7.13 – 7.09 (m, 1H), 7.08 – 7.02 (m, 3H), 6.91 (d, *J* = 7.8 Hz, 1H), 4.37 (t, *J* = 5.7 Hz, 2H), 4.07 – 3.90 (m, 2H), 3.13 – 3.06 (m, 2H), 2.82 (t, *J* = 7.9 Hz, 2H), 2.45 (t, *J* = 7.9 Hz, 2H), 1.99 – 1.89 (m, 1H), 1.81 – 1.71 (m, 1H), 1.56 – 1.40 (m, 2H), 1.35 (s, 3H), 1.13 – 1.02 (m, 1H), 0.89 – 0.86 (m, 1H). **^{13}C NMR** (101 MHz, $CDCl_3$) δ 181.0, 171.9, 161.7, 145.6, 142.5, 135.3, 134.2, 132.6, 129.1, 128.8, 128.7, 128.6, 128.4, 128.3, 128.2, 128.0, 127.8, 126.6, 125.7, 122.9, 122.7, 108.2, 61.7, 48.4, 38.8, 38.4, 35.7, 31.7, 31.0, 24.3, 24.0, 23.4. **IR** (ATR): 3058, 2927, 2856, 1738, 1709, 1353, 1158, 764, 751, 695 cm^{-1} **HRMS** (ESI): *m/z* [M+H]$^+$ calcd for $C_{39}H_{39}N_2O_4^+$: 599.2904; found 599.2900.

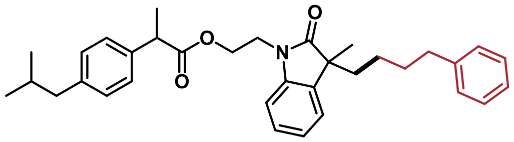

2-(3-methyl-2-oxo-3-(4-phenylbutyl)indolin - 1-yl)ethyl 4-(4-isopropylphenyl)-2-methylbutanoate (14) Following the general procedure A, the title product was obtained after purification by column chromatography (PE/EA = 5:1) as a yellow oil (57.2 mg, 0.112 mmol, 56%, d:r = 3:1). **^{1}H NMR** (500 MHz, $CDCl_3$) **(mixture of diastereomers)** δ 7.24 – 7.19 (m, 3H), 7.16 – 7.02 (m, 9H), 6.86 – 6.81 (m, 1H), 4.38 – 4.16 (m, 2H), 4.05 – 3.80 (m, 2H), 3.63 – 3.54 (m, 1H), 2.48 – 2.40 (m, 4H), 1.97 – 1.88 (m, 1H), 1.87 – 1.71 (m, 2H), 1.54 – 1.42 (m, 2H), 1.42 – 1.37 (m, 3H), 1.32 (d, *J* = 8.5 Hz, 3H), 1.11 – 0.97 (m, 1H), 0.94 – 0.81 (m, 8H). **^{13}C NMR** (151 MHz, $CDCl_3$) **(mixture of diastereomers)** δ 180.9, 174.7, 142.7, 142.6, 142.5, 140.7, 137.45, 137.43, 134.14, 134.13, 129.5, 128.4, 128.3, 127.7, 127.3, 125.7, 122.7, 122.6, 108.44, 108.42, 61.8, 48.32, 48.31, 38.8, 38.80, 38.38, 38.36, 35.7, 31.7, 30.3, 23.94, 23.91, 22.5, 18.5, 18.4. **IR** (ATR): 2952, 2928, 2866, 1734, 1712, 1612, 1352, 1156, 750, 699 cm^{-1} **HRMS** (ESI): *m/z* $[M+H]^+$ calcd for $C_{35}H_{44}NO_3^+$: 512.3159; found 512.3154.

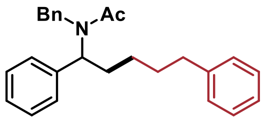

N-benzyl-N-(1,5-diphenylpentyl)acetamide (15) Following the general procedure A, the title product was obtained after purification by column chromatography (PE/EA = 20:1) as a colorless oil (31.0 mg, 0.836 mmol, 42%, d:r = 2:1(There is rotational isomerism)). **^{1}H NMR** (400 MHz, $CDCl_3$) **major isomer:** δ 7.33 – 7.14 (m, 13H), 6.98 – 6.92 (m, 2H), 5.96 (t, *J* = 7.8 Hz, 1H), 4.42 – 4.19 (m, 2H), 2.62 – 2.47 (m, 2H), 2.02 (s, 3H), 1.97 – 1.74 (m, 2H), 1.66 – 1.49 (m, 2H), 1.40 – 1.19 (m, 2H). **minor isomer:** δ 7.33 – 7.14 (m, 13H), 7.07 (d, *J* = 7.4 Hz, 2H), 4.97 – 4.86 (m, 2H), 3.84 (d, *J* = 15.2 Hz, 1H), 2.46 – 2.36 (m, 2H), 2.33 (s, 3H), 1.97 – 1.74 (m, 2H), 1.54 – 1.40 (m, 2H), 1.40 – 1.19 (m, 2H). **^{13}C NMR** (151 MHz, $CDCl_3$) **major isomer:** δ 172.0, 142.6, 139.9, 138.2, 128.7, 128.6, 128.4, 128.3, 127.7, 127.5, 126.2, 125.7, 56.6, 48.1, 35.8, 31.3, 30.7, 26.5, 22.8. **minor isomer:** δ 171.6, 142.3, 139.7, 139.4, 128.8, 128.5, 128.2, 128.1, 127.9, 127.1, 126.8, 125.9, 62.0, 45.8, 35.8, 31.9, 31.3, 26.6, 22.7. **IR** (ATR): 3027, 2932, 2858, 1642, 1453, 1409, 1276, 1261, 764, 699 cm^{-1}. **HRMS** (ESI): *m/z* $[M+Na]^+$ calcd for $C_{26}H_{29}ONNa^+$: 394.2123; found 394.2141.

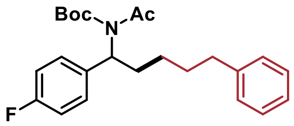

***tert*-butylacetyl(1-(4-fluorophenyl)-5-phenylpentyl)carbamate (16)** Following the general procedure A, the title product was obtained after purification by column chromatography (PE/EA = 20:1) as a yellow oil (35.9 mg, 0.900 mmol, 45%). **^{1}H NMR** (500 MHz, $CDCl_3$) δ 7.35 – 7.13 (m, 9H), 7.01 – 6.90 (m, 2H), 5.85 – 5.76 (m, 1H), 2.63 (t, *J* = 7.7 Hz, 2H), 2.44 (s, 3H), 2.26 – 2.06 (m, 2H), 1.77 – 1.62 (m, 2H), 1.42 – 1.33 (m, 2H), 1.28 (s, 9H). **^{13}C NMR** (126 MHz, $CDCl_3$) δ 173.6, 161.9 (d, *J* = 245.5 Hz), 153.5, 142.5, 136.8 (d, *J* = 3.3 Hz), 129.0 (d, *J* = 7.9 Hz), 128.5, 128.4, 125.9, 114.9 (d, *J* = 21.2 Hz), 83.4, 55.3, 35.9, 31.4, 31.2, 27.9, 26.7, 26.3. **^{19}F NMR** (376 MHz, $CDCl_3$) δ -115.84. **IR** (ATR): 2978, 2932, 2859, 1731, 1689, 1368, 1260, 1147, 750, 700 cm^{-1}. **HRMS** (APCI)**:** *m/z* $[M+Na]^+$ calcd for $C_{24}H_{30}FNO_3Na^+$: 422.2102; found 422.2100.

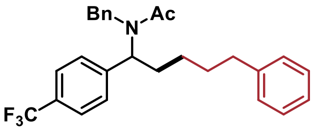

N-benzyl-N-(5-phenyl-1-(4-(trifluoromethyl)phenyl)pentyl) acetamide (17) Following the general procedure A, the title product was obtained after purification by column chromatography (PE/EA = 20:1) as a colorless oil (59.9 mg, 0.136 mmol, 68%, d:r = 4:1(There is rotational isomerism)). **^{1}H NMR** (600 MHz, $CDCl_3$) **major isomer:** δ 7.50 (d, *J* = 7.9 Hz, 2H), 7.41 (d, *J* = 8.0 Hz, 2H), 7.26 – 7.11 (m, 8H), 6.97 – 6.90 (m, 2H), 5.92 (t, *J* = 7.7 Hz, 1H), 4.38 (d, *J* = 17.5 Hz, 1H), 4.27 (d, *J* = 17.6 Hz, 1H), 2.59 – 2.48 (m, 2H), 2.06 (s, 3H), 1.95 – 1.83 (m, 2H), 1.65 – 1.51 (m, 2H), 1.37 – 1.29 (m, 2H). **minor isomer:** δ 7.56 (d, *J* = 7.9 Hz, 2H), 7.32 (d, *J* = 8.0 Hz, 2H), 7.26 – 7.11 (m, 8H), 7.09 – 7.07 (m, 2H), 4.94 (t, *J* = 7.6 Hz, 1H), 4.85 (d, *J* = 15.2 Hz, 1H), 3.92 (d, *J* = 15.2 Hz, 1H), 2.44 (d, *J* = 8.6 Hz, 2H), 2.32 (s, 3H), 1.99 – 1.80 (m, 2H), 1.51 – 1.44 (m, 1H), 1.29 – 1.24 (m, 2H), 0.92 – 0.81 (m, 1H). **^{13}C NMR** (126 MHz, $CDCl_3$) **(mixture of diastereomers)** δ 172.0, 171.5, 144.1, 143.7, 142.4, 142.2, 139.1, 137.6, 129.8 (q, *J* = 32.3 Hz), 128.9, 128.7, 128.5, 128.4, 128.3, 128.1, 127.9, 127.4, 127.0, 126.1, 125.9, 125.8, 125.7, 125.4 (q, *J* = 3.8 Hz), 124.2 (q, *J* = 272.1 Hz), 61.6, 56.3, 48.4, 45.9, 35.7, 31.8, 31.2, 30.5, 26.4, 26.3, 22.8, 22.7. **^{19}F NMR** (376 MHz, $CDCl_3$) δ -62.45 (s, 3F), -62.50 (s, 3F). **IR** (ATR): 3026, 2933, 2859, 1641, 1409, 1323, 1116, 1067, 750, 697 cm^{-1}. **HRMS** (ESI)**:** *m/z* $[M+Na]^+$ calcd for $C_{27}H_{28}F_3NONa^+$: 462.2015; found 462.1992.

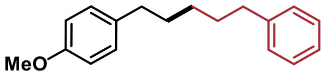

1-methoxy-4-(5-phenylpentyl)benzene (18) Following the general procedure A, the title product was obtained after purification by column chromatography (PE/EA = 50:1) as a colorless oil (16.3 mg, 0.064 mmol, 32%). **^{1}H NMR** (400 MHz, $CDCl_3$) δ 7.31 – 7.25 (m, 2H), 7.20 – 7.14 (m, 3H), 7.11 – 7.04 (m, 2H), 6.86 – 6.78 (m, 2H), 3.79 (s, 3H), 2.63 – 2.50 (m, 4H), 1.68 – 1.57 (m, 4H), 1.43 – 1.33 (m, 2H). **^{13}C NMR** (151 MHz, $CDCl_3$) δ 157.8, 143.0, 135.0, 129.4, 128.5, 128.4, 125.7, 113.8, 55.4, 36.0, 35.1, 31.8, 31.5, 29.0. These data are in agreement with those reported previously in the literature.[10]

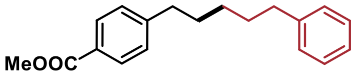

methyl 4-(5-phenylpentyl)benzoate (19) Following the general procedure A, the title product was obtained after purification by column chromatography (PE/EA = 50:1) as a colorless oil (24.8 mg, 0.880 mmol, 44%). **^{1}H NMR** (400 MHz, $CDCl_3$) δ 8.02 – 7.87 (m, 2H), 7.33 – 7.12 (m, 8H), 3.90 (s, 3H), 2.69 – 2.62 (m, 2H), 2.62 – 2.56 (m, 2H), 1.70 – 1.61 (m, 2H), 1.44 – 1.33 (m, 2H). **^{13}C NMR** (101 MHz, $CDCl_3$) δ 167.4, 148.5, 142.8, 129.8, 128.6, 128.5, 128.4, 127.8, 125.8, 52.1, 36.0, 36.0, 31.4, 31.1, 29.0. These data are in agreement with those reported previously in the literature.[10]

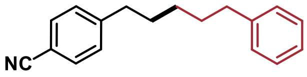

4-(5-phenylpentyl)benzonitrile (20) Following the general procedure A, the title product was obtained after purification by column chromatography (PE/EA = 100:1) as a colorless oil (20.4 mg, 0.082 mmol, 41%). **^{1}H NMR** (600 MHz, $CDCl_3$) δ 7.55 (d, J = 8.1 Hz, 2H), 7.29 – 7.26 (m, 2H), 7.25 (d, J = 8.1 Hz, 2H), 7.21 – 7.14 (m, 3H), 2.65 (t, J = 7.8 Hz, 2H), 2.60 (t, J = 7.7 Hz, 2H), 1.67 – 1.62 (m, 4H), 1.42 – 1.33 (m, 2H). **^{13}C NMR** (151 MHz, $CDCl_3$) δ 148.5, 142.6, 132.3, 129.3, 128.5, 128.4, 125.9, 119.3, 109.7, 36.1, 35.9, 31.3, 31.0, 28.9. **IR** (ATR): 3026, 2931, 2857, 2227, 1607, 1496, 1454, 842, 700, 560 cm^{-1}. **HRMS** (ESI): m/z $[M+H]^+$ calcd for $C_{18}H_{20}N^+$: 250.1590; found 250.1590.

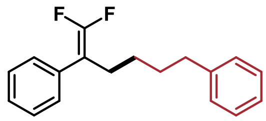

(6,6-difluorohex-5-ene-1,5-diyl)dibenzene (21) Following the general procedure B, the title product was obtained after purification by column chromatography (PE) as a colorless oil (41.3 mg, 0.152 mmol, 76%). **^{1}H NMR** (500 MHz, $CDCl_3$) δ 7.36 – 7.33 (m, 2H), 7.29 – 7.24 (m, 5H), 7.18 – 7.12 (m, 3H),

2.56 (t, J = 7.8 Hz, 2H), 2.44 – 2.40 (m, 2H), 1.65 – 1.59 (m, 2H), 1.46 – 1.36 (m, 2H). **^{13}C NMR** (126 MHz, $CDCl_3$) δ 153.7 (t, J = 288.4 Hz), 142.5, 133.9, 128.6, 128.5, 128.4, 127.3, 125.8, 92.4 (t, J = 17.5 Hz), 35.7, 30.8, 27.6, 27.4. **^{19}F NMR** (471 MHz, $CDCl_3$) δ -91.73. (s, 2F). These data are in agreement with those reported previously in the literature.[11]

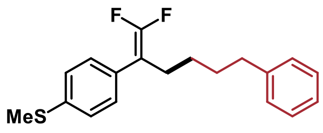

(4-(1,1-difluoro-6-phenylhex-1-en-2-yl)phenyl) (methyl) sulfane (22) Following the general procedure B, the title product was obtained after purification by column chromatography (PE/EA = 100:1) as a colorless oil (33.1 mg, 0.104 mmol, 52%). **^{1}H NMR** (400 MHz, $CDCl_3$) δ 7.28 – 7.24 (m, 2H), 7.24 – 7.15 (m, 5H), 7.15 – 7.10 (m, 2H), 2.60 – 2.53 (m, 2H), 2.49 (s, 3H), 2.40 (tt, J = 7.5, 2.4 Hz, 2H), 1.65 – 1.57 (m, 2H), 1.44 – 1.36 (m, 2H). **^{13}C NMR** (101 MHz, $CDCl_3$) δ153.7 (t, J = 284.1 Hz), 142.5, 137.5, 130.4, 128.7 (t, J = 3.3 Hz), 128.5, 128.4, 126.5, 125.8, 91.8 (dd, J = 16.4, 2.4 Hz), 35.6, 30.8, 27.3, 15.8. **^{19}F NMR** (376 MHz, $CDCl_3$) δ -91.38. (s, 2F). **IR** (ATR): 3025, 2924, 2858, 1726, 1495, 1232, 1094, 953, 819, 699 cm^{-1}. **HRMS** (ESI): *m/z* [M+K]$^+$ calcd for $C_{19}H_{20}F_2SK^+$: 357.0885; found 357.0895.

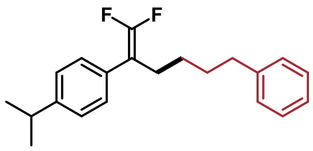

1-(1,1-difluoro-6-phenylhex-1-en-2-yl)-4-isopropylbenzene (23) Following the general procedure B, the title product was obtained after purification by column chromatography (PE) as a colorless oil (52.1 mg, 0.166 mmol, 83%). **^{1}H NMR** (400 MHz, $CDCl_3$) δ 7.31 – 7.20 (m, 6H), 7.20 – 7.15 (m, 1H), 7.16 – 7.09 (m, 2H), 2.90 (hept, J = 7.0 Hz, 1H), 2.63 – 2.53 (m, 2H), 2.43 – 2.38 (m, 2H), 1.67 – 1.58 (m, 2H), 1.46 – 1.38 (m, 2H), 1.26 (d, J = 7.0 Hz, 6H). **^{13}C NMR** (126 MHz, $CDCl_3$) δ 153.7 (dd, J = 288.12 Hz, 288.04 Hz), 148.0, 142.6, 131.1, 128.5, 128.4, 128.24 (t, J = 3.3 Hz), 126.6, 125.8, 92.2 (dd, J = 18.6 Hz, 13.3 Hz), 35.7, 33.9, 30.9, 27.5, 27.5 (t, J = 2.6 Hz), 24.1. **^{19}F NMR** (376 MHz, $CDCl_3$) δ -91.97 (2F). **IR** (ATR): 2960, 2928, 2860, 1838, 1729, 1455, 1231, 1056, 835, 699 cm^{-1}. **HRMS** (ESI): *m/z* [M+K]$^+$ calcd for $C_{21}H_{24}F_2K^+$: 353.1478; found 353.1484.

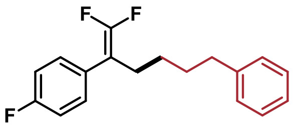

1-(1,1-difluoro-6-phenylhex-1-en-2-yl)-4-fluorobenzene (24) Following the general procedure B, the title product was obtained after

purification by column chromatography (PE) as a yellow oil (40.6 mg, 0.140 mmol, 70%). **^{1}H NMR** (600 MHz, $CDCl_3$) δ 7.27 – 7.22 (m, 4H), 7.19 – 7.15 (m, 1H), 7.12 (d, *J* = 7.5 Hz, 2H), 7.06 – 6.96 (m, 2H), 2.57 (t, *J* = 7.8 Hz, 2H), 2.39 (dd, 2H), 1.61 (p, *J* = 7.8 Hz, 2H), 1.39 (p, *J* = 7.6 Hz, 2H). **^{13}C NMR** (151 MHz, $CDCl_3$) δ 162.0 (d, *J* = 246.5 Hz), 153.7 (t, *J* = 287.9 Hz), 142.4, 130.1 (dt, *J* = 7.3, 3.3 Hz), 129.7, 128.5, 128.4, 125.9, 115.5 (d, *J* = 21.6 Hz), 91.6 (dd, *J* = 19.8, 15.6 Hz), 35.6, 30.8, 27.6, 27.2. **^{19}F NMR** (565 MHz, $CDCl_3$) δ -91.8 (s, 2F), -114.7 (s, 1F). **IR (ATR)**: 3026, 2932, 2859, 1751, 1601, 1508, 1230, 1160, 836, 699 cm^{-1}. **HRMS (ESI):** *m/z* $[M+K]^+$ calcd for $C_{18}H_{17}F_3K^+$: 329.0914.9603; found 329.0918.

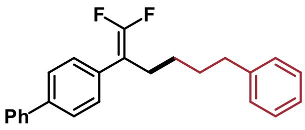

4-(1,1-difluoro-6-phenylhex-1-en-2-yl)-1,1'-biphenyl (25) Following the general procedure B, the title product was obtained after purification by column chromatography (PE) as a yellow oil (40.4 mg, 0.116 mmol, 58%). **^{1}H NMR** (600 MHz, $CDCl_3$) δ 7.61 – 7.57 (m, 4H), 7.46 – 7.44 (m, 2H), 7.39 – 7.33 (m, 3H), 7.27 – 7.24 (m, 2H), 7.19 – 7.11 (m, 3H), 2.62 – 2.56 (m, 2H), 2.48 – 2.45 (m, 2H), 1.68 – 1.60 (m, 2H), 1.48 – 1.43 (m, 2H). **^{13}C NMR** (151 MHz, $CDCl_3$) δ 153.8 (dd, *J* = 290.6 Hz, 287.5 Hz), 142.5, 140.7, 140.1, 132.8, 129.0, 128.7 (t, *J* = 3.4 Hz), 128.5, 128.4, 127.5, 127.3, 127.2, 125.9, 92.1 (dd, *J* = 20.6 Hz, 13.0 Hz), 35.7, 30.9, 29.9, 27.4. **^{19}F NMR** (565 MHz, $CDCl_3$) δ -90.95 – -91.21 (m, 2F). **IR (ATR)**: 3028, 2927, 2857, 1721, 1488, 1276, 1261, 1233, 841, 697 cm^{-1}. **HRMS (ESI):** *m/z* $[M+K]^+$ calcd for $C_{24}H_{22}F_2K^+$: 387.1321; found 387.1313.

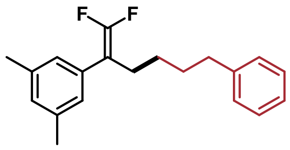

1-(1,1-difluoro-6-phenylhex-1-en-2-yl)-3,5-dimethylbenzene (26) Following the general procedure B, the title product was obtained after purification by column chromatography (PE) as a colorless oil (45.6 mg, 0.152 mmol, 76%). **^{1}H NMR** (500 MHz, $CDCl_3$) δ 7.27 – 7.23 (m, 2H), 7.19 – 7.10 (m, 3H), 6.91 (s, 1H), 6.88 (s, 2H), 2.57 (t, *J* = 7.8 Hz, 2H), 2.40 – 2.37 (m, 2H), 2.31 (s, 6H), 1.66 – 1.59 (m, 2H), 1.43 – 1.37 (m, 2H). **^{13}C NMR** (126 MHz, $CDCl_3$) δ 153.7 (dd, *J* = 289.8 Hz, 286.8 Hz), 142.6, 138.0, 133.7 (t, *J* = 26.0 Hz), 129.1, 128.5, 128.4, 126.2 (t, *J* = 3.0 Hz), 125.8, 92.4 (dd, *J* = 21.5, 13.1 Hz), 35.6, 30.8, 27.6, 27.4, 21.5. **^{19}F NMR** (471 MHz, $CDCl_3$) δ -91.79 – -92.32 (m, 2F). **IR** (ATR): 3026, 2924,

2859, 1838, 1737, 1603, 1454, 1275, 851, 699 cm^{-1}. **HRMS** (ESI): *m/z* $[M+K]^+$ calcd for $C_{20}H_{22}F_2K^+$: 339.1321; found 339.1323

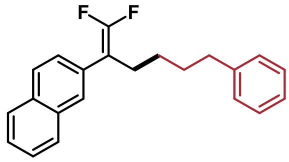

2-(1,1-difluoro-6-phenylhex-1-en-2-yl)naphthalene (27)

Following the general procedure B, the title product was obtained after purification by column chromatography (PE) as a yellow oil (38.7 mg, 0.120 mmol, 60%). **^{1}H NMR** (400 MHz, $CDCl_3$) δ 7.84 – 7.80 (m, 3H), 7.74 (s, 1H), 7.51 – 7.45 (m, 2H), 7.43 – 7.40 (m 1H), 7.25 – 7.22 (m, 2H), 7.17 – 7.11 (m, 3H), 2.58 – 2.50 (m, 4H), 1.68 – 1.61 (m, 2H), 1.48 – 1.40 (m, 2H). **^{13}C NMR** (151 MHz, $CDCl_3$) δ 154.0 (dd, *J* = 286.9 Hz, 290.6 Hz), 142.5, 133.4, 132.6, 131.3 (t, *J* = 4.1 Hz), 128.5, 128.4, 128.1, 128.0, 127.7, 127.5 (t, *J* = 3.3 Hz), 126.4, 126.3 (t, *J* = 3.2 Hz), 126.2, 125.8, 92.5 (dd, *J* = 21.9, 12.8 Hz), 35.6, 30.8, 27.6, 27.4 (t, *J* = 2.5 Hz). **^{19}F NMR** (376 MHz, $CDCl_3$) δ -91.08 – -91.47 (m, 2F). These data are in agreement with those reported previously in the literature.[12]

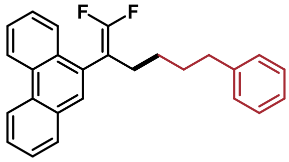

9-(1,1-difluoro-6-phenylhex-1-en-2-yl)phenanthrene (28)

Following the general procedure B, the title product was obtained after purification by column chromatography (PE) as a yellow oil (32.0 mg, 0.086 mmol, 43%). **^{1}H NMR** (400 MHz, $CDCl_3$) δ 8.74 (d, *J* = 8.2 Hz, 1H), 8.69 (d, *J* = 8.3 Hz, 1H), 7.94 (d, *J* = 8.1 Hz, 1H), 7.85 (d, *J* = 7.8 Hz, 1H), 7.71 – 7.65 (m, 2H), 7.64 – 7.59 (m, 2H), 7.24 – 7.20 (m, 2H), 7.16 – 7.11 (m, 1H), 7.08 (d, *J* = 7.4 Hz, 2H), 2.67 – 2.35 (m, 4H), 1.74 – 1.57 (m, 2H), 1.48 – 1.39 (m, 2H). **^{13}C NMR** (151 MHz, $CDCl_3$) δ 153.7 (t, *J* = 287.9 Hz), 142.5, 131.4, 130.8, 130.7, 130.3, 130.0, 128.7, 128.5 (d, *J* = 6.8 Hz), 128.4, 127.0, 126.8, 125.8 (d, *J* = 3.1 Hz), 123.2, 122.7, 90.5 (dd, *J* = 22.9, 16.9 Hz), 35.7, 31.1, 29.0, 27.4. **^{19}F NMR** (565 MHz, $CDCl_3$) δ-88.6 (d, *J* = 45.1 Hz, 2F), -93.1 (d, *J* = 42.7 Hz). **IR** (ATR): 3025, 2930, 2858, 1603, 1495, 1452, 1207, 1142, 896, 699 cm^{-1}. **HRMS** (ESI): *m/z* $[M+H]^+$ calcd for $C_{26}H_{22}F_2Na^+$: 395.1582; found 395.1585

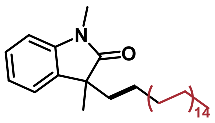

4,4'-(2-chlorocyclopropane-1,1-diyl)bis(methoxybenzene) (29)

Following the general procedure A, the title product was obtained after purification by column chromatography (PE/EA = 5:1) as a white solid (33.6

mg, 0.084 mmol, 42%). **^{1}H NMR** (400 MHz, $CDCl_3$) δ 7.29 – 7.24 (m, 1H), 7.18 – 7.16 (m, 1H), 7.09 – 7.05 (m, 1H), 6.84 (d, *J* = 7.7 Hz, 1H), 3.21 (s, 3H), 1.88 (td, *J* = 12.8, 4.6 Hz, 1H), 1.72 (td, *J* = 12.8, 4.4 Hz, 1H), 1.35 (s, 3H), 1.30 – 1.13 (m, 28H), 1.03 – 0.93 (m, 1H), 0.88 (t, *J* = 6.8 Hz, 3H), 0.84 – 0.77 (m, 1H). **^{13}C NMR** (101 MHz, $CDCl_3$) δ 181.0, 143.4, 134.4, 127.7, 122.6, 122.5, 108.0, 48.6, 38.7, 32.1, 29.9, 29.82, 29.79, 29.7, 29.50, 29.46, 26.2, 24.6, 23.9, 22.8, 14.3. **IR** (ATR): 3006, 2923, 2853, 1717, 1614, 1469, 1377, 1347, 897, 740 cm^{-1} **HRMS** (ESI): *m/z* [M+H]$^+$ calcd for $C_{27}H_{46}NO^+$: 400.3571; found 400.3574. **Melting Point** (Experimental): 57 – 58 °C

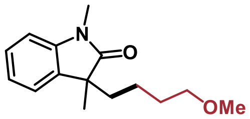

3-(4-methoxybutyl)-1,3-dimethylindolin-2-one (30) Following the general procedure A, the title product was obtained after purification by column chromatography (PE/EA = 5:1) as a yellow solid (27.7 mg, 0.112 mmol, 56%). **^{1}H NMR** (600 MHz, $CDCl_3$) δ 7.30 – 7.24 (m, 1H), 7.19 – 7.15 (m, 1H), 7.08 – 7.05 (m, 1H), 6.84 (d, *J* = 7.8 Hz, 1H), 3.27 – 3.20 (m, 8H), 1.96 – 1.87 (m, 1H), 1.78 – 1.73 (m, 1H), 1.51 – 1.39 (m, 2H), 1.35 (s, 3H), 1.07 – 0.98 (m, 1H), 0.97 – 0.86 (m, 1H).**^{13}C NMR** (151 MHz, $CDCl_3$) δ 180.9, 143.4, 134.2, 127.8, 122.7, 122.6, 108.0, 72.6, 58.6, 48.5, 38.5, 29.8, 26.3, 24.0, 21.3. **IR** (ATR): 2928, 2865, 1709, 1612, 1493, 1470, 1377, 1348, 1119, 755 cm^{-1}. **HRMS** (ESI): *m/z* [M+H]$^+$ calcd for $C_{15}H_{22}NO_2^+$: 248.1645; found 248.1642. **Melting Point** (Experimental): 60 – 61 °C

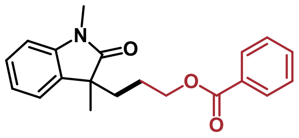

4,4'-(2-chlorocyclopropane-1,1-diyl)bis(methylbenzene) (31) Following the general procedure A, the title product was obtained after purification by column chromatography (PE/EA = 5:1) as a yellow wax (47.9 mg, 0.142 mmol, 71%). **^{1}H NMR** (500 MHz, $CDCl_3$) δ 8.02 – 7.97 (m, 2H), 7.57 – 7.52 (m, 1H), 7.44 – 7.41 (m, 2H), 7.30 – 7.26 (m, 1H), 7.20 – 7.18 (m, 1H), 7.09 – 7.06 (m, 1H), 6.86 (d, *J* = 7.8 Hz, 1H), 4.17 (t, *J* = 6.5 Hz, 2H), 3.23 (s, 3H), 2.10 – 2.04 (m, 1H), 1.92 – 1.86 (m, 1H), 1.56 – 1.45 (m, 1H), 1.39 (s, 3H), 1.38 – 1.28 (m, 1H). **^{13}C NMR** (126 MHz, $CDCl_3$) δ 180.4, 166.6, 143.4, 133.7, 133.0, 130.3, 129.6, 128.4, 128.0, 122.7, 122.6, 108.2, 64.7, 48.1, 34.9, 26.3, 24.1, 23.9. **IR** (ATR): 2959, 2924, 2868, 1708, 1611, 1460, 1269, 1108, 746, 709 cm^{-1} **HRMS** (ESI): *m/z* [M+H]$^+$ calcd for $C_{20}H_{22}NO_3^+$: 324.1594; found 324.1593.

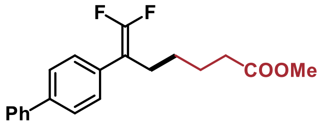

methyl 6-([1,1'-biphenyl]-4-yl)-7,7-difluorohept-6-enoate (32) Following the general procedure B, the title product was obtained after purification by column chromatography (PE/EA = 10:1) as a colorless oil (48.8 mg, 0.148 mmol, 74%). **^{1}H NMR** (600 MHz, $CDCl_3$) δ 7.62 – 7.57 (m, 4H), 7.47 – 7.43 (m, 2H), 7.40 – 7.33 (m, 3H), 3.64 (s, 3H), 2.46 (tt, *J* = 7.6, 2.4 Hz, 2H), 2.30 (t, *J* = 7.5 Hz, 2H), 1.70 – 1.61 (m, 2H), 1.46 – 1.41 (m, 2H). **^{13}C NMR** (151 MHz, $CDCl_3$) δ 174.1, 153.9 (dd, *J* = 288.0, 287.8 Hz), 140.7, 140.2, 132.6, 129.0, 128.7 (t, *J* = 3.4 Hz), 127.6, 127.3, 127.2, 91.8 (dd, *J* = 20.5, 14.4 Hz), 51.7, 33.9, 27.32 (d, *J* = 4.9 Hz), 27.30, 24.4. **^{19}F NMR** (565 MHz, $CDCl_3$) δ -90.76 – -90.95 (m, 2F).**IR** (ATR):2950, 2859, 1737, 1488, 1437, 1234, 1106, 1007, 842, 698 cm^{-1}. **HRMS** (ESI)**:** *m/z* $[M+K]^+$ calcd for $C_{20}H_{20}F_2O_2K^+$: 369.1063; found 369.1068.

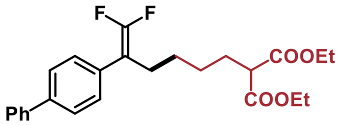

diethyl 2-(5-([1,1'-biphenyl]-4-yl)-6,6-difluorohex-5-en-1-yl)malonate (33) Following the general procedure B, the title product was obtained after purification by column chromatography (PE/EA = 5:1) as a yellow oil (53.4 mg, 0.124 mmol, 62%). **^{1}H NMR** (500 MHz, $CDCl_3$) δ 7.61 – 7.57 (m, 4H), 7.46 – 7.44 (m, 2H), 7.39 – 7.32 (m, 3H), 4.22 – 4.14 (m, 4H), 3.28 (t, *J* = 7.5 Hz, 1H), 2.45 – 2.41 (m, 2H), 1.90 – 1.85 (m, 2H), 1.47 – 1.41 (m, 2H), 1.39 – 1.32 (m, 2H), 1.25 (t, *J* = 7.1 Hz, 6H). **^{13}C NMR** (126 MHz, $CDCl_3$) δ 169.6, 153.8 (dd, *J* = 287.8, 287.9 Hz), 140.7, 140.2, 132.6, 128.9, 128.7 (t, *J* = 3.4 Hz), 127.5, 127.3, 127.2, 92.0 (dd, *J* = 20.8, 13.8 Hz), 61.5, 52.1, 28.5, 27.5, 27.4, 26.9, 14.2. **^{19}F NMR** (471 MHz, $CDCl_3$) δ-90.81 – -91.04 (m, 2F). **IR** (ATR):2930, 2859, 1728, 1231, 1148, 1027, 1008, 842, 732, 697 cm^{-1}. **HRMS** (ESI)**:** *m/z* $[M+K]^+$ calcd for $C_{25}H_{28}F_2O_4K^+$: 469.1587; found 469.1587.

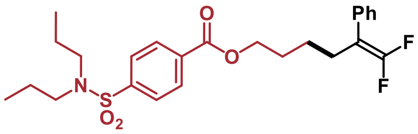

6,6-difluoro-5-phenylhex-5-en-1-yl 4-(N,N-dipropylsulfamoyl)benzoate (34) Following the general procedure B, the title product was obtained after purification by column chromatography (PE/EA = 10:1) as a yellow wax (32.4 mg, 0.072 mmol, 36%). **^{1}H NMR** (600 MHz, $CDCl_3$) δ 8.09 – 8.06 (m, 2H), 7.87 – 7.84 (m, 2H), 7.38 – 7.25 (m, 5H), 4.31 (t, *J* = 6.6 Hz, 2H), 3.14 – 3.05 (m, 4H), 2.49 (tt, *J* = 7.5, 2.4 Hz, 2H), 1.81 – 1.76 (m, 2H), 1.58 –

1.50 (m, 6H), 0.87 (t, *J* = 7.4 Hz, 6H). **^{13}C NMR** (151 MHz, $CDCl_3$) δ 165.4, 153.7 (dd, *J* = 289.5, 286.9 Hz), 144.3, 133.7, 133.5, 130.3, 128.6, 128.4 (t, *J* = 3.2 Hz), 127.5, 127.1, 92.0 (dd, *J* = 21.4, 13.5 Hz), 65.3, 50.1, 28.0, 27.3, 24.1, 22.1, 11.3. **^{19}F NMR** (565 MHz, $CDCl_3$) δ -91.17 – -91.42 (m, 2F). **IR** (ATR): 2965, 2933, 2876, 1723, 1342, 1274, 1159, 697, 603, 564 cm^{-1}. **HRMS** (ESI): *m/z* $[M+H]^+$ calcd for $C_{23}H_{27}F_2NO_4SNa^+$: 480.2015; found 480.2014.

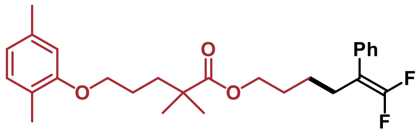

6,6-difluoro-5-phenylhex-5-en-1-yl 5-(2,5-dimethylphenoxy)-2,2-dimethylpentanoate (35) Following the general procedure B, the title product was obtained after purification by column chromatography (PE/EA = 20:1) as a yellow oil (38.2 mg, 0.086 mmol, 43%). **^{1}H NMR** (600 MHz, $CDCl_3$) δ 7.37 – 7.31 (m, 2H), 7.30 – 7.23 (m, 3H), 7.00 (d, *J* = 7.4 Hz, 1H), 6.65 (d, *J* = 7.4 Hz, 1H), 4.02 (t, *J* = 6.5 Hz, 2H), 3.88 (t, *J* = 5.6 Hz, 2H), 2.43 (tt, *J* = 7.6, 2.4 Hz, 2H), 2.30 (s, 3H), 2.16 (s, 3H), 1.72 – 1.59 (m, 6H), 1.42 (p, *J* = 7.7 Hz, 2H), 1.16 (s, 6H). **^{13}C NMR** (151 MHz, $CDCl_3$) δ 177.9, 157.1, 153.8 (dd, *J* = 289.7, 287.1 Hz), 136.6, 133.6, 130.4, 128.6, 128.3 (t, *J* = 3.2 Hz), 127.4, 123.7, 120.8, 112.0, 92.1 (dd, *J* = 21.2, 13.6 Hz), 68.0, 64.0, 42.2, 37.2, 28.1, 27.3, 25.29, 25.25, 24.2 (t, *J* = 2.7 Hz), 21.5, 15.9. **^{19}F NMR** (565 MHz, $CDCl_3$) -91.22 – -91.67 (m, 2F). **IR** (ATR): 2952, 2925, 2868, 1724, 1230, 1144, 1128, 1047, 803, 768, 697 cm^{-1}. **HRMS** (ESI): *m/z* $[M+H]^+$ calcd for $C_{27}H_{35}F_2O_3^+$: 445.2549; found 445.2546.

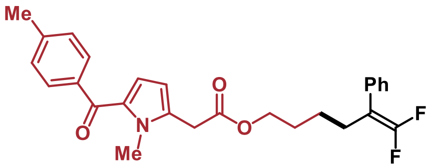

6,6-difluoro-5-phenylhex-5-en-1-yl 2-(1-methyl-5-(4-methylbenzoyl)-1H-pyrrol-2-yl)acetate (36) Following the general procedure B, the title product was obtained after purification by column chromatography (PE/EA = 20:1) as a brown oil (40.6 mg, 0.090 mmol, 45%). **^{1}H NMR** (400 MHz, $CDCl_3$) δ 7.76 – 7.69 (m, 2H), 7.42 – 7.32 (m, 2H), 7.29 – 7.22 (m, 4H), 6.66 (d, *J* = 4.1 Hz, 1H), 6.07 (d, *J* = 4.1 Hz, 1H), 4.10 (t, *J* = 6.6 Hz, 2H), 3.90 (s, 3H), 3.66 (s, 2H), 2.45 – 2.40 (m, 5H), 1.68 – 1.61 (m, 2H), 1.45 – 1.37 (m, 2H). **^{13}C NMR** (101 MHz, $CDCl_3$) δ 186.0, 169.5, 153.7 (t, *J* = 288.6 Hz), 142.0, 137.4, 134.6, 133.4, 131.5, 129.6, 128.8, 128.6, 128.3 (t, *J* = 3.3 Hz), 127.5, 122.4, 109.5, 92.0 (t, *J* = 18.7, 16.3 Hz), 65.1, 33.3, 33.0, 27.9, 27.2, 24.1 (t, *J* = 2.7 Hz), 21.7. **^{19}F NMR** (376 MHz, $CDCl_3$) δ -91.34 (s, 2F). **IR** (ATR): 2950,

2932, 2867, 1737, 1625, 1607, 1376, 1232, 884, 700 cm^{-1}. **HRMS** (ESI): *m/z* [M+H]$^+$ calcd for $C_{27}H_{28}F_2NO_3^+$: 452.2032; found 452.2030.

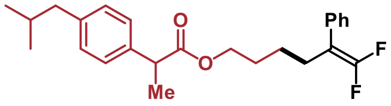

6,6-difluoro-5-phenylhex-5-en-1-yl 2-(4-isobutylphenyl)propanoate (37) Following the general procedure B, the title product was obtained after purification by column chromatography (PE/EA = 20:1) as a yellow wax (48.0 mg, 0.120 mmol, 60%). **^{1}H NMR** (400 MHz, $CDCl_3$) δ 7.40 – 7.32 (m, 2H), 7.29 – 7.23 (m, 3H), 7.17 – 7.04 (m, 4H), 4.01 (t, *J* = 6.5 Hz, 2H), 3.64 (q, *J* = 7.2 Hz, 1H), 2.43 (d, *J* = 7.2 Hz, 2H), 2.36 (tt, *J* = 7.6, 2.4 Hz, 2H), 1.83 (dp, *J* = 13.5, 6.7 Hz, 1H), 1.60 – 1.53 (m, 2H), 1.45 (d, *J* = 7.2 Hz, 3H), 1.38 – 1.28 (m, 2H), 0.89 (d, *J* = 6.6 Hz, 6H). **^{13}C NMR** (101 MHz, $CDCl_3$) δ 174.9, 153.7 (t, *J* = 288.5 Hz), 140.6, 137.9, 133.6, 129.4, 128.6, 128.3 (t, *J* = 3.2 Hz), 127.4, 127.2, 92.1 (t, *J* = 17.4 Hz), 64.3, 45.2, 45.1, 30.3, 27.9, 27.2, 24.0 (d, *J* = 2.8 Hz), 22.5, 18.5. **^{19}F NMR** (376 MHz, $CDCl_3$) δ-91.47 (s, 2F). **IR** (ATR): 2955, 2933, 2869, 1733, 1450, 1264, 1202, 1167, 849, 699 cm^{-1}. **HRMS** (ESI): *m/z* [M+Na]$^+$ calcd for $C_{25}H_{30}F_2O_2Na^+$: 423.2106; found 423.2104.

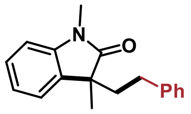

1,3-dimethyl-3-phenethylindolin-2-one (38) Following the general procedure A, the title product was obtained after purification by column chromatography (PE/EA = 20:1) as a white solid (49.9 mg, 0.188 mmol, 94%). **^{1}H NMR** (400 MHz, $CDCl_3$) δ 7.33 – 7.26 (m, 1H), 7.24 – 7.16 (m, 3H), 7.16 – 7.06 (m, 2H), 7.02 (d, *J* = 7.1 Hz, 2H), 6.86 (d, *J* = 7.7 Hz, 1H), 3.20 (s, 3H), 2.37 – 2.20 (m, 2H), 2.19 – 2.08 (m, 1H), 2.07 – 1.95 (m, 1H) 1.39 (s, 3H). **^{13}C NMR** (101 MHz, $CDCl_3$) δ 180.5, 143.6, 141.5, 133.9, 128.4, 128.4, 128.0, 126.0, 122.7, 122.6, 108.1, 48.5, 40.4, 31.1, 26.3, 24.1. These data are in agreement with those reported previously in the literature.[13]

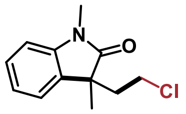

3-(2-chloroethyl)-1,3-dimethylindolin-2-one (39) Following the general procedure A, the title product was obtained after purification by column chromatography (PE/EA = 20:1) as a colorless oil (33.6 mg, 0.150 mmol, 75%). **^{1}H NMR** (400 MHz, $CDCl_3$) δ 7.34 – 7.27 (m, 1H), 7.19 (d, *J* = 7.0 Hz, 1H), 7.13 – 7.06 (m, 1H), 6.86

(d, *J* = 7.7 Hz, 1H), 3.26 – 3.18 (m, 4H), 3.18 – 3.11 (m, 1H), 2.50 – 2.39 (m, 1H), 2.27 – 2.16 (m, 1H) 1.40 (s, 3H). **^{13}C NMR** (101 MHz, $CDCl_3$) δ 179.6, 143.3, 132.6, 128.4, 122.9, 122.7, 108.4, 47.6, 40.9, 40.1, 26.4, 24.1. These data are in agreement with those reported previously in the literature.[14]

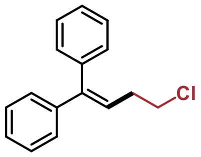

(4-chlorobut-1-ene-1,1-diyl)dibenzene (40) Following the general procedure A, the title product was obtained after purification by column chromatography (PE) as a colorless oil (28.6 mg, 0.118 mmol, 59%). **^{1}H NMR** (400 MHz, $CDCl_3$) δ 7.43 – 7.31 (m, 4H), 7.23 (d, *J* = 7.4 Hz, 4H), 7.20 – 7.14 (m, 2H), 6.11 (t, *J* = 7.3 Hz, 1H), 3.58 (t, *J* = 6.8 Hz, 2H), 2.59 (q, *J* = 7.0 Hz, 2H). **^{13}C NMR** (151 MHz, $CDCl_3$) δ 144.6, 142.3, 139.8, 129.9, 128.5, 128.3, 127.45, 127.43, 127.41, 124.9, 44.5, 33.0. These data are in agreement with those reported previously in the literature.[15]

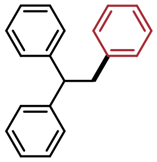

ethane-1,1,2-triyltribenzene (41) Following the general procedure A, the title product was obtained after purification by column chromatography (PE) as a colorless oil (16.5 mg, 0.064 mmol, 32%). **^{1}H NMR** (400 MHz, $CDCl_3$) δ 7.25 – 7.24 (m, 2H), 7.23 – 7.18 (m, 6H), 7.18 – 7.08 (m, 5H), 7.03 – 6.97 (m, 2H), 4.23 (t, *J* = 7.8 Hz, 1H), 3.36 (d, *J* = 7.8 Hz, 2H). **^{13}C NMR** (101 MHz, $CDCl_3$) δ 144.6, 140.4, 129.2, 128.5, 128.2, 126.3, 126.0, 53.2, 42.2. These data are in agreement with those reported previously in the literature.[16]

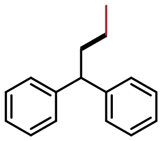

butane-1,1-diyldibenzene (43) Following the procedure C, the title product was obtained after purification by column chromatography (PE/EA = 20:1) as a colorless oil (24.8 mg, 0.118 mmol, 59%). **^{1}H NMR** (400 MHz, $CDCl_3$) δ 7.30 – 7.25 (m, 3H), 7.24 – 7.20 (m, 5H), 7.17 – 7.13 (m, 2H), 3.90 (t, *J* = 7.8 Hz, 1H), 2.07 – 1.96 (m, 2H), 1.31 – 1.24 (m, 2H), 0.92 (t, *J* = 7.4 Hz, 3H). **^{13}C NMR** (101 MHz, $CDCl_3$) δ 145.5, 128.5, 128.0, 126.1, 51.2, 38.1, 21.3, 14.2. These data are in agreement with those reported previously in the literature.[17]

11. NMR Spectra

^{1}H NMR of 3 (500 MHz, $CDCl_3$)

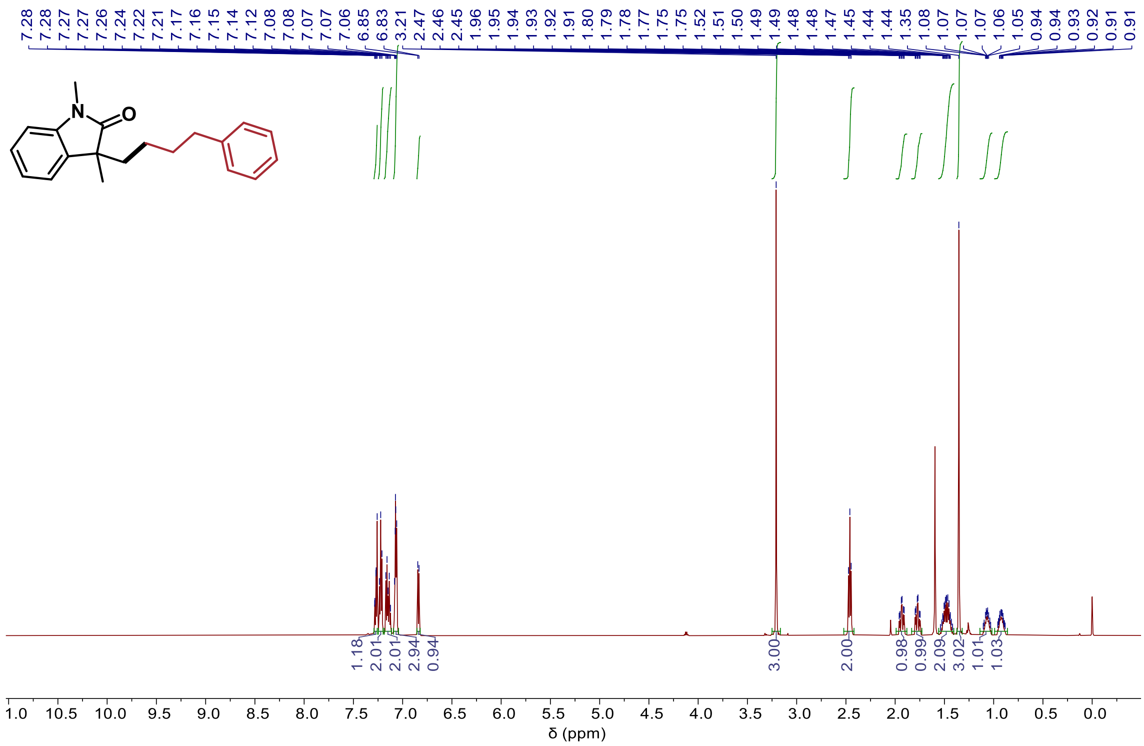

^{13}C NMR of 3 (126 MHz, $CDCl_3$)

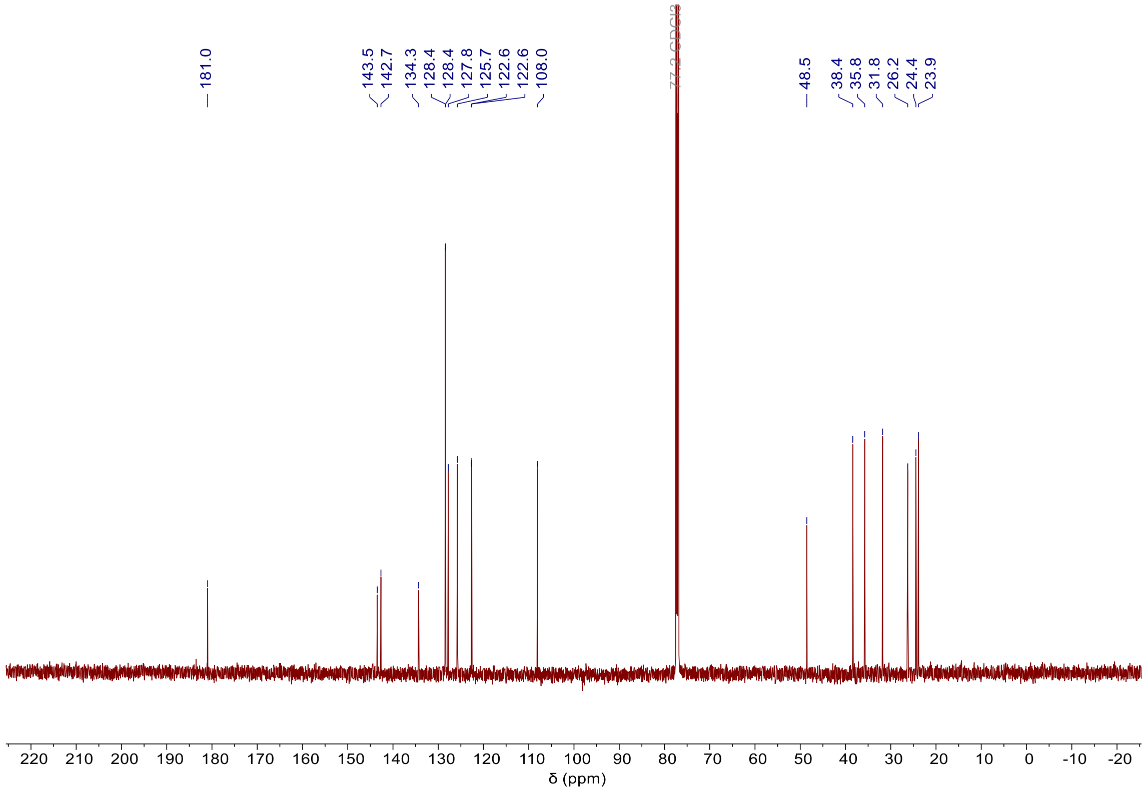

^{1}H NMR of 4 (500 MHz, $CDCl_3$)

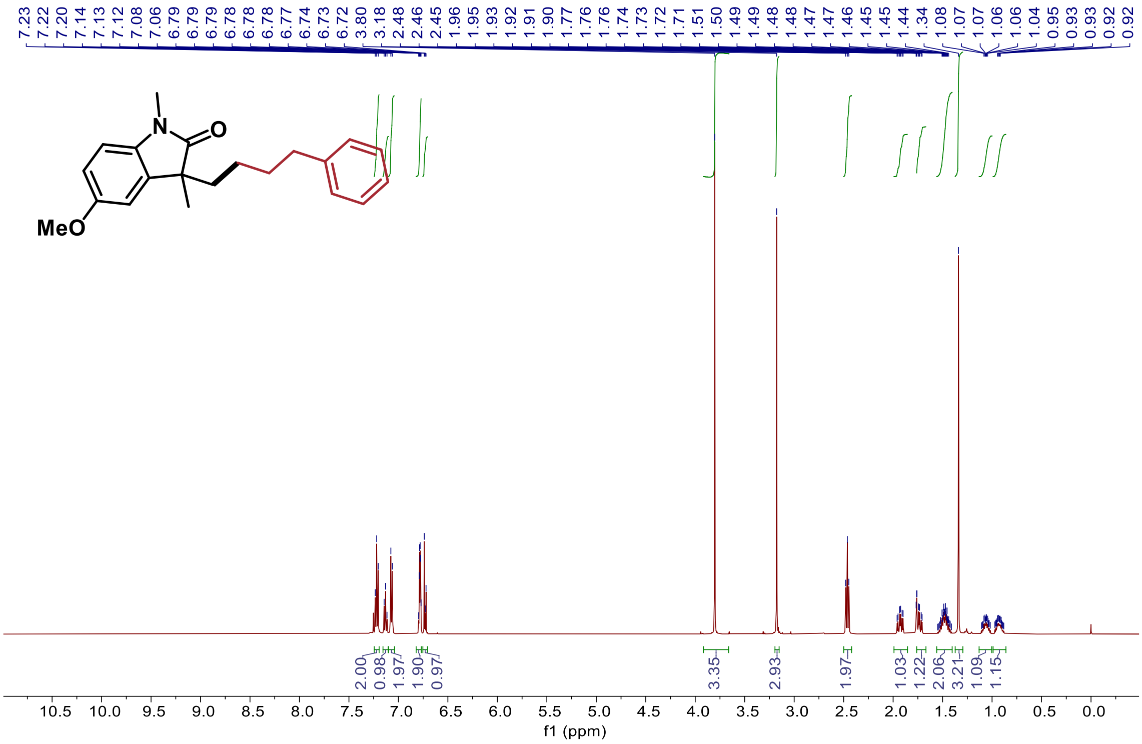

^{13}C NMR of 4 (126 MHz, $CDCl_3$)

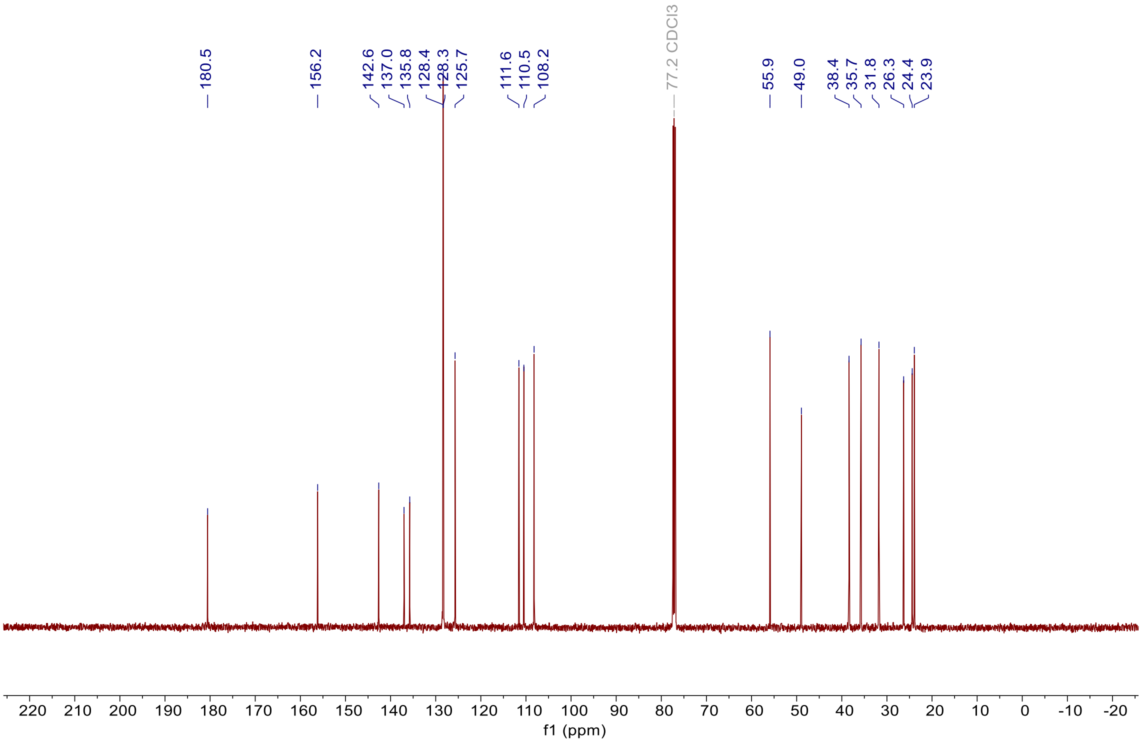

^{1}H NMR of 5 (600 MHz, $CDCl_3$)

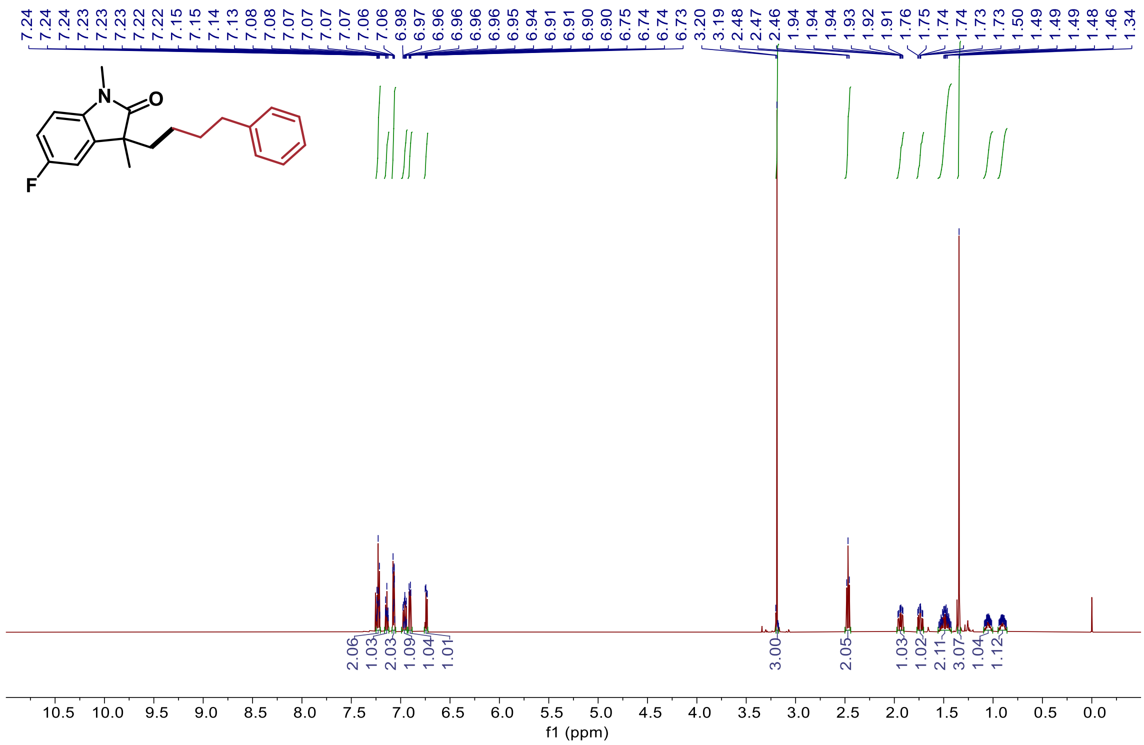

^{13}C NMR of 5 (151 MHz, $CDCl_3$)

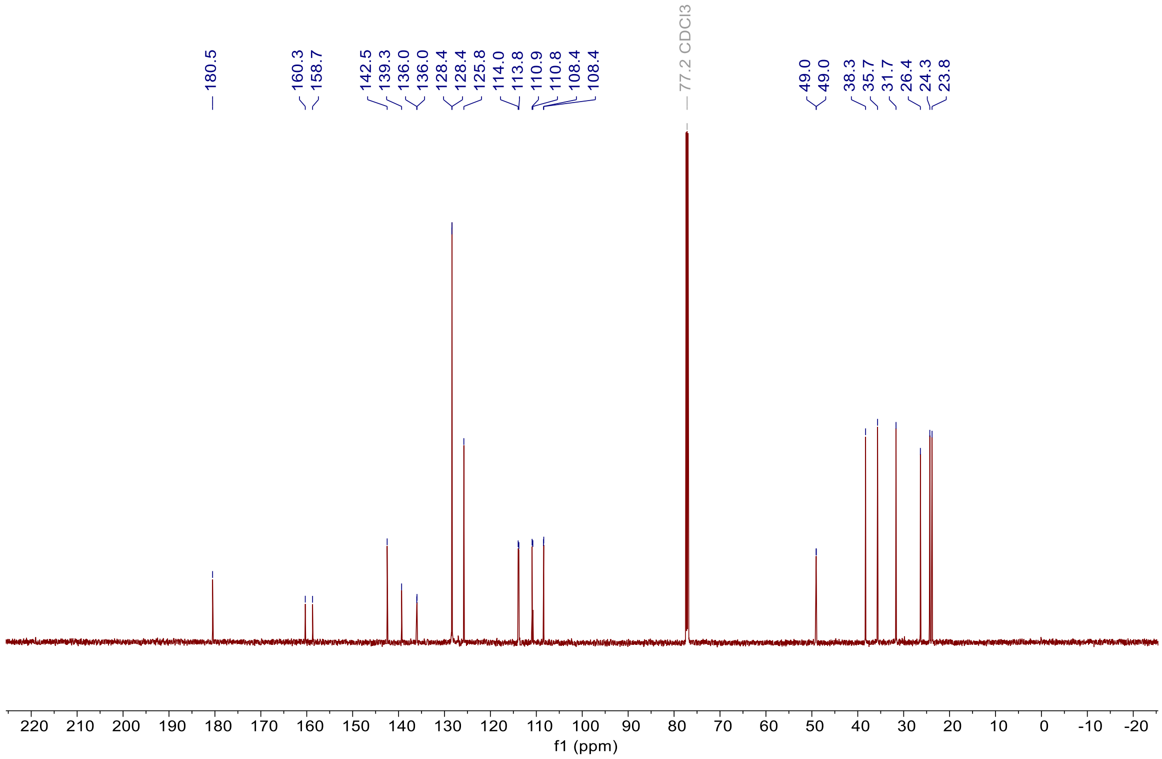

^{19}F NMR of 5 (376 MHz, CDCl$_3$)

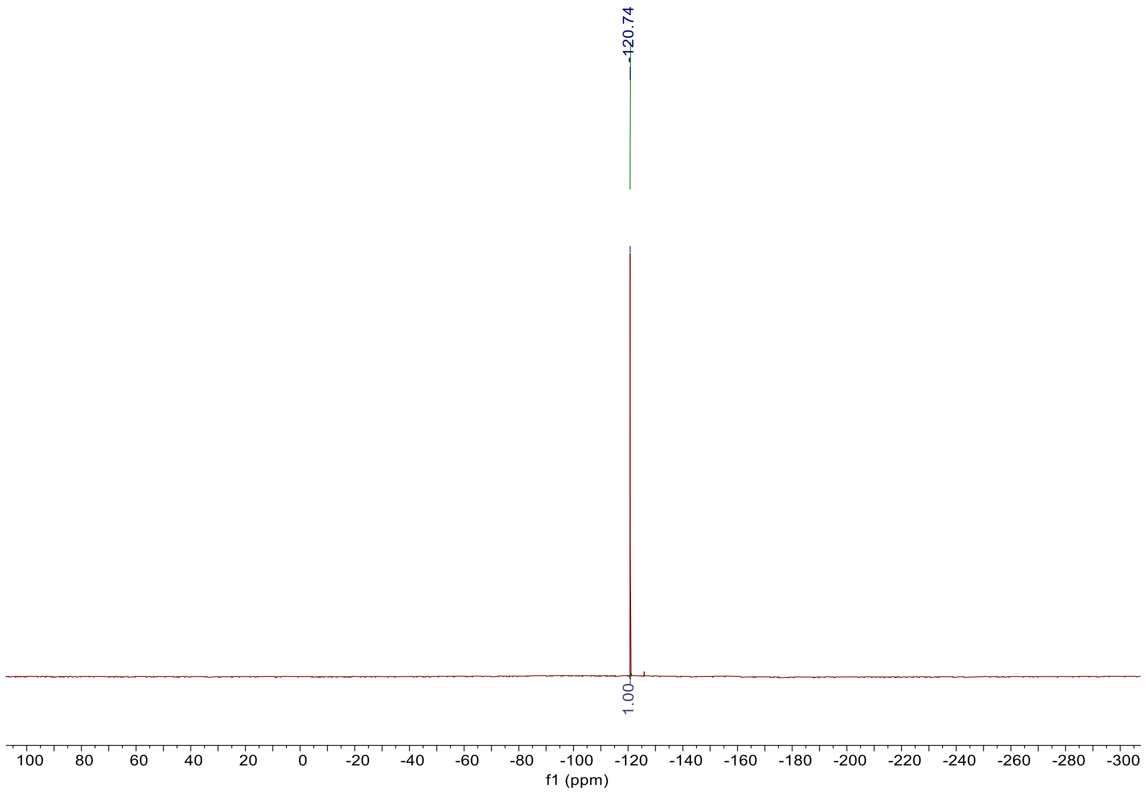

^{1}H NMR of 6 (500 MHz, $CDCl_3$)

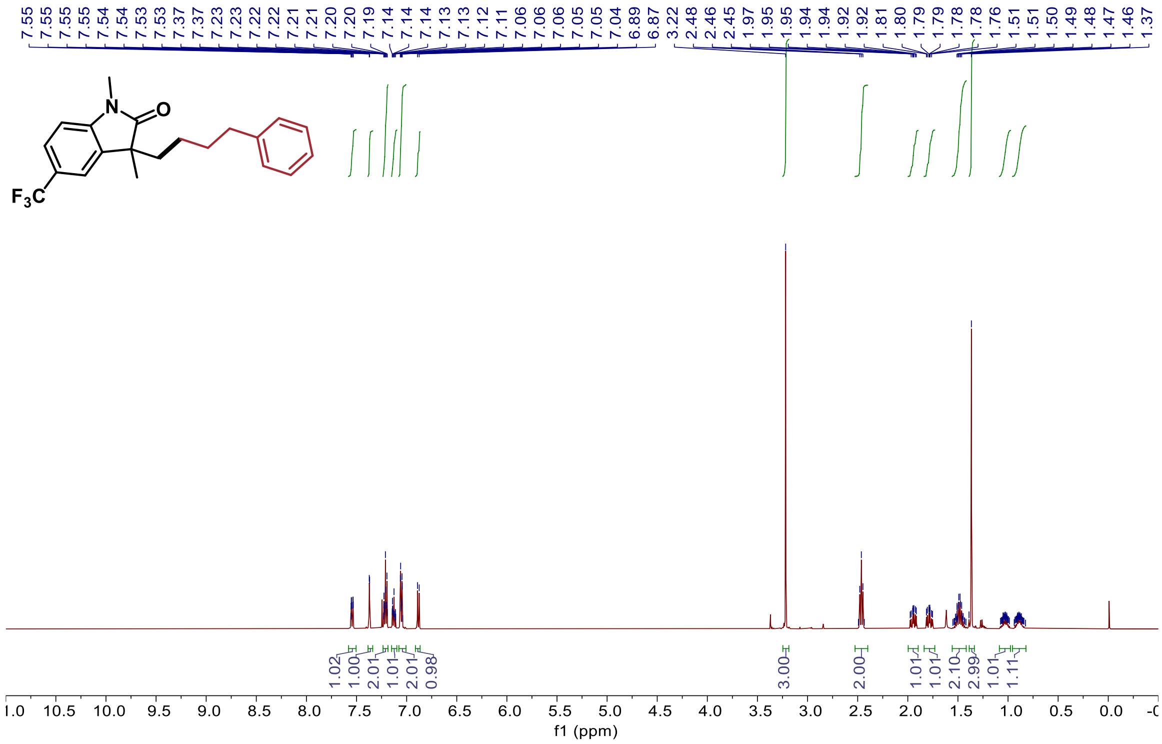

^{13}C NMR of 6 (126 MHz, $CDCl_3$)

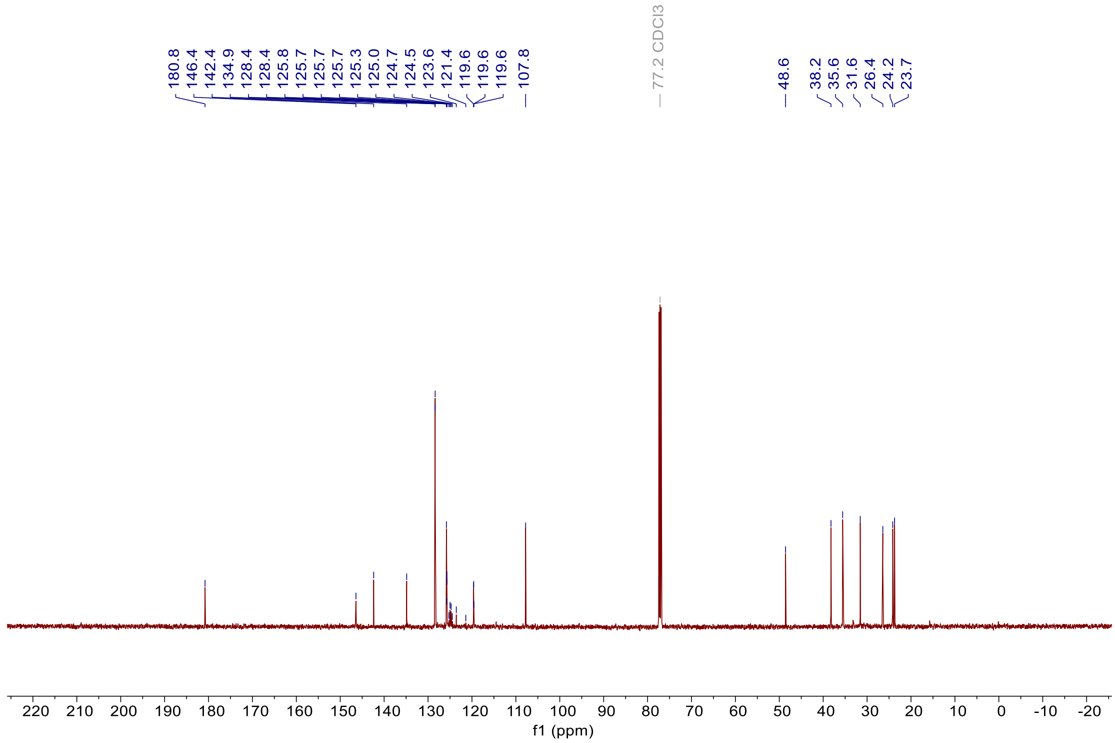

^{19}F NMR of 6 (376 MHz, $CDCl_3$)

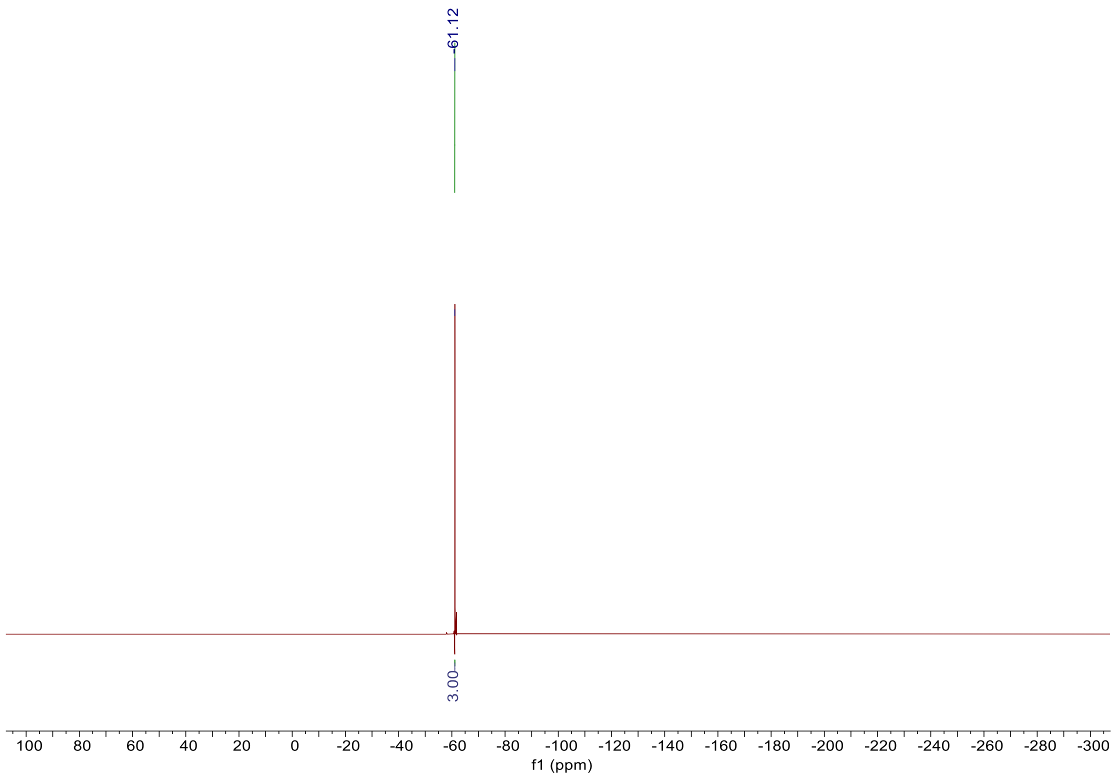

^{1}H NMR of 7 (500 MHz, $CDCl_3$)

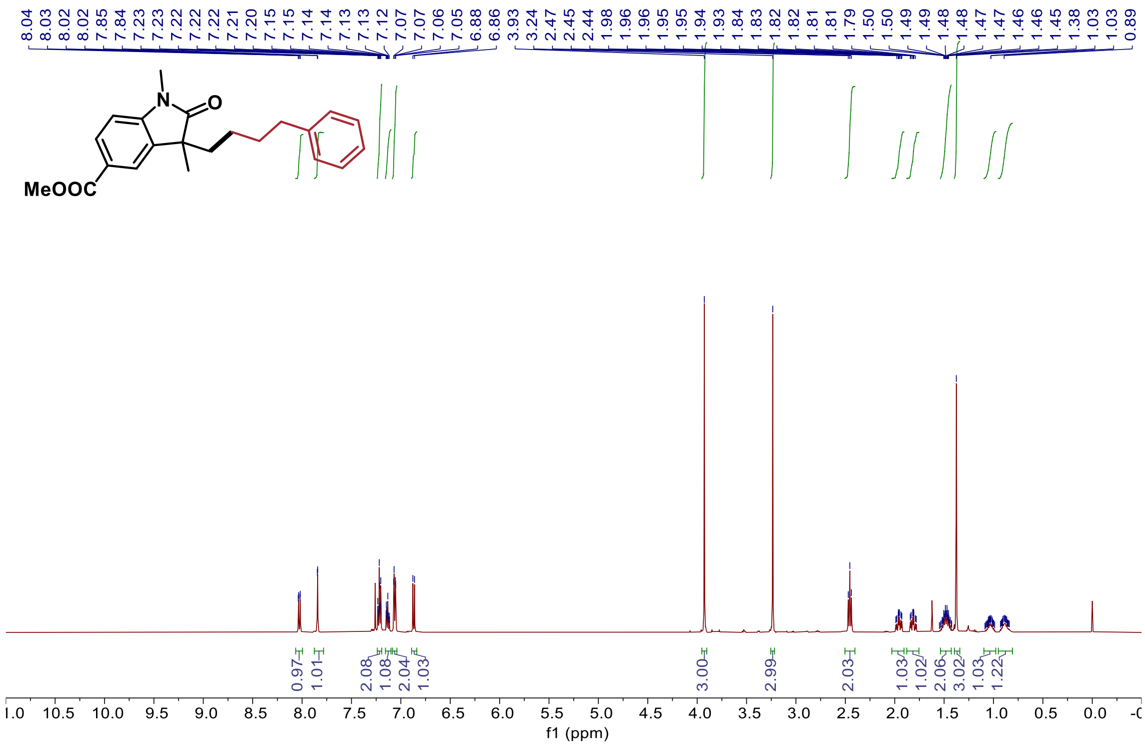

^{13}C NMR of 7 (126 MHz, $CDCl_3$)

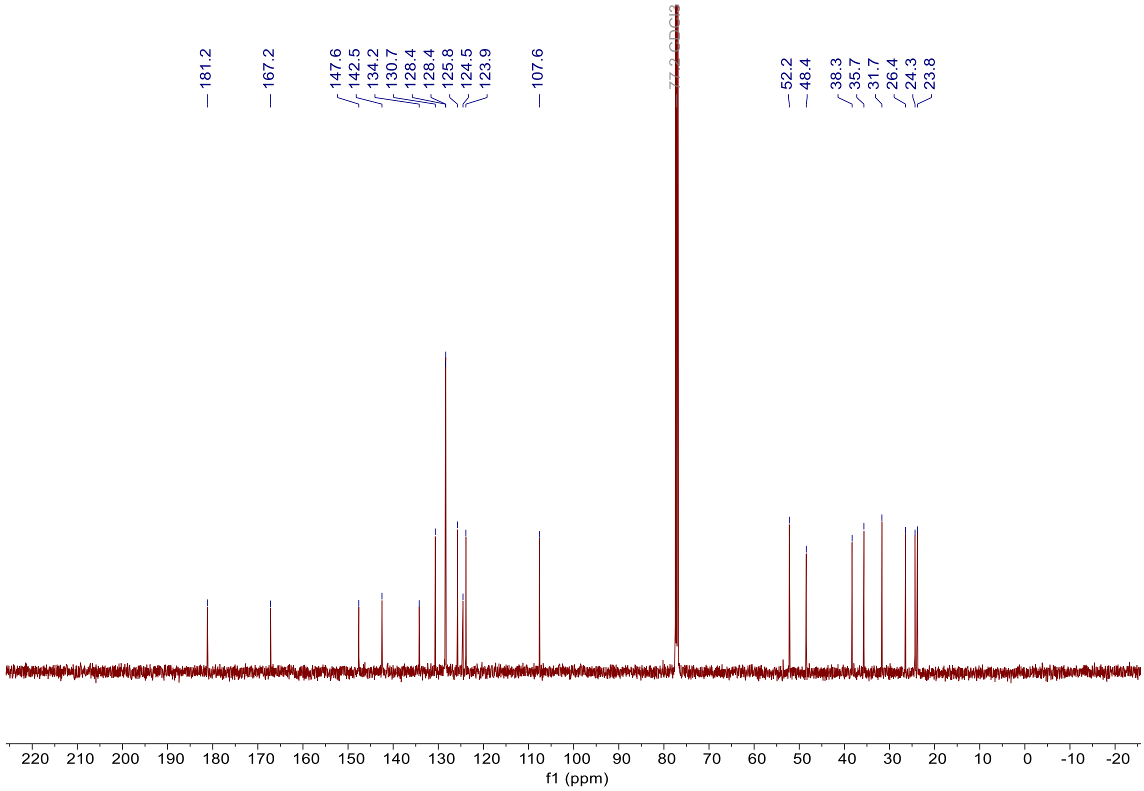

^{1}H NMR of 8 (500 MHz, $CDCl_3$)

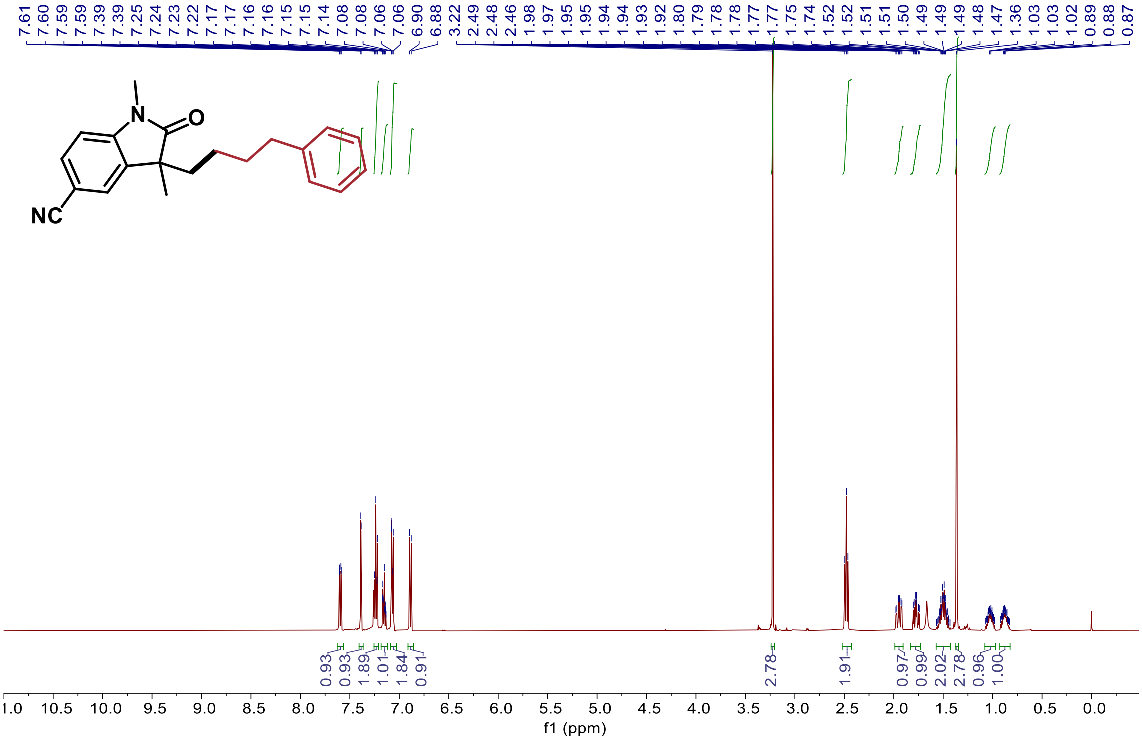

^{13}C NMR of 8 (126 MHz, $CDCl_3$)

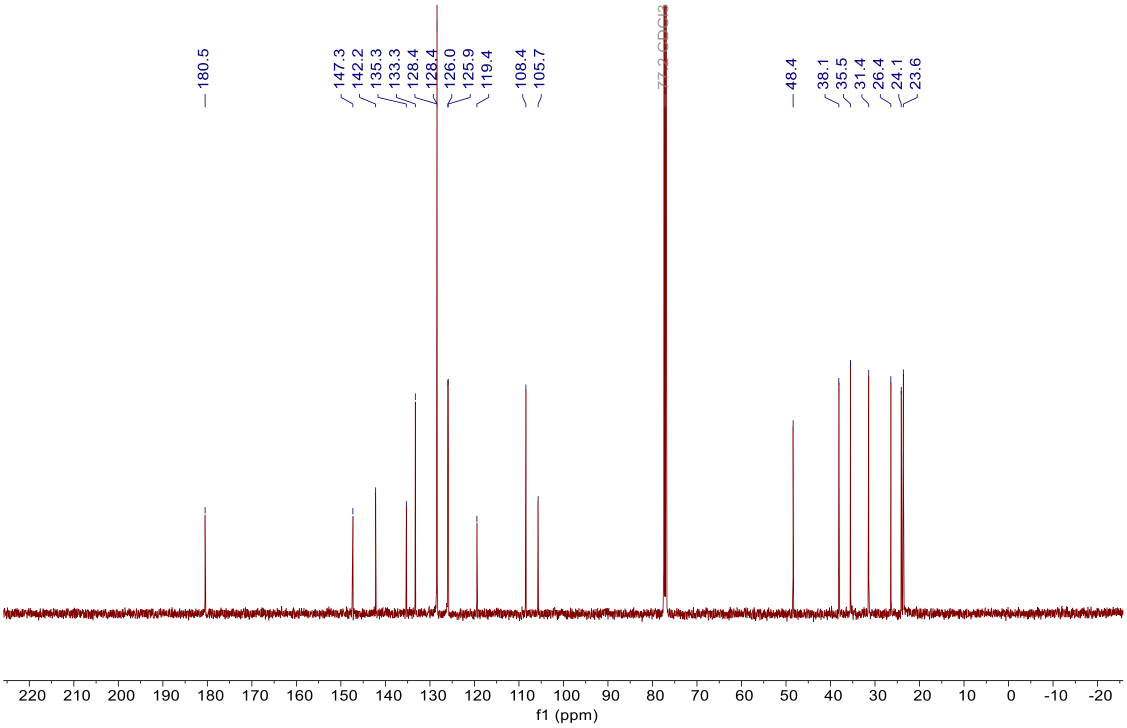

^{1}H NMR of 9 (500 MHz, $CDCl_3$)

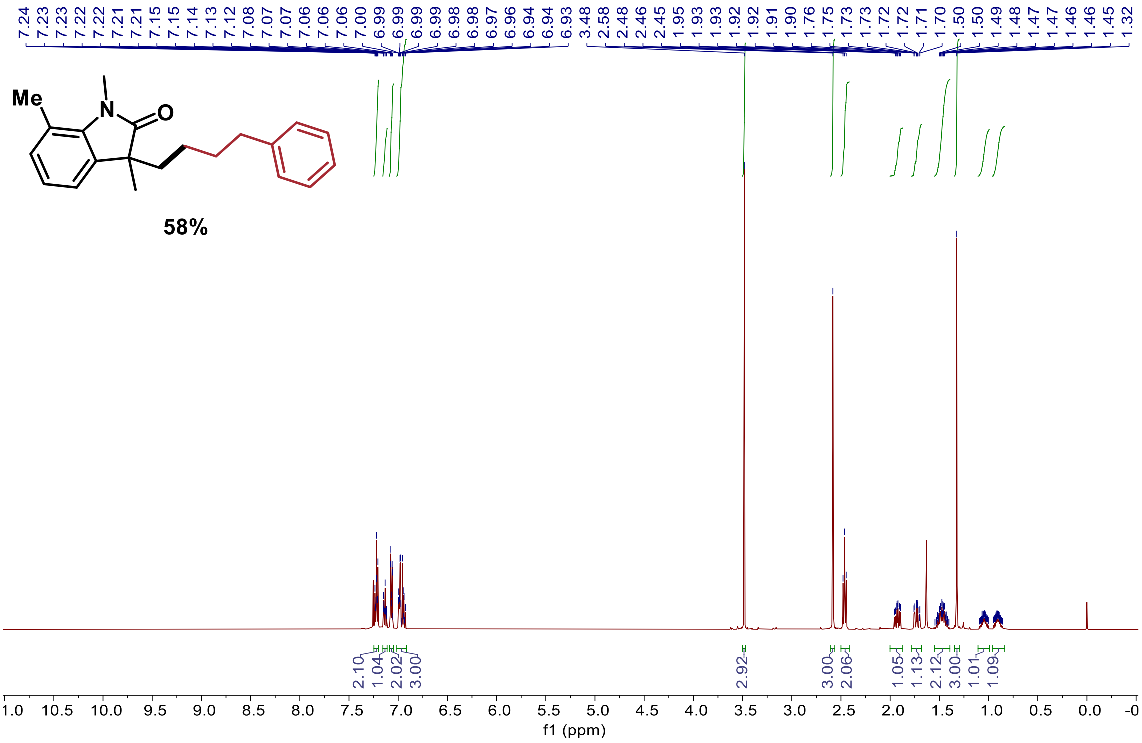

^{13}C NMR of 9 (126 MHz, $CDCl_3$)

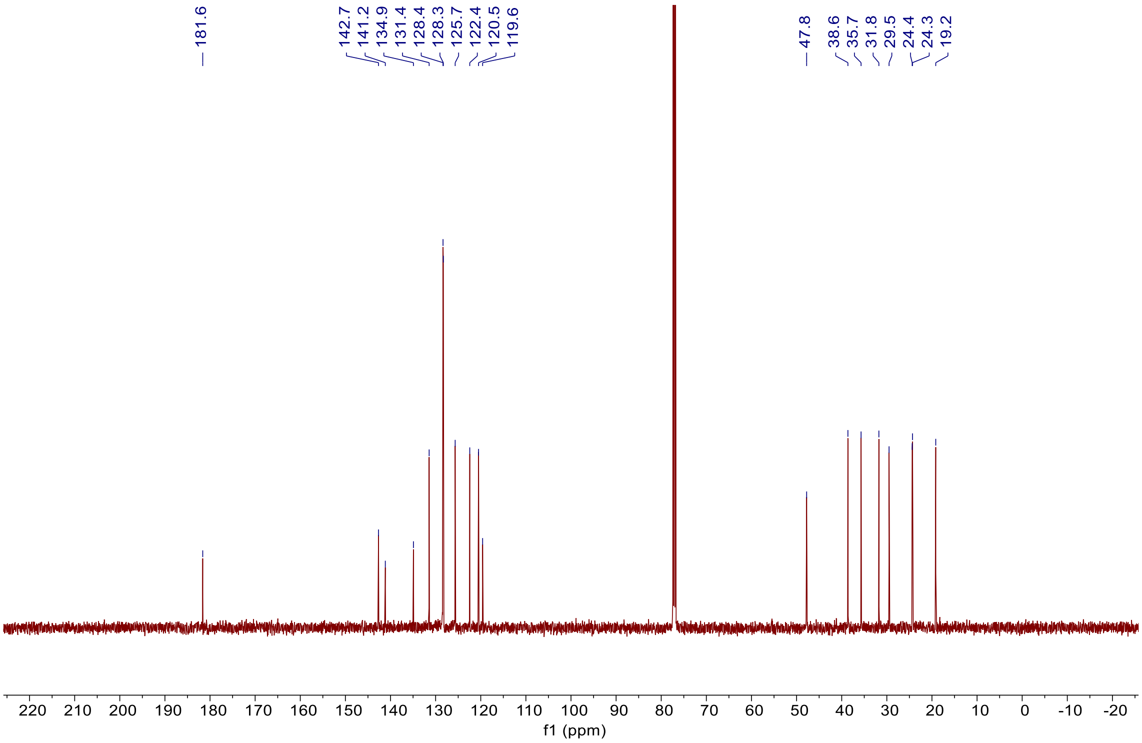

^{1}H NMR of 10 (500 MHz, $CDCl_3$)

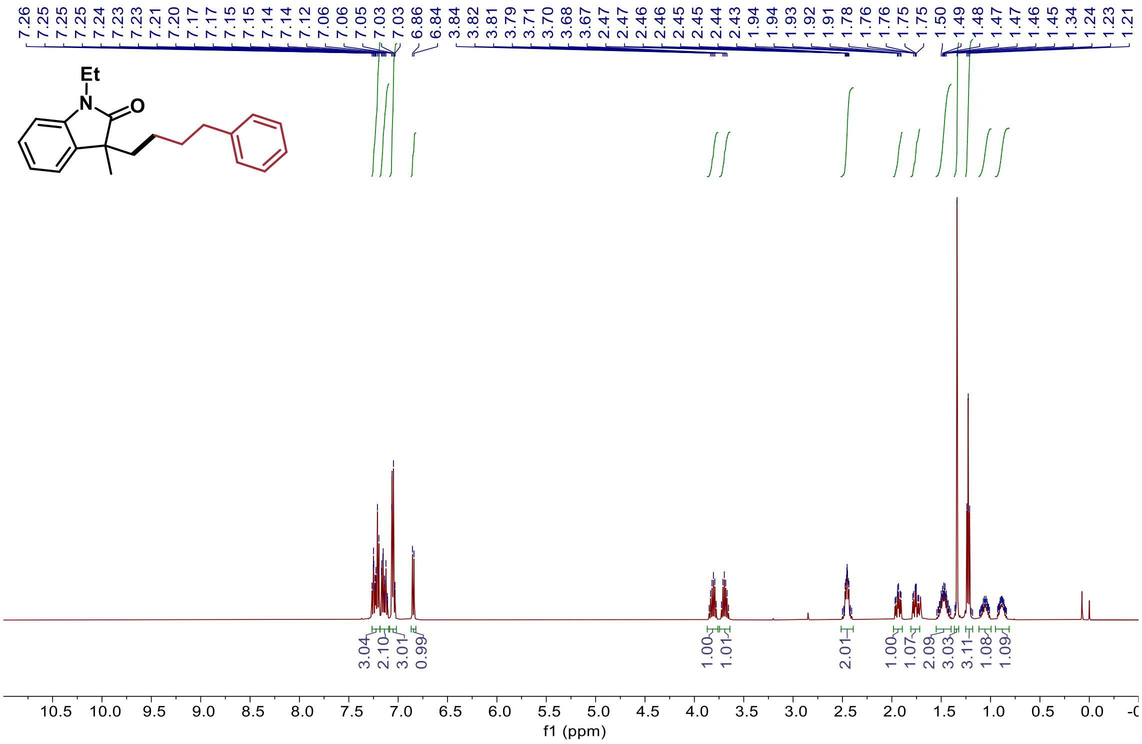

^{13}C NMR of 10 (126 MHz, $CDCl_3$)

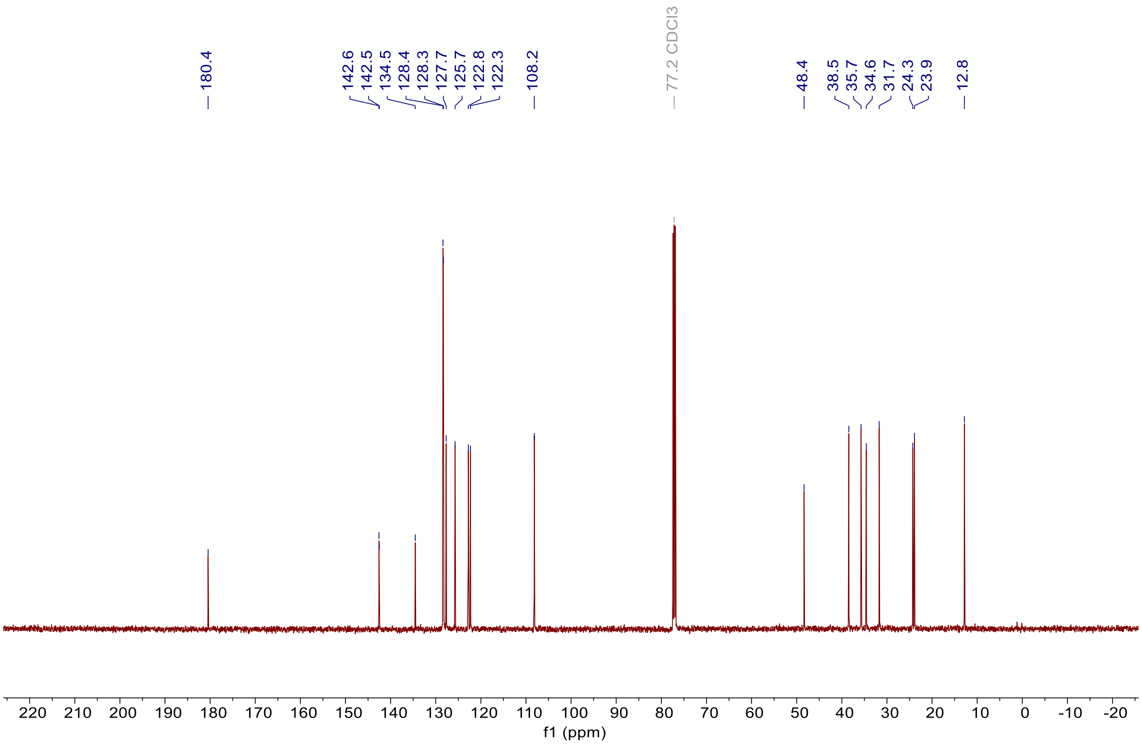

^{1}H NMR of 11 (500 MHz, $CDCl_3$)

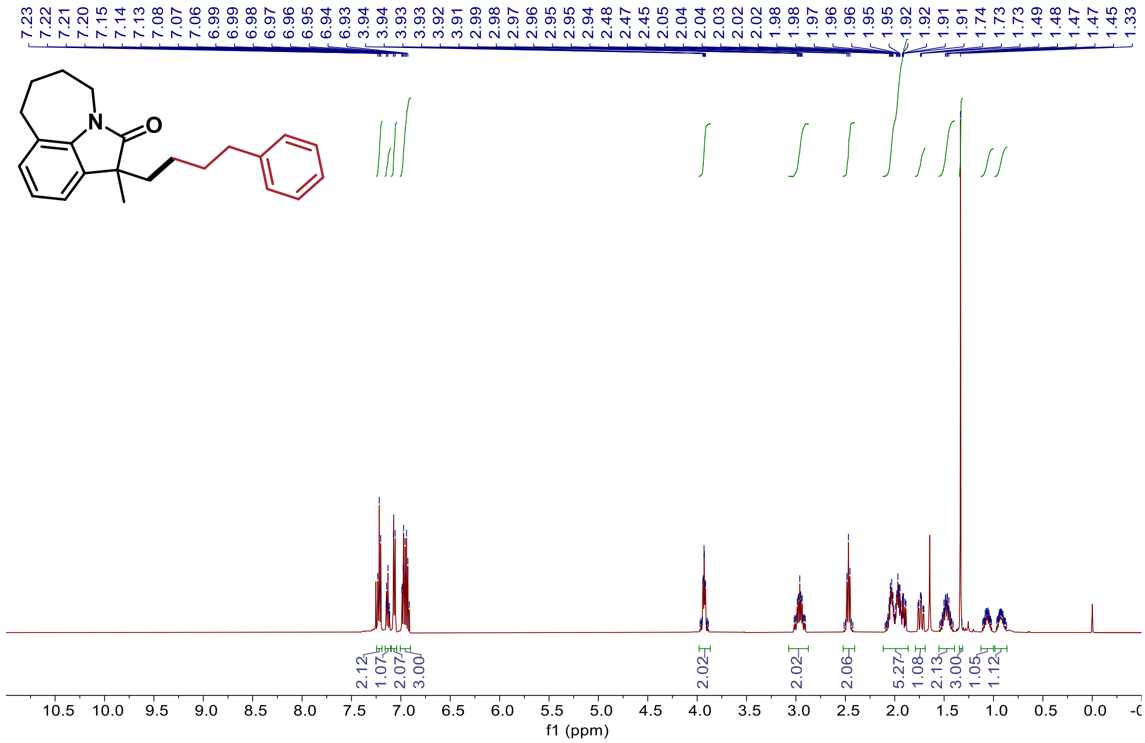

^{13}C NMR of 11 (126 MHz, $CDCl_3$)

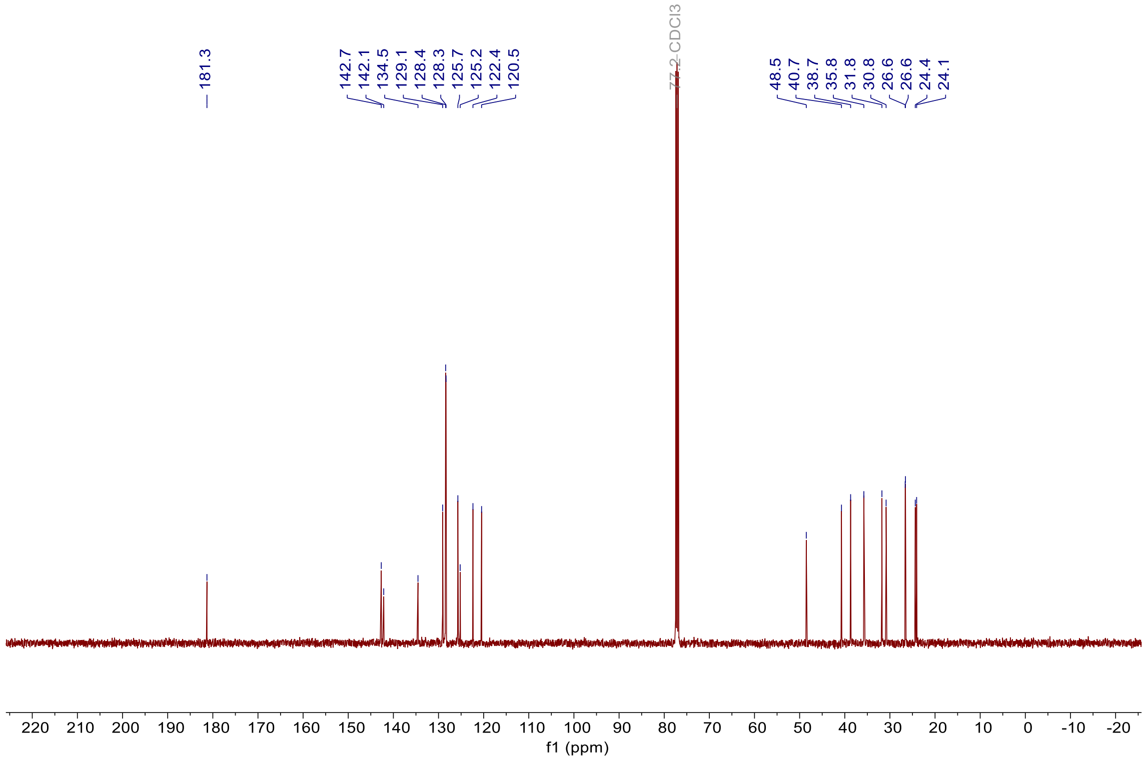

^{1}H NMR of 12 (500 MHz, $CDCl_3$)

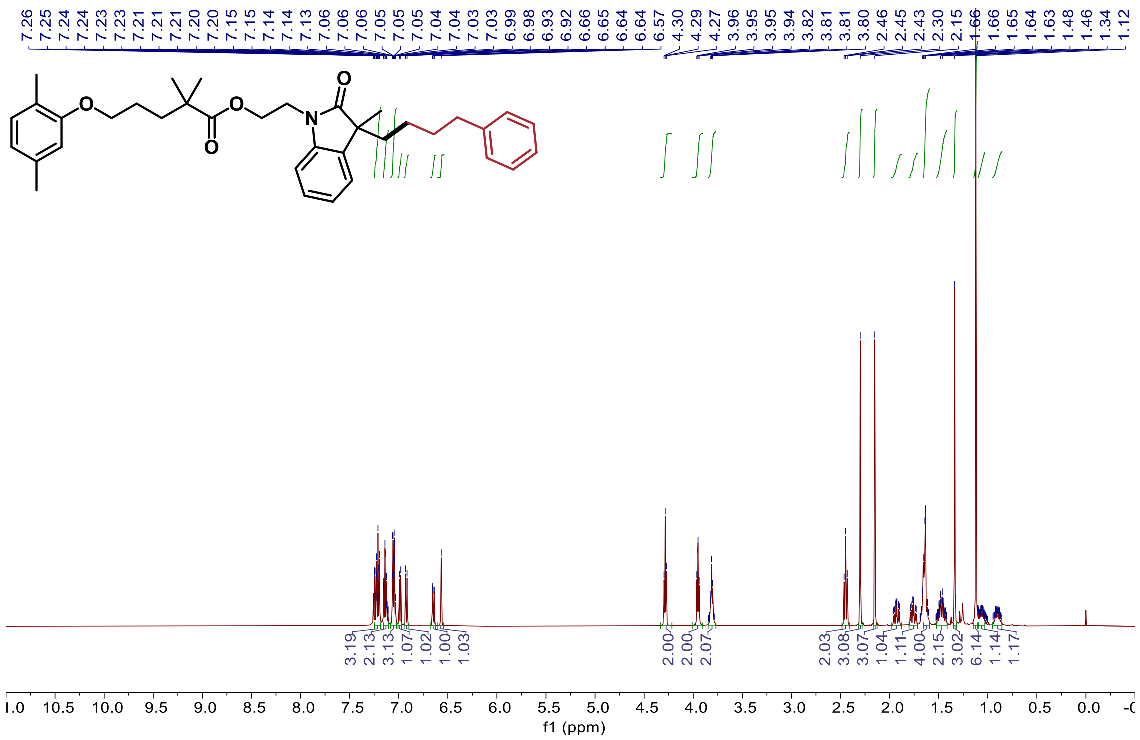

^{13}C NMR of 12 (151 MHz, $CDCl_3$)

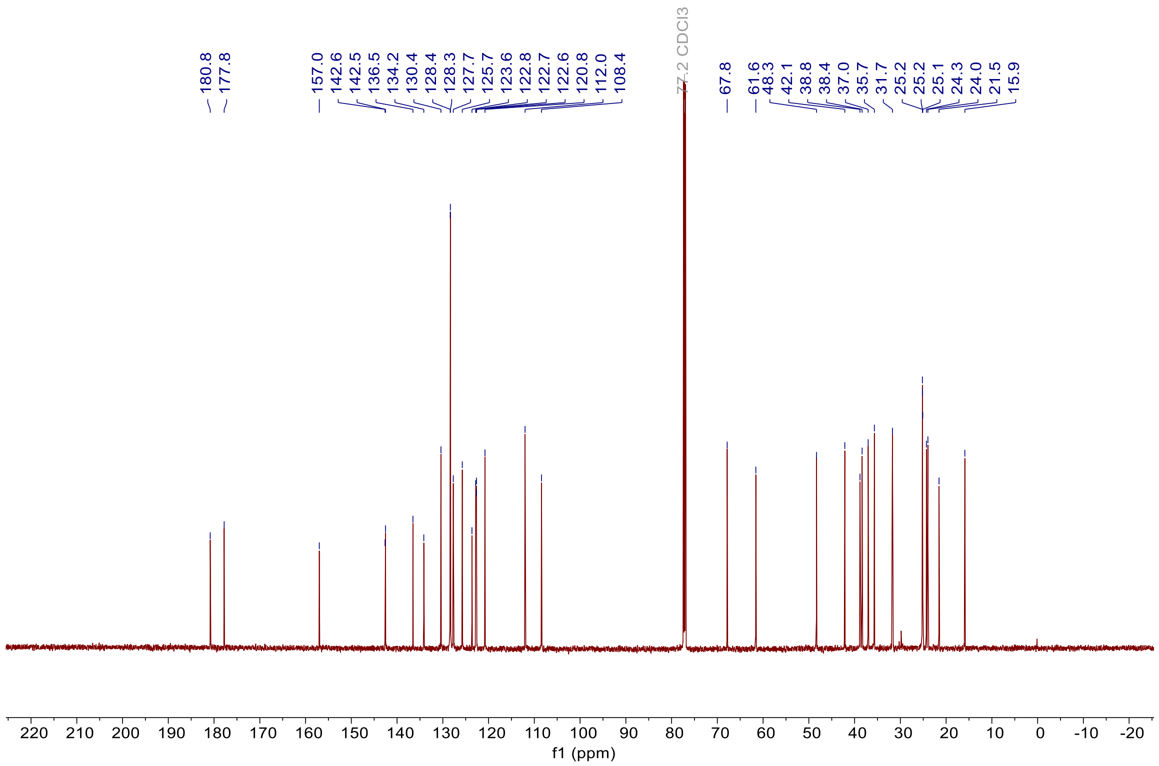

^{1}H NMR of 13 (500 MHz, $CDCl_3$)

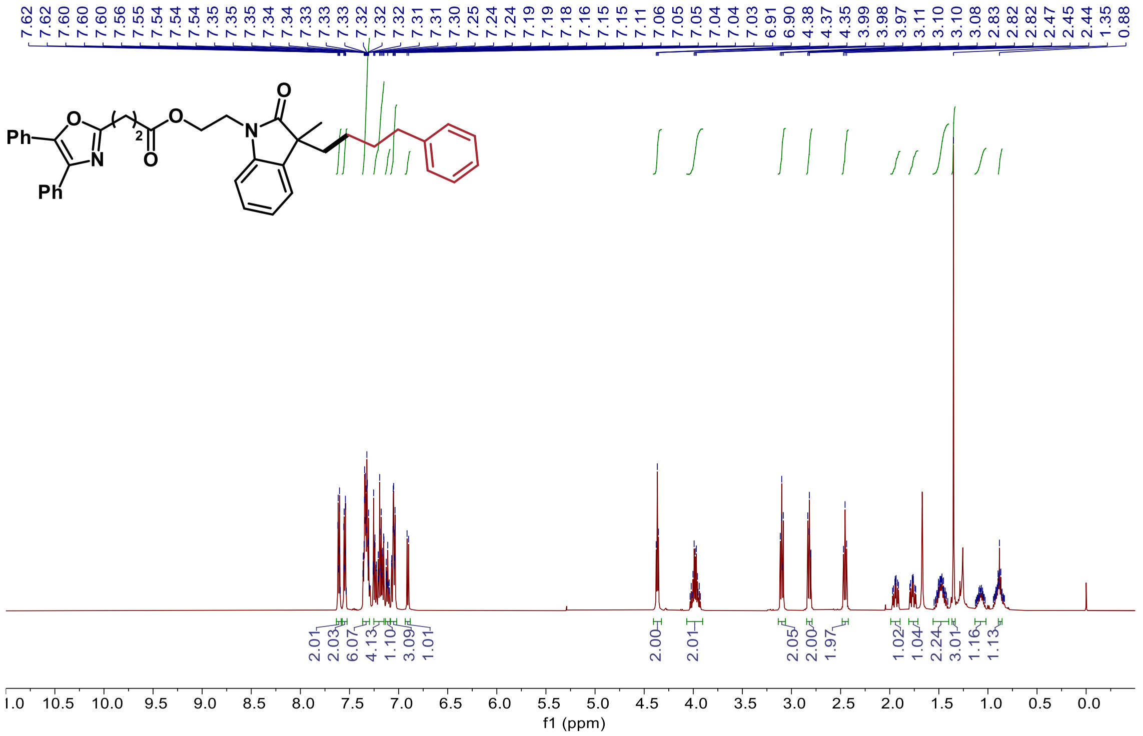

^{13}C NMR of 13 (101 MHz, $CDCl_3$)

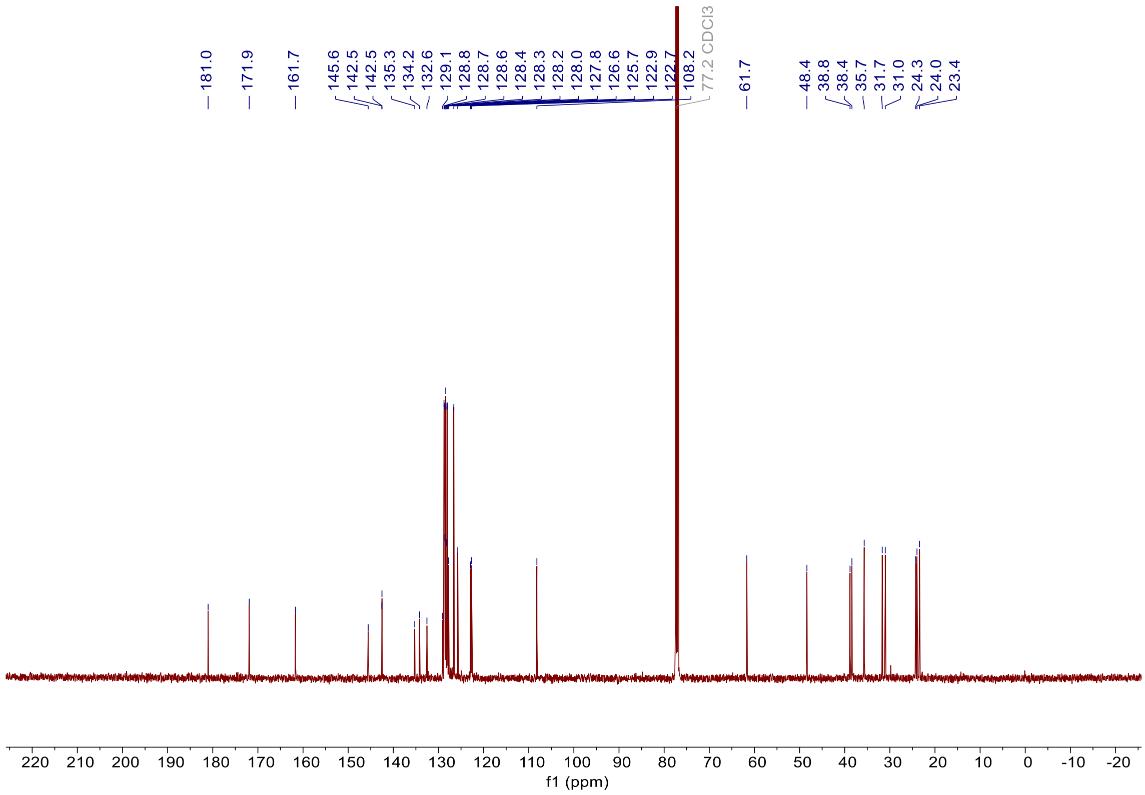

^{1}H NMR of 14 (500 MHz, $CDCl_3$)

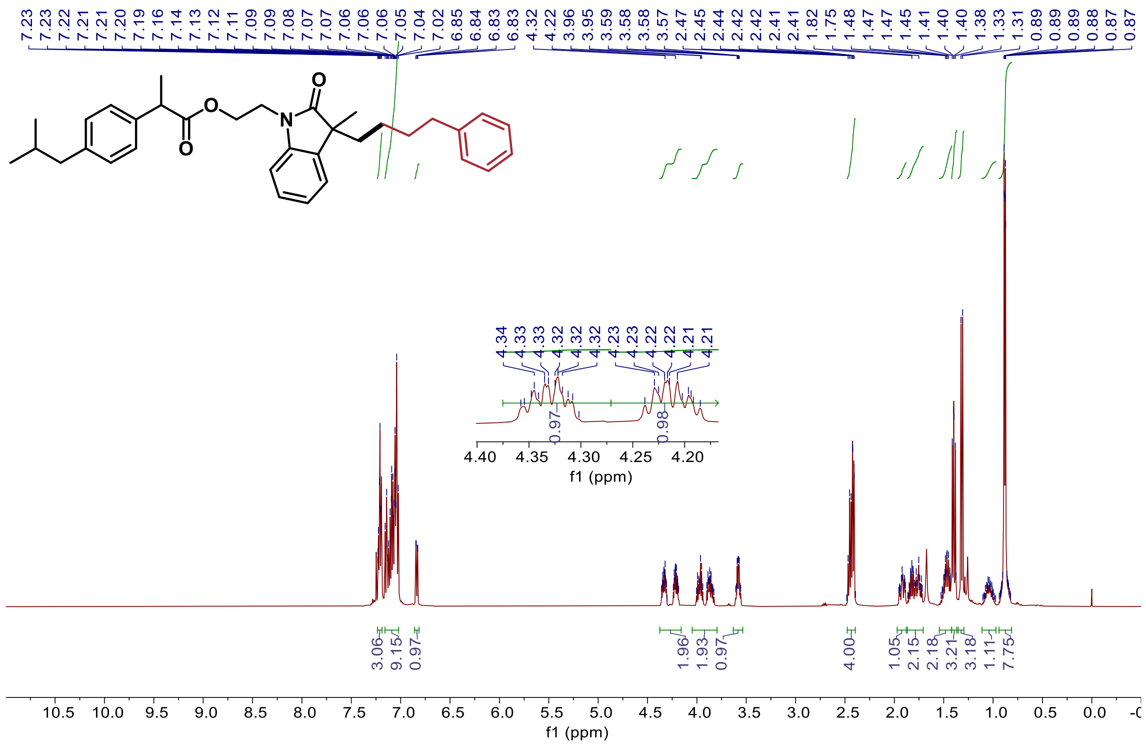

^{13}C NMR of 14 (151 MHz, $CDCl_3$)

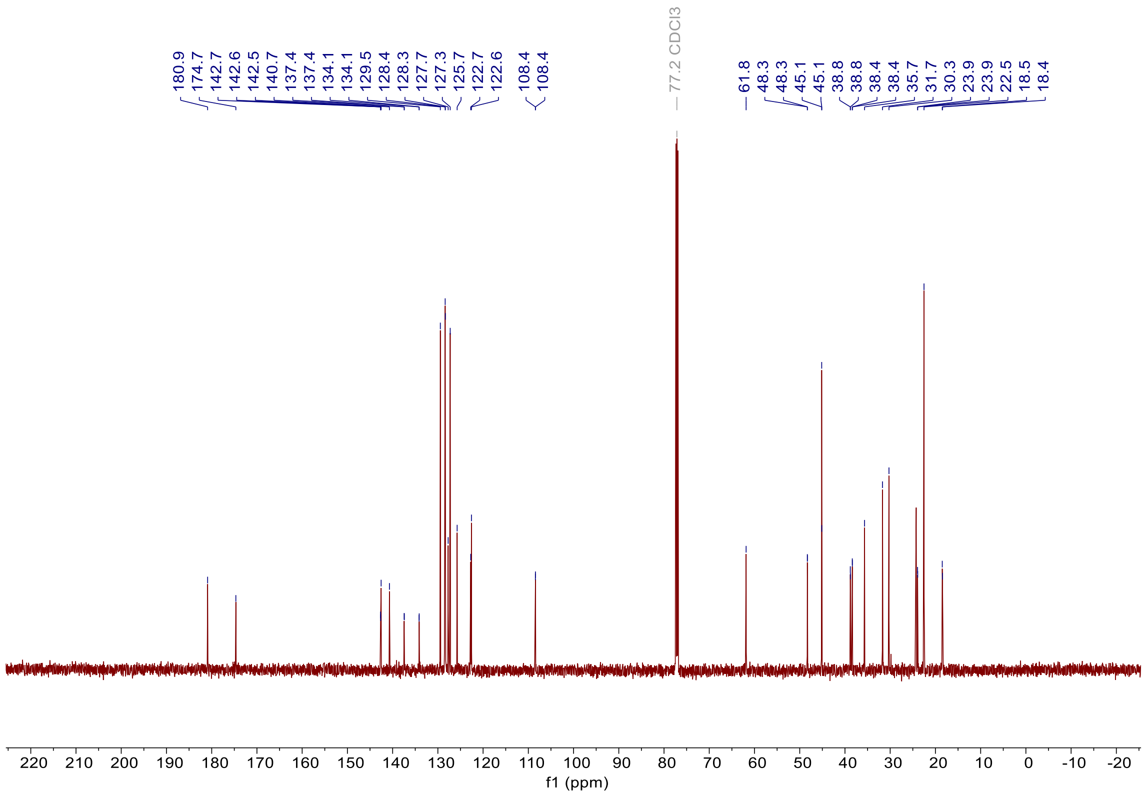

^{1}H NMR of 15 (400 MHz, CDCl$_3$)

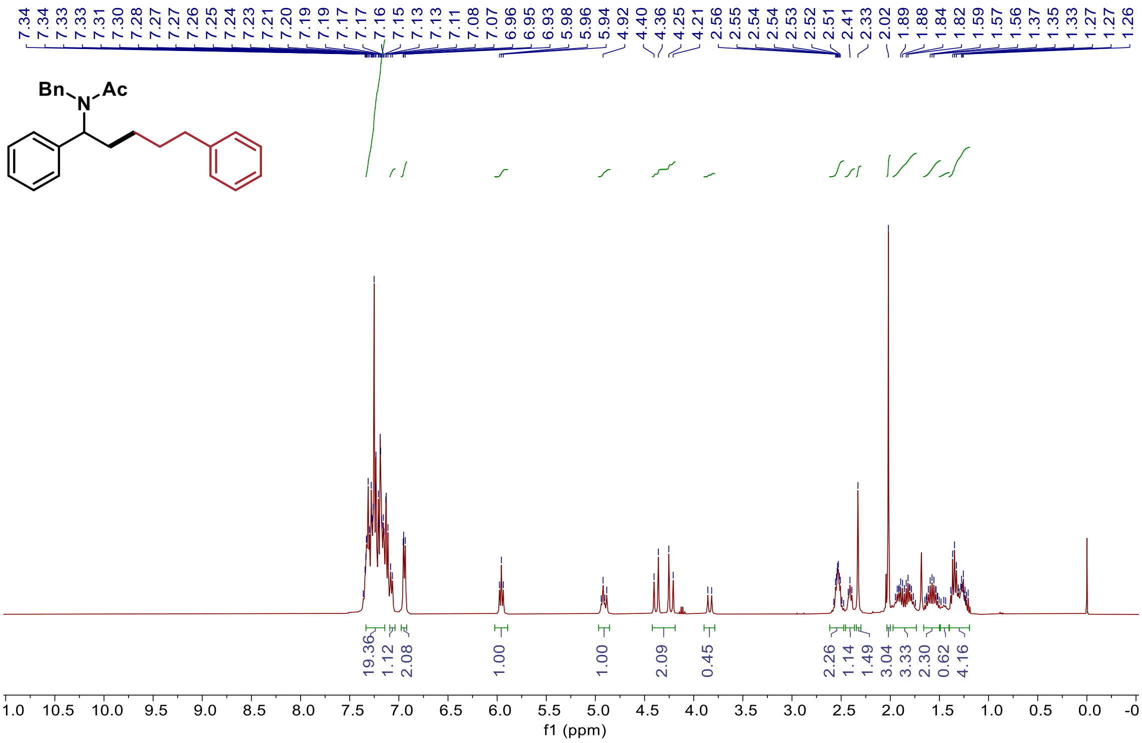

^{13}C NMR of 15 (151 MHz, CDCl$_3$)

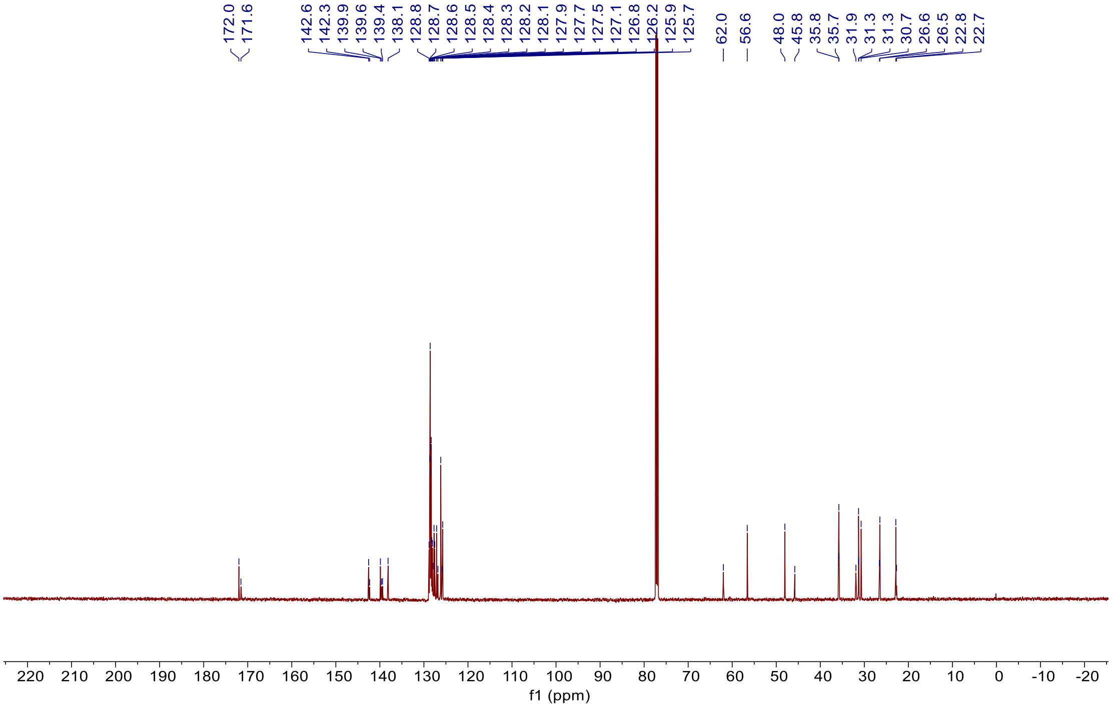

^{1}H NMR of 16 (500 MHz, $CDCl_3$)

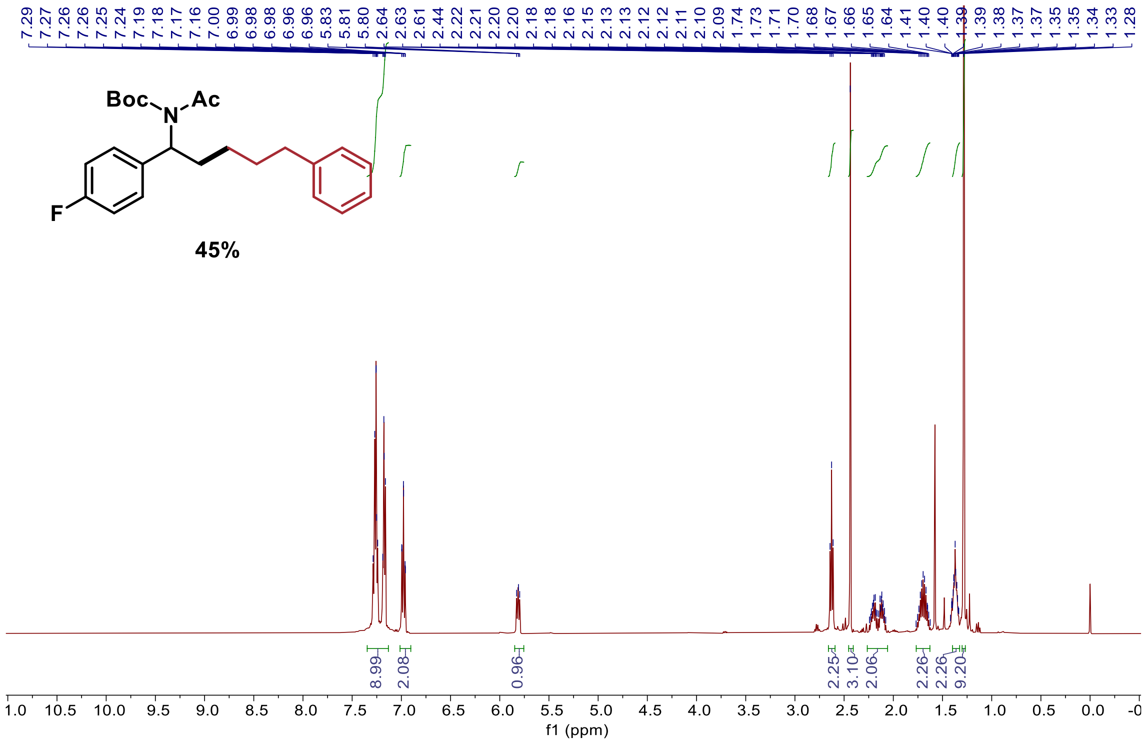

^{13}C NMR of 16 (126 MHz, $CDCl_3$)

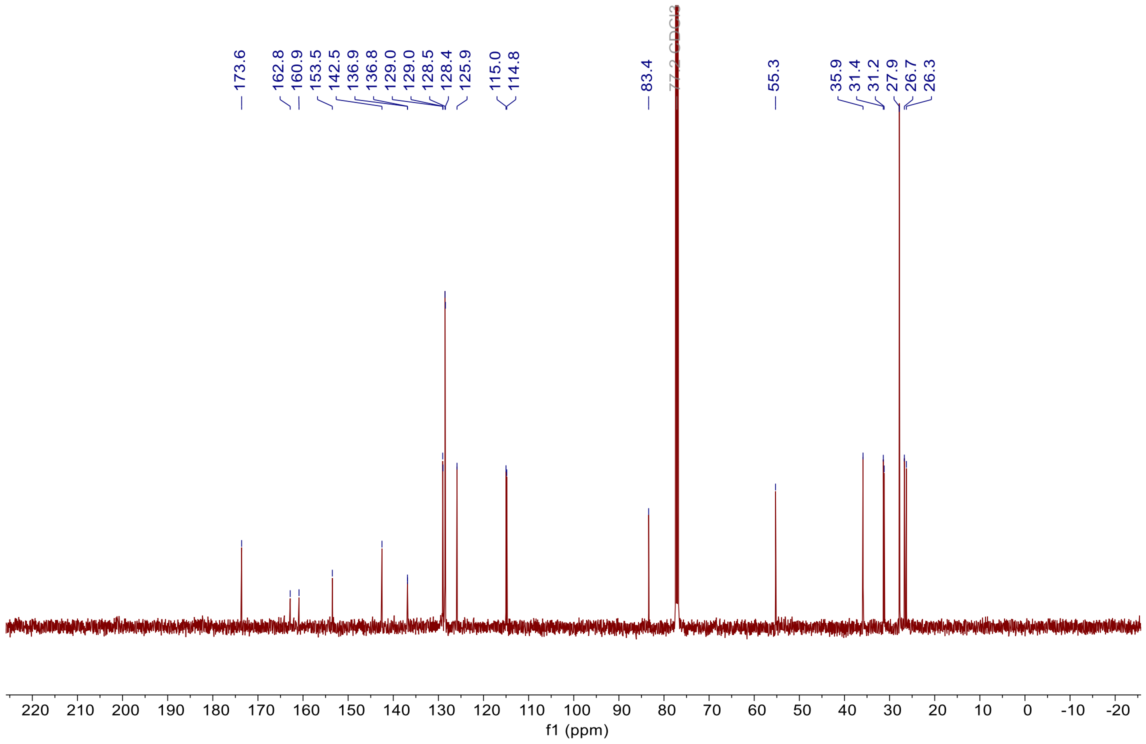

^{19}F NMR of 16 (376 MHz, $CDCl_3$)

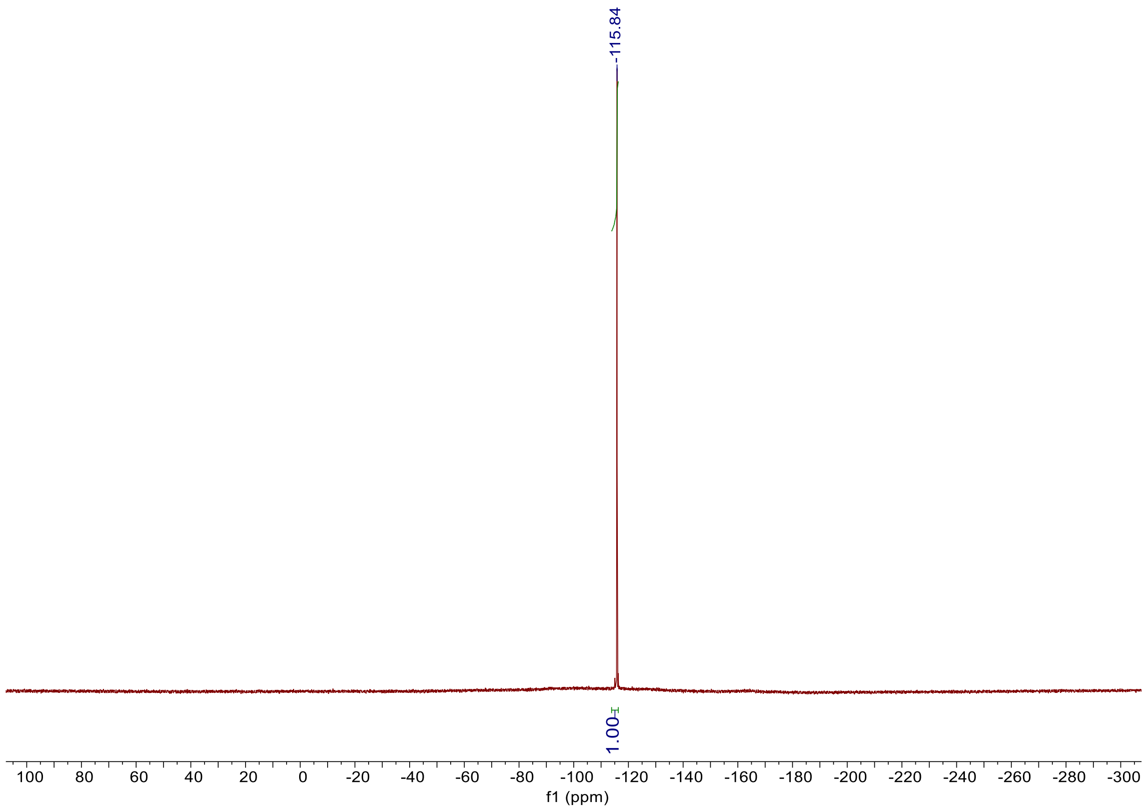

^{1}H NMR of 17 (600 MHz, CDCl$_3$)

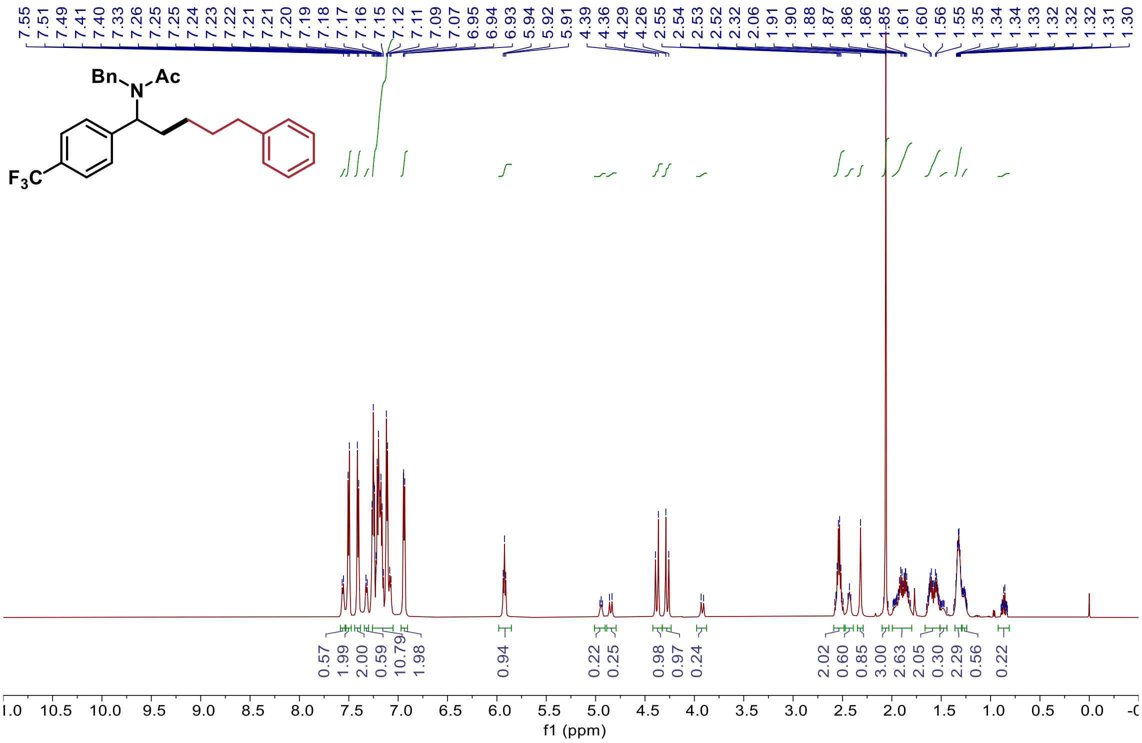

^{13}C NMR of 17 (126 MHz, CDCl$_3$)

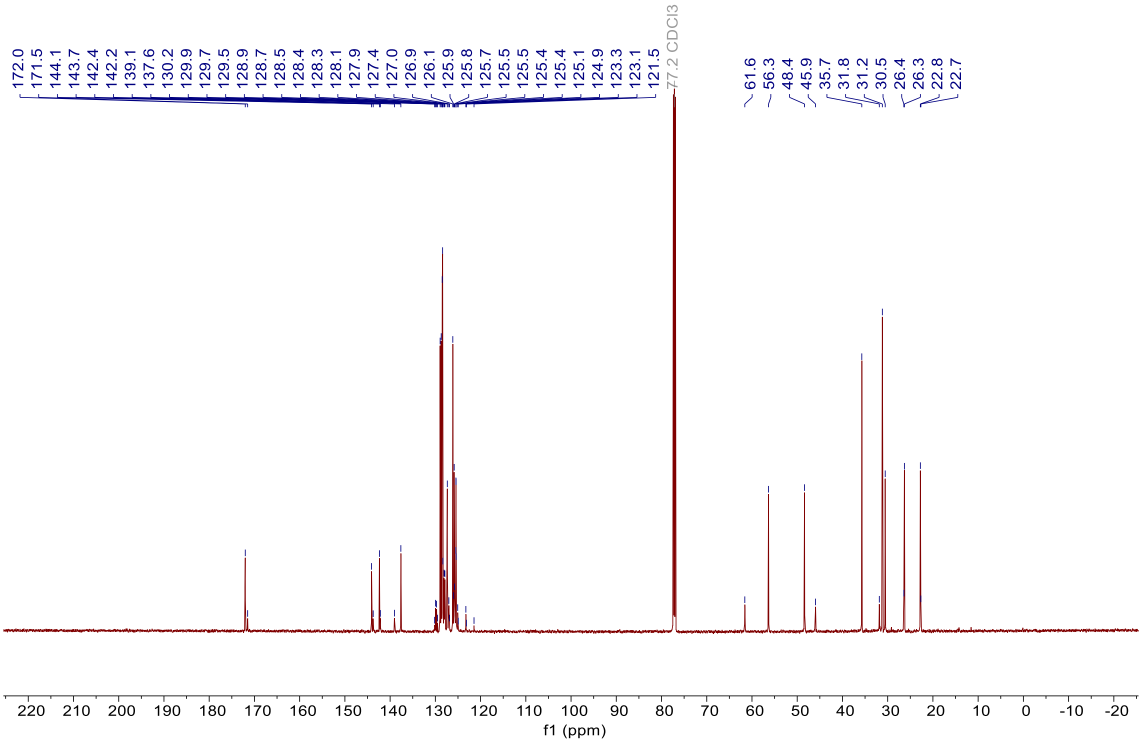

^{19}F NMR of 17 (565 MHz, $CDCl_3$)

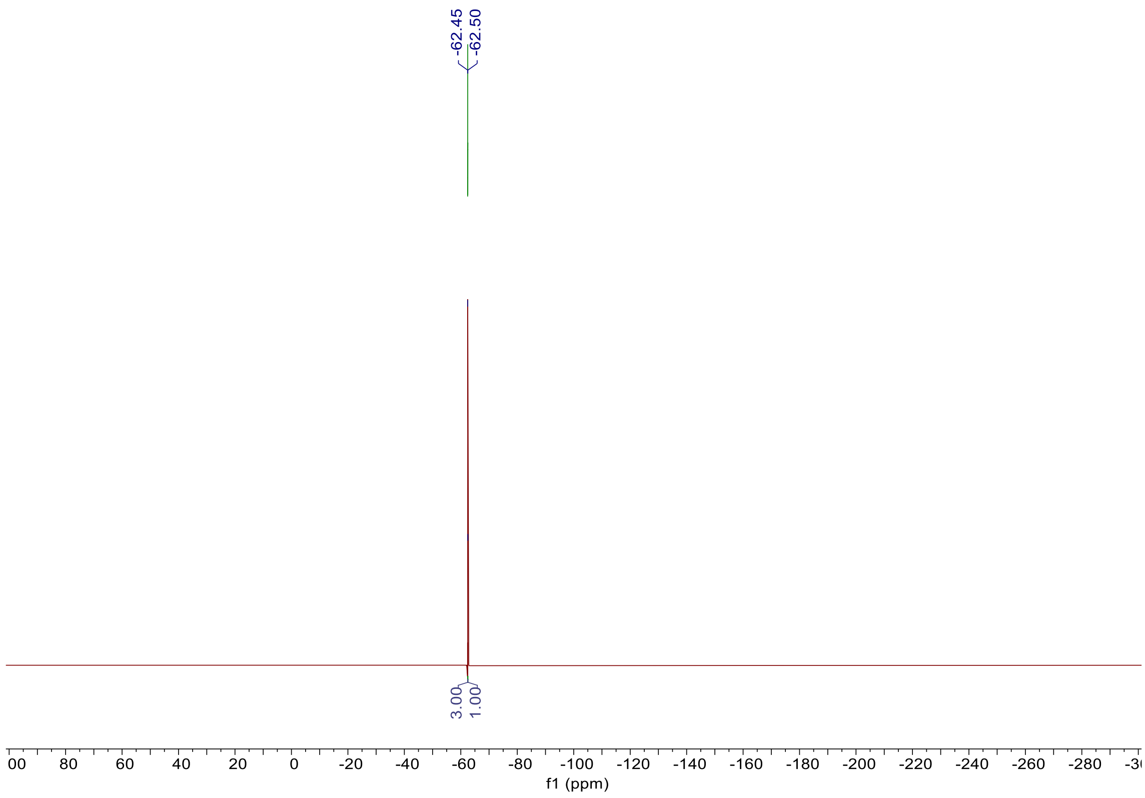

^{1}H NMR of 18 (400 MHz, CDCl$_3$)

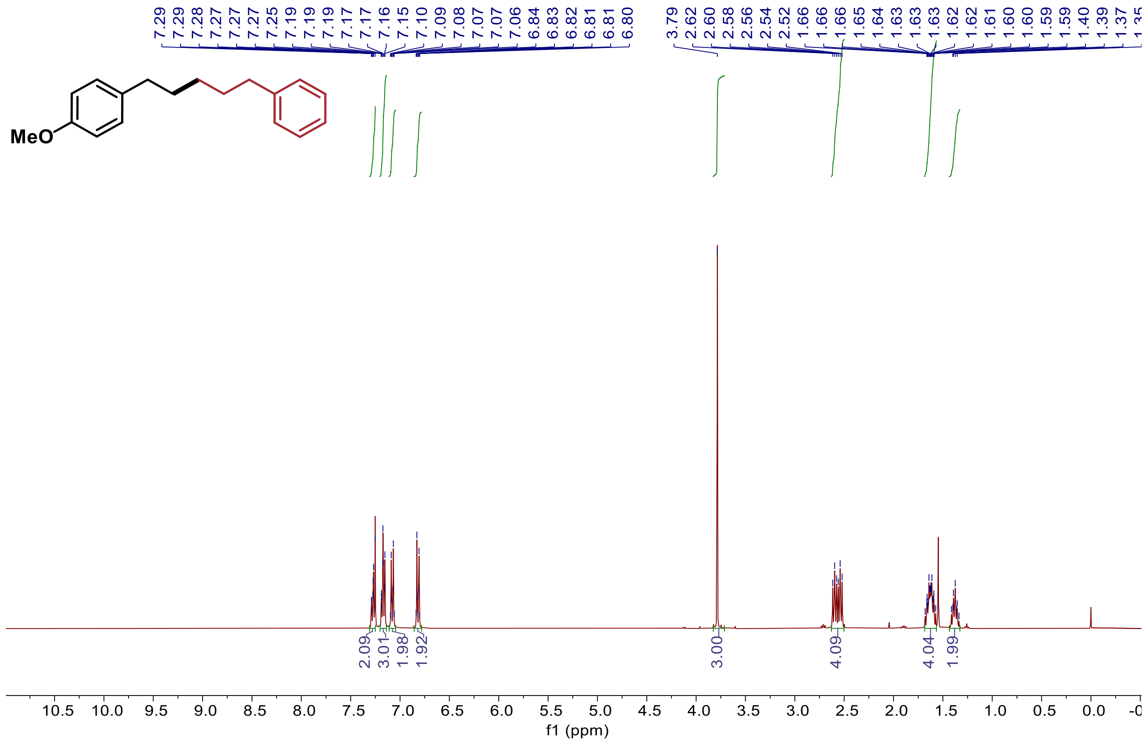

^{13}C NMR of 18 (101 MHz, CDCl$_3$)

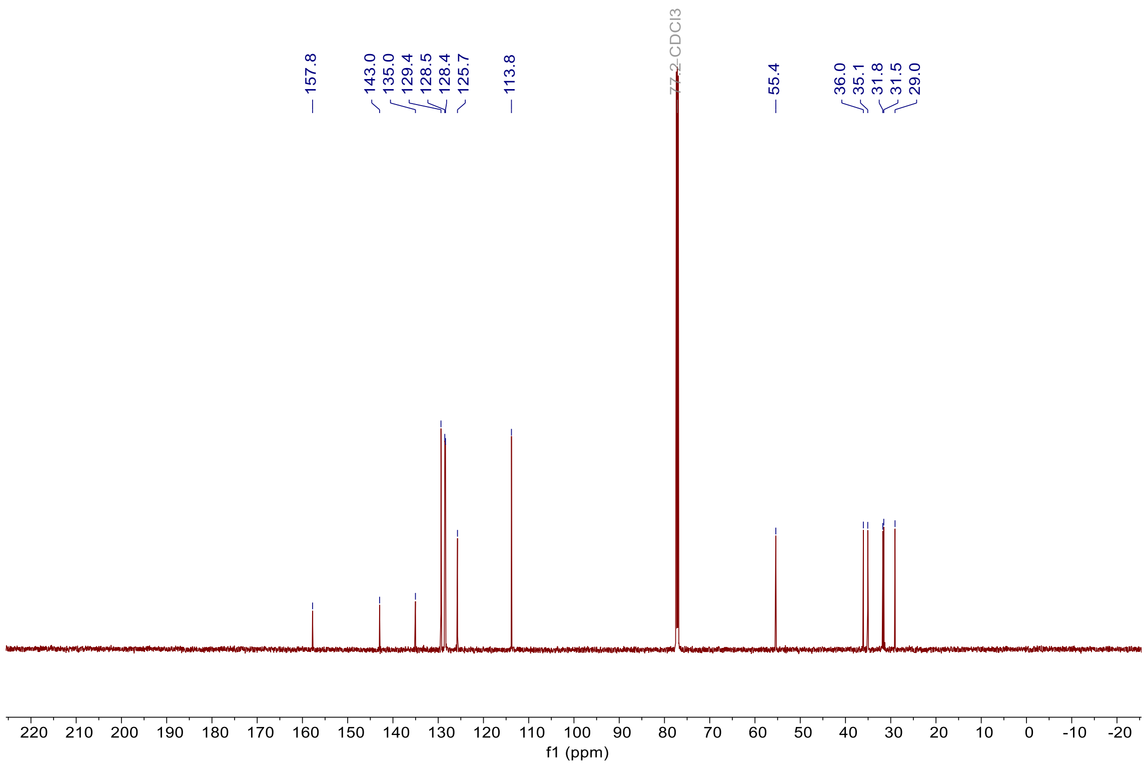

^{1}H NMR of19 (400 MHz, $CDCl_3$)

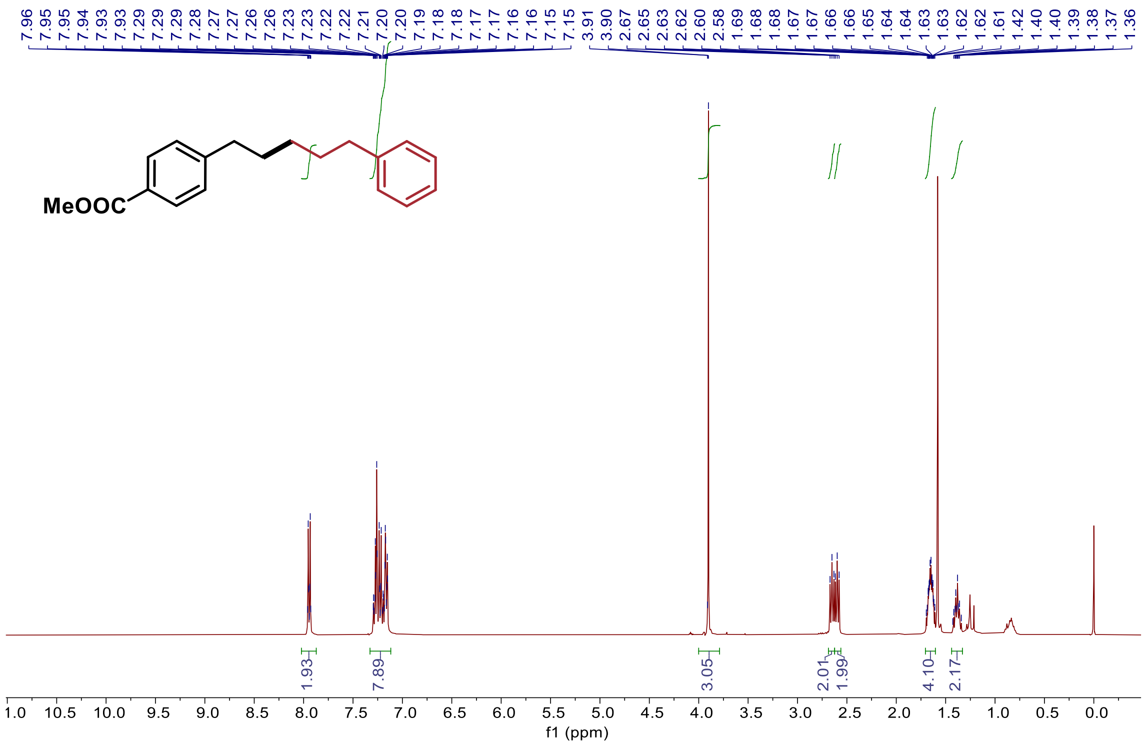

^{13}C NMR of 19 (101 MHz, $CDCl_3$)

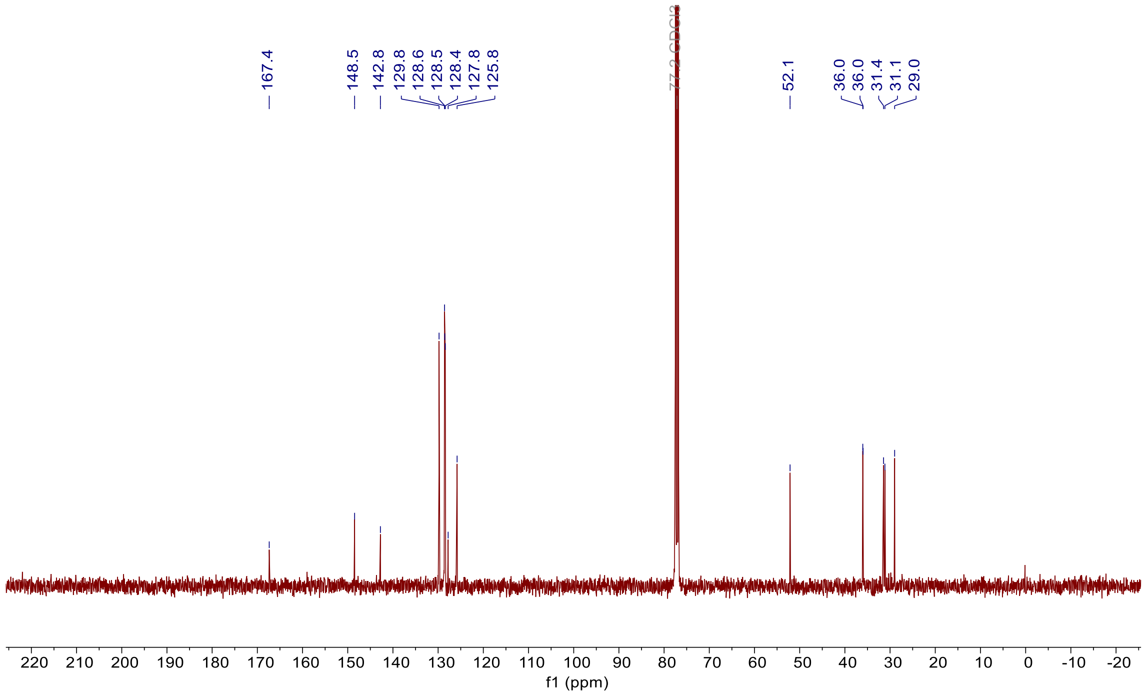

^{1}H NMR of 20 (600 MHz, CDCl$_3$)

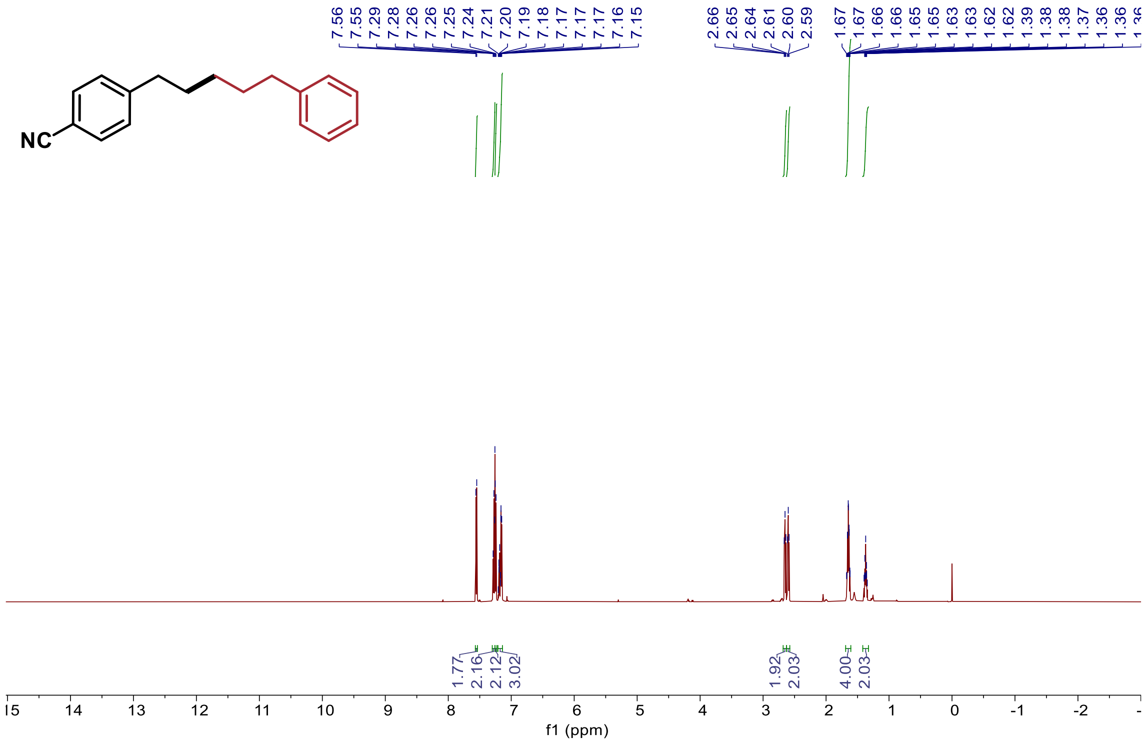

^{13}C NMR of 20 (151 MHz, CDCl$_3$)

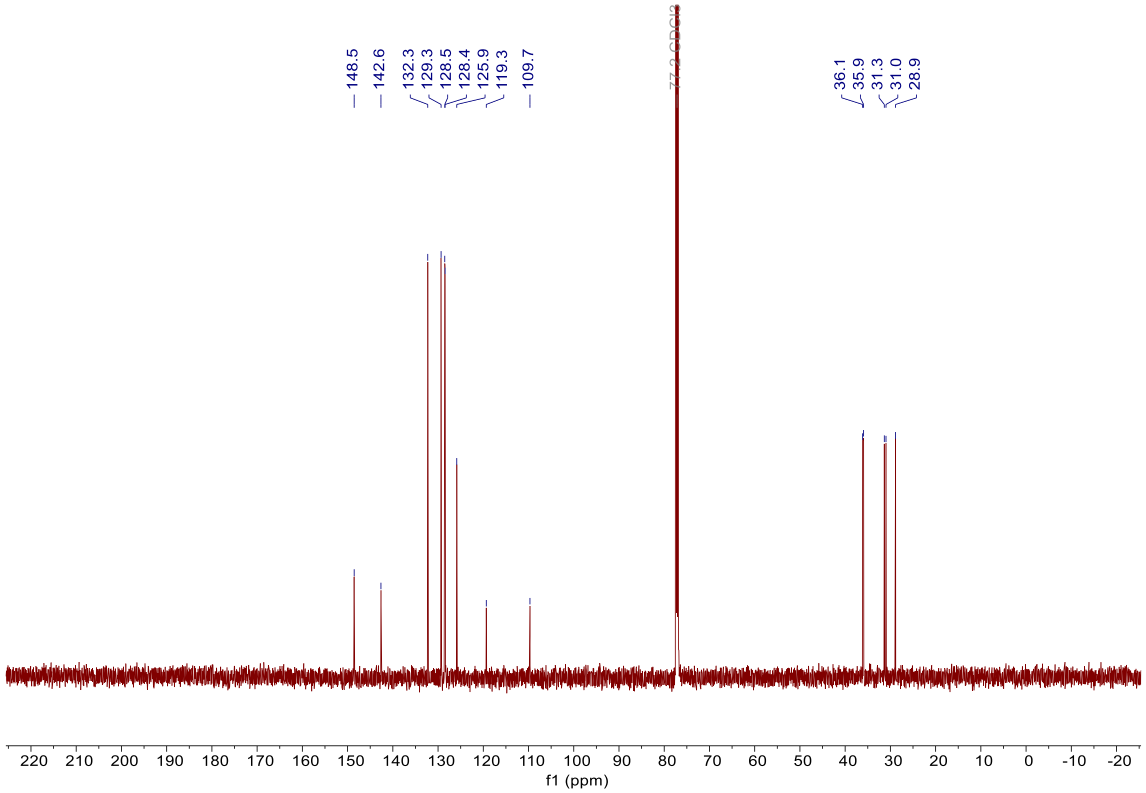

^{1}H NMR of 21 (500 MHz, $CDCl_3$)

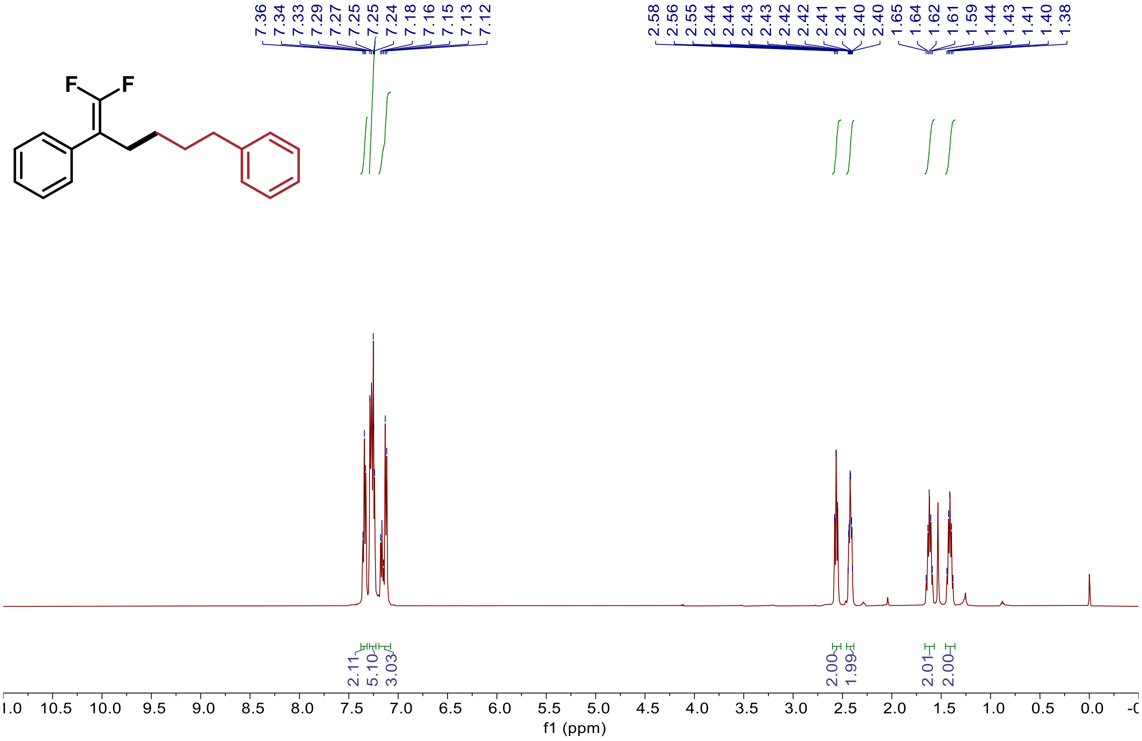

^{13}C NMR of 21 (126 MHz, $CDCl_3$)

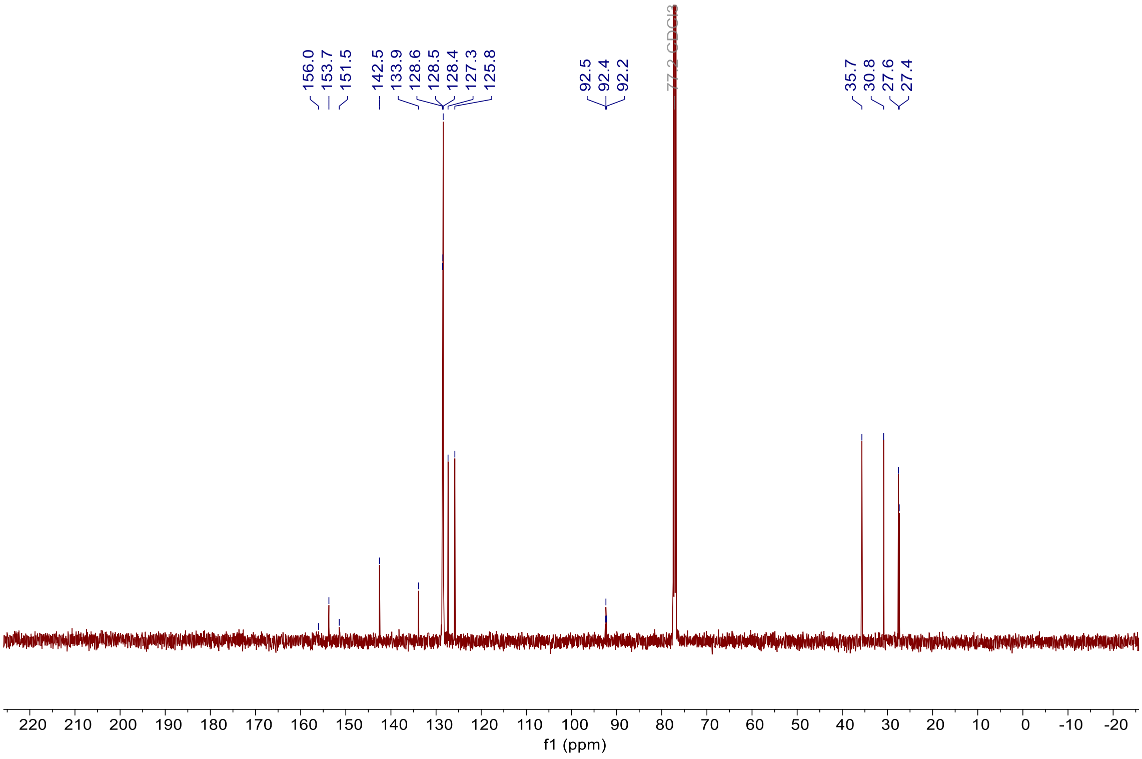

^{19}F NMR of 21 (471 MHz, $CDCl_3$)

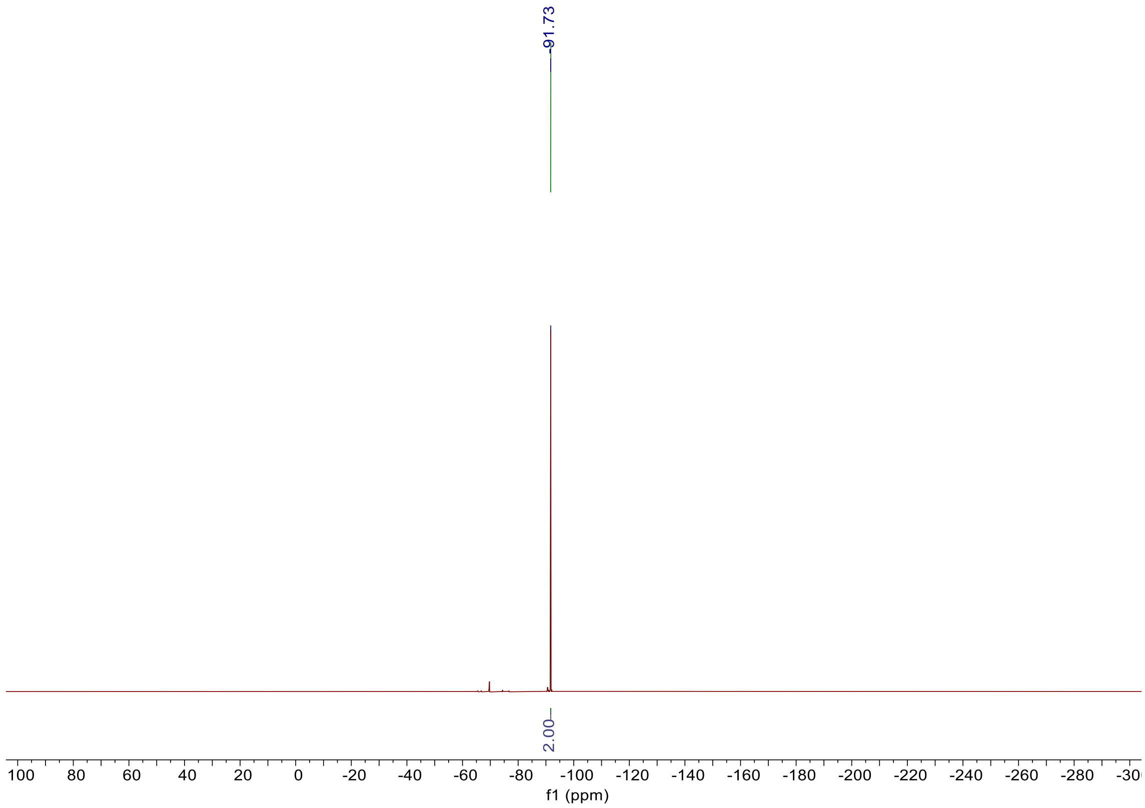

^{1}H NMR of 22 (400 MHz, $CDCl_3$)

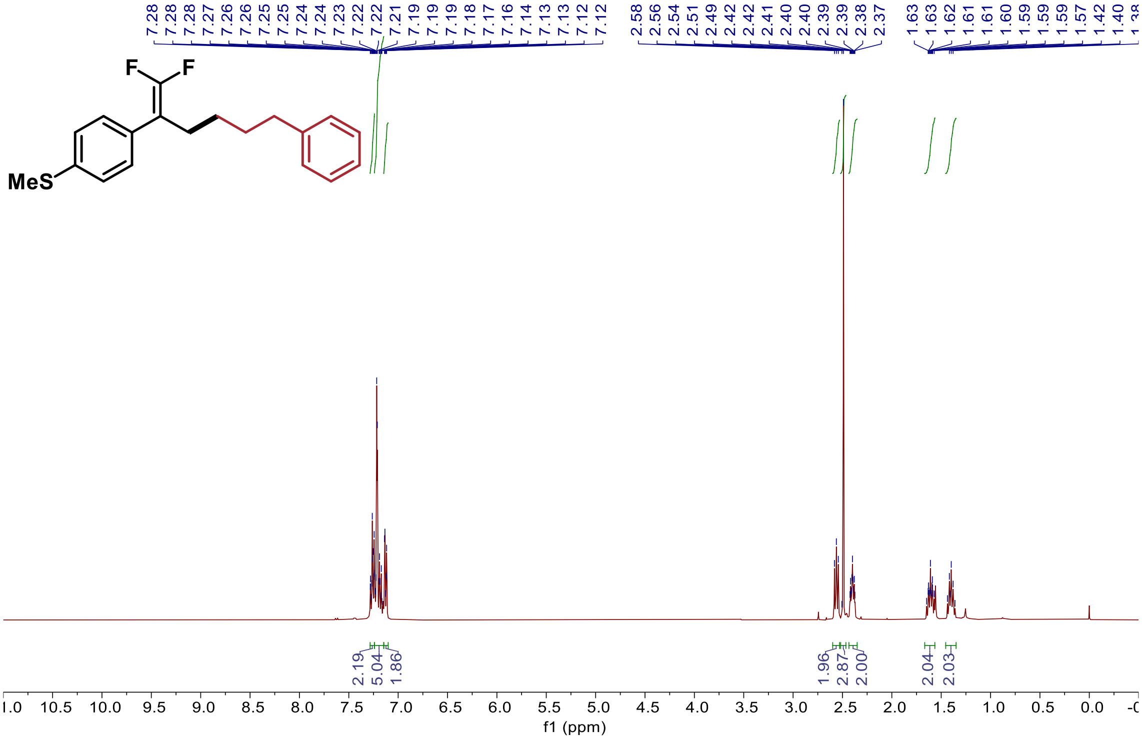

^{13}C NMR of 22 (101 MHz, $CDCl_3$)

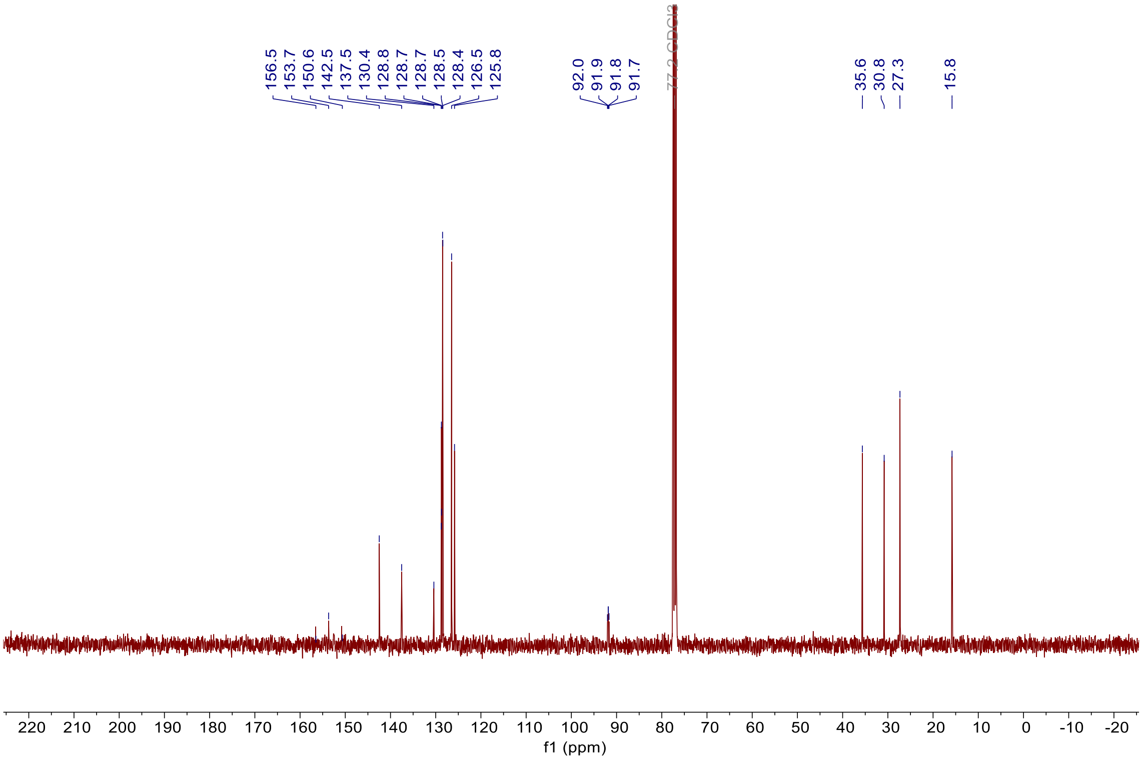

^{19}F NMR of 22 (376 MHz, $CDCl_3$)

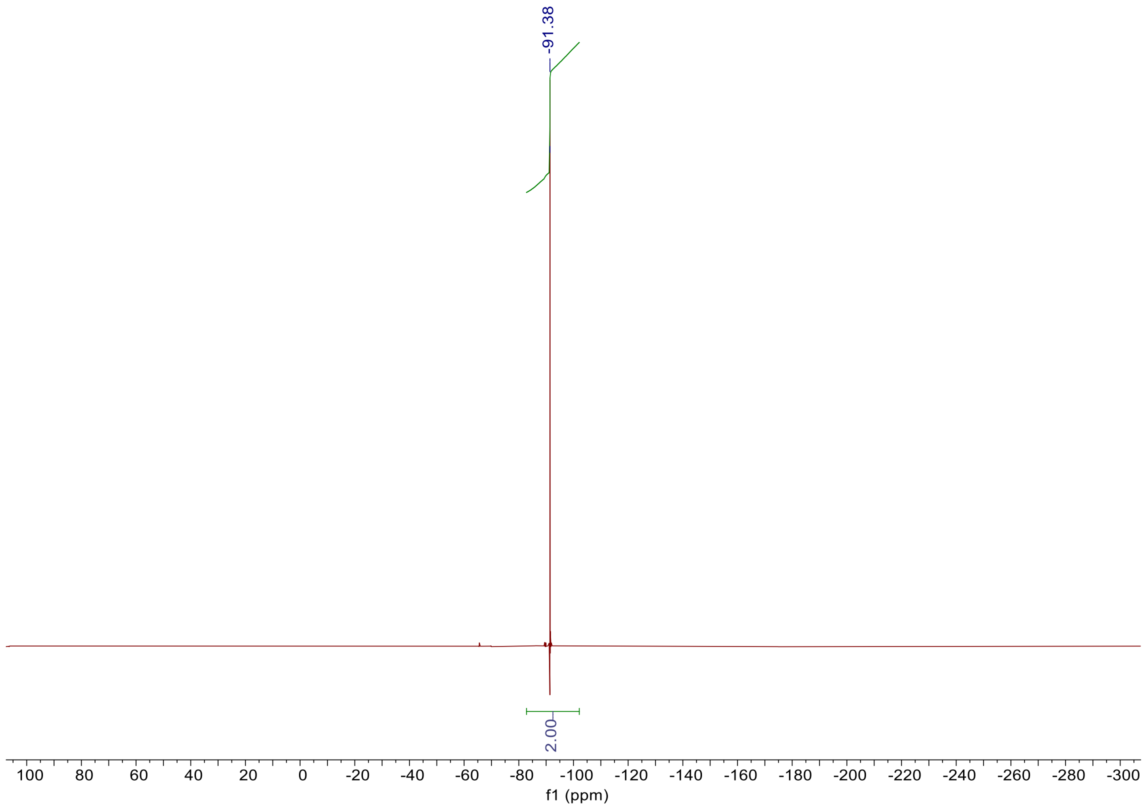

^{1}H NMR of 23 (400 MHz, CDCl$_3$)

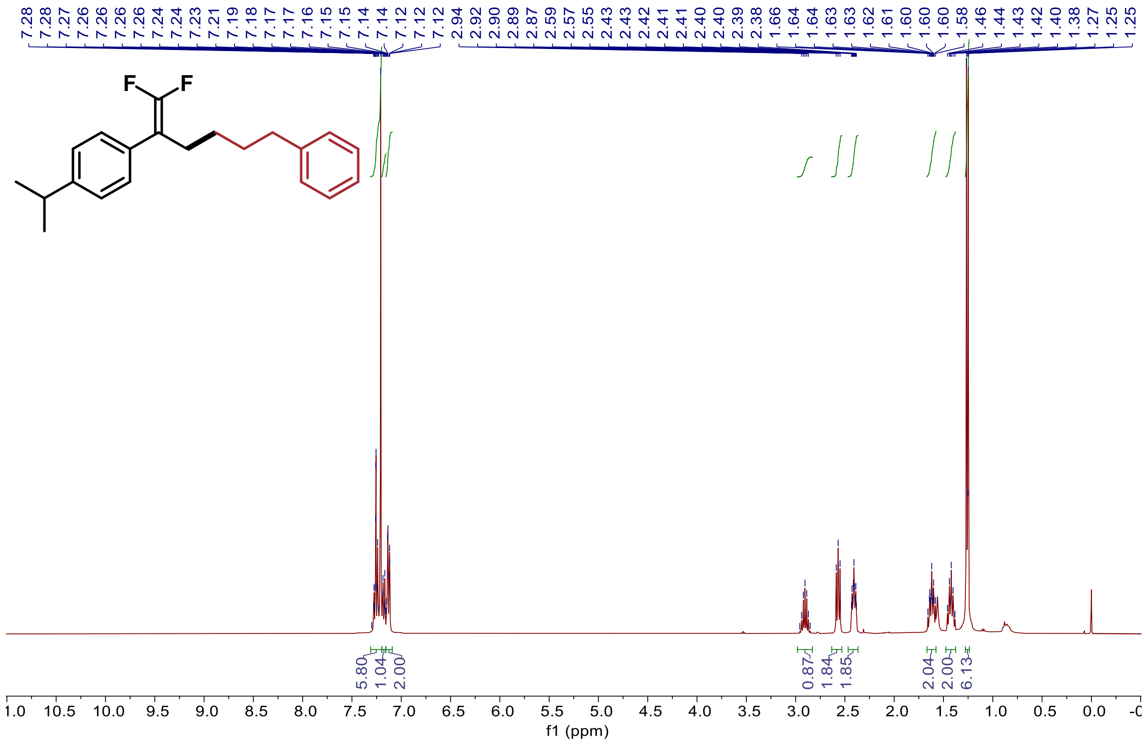

^{13}C NMR of 23 (126 MHz, CDCl$_3$)

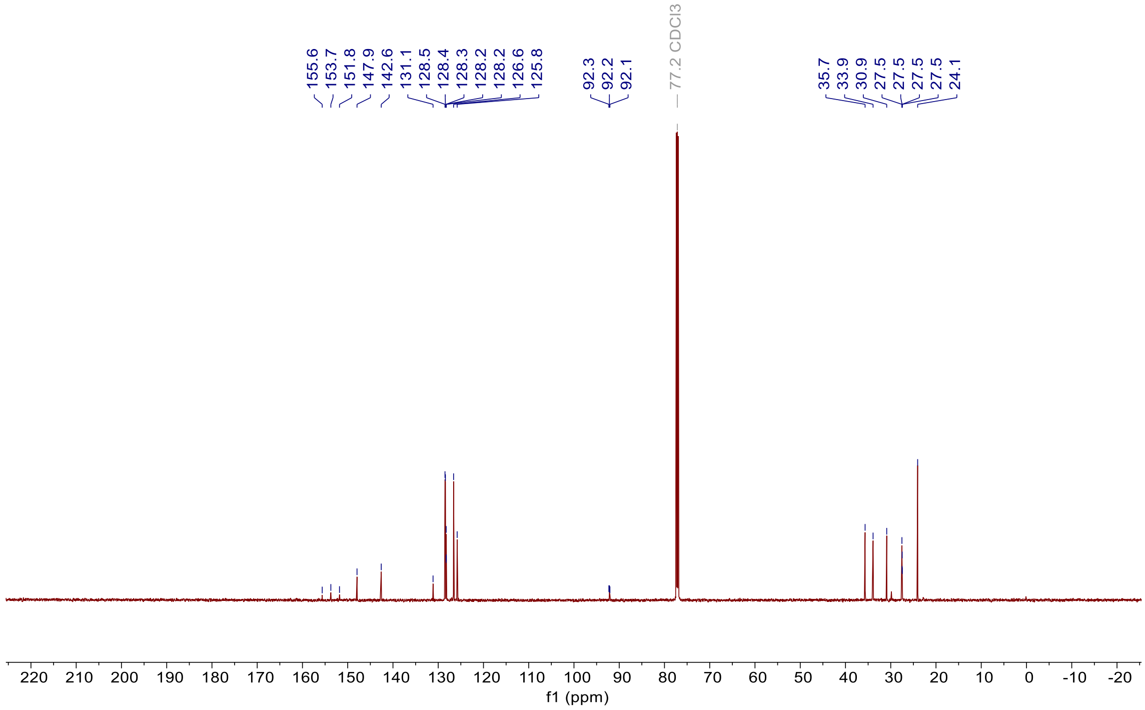

^{19}F NMR of 23 (376 MHz, $CDCl_3$)

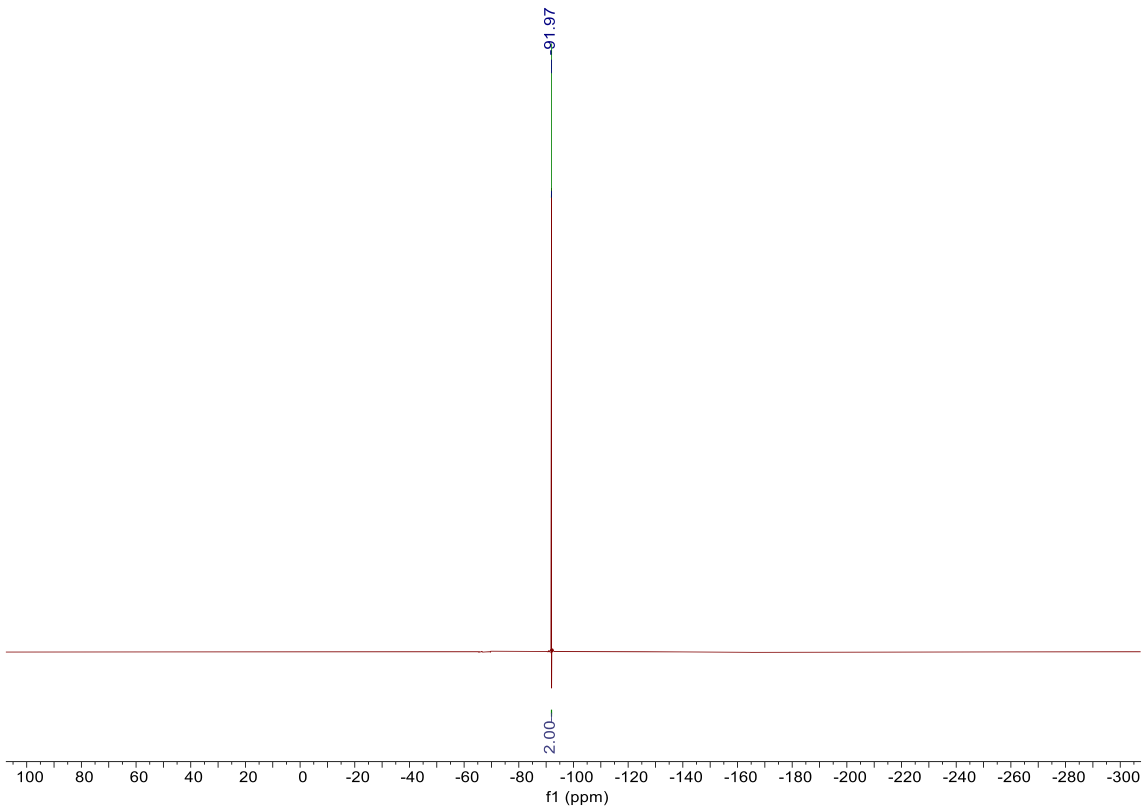

^{1}H NMR of 24 (600 MHz, $CDCl_3$)

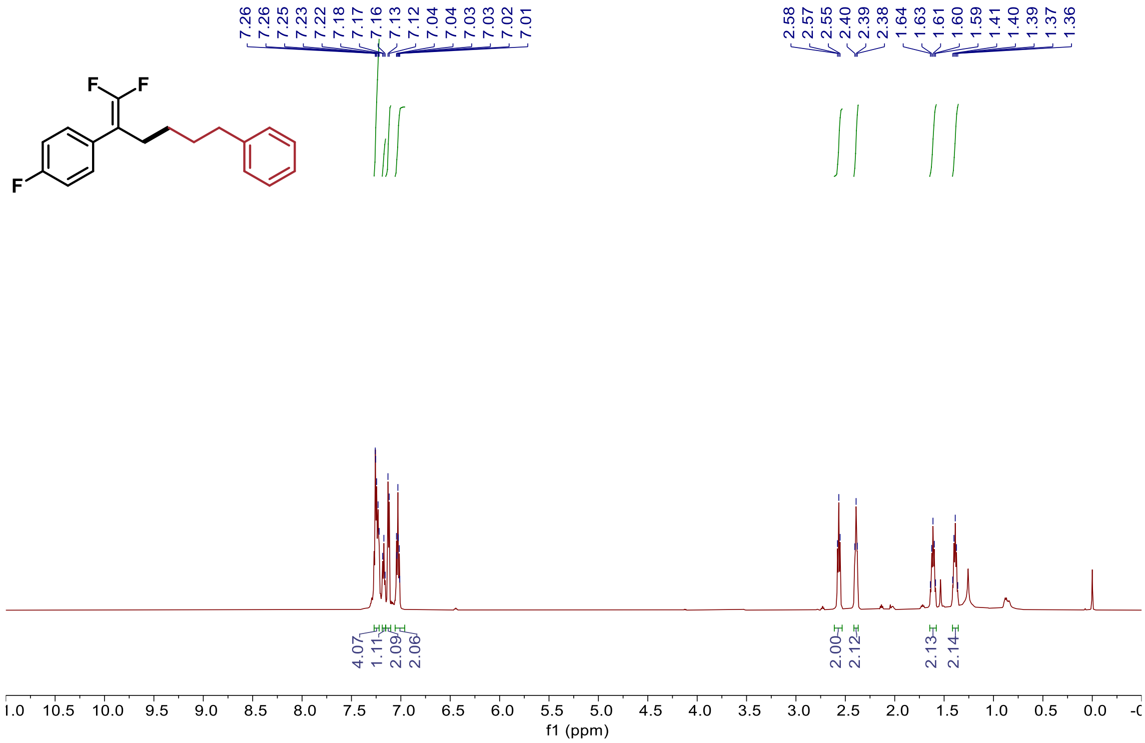

^{13}C NMR of 24 (600 MHz, $CDCl_3$)

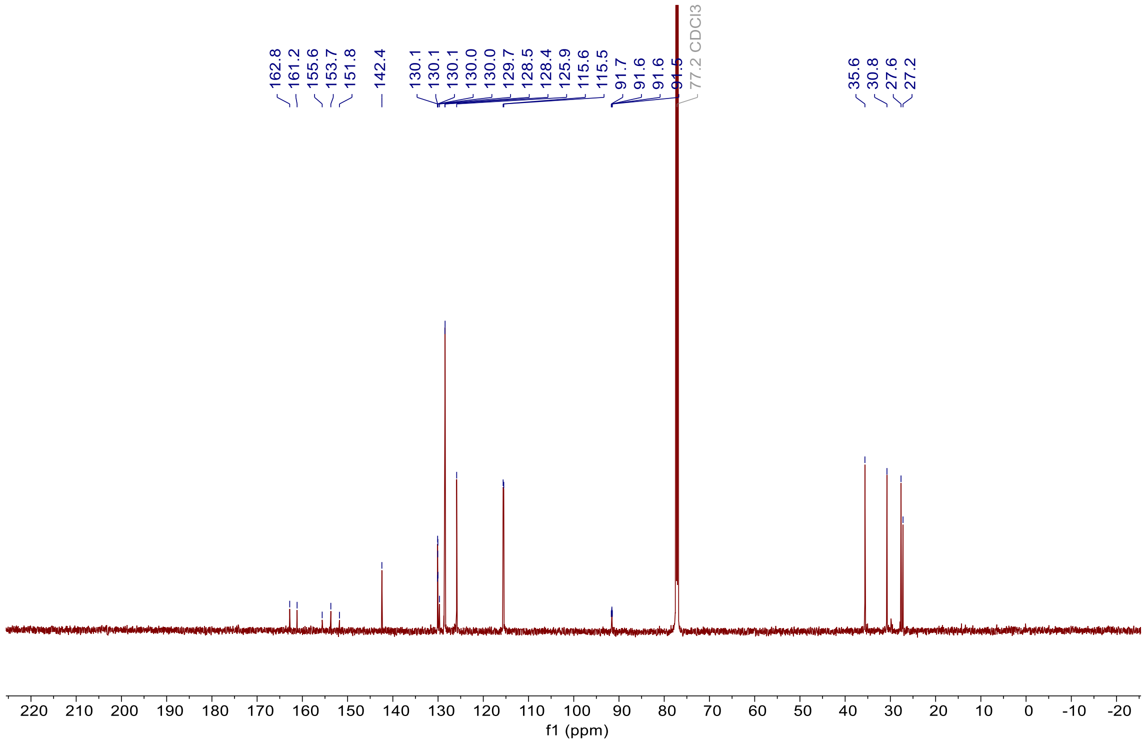

^{19}F NMR of 24 (565 MHz, $CDCl_3$)

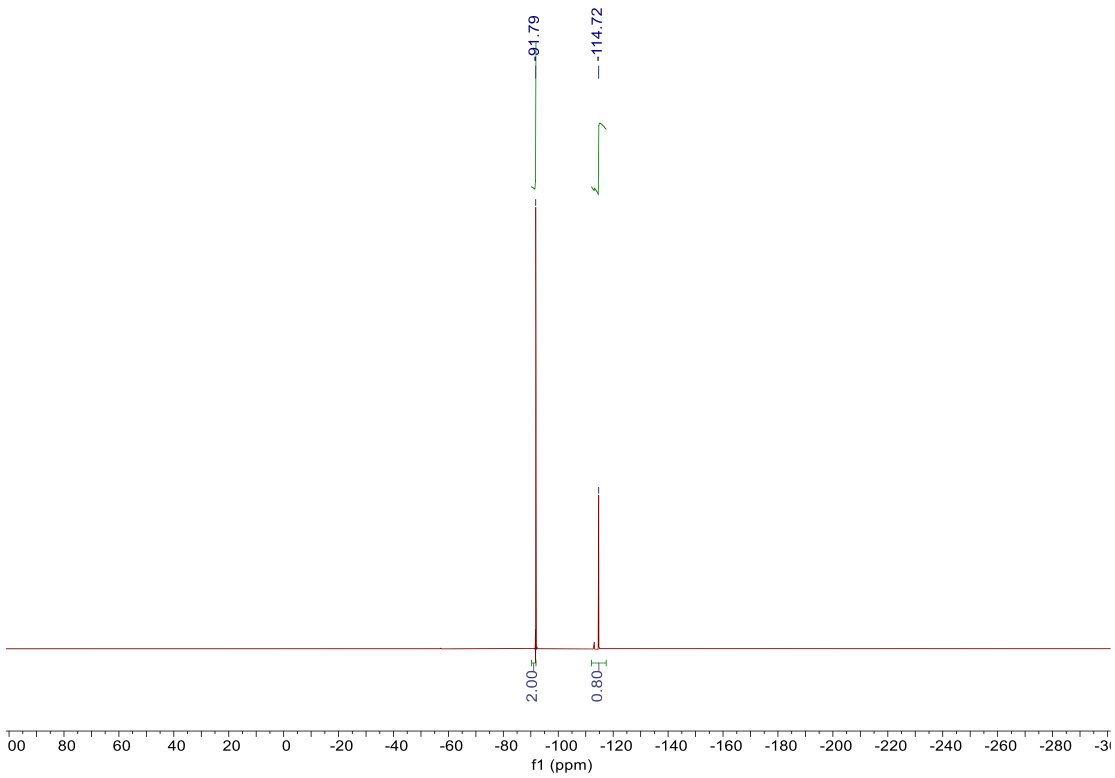

^{1}H NMR of 25 (600 MHz, $CDCl_3$)

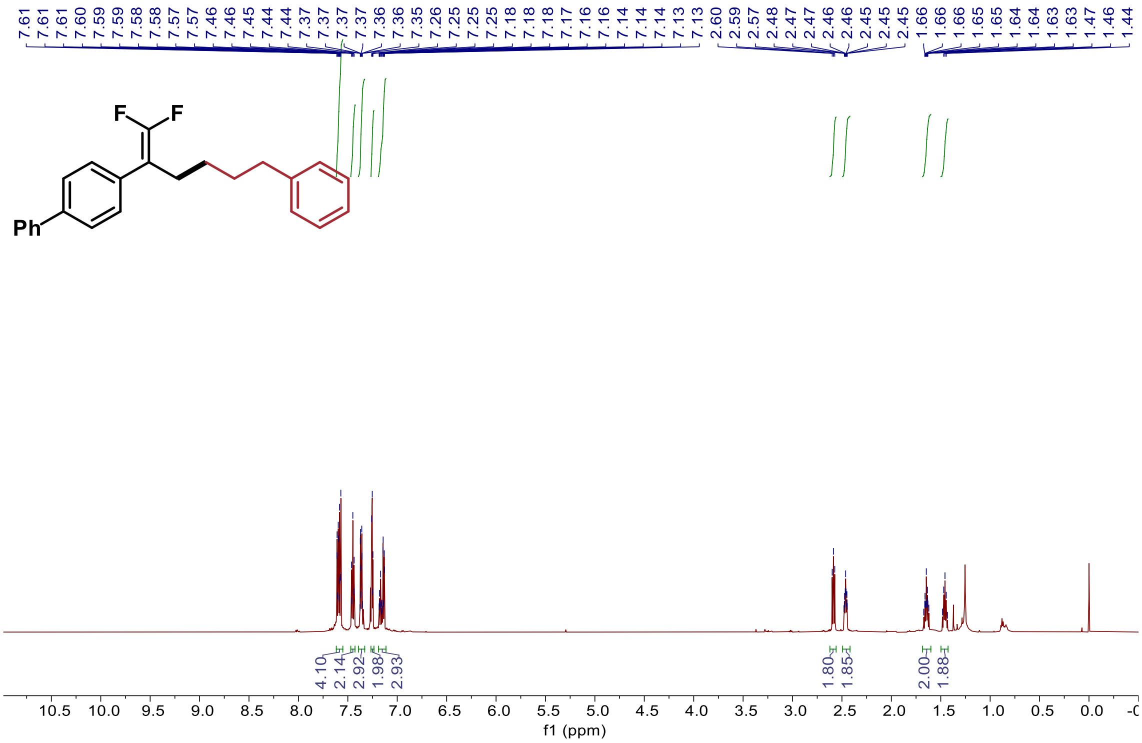

^{13}C NMR of 25 (151 MHz, $CDCl_3$)

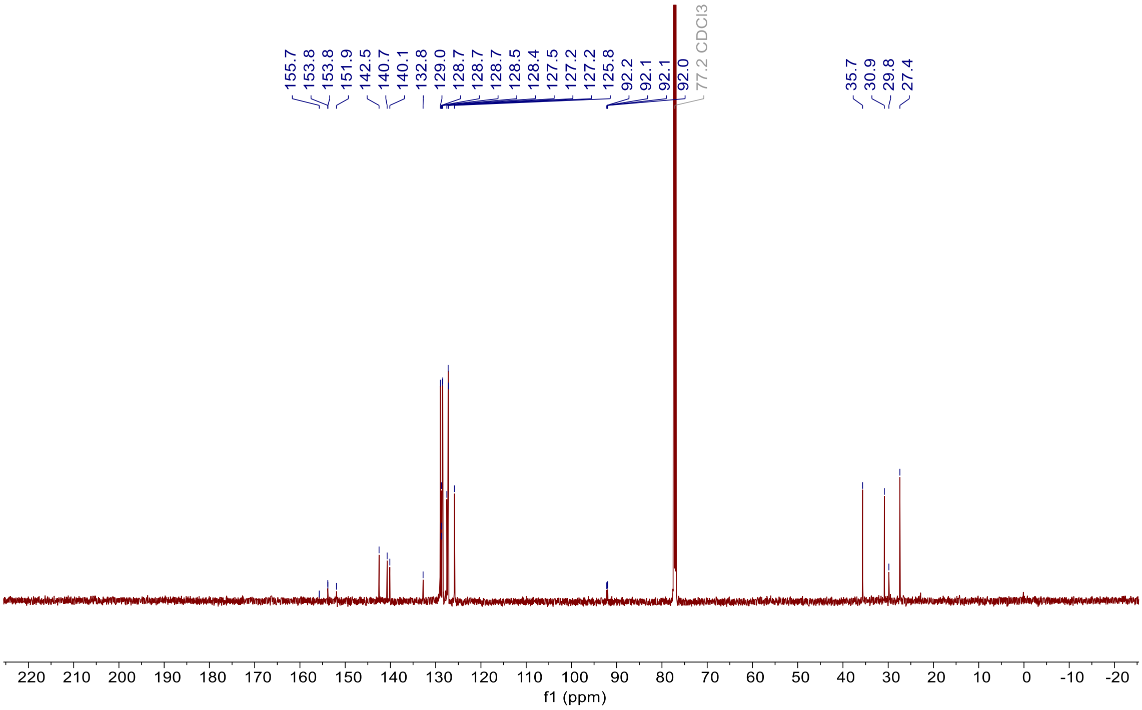

^{19}F NMR of 25 (565 MHz, CDCl$_3$)

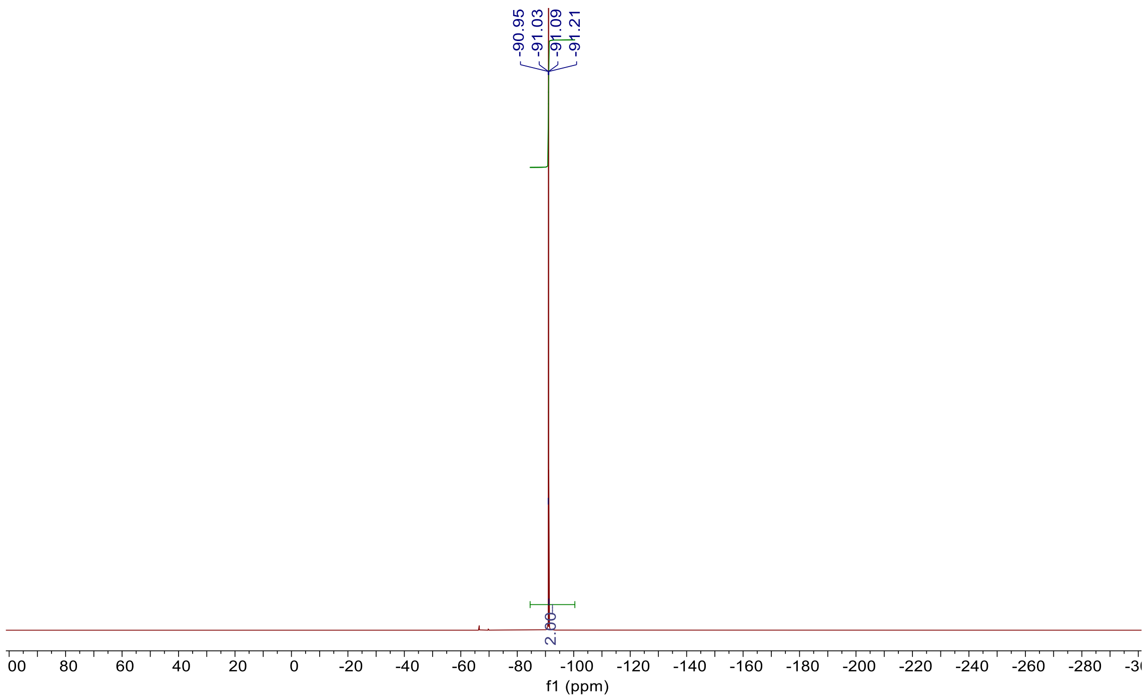

^{1}H NMR of 26 (500 MHz, CDCl$_3$)

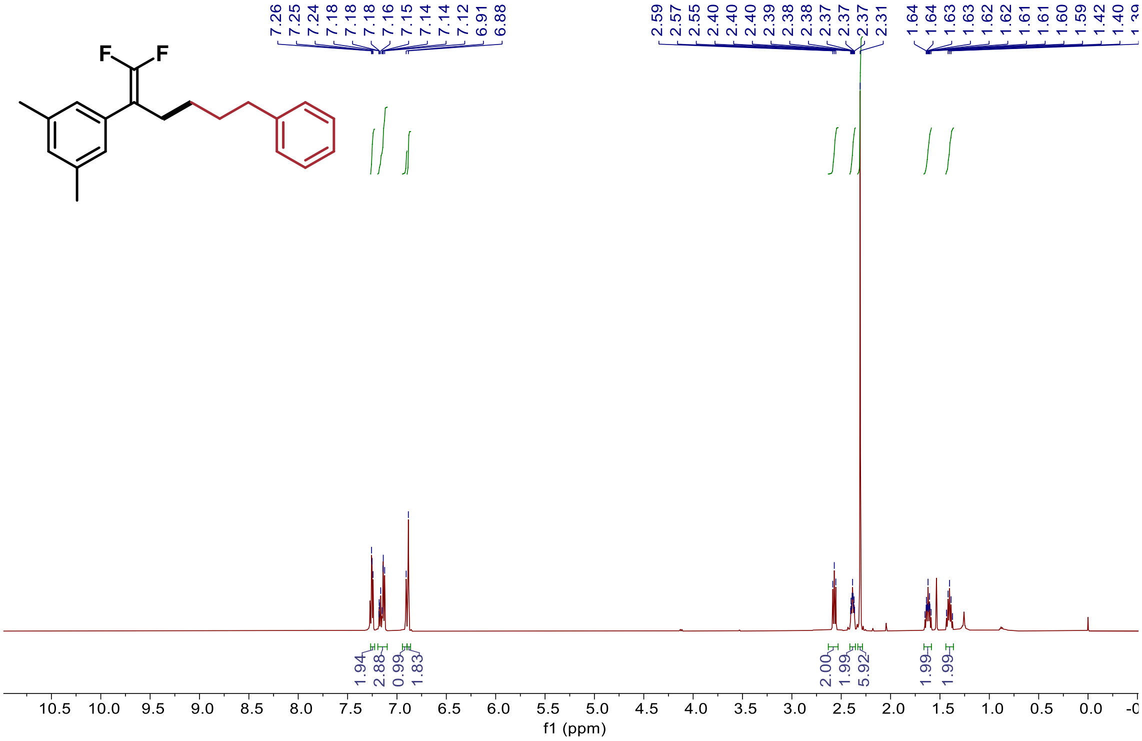

^{13}C NMR of 26 (126 MHz, CDCl$_3$)

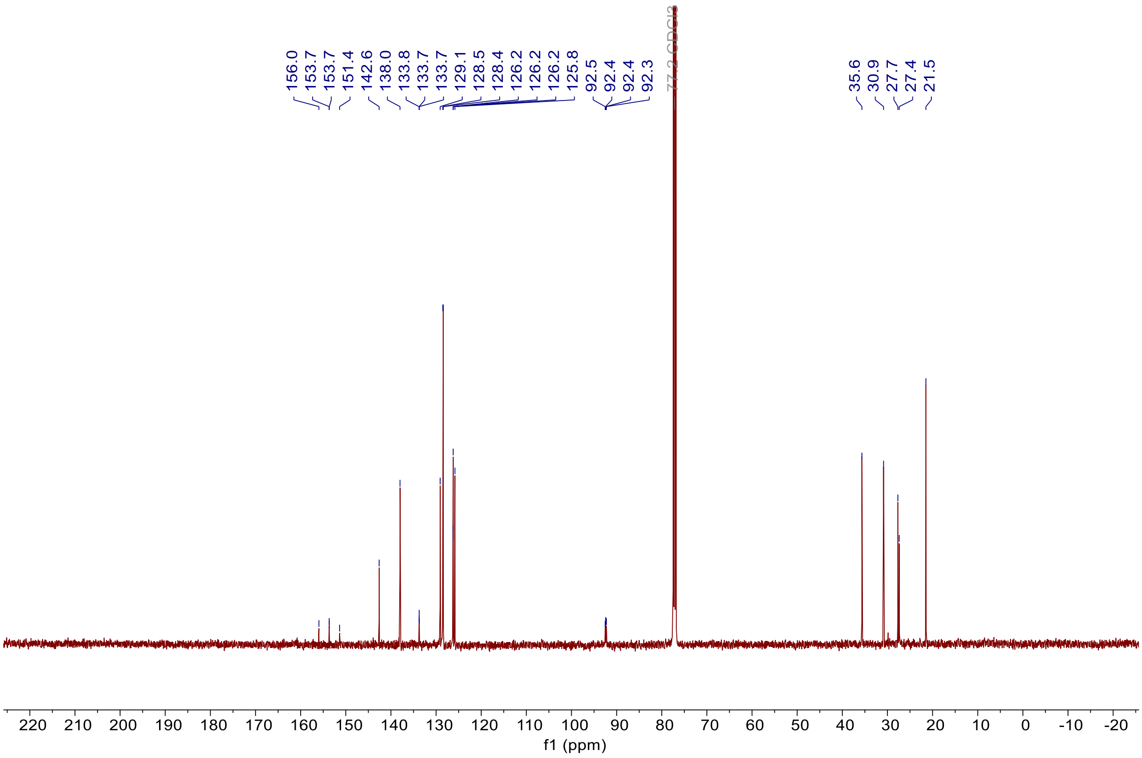

^{19}F NMR of 26 (471 MHz, CDCl$_3$)

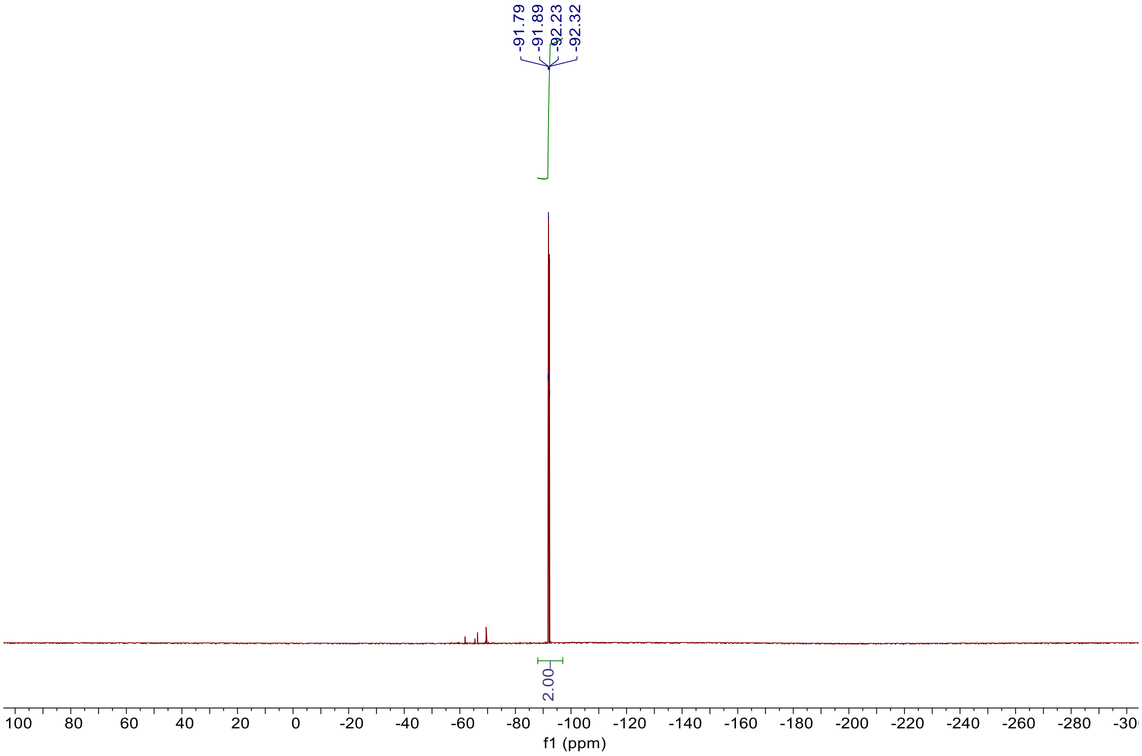

^{1}H NMR of 27 (400 MHz, CDCl$_3$)

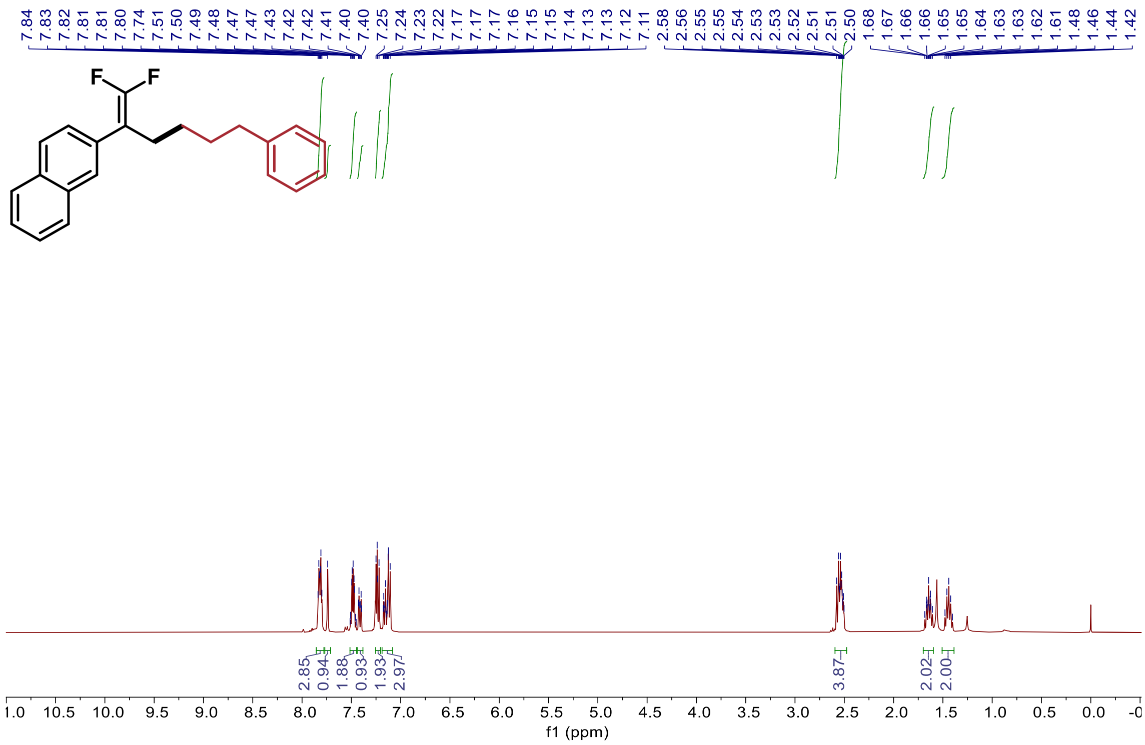

^{13}C NMR of 27 (151 MHz, CDCl$_3$)

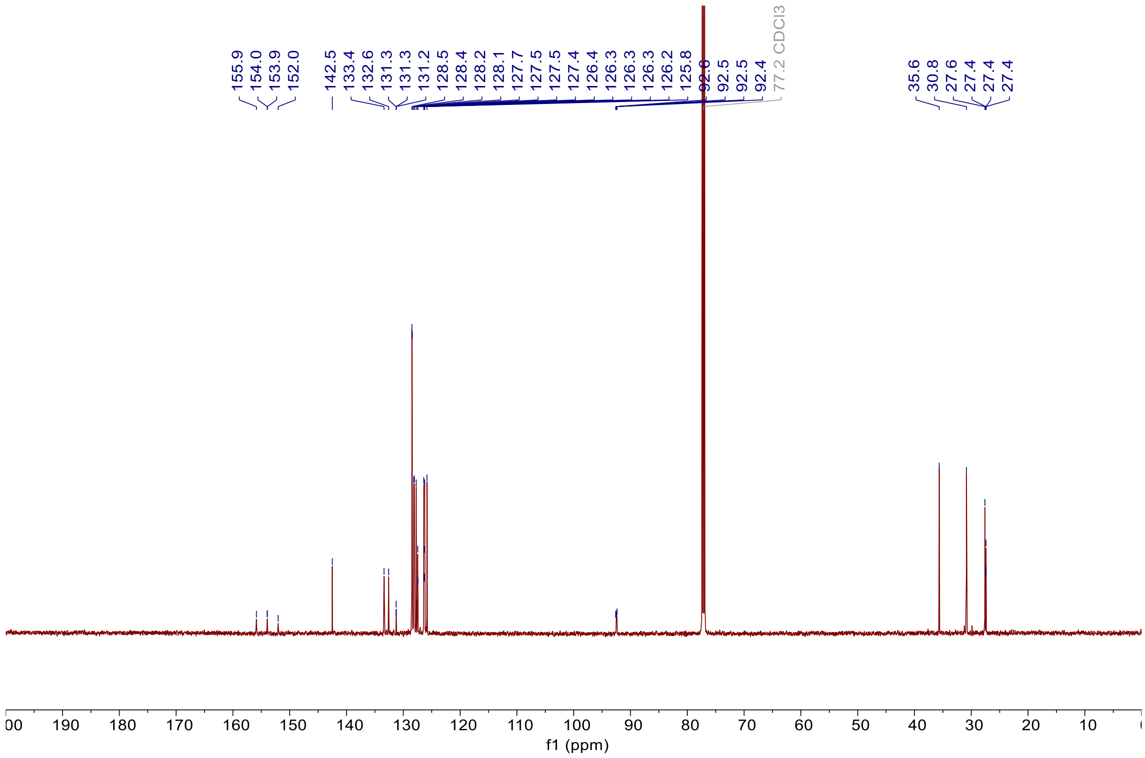

^{19}F NMR of 27 (376 MHz, $CDCl_3$)

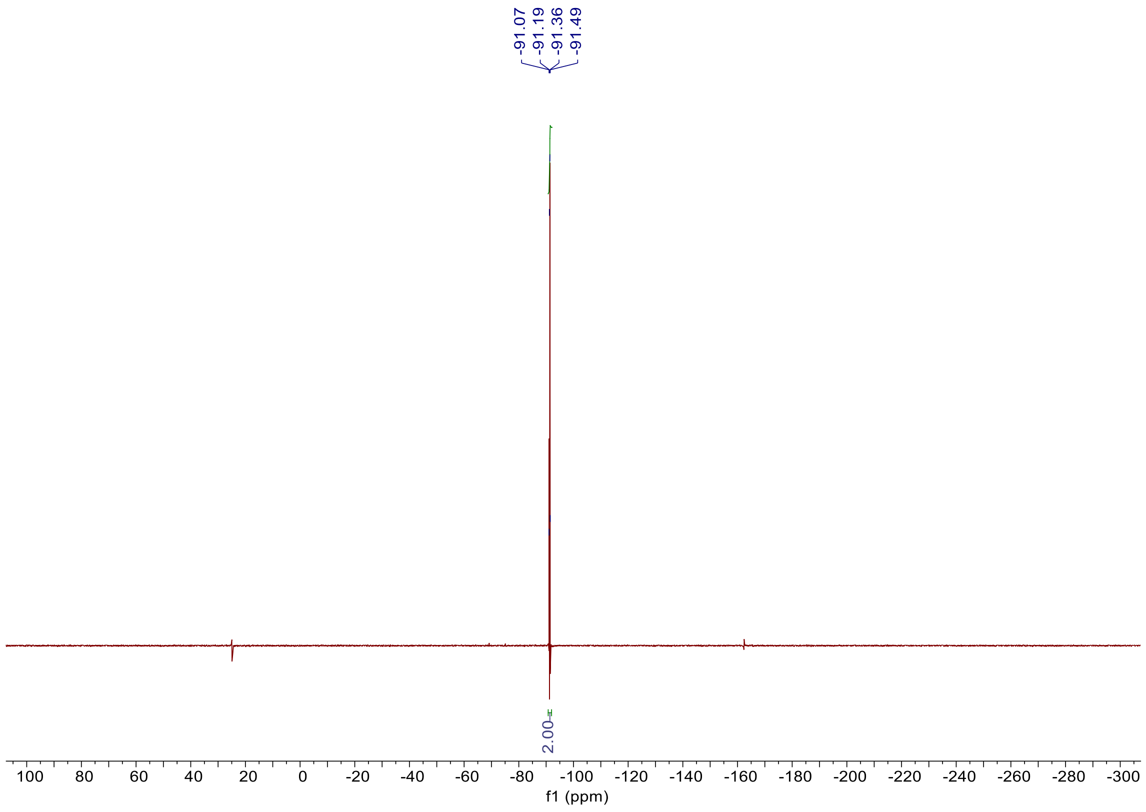

^{1}H NMR of 28 (600 MHz, $CDCl_3$)

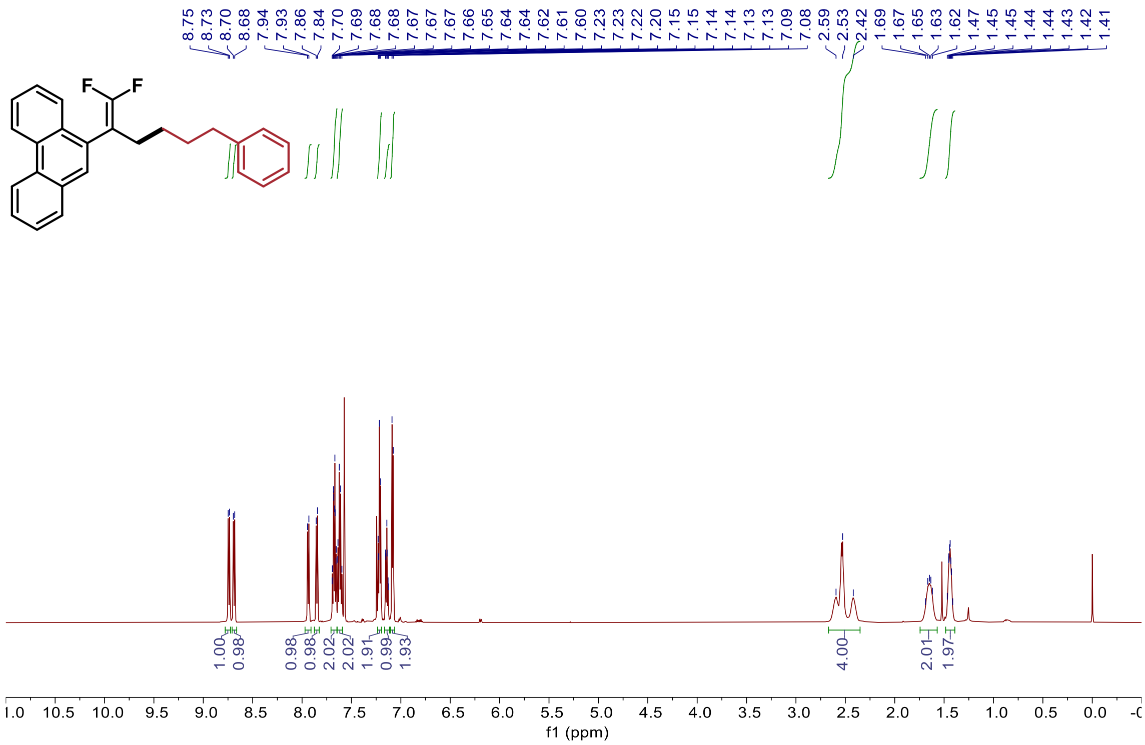

^{13}C NMR of 28 (151 MHz, $CDCl_3$)

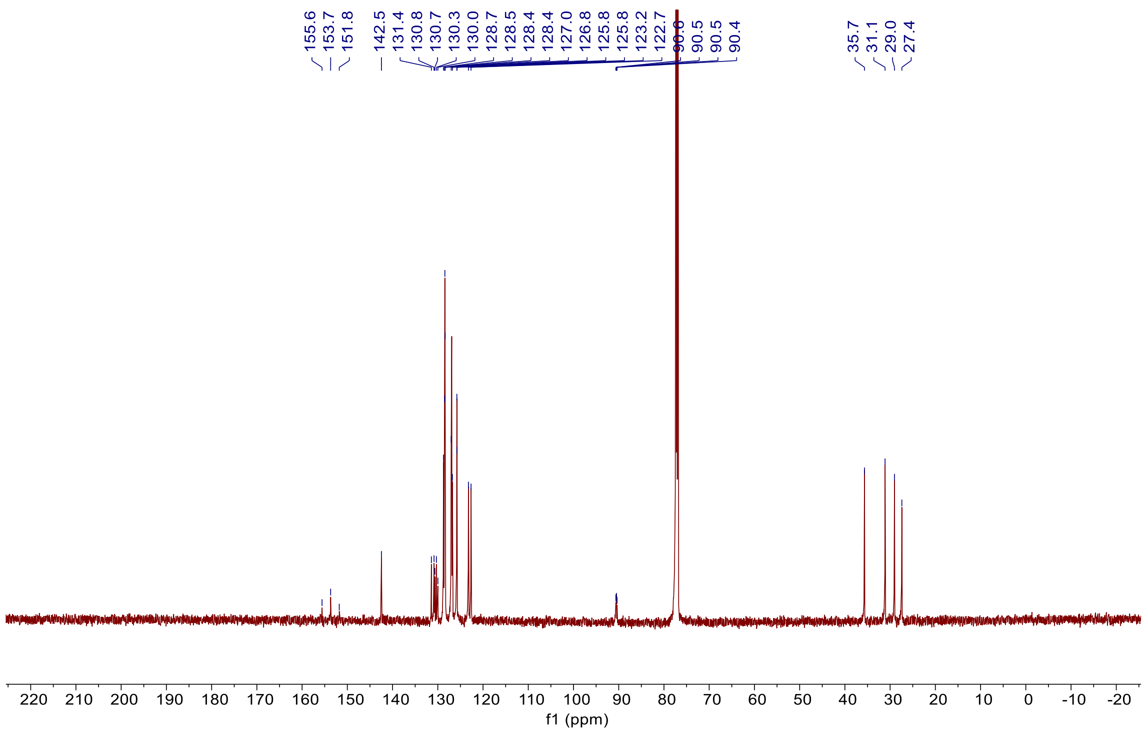

^{19}F NMR of 28 (565 MHz, $CDCl_3$)

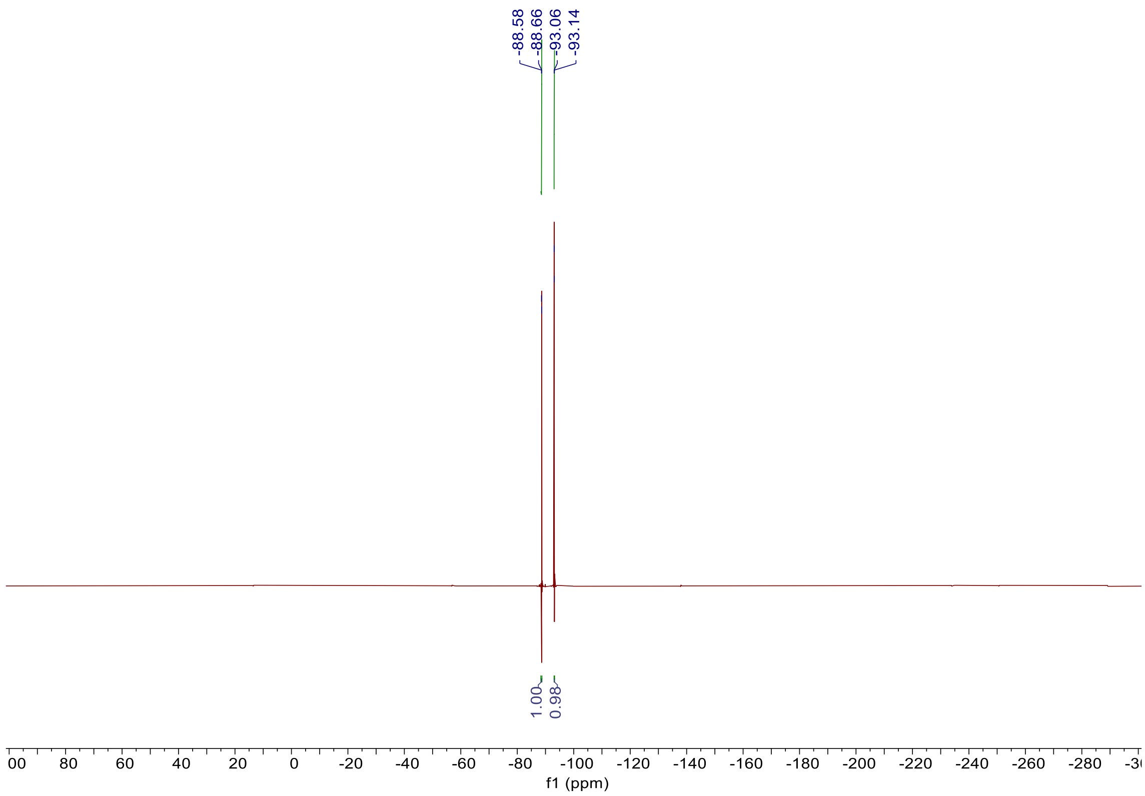

^{1}H NMR of 29 (400 MHz, $CDCl_3$)

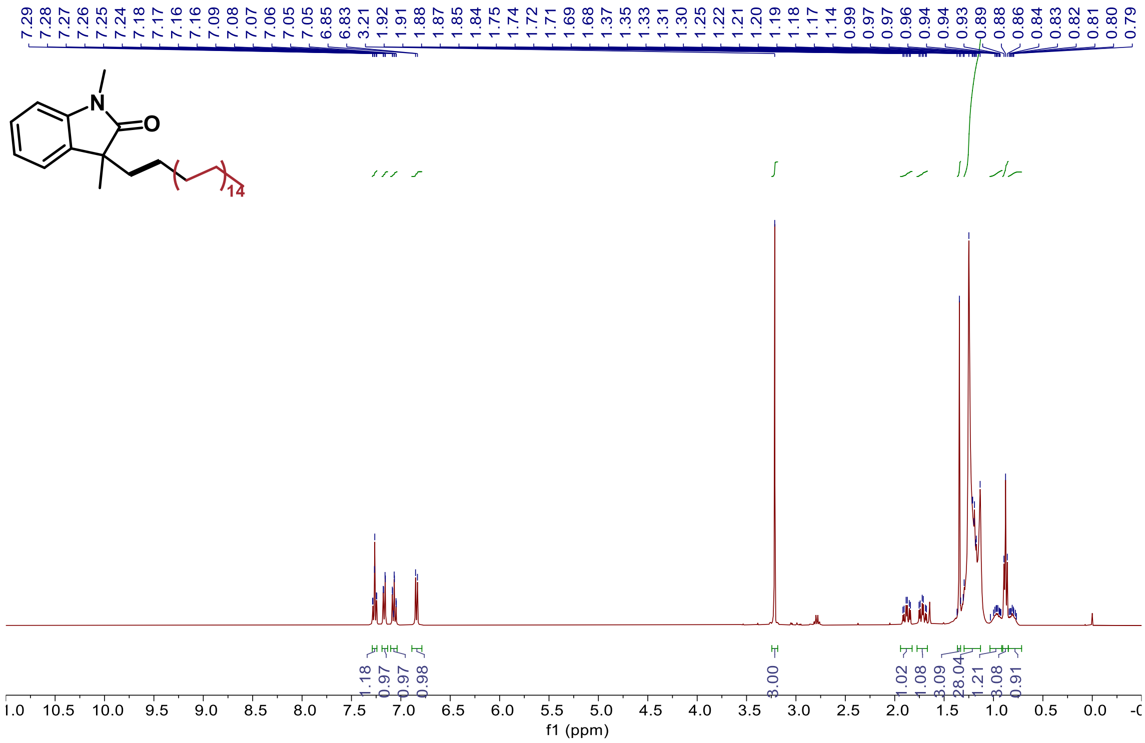

^{13}C NMR of 29 (101 MHz, $CDCl_3$)

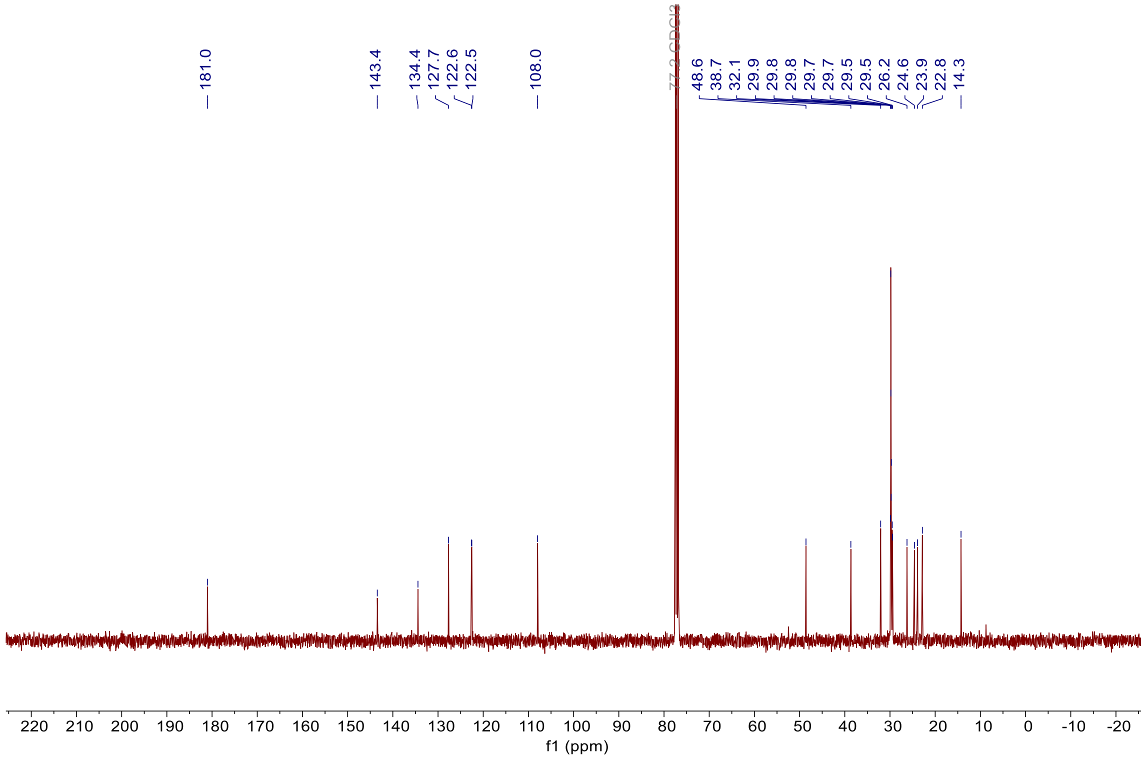

^{1}H NMR of 30 (600 MHz, $CDCl_3$)

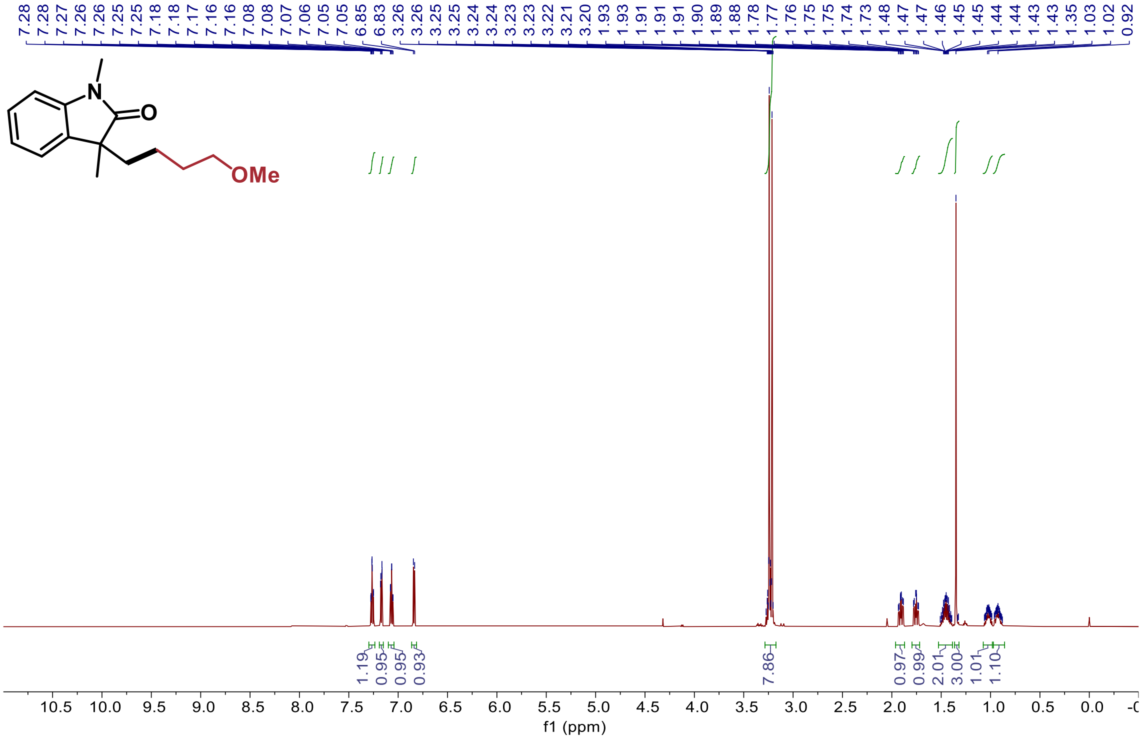

^{13}C NMR of 30 (151 MHz, $CDCl_3$)

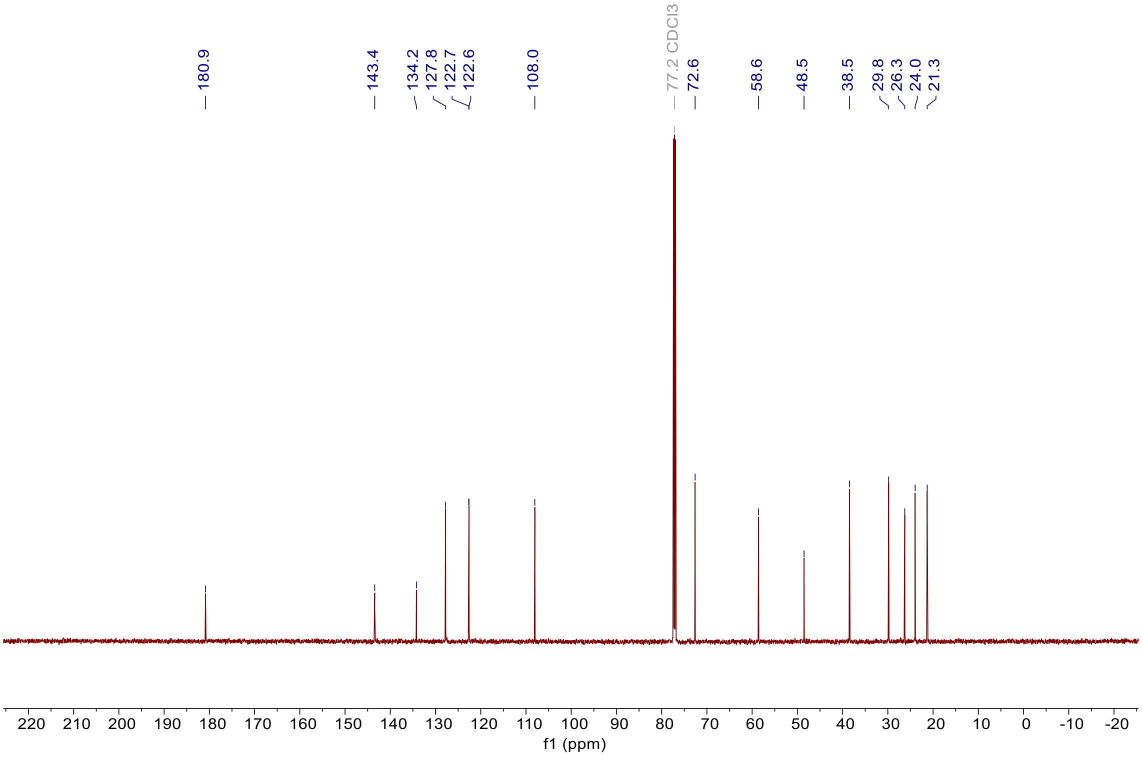

^{1}H NMR of 31 (500 MHz, CDCl3)

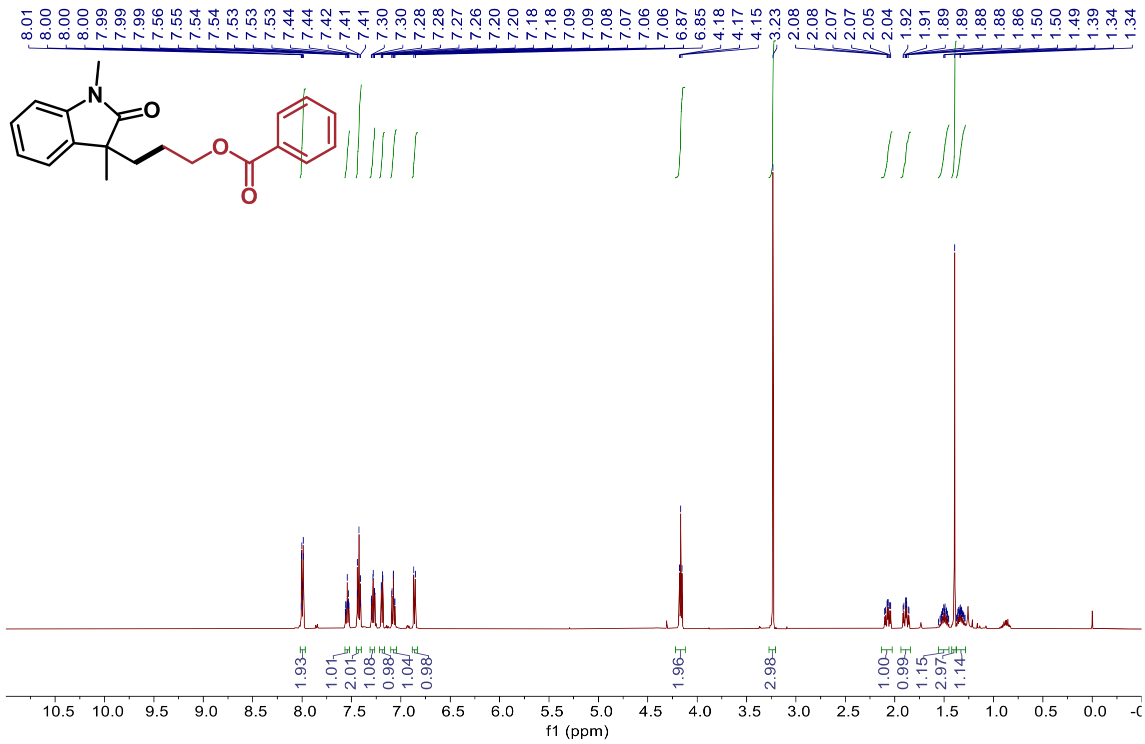

^{13}C NMR of 31 (126 MHz, CDCl3)

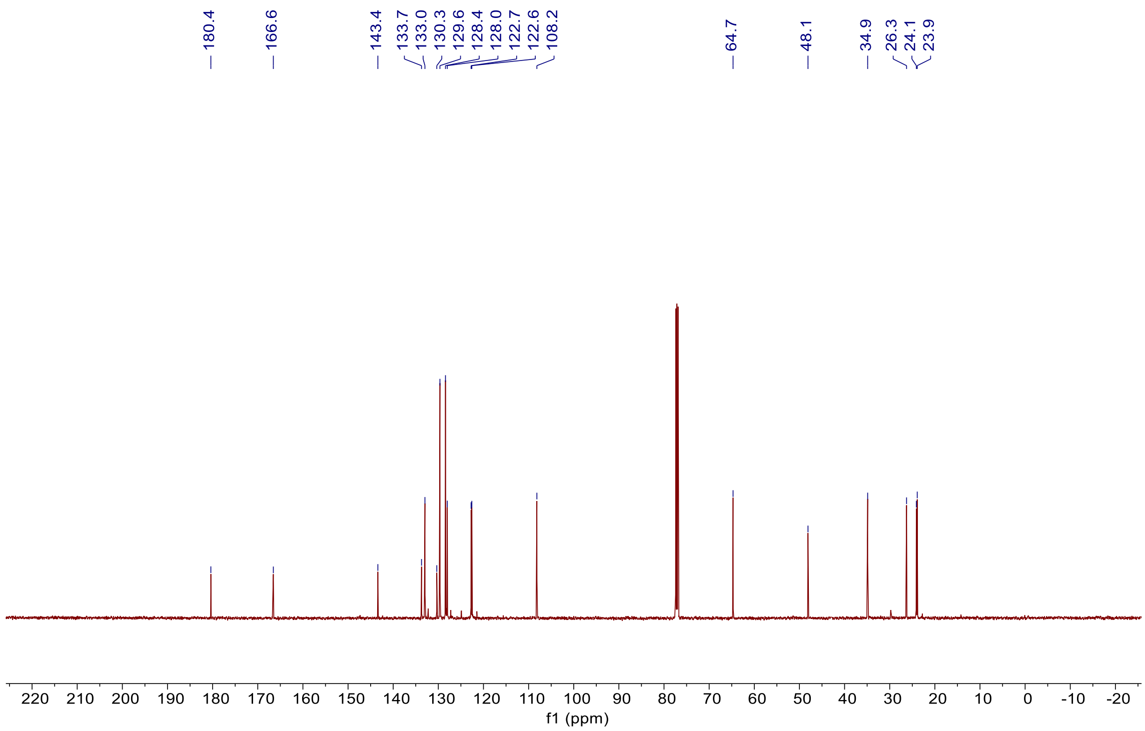

^{1}H NMR of 32 (600 MHz, $CDCl_3$)

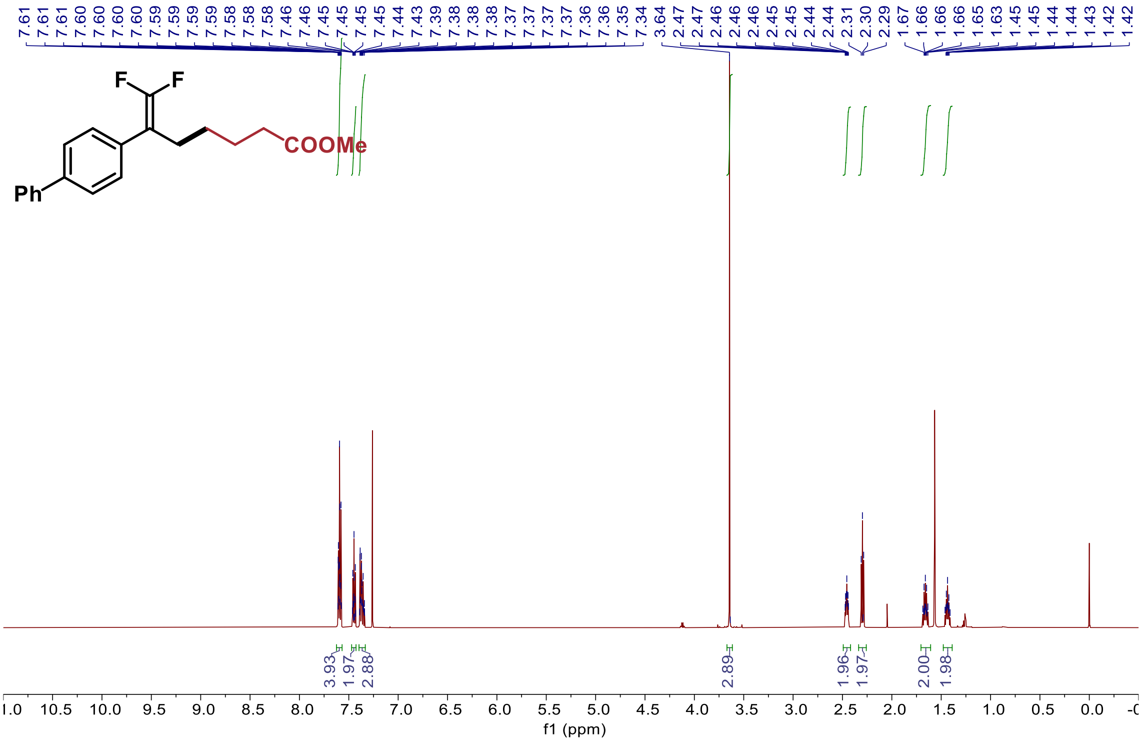

^{13}C NMR of 32 (151 MHz, $CDCl_3$)

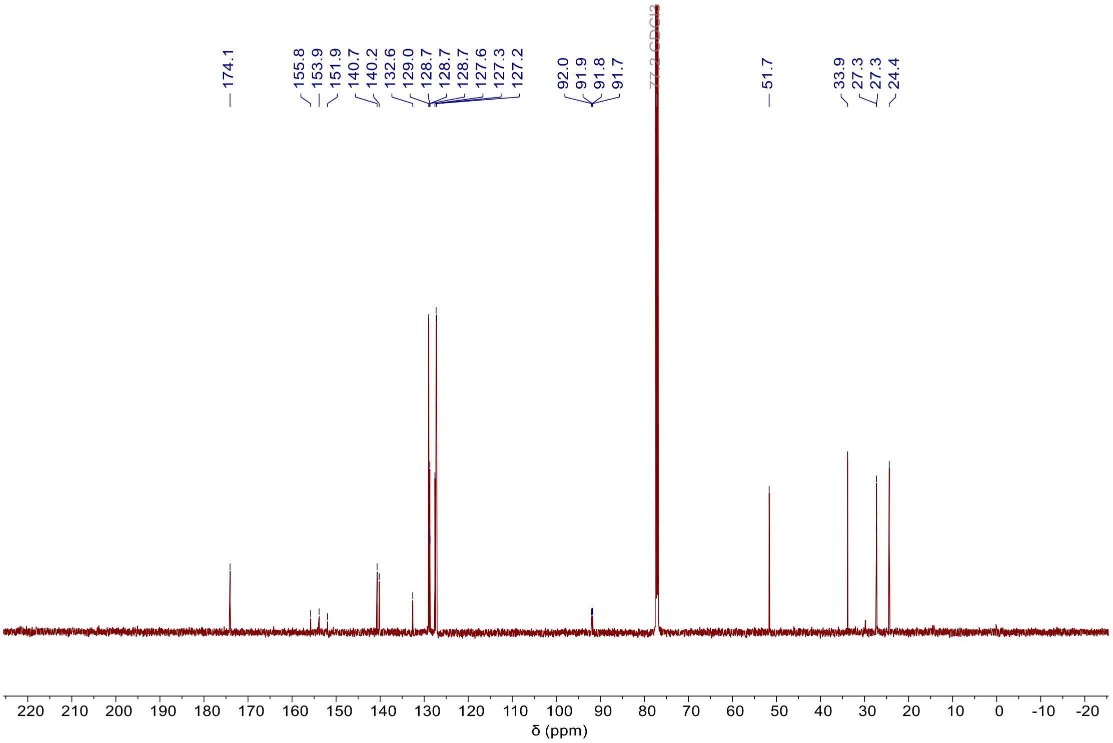

^{19}F NMR of 32 (565 MHz, $CDCl_3$)

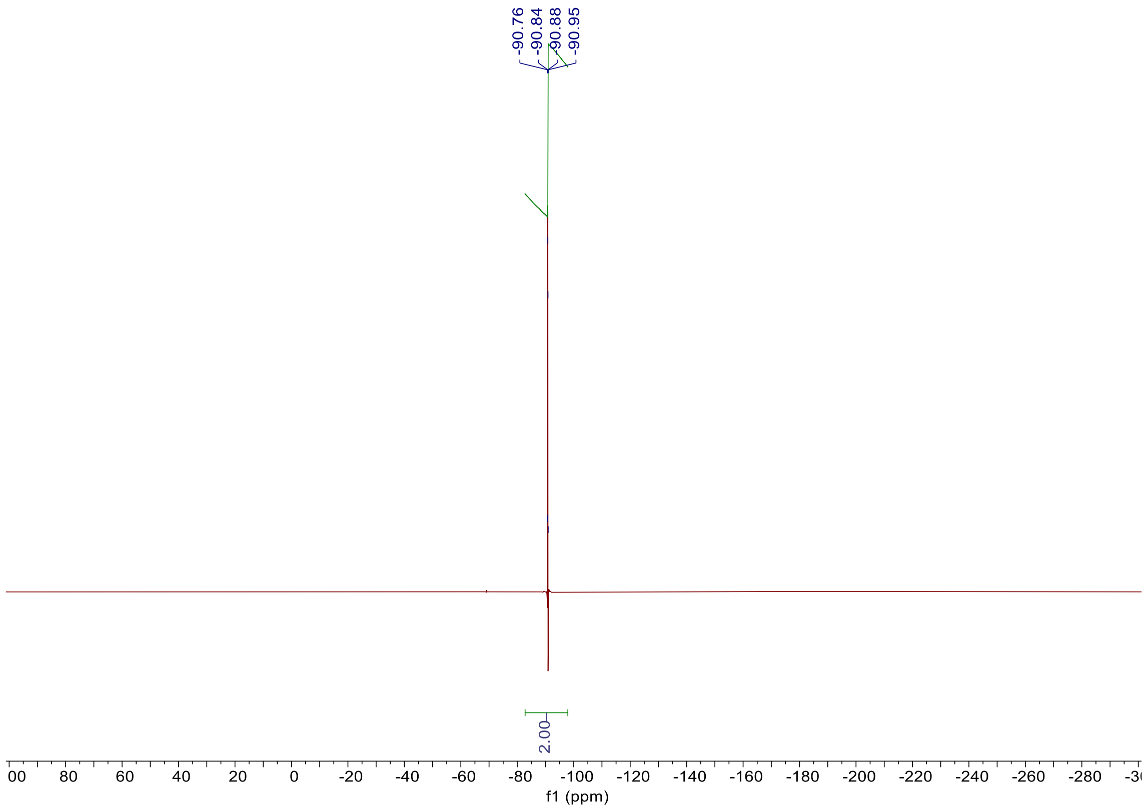

^{1}H NMR of 33 (500 MHz, CDCl$_3$)

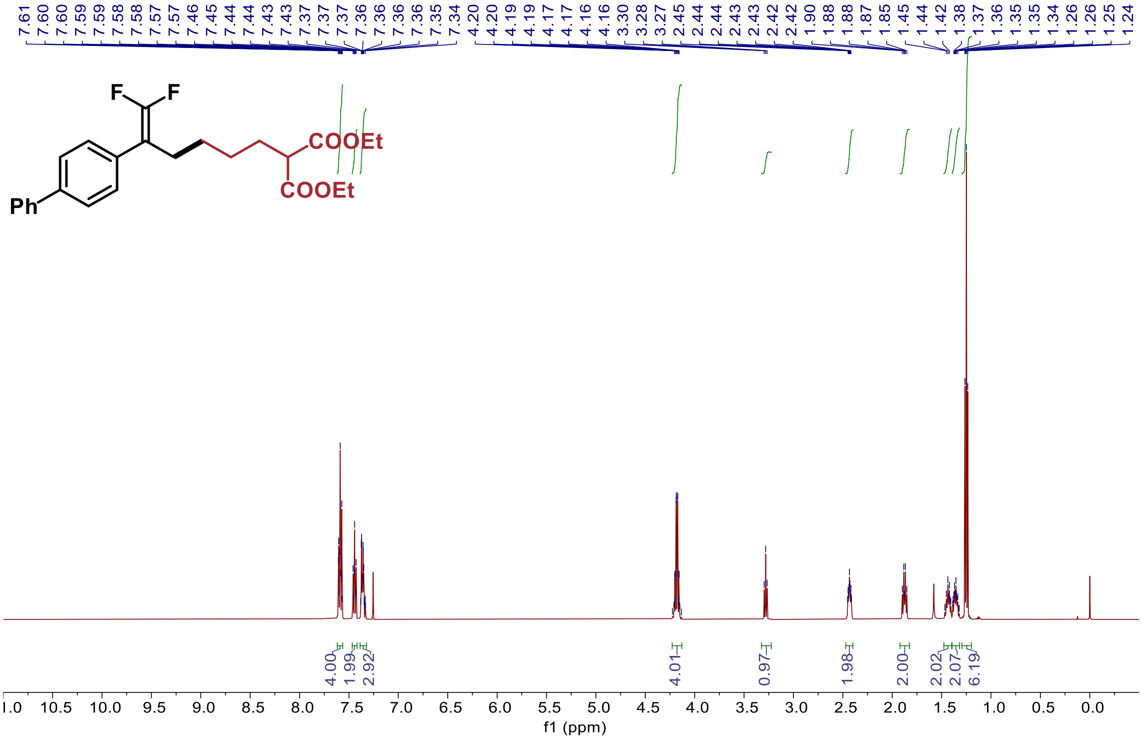

^{13}C NMR of 33 (126 MHz, CDCl$_3$)

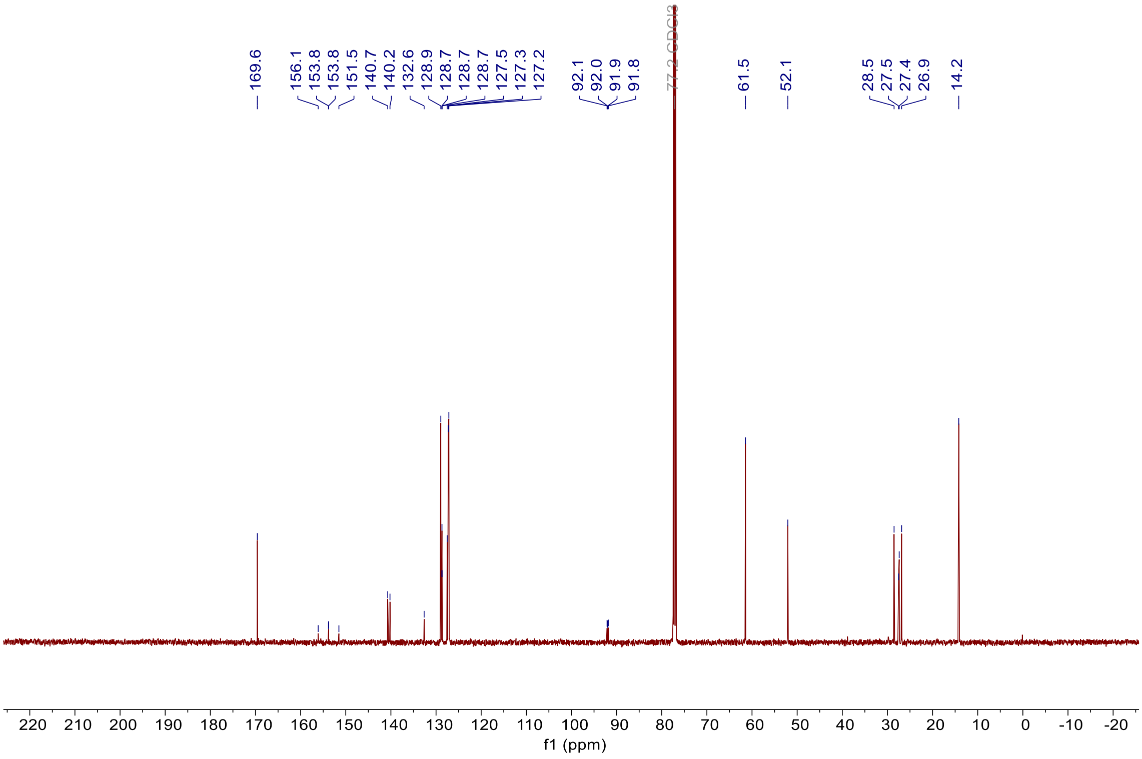

^{19}F NMR of 33 (471 MHz, $CDCl_3$)

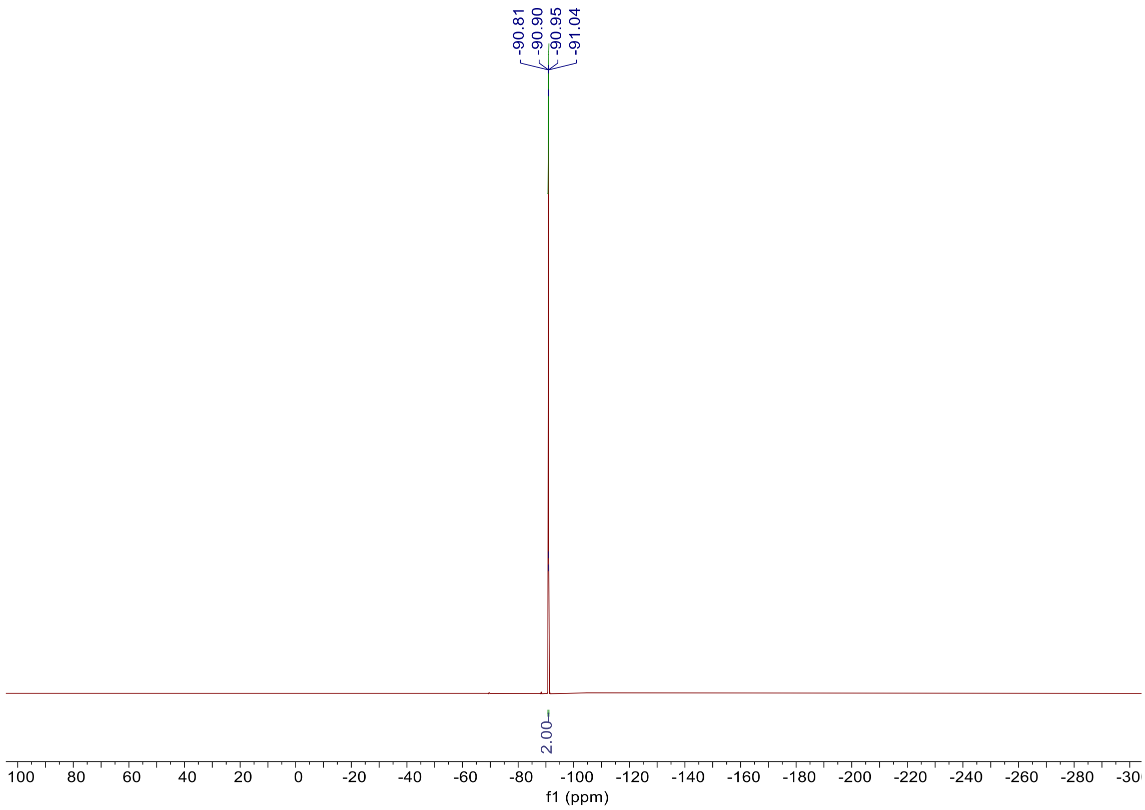

^{1}H NMR of 34 (600 MHz, CDCl3)

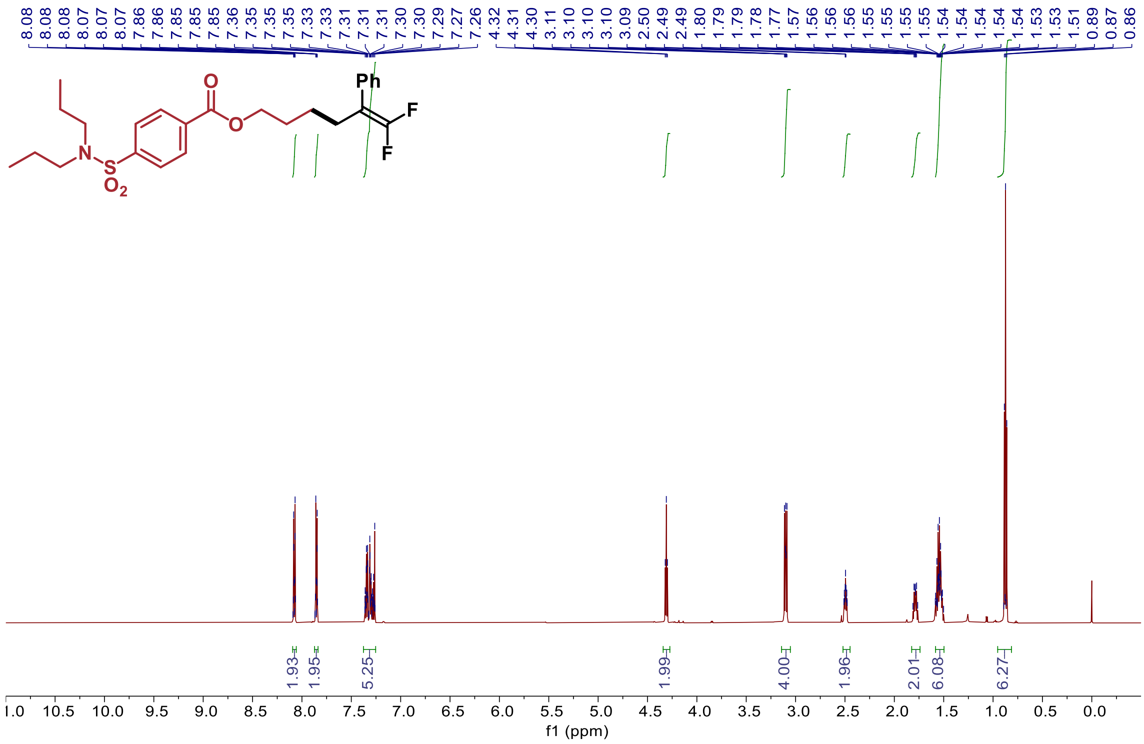

^{13}C NMR of 34 (151 Hz, CDCl3)

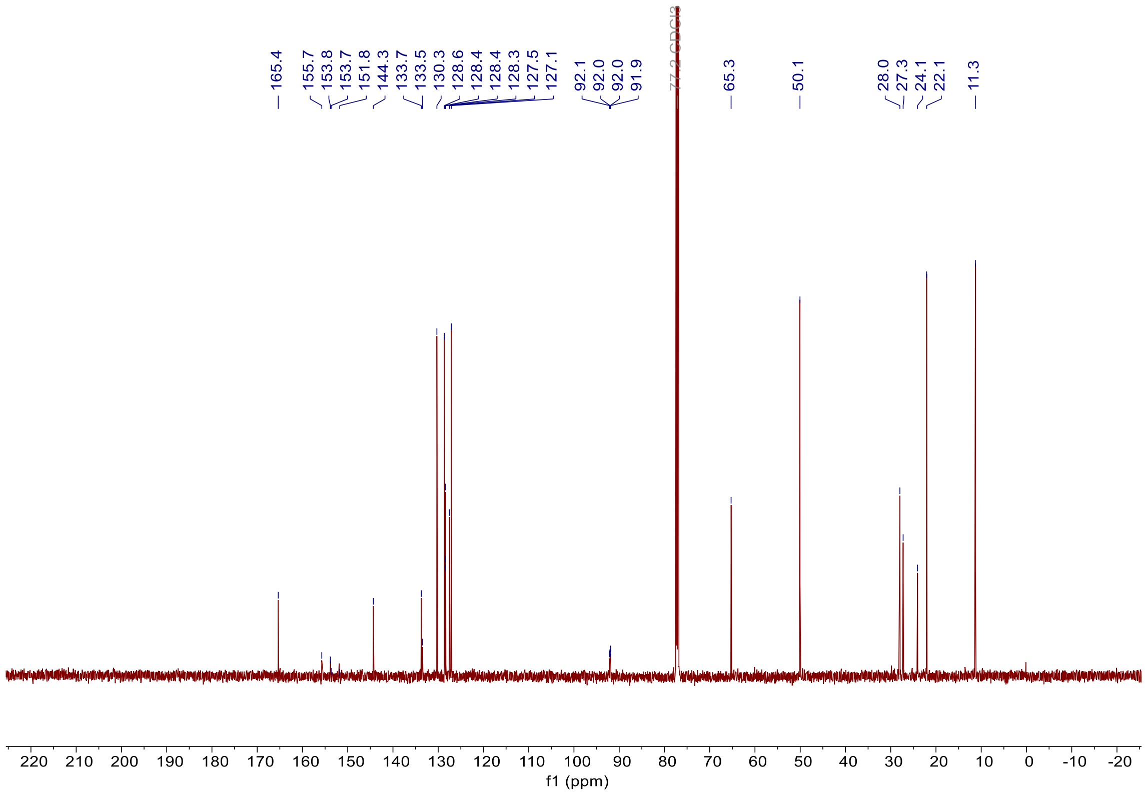

^{19}F NMR of 34 (565 MHz, $CDCl_3$)

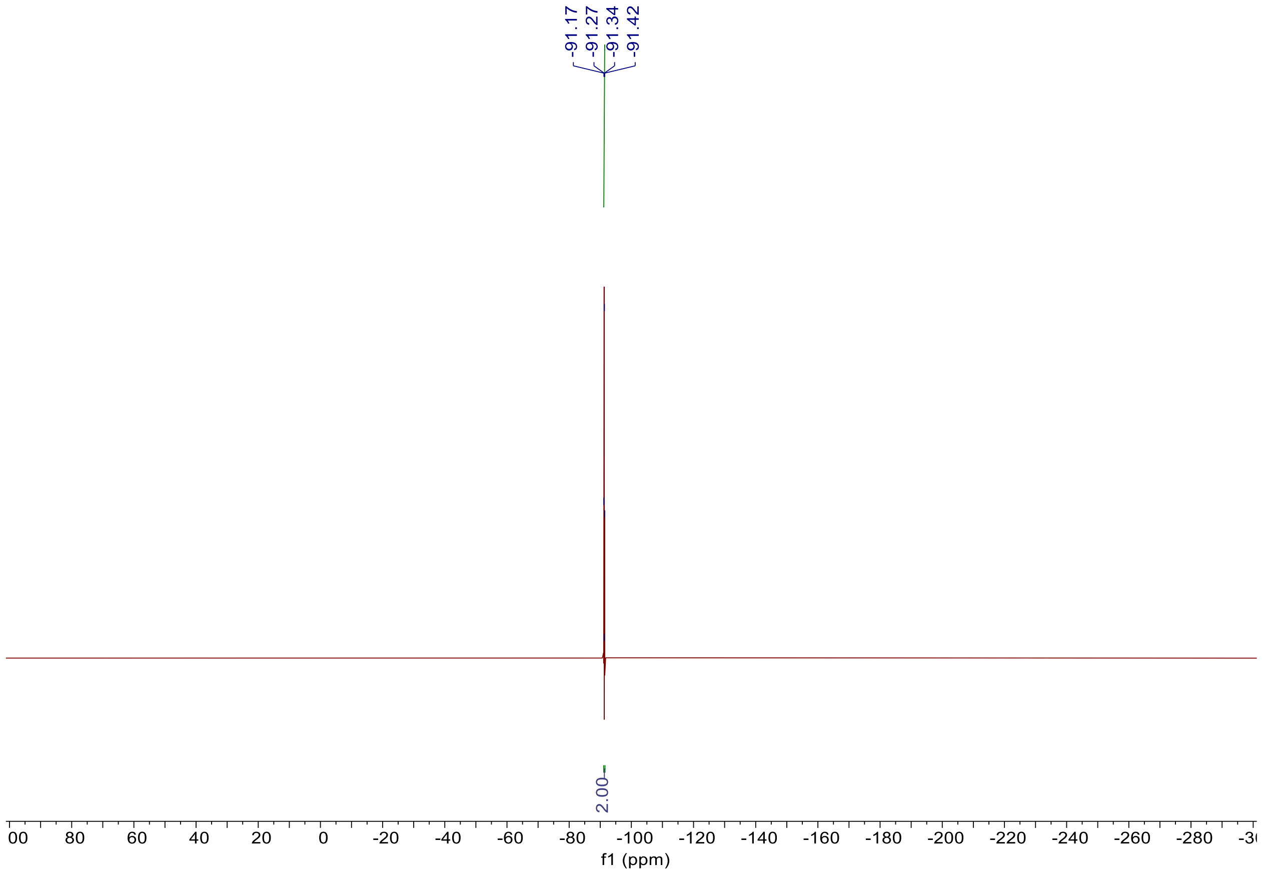

^{1}H NMR of 35 (600 MHz, CDCl$_3$)

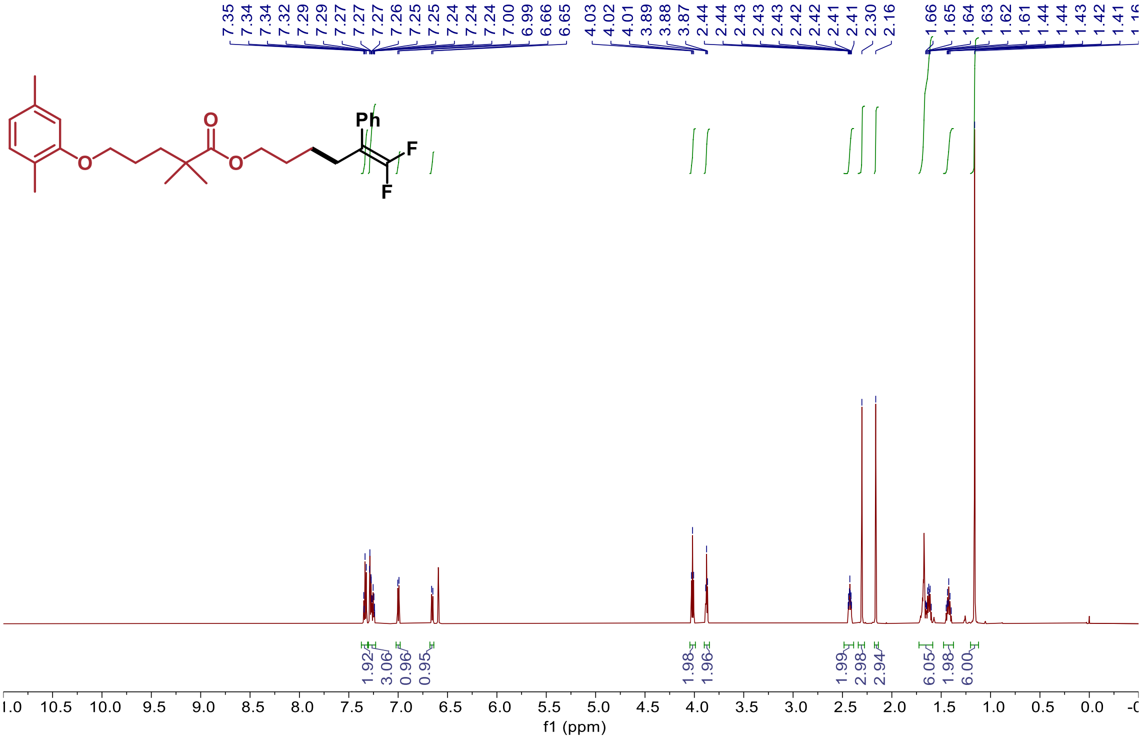

^{13}C NMR of 35 (151 Hz, CDCl$_3$)

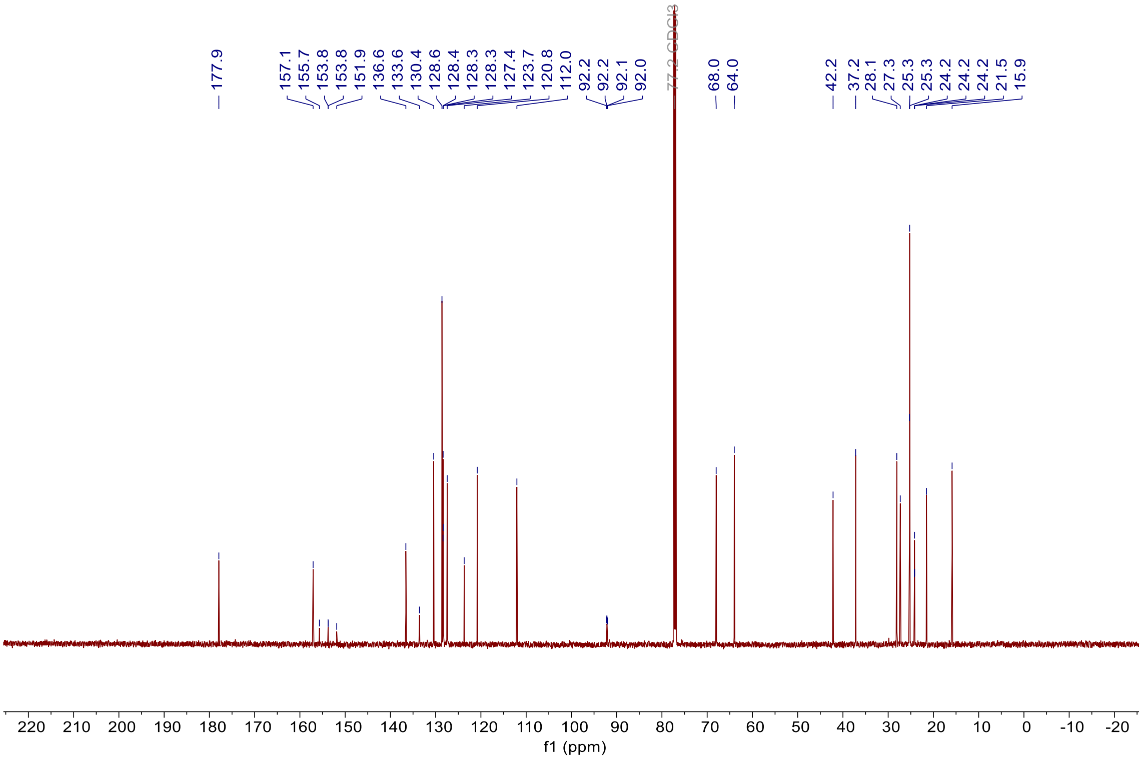

^{19}F NMR of 35 (565 Hz, CDCl$_3$)

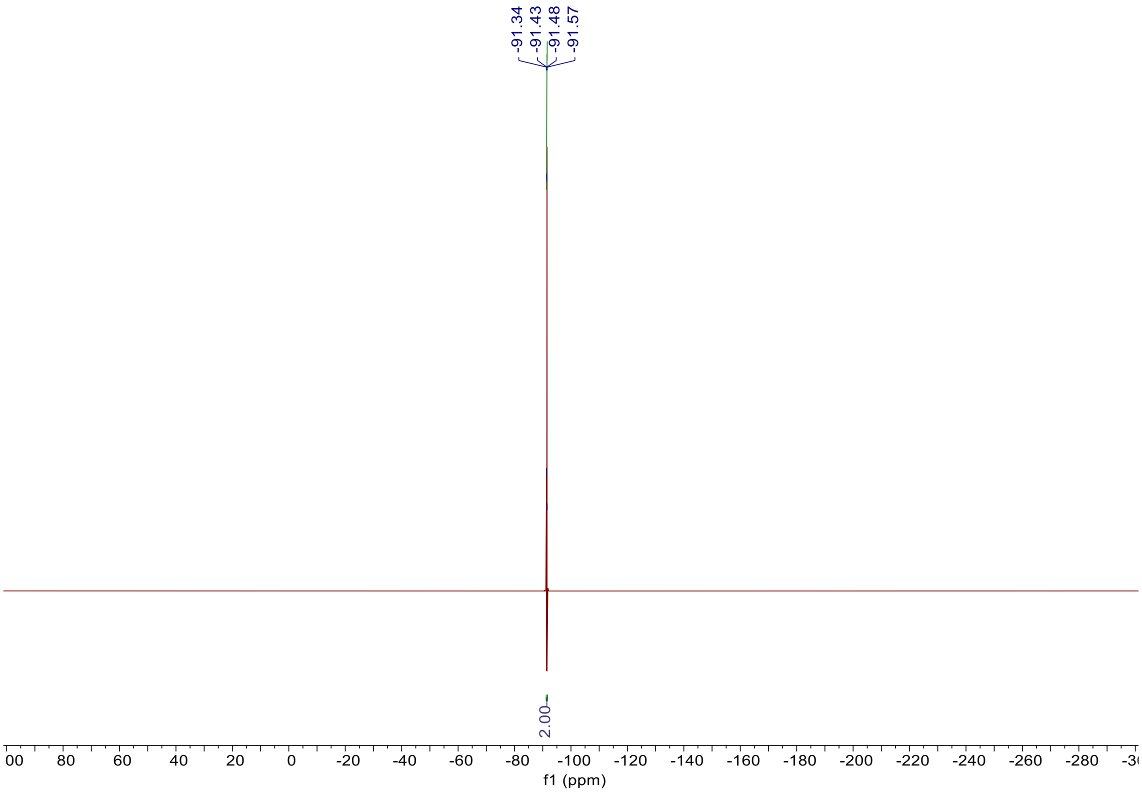

¹H NMR of 36 (400 MHz, CDCl₃)

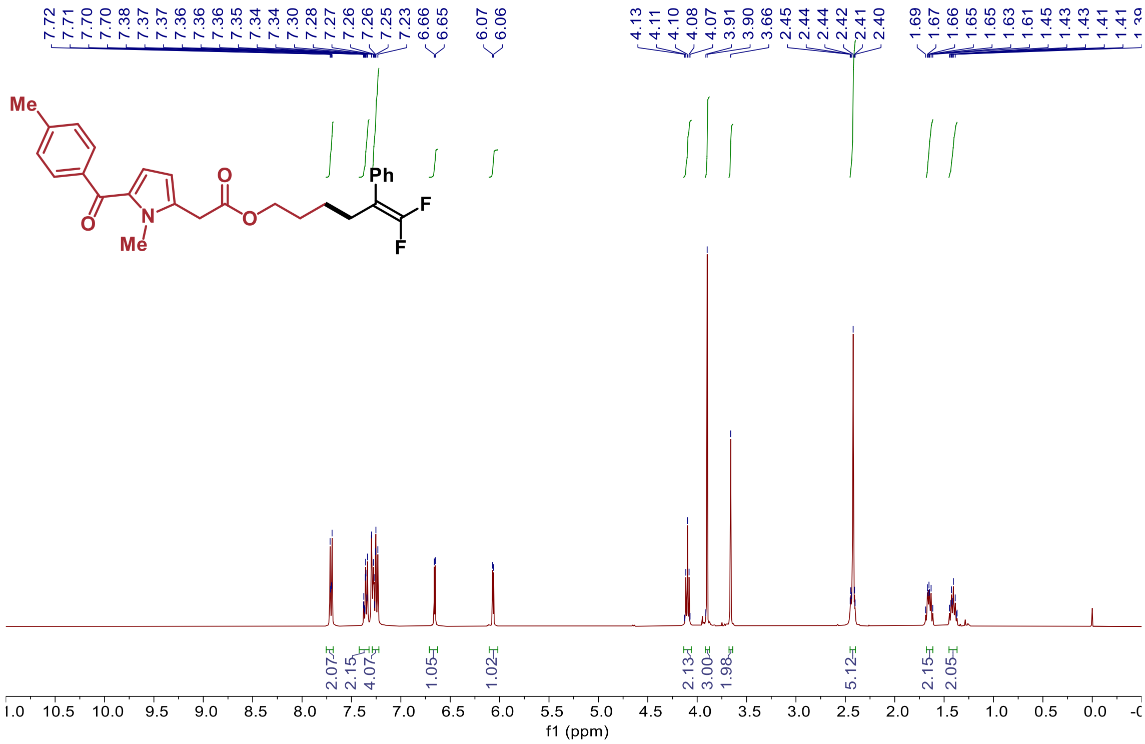

¹³C NMR of 36 (101 Hz, CDCl₃)

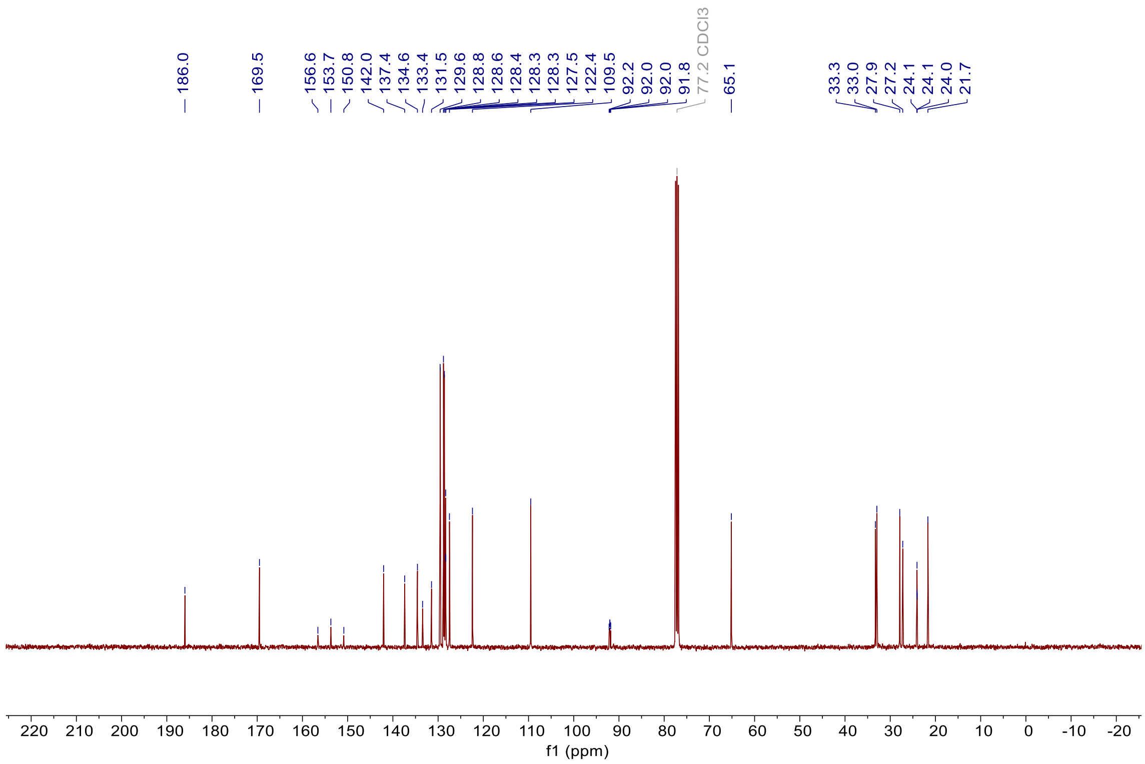

^{19}F NMR of 36 (376 Hz, $CDCl_3$)

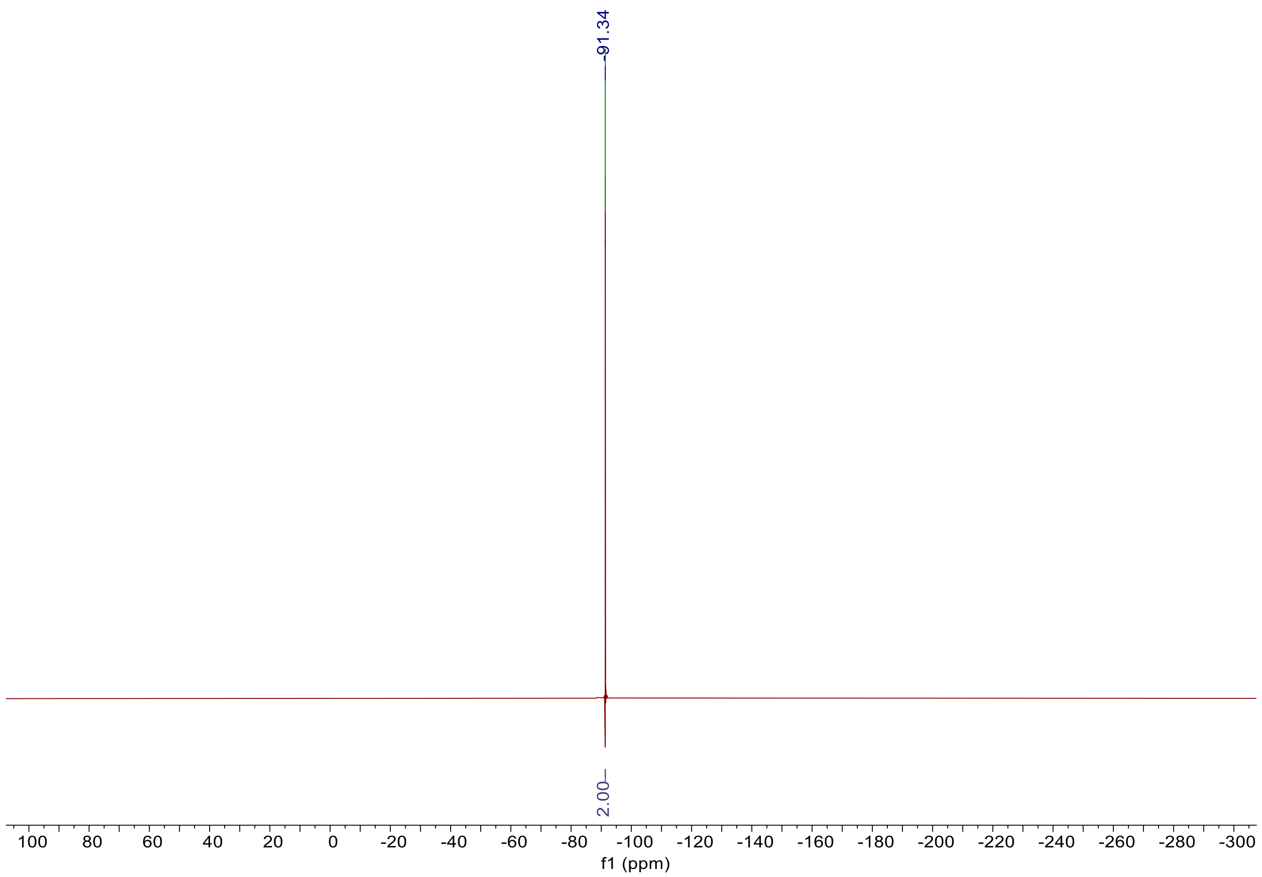

^{1}H NMR of 37 (400 MHz, $CDCl_3$)

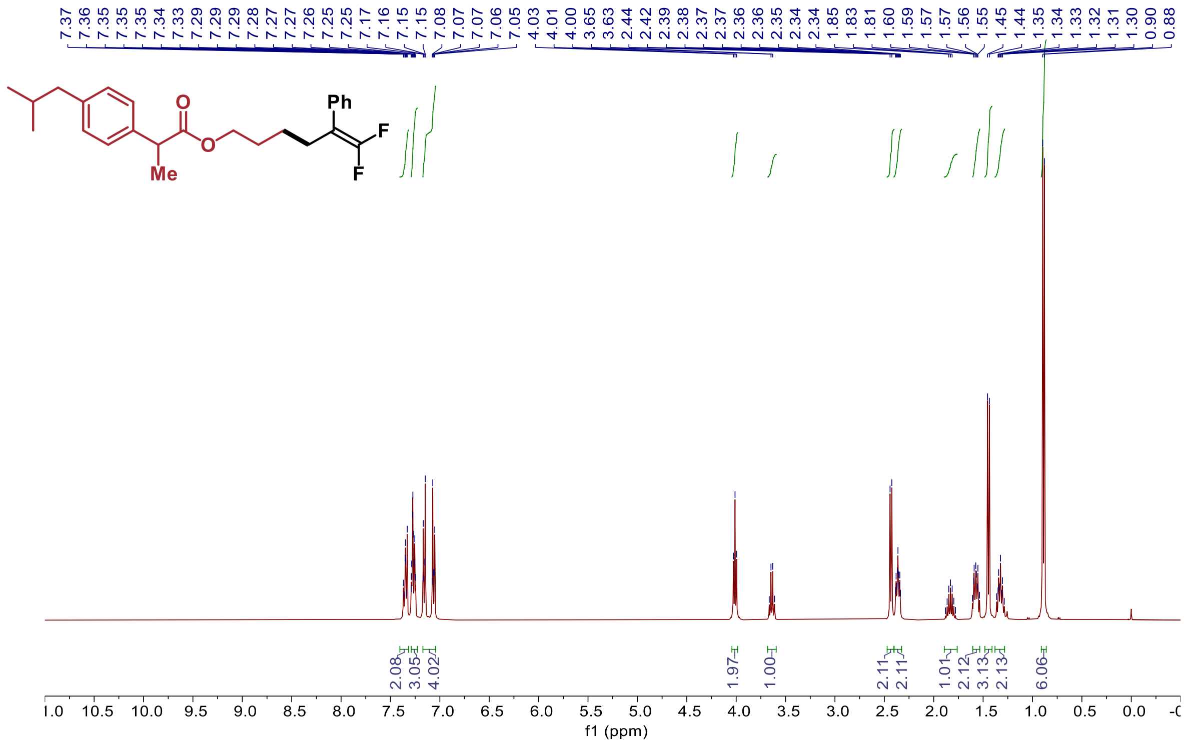

^{13}C NMR of 37 of (101 Hz, $CDCl_3$)

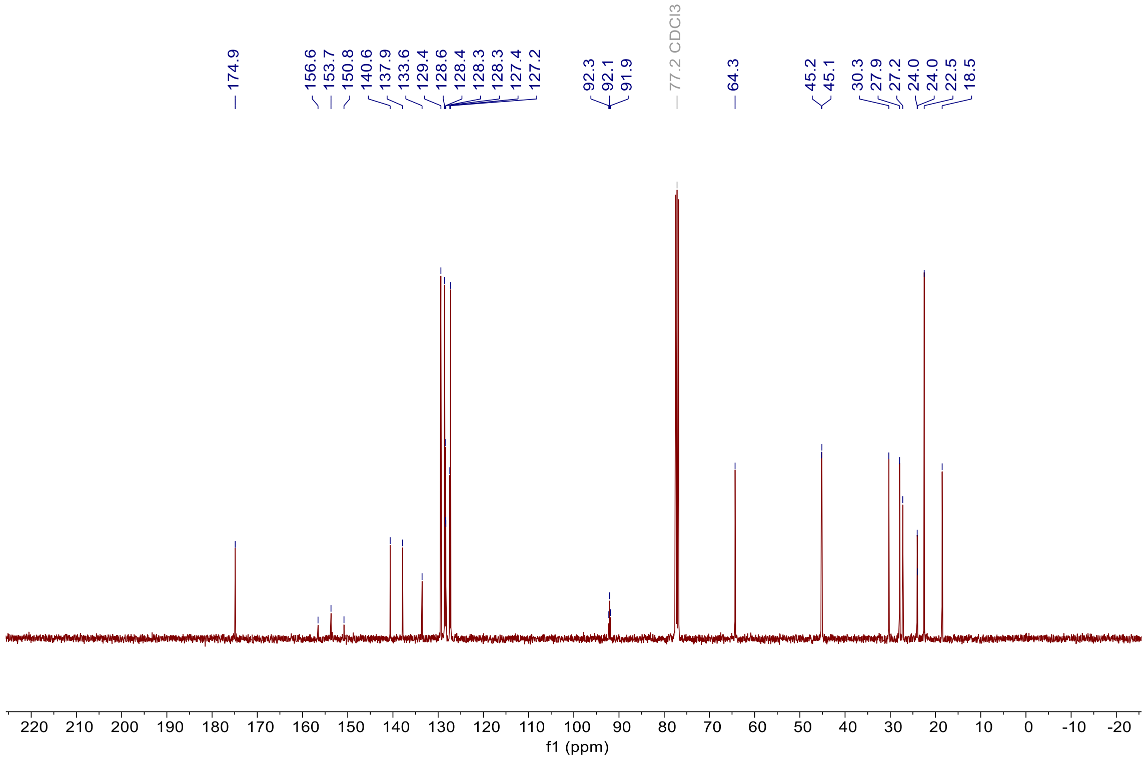

^{19}F NMR of 37 (376 MHz, $CDCl_3$)

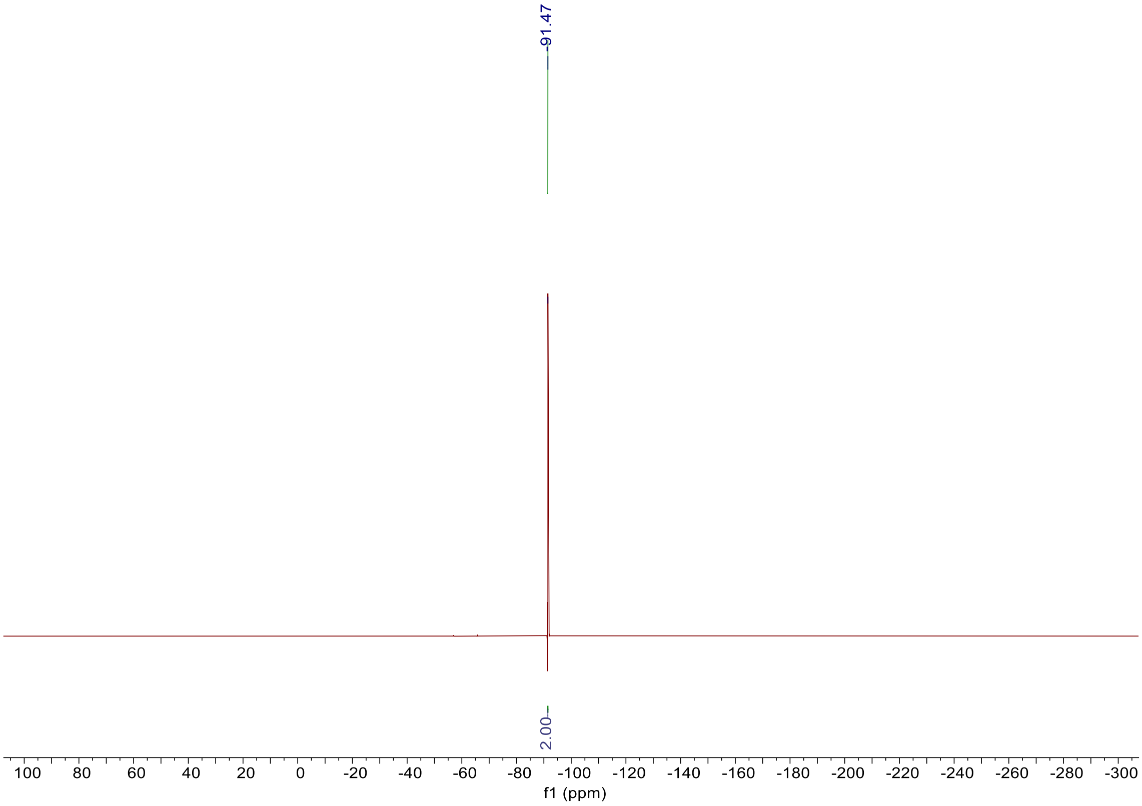

^{1}H NMR of 38 (400 MHz, $CDCl_3$)

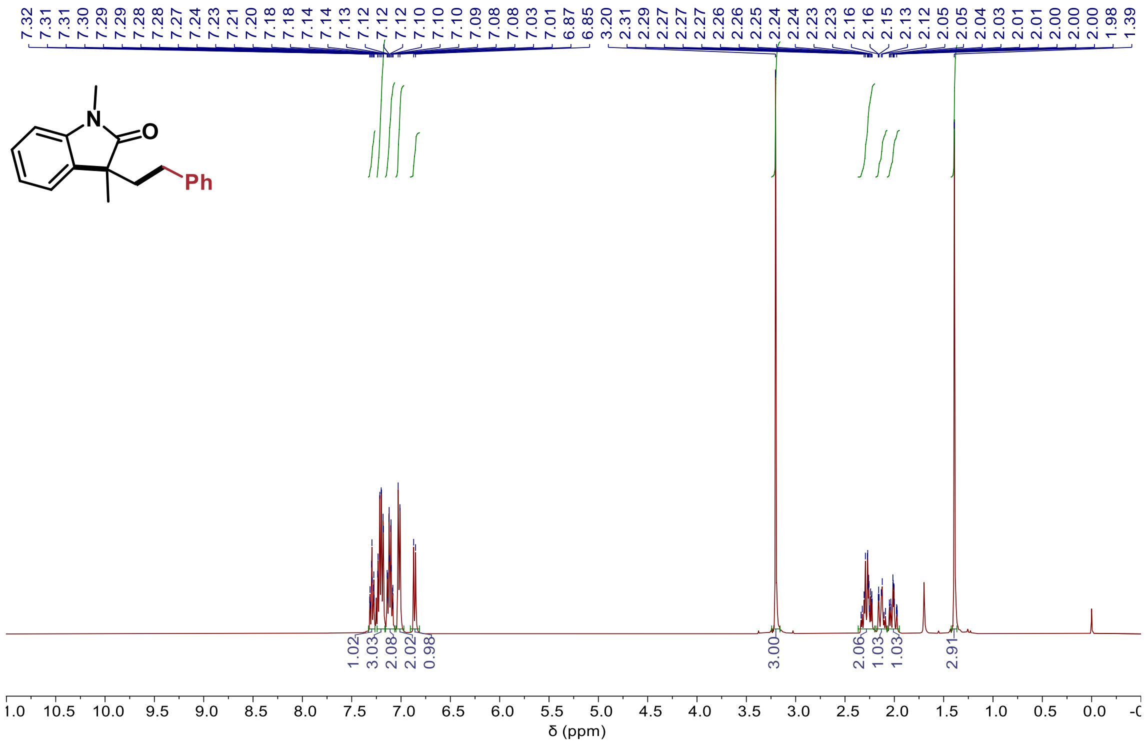

^{13}C NMR of 38 of (101 Hz, $CDCl_3$)

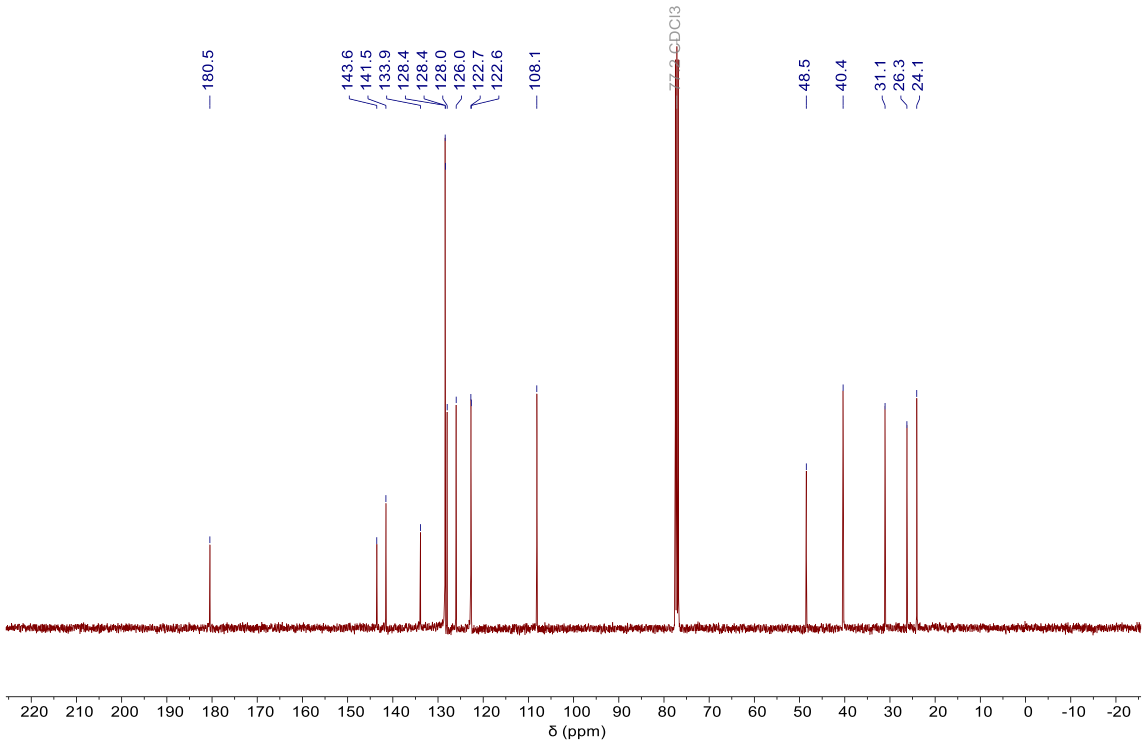

^{1}H NMR of 39 (400 MHz, $CDCl_3$)

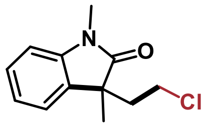

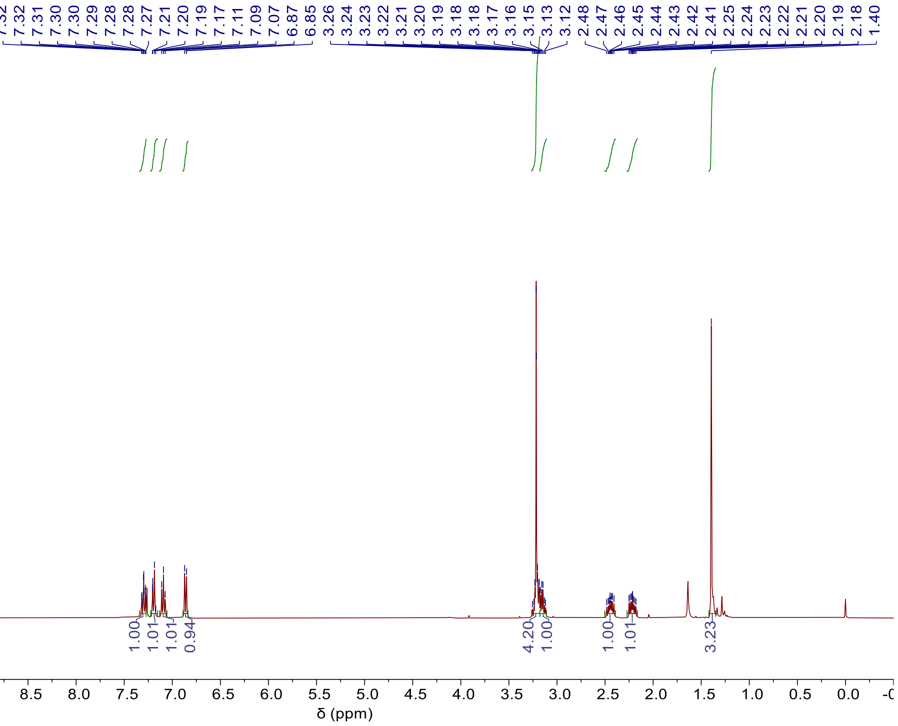

^{13}C NMR of 39 of (101 Hz, $CDCl_3$)

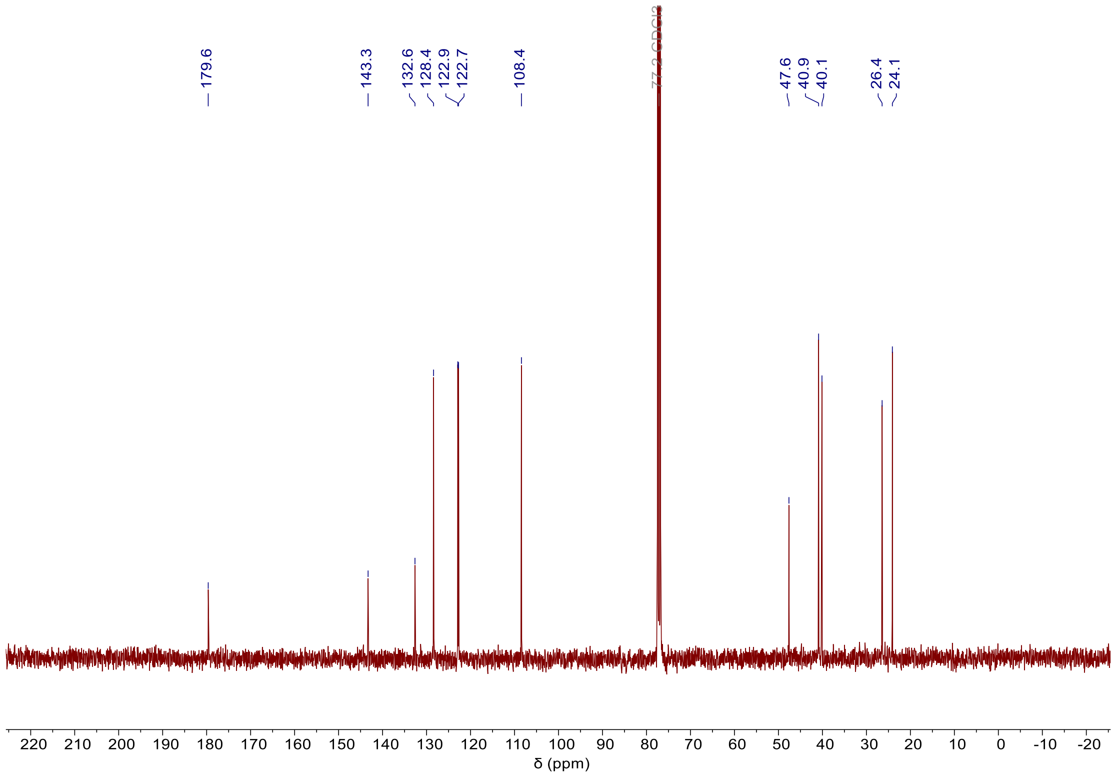

^{1}H NMR of 40 (400 MHz, $CDCl_3$)

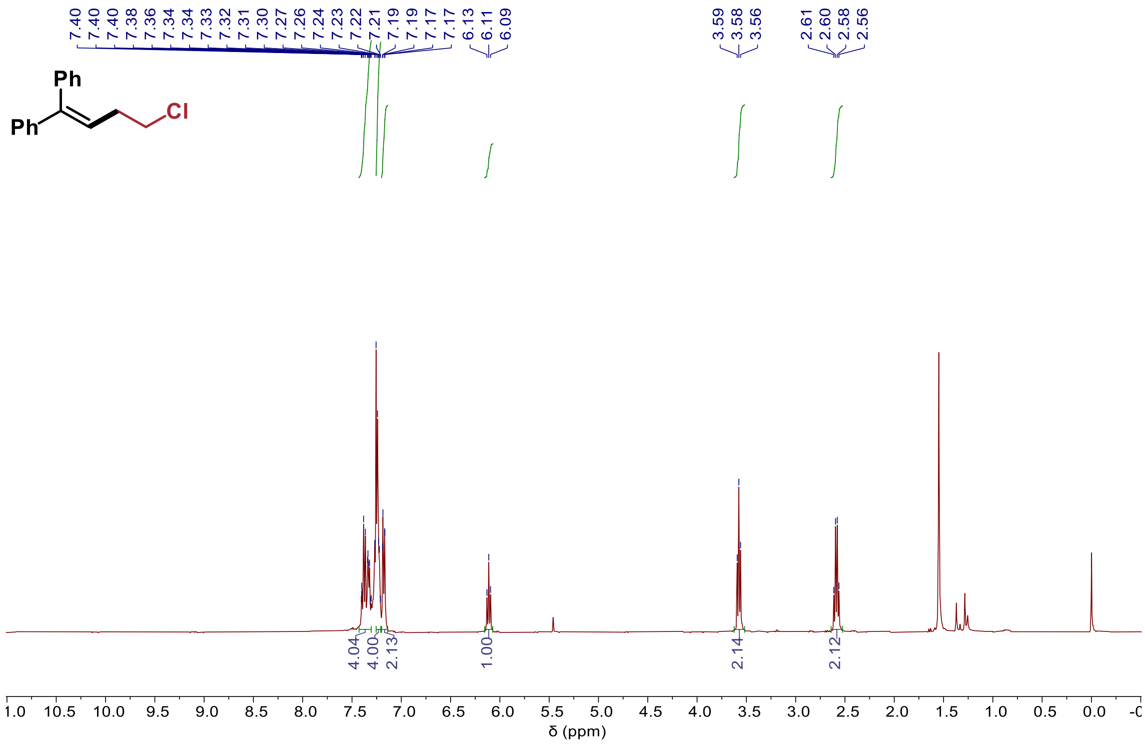

^{13}C NMR of 40 of (151 Hz, $CDCl_3$)

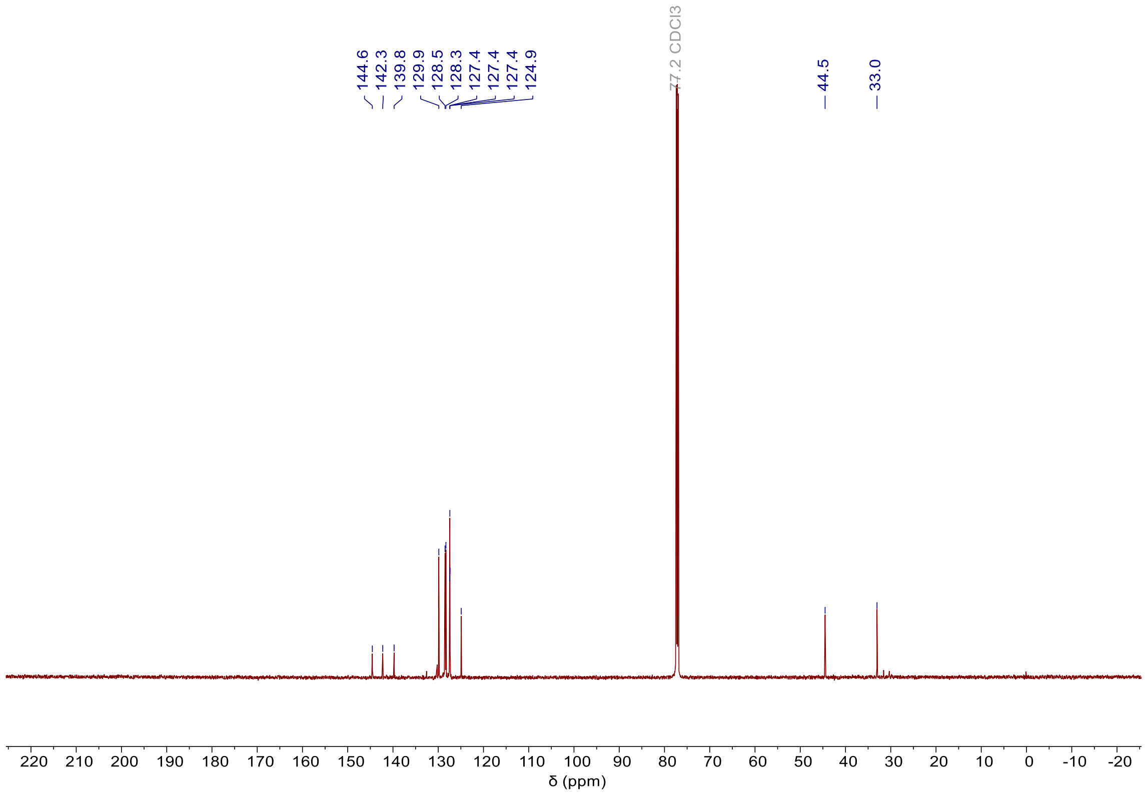

^{1}H NMR of 41 (400 MHz, CDCl$_3$)

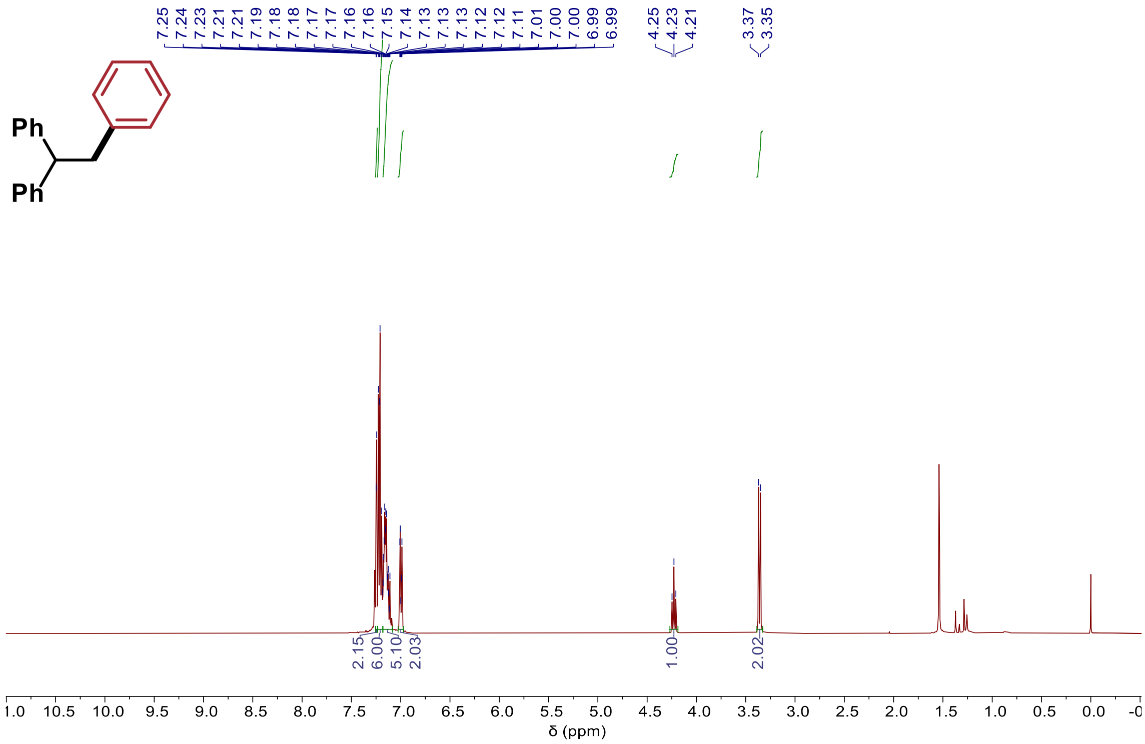

^{13}C NMR of 41 of (101 Hz, CDCl$_3$)

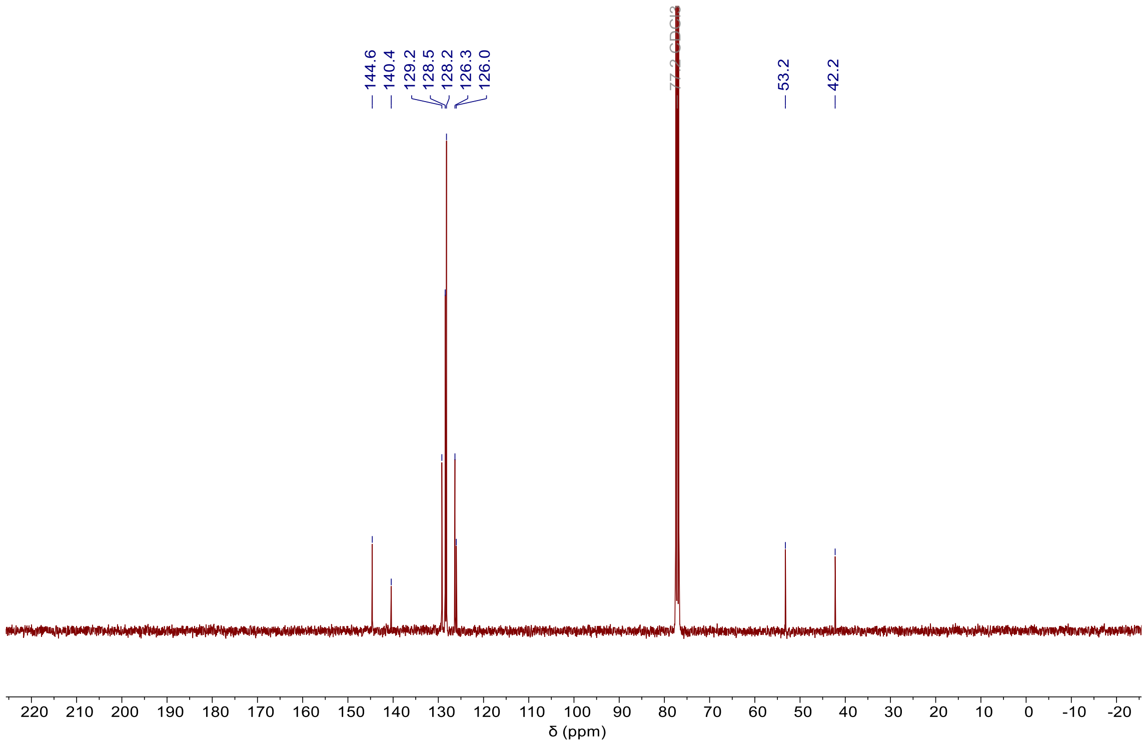

^{1}H NMR of 43 (400 MHz, CDCl$_3$)

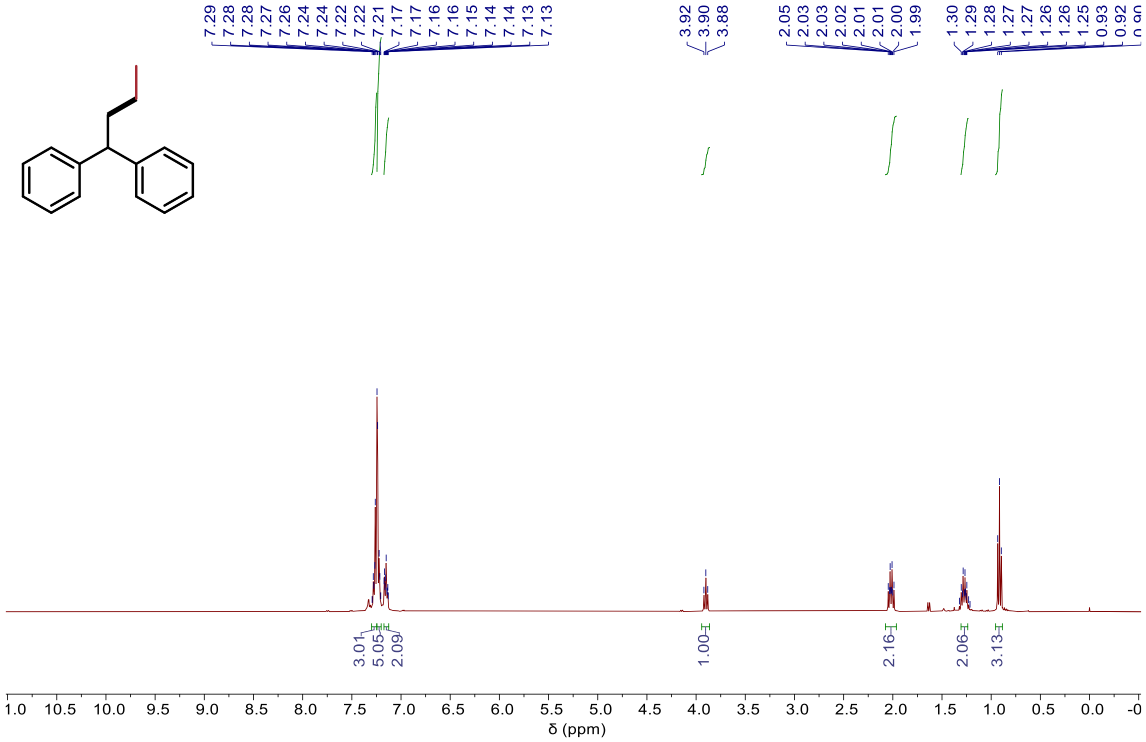

^{13}C NMR of 43 of (101 Hz, CDCl$_3$)

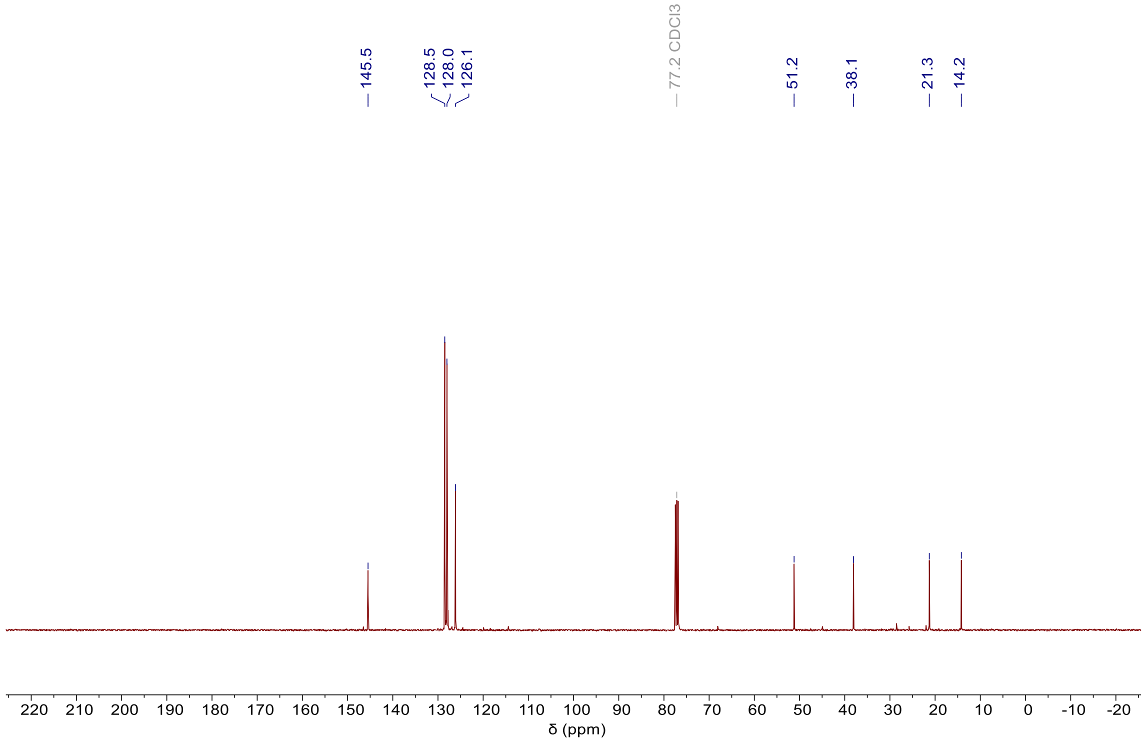

12. Computational details and additional results

All DFT calculations were carried out by using Gaussian16 Rev. A.03.[18] The B3LYP[19] functional, in combination with Grimme's D3(BJ)[20] dispersion corrections, was used throughout the DFT calculations. Solvent effects of *t*-BuCN (pivalonitrile) were included in both geometric optimizations and single point energy calculations with the SMD[21] solvent model (scrf=(smd,butanonitrile,read); eps=20.09[22], epsinf=1.896[22]). With this protocol, the structures of the intermediates and transition states were optimized and characterized by frequency analyses with the SDD[23] for I and Cs atoms and the 6-31G(d,p) basis set for other atoms (BS1 basis set). It was confirmed that the optimized intermediates have no imaginary frequency and transition states have unique imaginary frequency. When necessary, intrinsic reaction coordinate (IRC)[24] calculations were carried out to confirm that a transition state connects with two nearby minima. Single point energy calculations with the SDD for I and Cs atoms and the 6-311++G(d,p) basis set for other atoms (BS2 basis set) were carried out to improve the energies. Using the single-point energies and the thermal corrections from frequency analysis calculations, the free energies were calculated at 298.15 K and 1 M, and a correction factor of 1.89 kcal/mol for the standard state change from 1 atm to 1 M was applied.

For the transition states of the XAT and the radical replacement processes in the presence of K_3PO_4, we conducted conformational searches using the xTB program[25] via fixing the main reaction coordinates. The resulting conformations were firstly screened and then were fully optimized by using Gaussian program. The transition states with the lowest free energy were employed for the comparisons and discussions of XAT and the radical replacement processes. The other conformations of the transition states are given in Figure S21.

The selected geometries were illustrated by the CYLview program.[26]

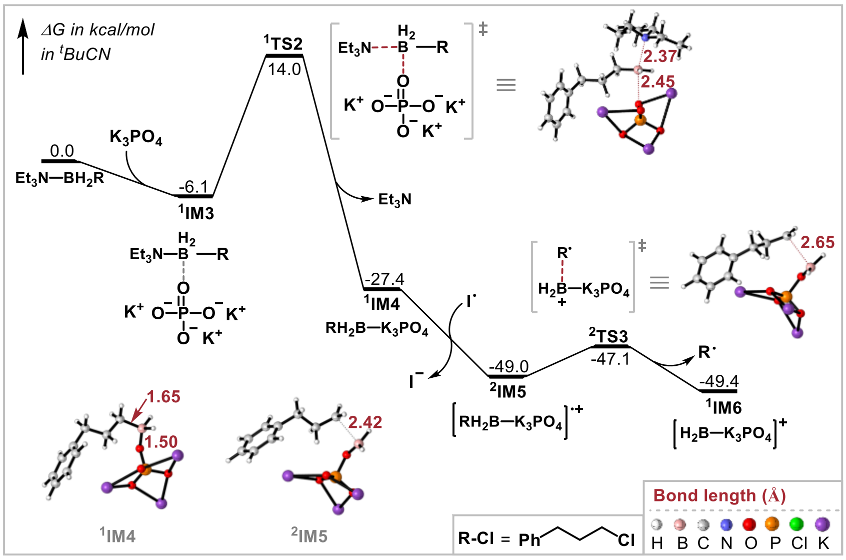

Figure S19. The free energy profile for the generation of alkyl radical from alkyl borane (Et_3NBH_2-alkyl) via base exchange and single-electron oxidation processes. The feasible energetics supports our proposed mechanism for the generation of alkyl radical from alkyl borane (Figure 4A).

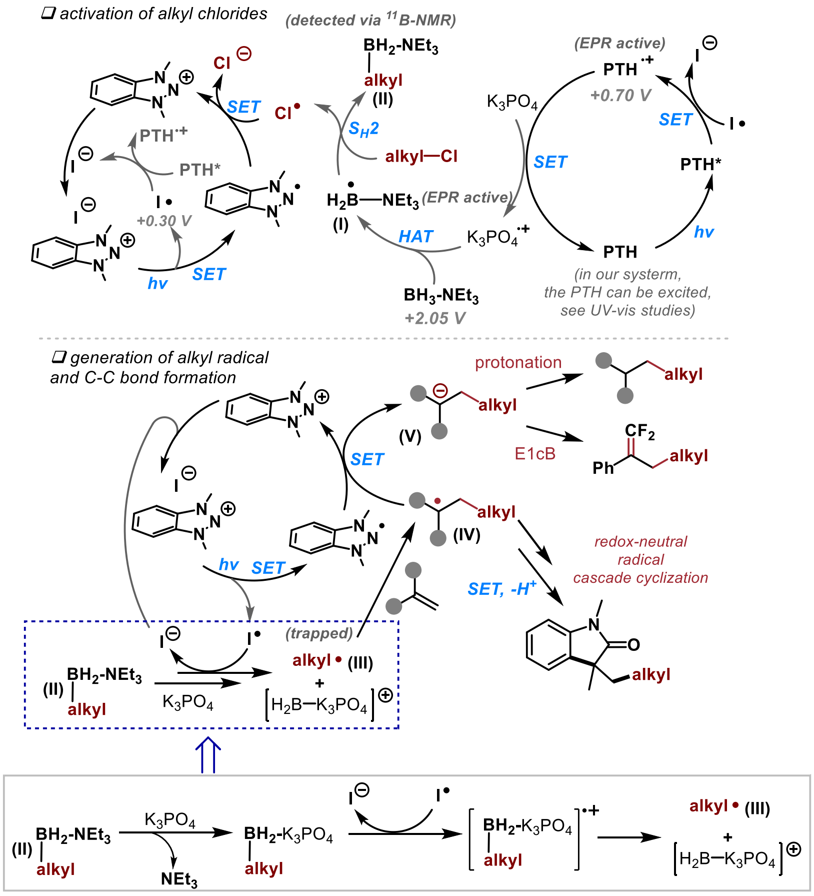

Figure S20. Proposed possible catalytic cycle.

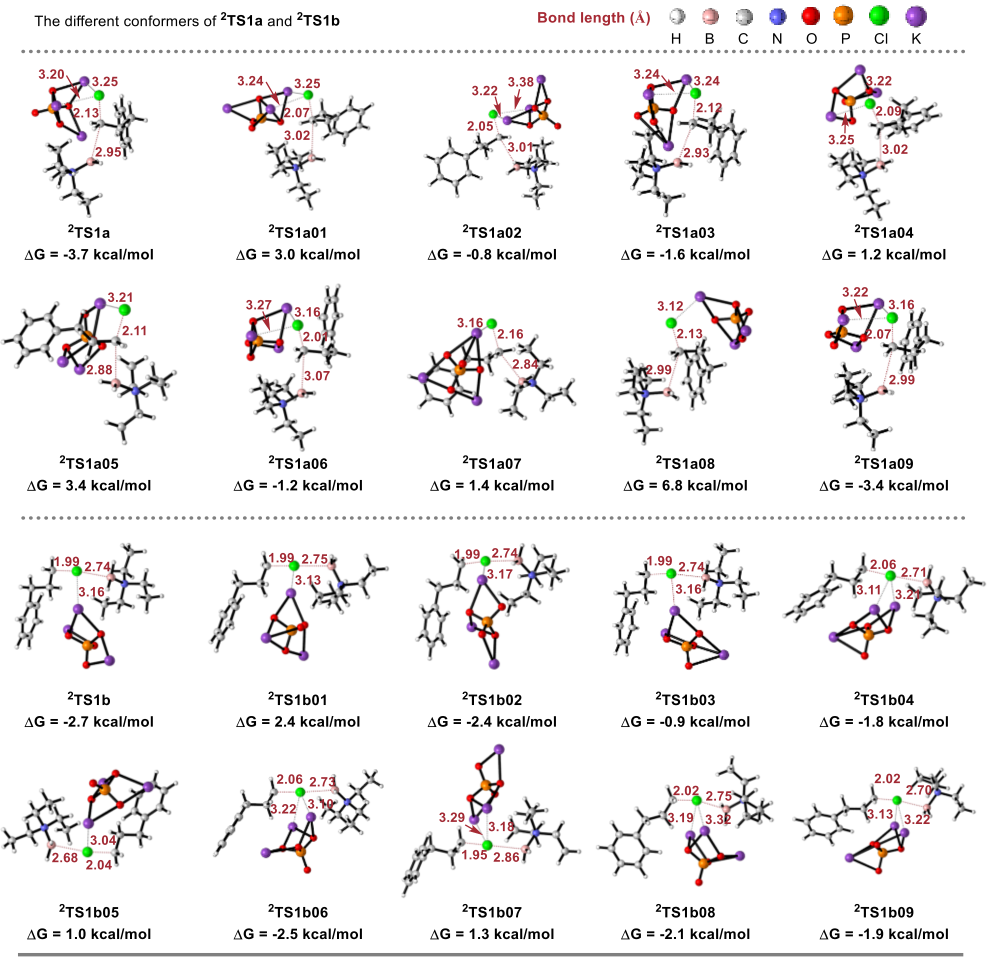

Figure S21. The different conformers of ^{2}**TS1a** and ^{2}**TS1b**, along with their free energies.

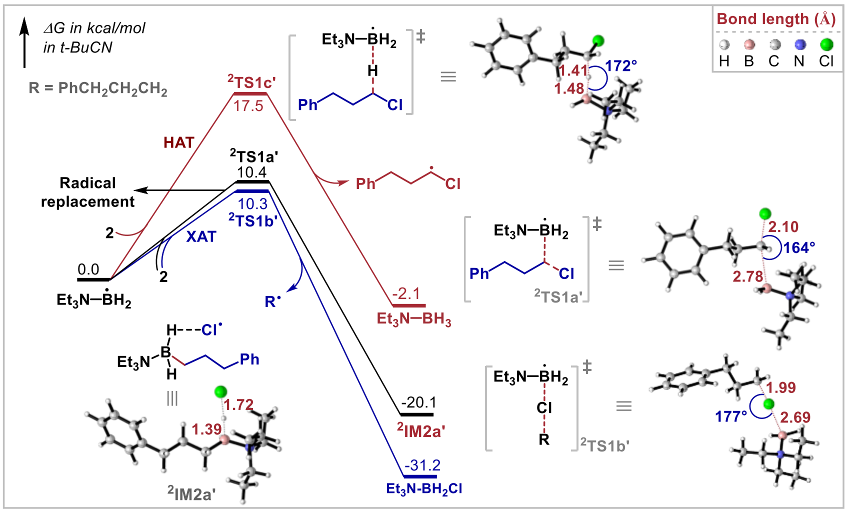

Figure S22. Comparison of the HAT, XAT and radical replacement processes in the absence of base.

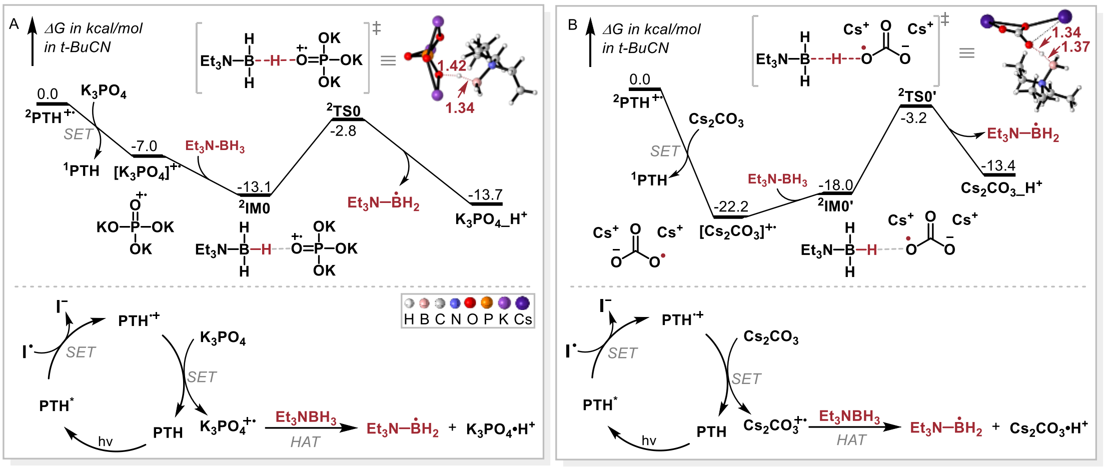

Figure S23. DFT results for K_3PO_4 (**A**) and Cs_2CO_3 (**B**) to generate LBR ($Et_3NBH_2^{\bullet}$) via one-electron oxidation, followed by HAT. The energetic results indicate that Cs_2CO_3 is inferior to K_3PO_4 in generating $Et_3NBH_2^{\bullet}$ radical.

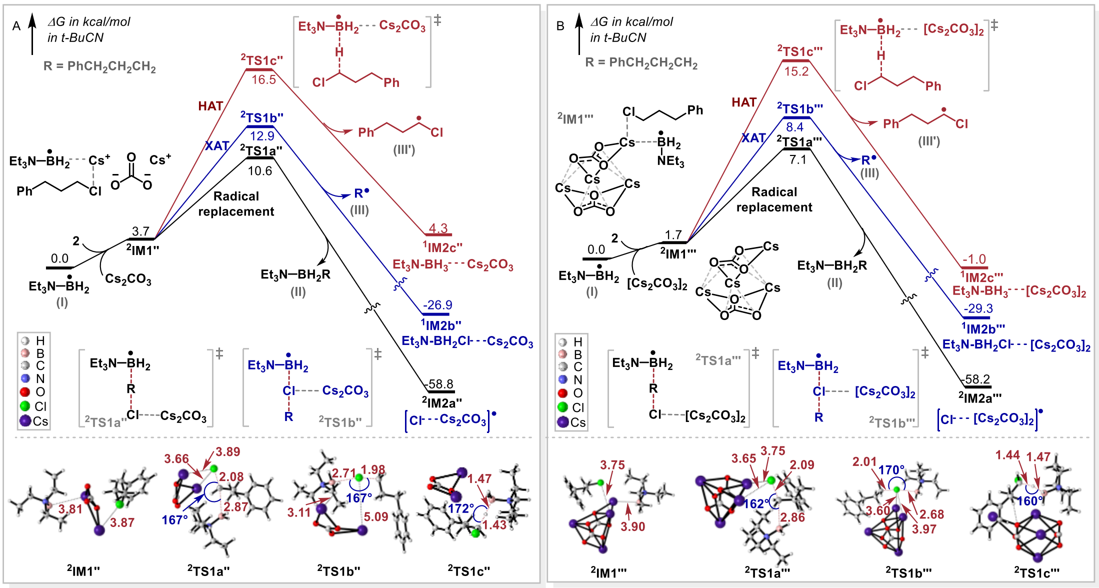

Figure S24. Comparing the energetics of the three processes with Cs_2CO_3 monomer and Cs_2CO_3 dimer. These results indicate that Cs_2CO_3 could play similar roles of K_3PO_4 but energetically inferior.

Cartesian Coordinates in Å, SCF Energies and Free Energies (in a.u.) at 298.15 K for the Optimized Structures. [G_{corr} is the correction of free energy]

$PhCH_2CH_2CH_2Cl$ (2)

B3LYP-D3BJ/BS1 G_{corr} in *t*-BuCN:
0.140199 a.u.
B3LYP-D3BJ/BS1 SCF energy in *t*-BuCN:
-809.848157 a.u.
B3LYP-D3BJ/BS2 SCF energy in *t*-BuCN:
-809.965385 a.u.
B3LYP-D3BJ/BS2 free energy in *t*-BuCN:
-809.822174 a.u.

C	3.078832	1.207278	-0.356636
C	1.809846	1.204669	0.225497
C	1.159303	-0.000147	0.524768
C	1.809997	-1.204784	0.225116
C	3.078985	-1.207048	-0.357017
C	3.718091	0.000201	-0.649786
H	3.569704	2.150974	-0.577702
H	1.316744	2.146364	0.453954
H	1.317012	-2.146612	0.453271
H	3.569976	-2.150612	-0.578383
H	4.706679	0.000335	-1.099645
C	-0.233716	-0.000322	1.109846
C	-1.312554	0.000013	0.008205
H	-0.369416	0.881768	1.745983
H	-0.369417	-0.882798	1.745446
C	-2.700881	-0.000151	0.620720
H	-1.186936	0.882921	-0.627465
H	-1.186955	-0.882531	-0.627975
H	-2.885454	-0.890706	1.223040
H	-2.885474	0.890107	1.223477
Cl	-4.002581	0.000149	-0.672840

K_3PO_4

B3LYP-D3BJ/BS1 G_{corr} in *t*-BuCN:
-0.021498 a.u.
B3LYP-D3BJ/BS1 SCF energy in *t*-BuCN:
-2442.262287 a.u.
B3LYP-D3BJ/BS2 SCF energy in *t*-BuCN:
-2442.542030 a.u.
B3LYP-D3BJ/BS2 free energy in *t*-BuCN:
-2442.560516 a.u.

P	0.001400	0.001267	0.626483
O	1.018020	-1.110314	1.059039
O	0.456964	1.437207	1.057719
O	-0.001874	-0.000488	-0.983893
O	-1.468400	-0.321269	1.064181
K	-0.784690	-2.503975	-0.472227
K	2.562164	0.573090	-0.474010
K	-1.780563	1.927722	-0.473427

Et_3N-$BH_2^{\bullet}$ (I)

B3LYP-D3BJ/BS1 G_{corr} in *t*-BuCN:
0.194320 a.u.
B3LYP-D3BJ/BS1 SCF energy in *t*-BuCN:
-318.481594 a.u.
B3LYP-D3BJ/BS2 SCF energy in *t*-BuCN:
-318.558277 a.u.
B3LYP-D3BJ/BS2 free energy in *t*-BuCN:
-318.360945 a.u.

N	0.001184	-0.006161	0.125796
C	1.259109	-0.648048	-0.419404
C	1.436539	-2.119323	-0.070758
H	2.092172	-0.078093	-0.011277
H	1.247199	-0.509491	-1.505914
H	2.447441	-2.418994	-0.364907
H	1.328398	-2.285473	1.004455
H	0.732967	-2.767424	-0.598513
C	-1.184861	-0.797909	-0.391651
C	-2.557211	-0.222731	-0.072207
H	-1.101295	-1.788782	0.052481
H	-1.057406	-0.890748	-1.476339
H	-3.306553	-0.937721	-0.426860
H	-2.699783	-0.091037	1.002335
H	-2.748955	0.728146	-0.574412
C	-0.094117	1.400191	-0.442122
C	1.094530	2.300670	-0.140459
H	-0.994575	1.837842	-0.013939
H	-0.238251	1.304692	-1.524866
H	0.846280	3.311612	-0.478781
H	1.299126	2.342702	0.932644

H	2.004198	1.992857	-0.661363
B	0.029887	0.009669	1.723572
H	1.141410	0.123907	2.168317
H	-0.874023	0.643687	2.198118

[2]IM1

B3LYP-D3BJ/BS1 G_{corr} in *t*-BuCN:
0.357183 a.u.
B3LYP-D3BJ/BS1 SCF energy in *t*-BuCN:
-3570.655927 a.u.
B3LYP-D3BJ/BS2 SCF energy in *t*-BuCN:
-3571.118515 a.u.
B3LYP-D3BJ/BS2 free energy in *t*-BuCN:
-3570.758320 a.u.

P	0.092693	1.247922	1.406076
O	-1.376259	1.041145	1.911420
O	0.141527	2.228778	0.146996
O	1.026839	1.837111	2.508893
O	0.621196	-0.161614	0.892195
K	-1.455362	-1.512123	1.437978
K	1.855544	3.786690	1.175169
K	1.068379	0.511919	-1.507823
N	-4.311900	0.201382	-0.804337
C	-5.174077	0.418534	0.420673
C	-4.509382	0.108812	1.756148
H	-6.054169	-0.211904	0.300199
H	-5.497910	1.464211	0.390591
H	-5.195511	0.426127	2.549667
H	-4.358464	-0.968580	1.874250
H	-3.554840	0.625286	1.898472
C	-3.168916	1.203841	-0.736827
C	-2.208407	1.205548	-1.916335
H	-2.595937	1.018011	0.176775
H	-3.635697	2.191937	-0.643870
H	-1.380390	1.847797	-1.596657
H	-1.827381	0.198697	-2.115341
H	-2.640745	1.598643	-2.840741
C	-5.143137	0.543471	-2.030370
C	-6.378594	-0.317524	-2.249419
H	-4.474864	0.436305	-2.883686
H	-5.420658	1.600313	-1.944292
H	-6.809599	-0.047640	-3.218912
H	-6.125516	-1.380574	-2.275419
H	-7.149856	-0.158699	-1.491955
B	-3.792148	-1.301854	-0.884522
H	-3.283566	-1.567377	-1.940772
H	-4.540441	-2.090097	-0.369330
C	4.490335	1.452586	-0.482566
C	4.286887	0.225625	0.157818
C	4.169876	-0.961580	-0.582188
C	4.258632	-0.887868	-1.982903
C	4.447892	0.339628	-2.628481
C	4.561157	1.517342	-1.878895
H	4.608629	2.357544	0.108861
H	4.209204	0.188156	1.240462
H	4.174180	-1.798735	-2.570674
H	4.513435	0.375611	-3.712358
H	4.715209	2.470367	-2.376295
C	3.867921	-2.276446	0.101138
C	2.361680	-2.606186	0.006970
H	4.159785	-2.215250	1.155384
H	4.455259	-3.081896	-0.355384
C	2.039612	-3.869750	0.778399
H	1.783723	-1.764926	0.410422
H	2.089605	-2.744521	-1.047201
H	2.561525	-4.750472	0.401971
H	2.225073	-3.761164	1.847816
Cl	0.246213	-4.291716	0.641981

[2]TS1a

B3LYP-D3BJ/BS1 G_{corr} in *t*-BuCN:
0.359012 a.u.
B3LYP-D3BJ/BS1 SCF energy in *t*-BuCN:
-3570.645884 a.u.
B3LYP-D3BJ/BS2 SCF energy in *t*-BuCN:
-3571.111550 a.u.
B3LYP-D3BJ/BS2 free energy in *t*-BuCN:
-3570.749526 a.u.

C	0.811274	4.177773	0.965001
C	0.620963	3.687805	-0.331030
C	1.599480	2.891014	-0.953168
C	2.774216	2.607838	-0.239461
C	2.968744	3.089604	1.060537
C	1.985551	3.876230	1.669067
H	0.045154	4.795602	1.425271
H	-0.292679	3.930484	-0.866332

H	3.555405	2.022470	-0.719179
H	3.884001	2.849564	1.593704
H	2.131826	4.254310	2.676558
C	1.419019	2.397772	-2.372100
C	0.125960	1.612548	-2.647791
H	2.281800	1.788743	-2.655939
H	1.413847	3.266479	-3.042095
C	0.004692	0.239884	-2.043922
H	-0.736129	2.193813	-2.298625
H	0.000939	1.515131	-3.731224
H	0.216731	0.034640	-0.999647
H	-0.812357	-0.350598	-2.435229
Cl	1.597204	-0.903219	-2.874816
N	-3.593326	0.100739	0.064589
C	-4.081159	-1.050237	-0.789168
C	-3.027802	-2.094589	-1.122646
H	-4.465374	-0.617336	-1.711607
H	-4.914471	-1.508650	-0.248047
H	-3.509991	-2.887465	-1.703184
H	-2.241199	-1.651378	-1.736401
H	-2.586109	-2.536678	-0.227520
C	-3.021239	-0.478891	1.352301
C	-2.889225	0.509921	2.508029
H	-2.044793	-0.915265	1.108033
H	-3.695186	-1.286403	1.656051
H	-2.269277	0.042281	3.281333
H	-2.432105	1.458737	2.210656
H	-3.848641	0.749898	2.971145
C	-4.804633	0.958627	0.419802
C	-5.615023	1.479974	-0.758200
H	-4.419639	1.798956	0.995457
H	-5.435450	0.349855	1.076600
H	-6.371011	2.164251	-0.360288
H	-4.994890	2.037324	-1.464058
H	-6.140280	0.688009	-1.296370
B	-2.521602	0.973117	-0.701178
H	-2.809823	1.213598	-1.832935
H	-1.951311	1.766397	-0.010774
P	1.212014	-2.081178	1.621144
O	-0.093481	-1.552578	0.847868
O	0.844269	-2.818345	2.917638
O	1.993036	-2.993758	0.579688
O	2.107757	-0.790607	1.877875
K	0.196944	0.861498	1.675872
K	0.140764	-3.158375	-1.129411
K	3.495558	-1.090200	-0.237772

[2]TS1b

B3LYP-D3BJ/BS1 G_{corr} in *t*-BuCN:
0.357735 a.u.
B3LYP-D3BJ/BS1 SCF energy in *t*-BuCN:
-3570.643630 a.u.
B3LYP-D3BJ/BS2 SCF energy in *t*-BuCN:
-3571.108743 a.u.
B3LYP-D3BJ/BS2 free energy in *t*-BuCN:
-3570.747996 a.u.

P	-0.233774	2.642266	-0.199050
O	-1.674864	2.094487	0.221110
O	0.073877	2.287592	-1.696287
O	-0.219851	4.181286	0.064390
O	0.829545	1.876101	0.706016
K	-0.835793	0.401380	1.944275
K	-2.621313	4.367212	0.779115
K	2.069502	0.802138	-1.244357
N	-3.329542	-1.734741	-0.715813
C	-4.813267	-1.497552	-0.964743
C	-5.350501	-0.171533	-0.444508
H	-5.343035	-2.320248	-0.485967
H	-4.967050	-1.571423	-2.047120
H	-6.430997	-0.157091	-0.619432
H	-5.180077	-0.059350	0.628854
H	-4.917646	0.688359	-0.960310
C	-2.570504	-0.553269	-1.309239
C	-1.077939	-0.745751	-1.530962
H	-2.696216	0.302802	-0.643873
H	-3.054223	-0.330547	-2.266421
H	-0.683420	0.259310	-1.740880
H	-0.583856	-1.136255	-0.638257
H	-0.853022	-1.416267	-2.366041
C	-2.917861	-2.985898	-1.466595
C	-3.705739	-4.250277	-1.154440
H	-1.867361	-3.144894	-1.228186
H	-2.998522	-2.748591	-2.532581
H	-3.255987	-5.066831	-1.728599
H	-3.653258	-4.516006	-0.097083
H	-4.755099	-4.181675	-1.450132
B	-3.088571	-1.884583	0.841703

H -3.654612 -2.819024 1.326852
H -3.091015 -0.835460 1.418947
C 5.393678 0.948576 0.051489
C 4.794561 -0.018931 0.865548
C 4.451574 -1.280651 0.352148
C 4.727527 -1.552462 -0.998648
C 5.327161 -0.588263 -1.816400
C 5.658981 0.668261 -1.294325
H 5.650059 1.919247 0.466262
H 4.582279 0.207382 1.907359
H 4.467288 -2.524558 -1.410243
H 5.536102 -0.817201 -2.857614
H 6.123747 1.418019 -1.927692
C 3.700548 -2.280199 1.200486
C 2.175142 -2.092538 1.033129
H 3.966252 -2.153260 2.255384
H 3.971369 -3.301293 0.911025
C 1.364692 -3.057549 1.863541
H 1.919535 -1.061015 1.308359
H 1.917151 -2.222824 -0.025746
H 1.508202 -4.103129 1.591759
H 1.465262 -2.911864 2.939158
Cl -0.559946 -2.710328 1.486928

^{2}**TS1c**

B3LYP-D3BJ/BS1 G_{corr} in *t*-BuCN:
0.352470 a.u.
B3LYP-D3BJ/BS1 SCF energy in *t*-BuCN:
-3570.626613 a.u.
B3LYP-D3BJ/BS2 SCF energy in *t*-BuCN:
-3571.092531 a.u.
B3LYP-D3BJ/BS2 free energy in *t*-BuCN:
-3570.737049 a.u.

P 1.883484 -2.075389 0.632115
O 1.564454 -1.053592 1.775994
O 2.255876 -1.292978 -0.715468
O 3.088789 -3.003989 0.981216
O 0.555002 -2.890120 0.322429
K -0.883160 -1.651574 2.045175
K 4.633246 -2.141547 -0.804351
K 0.089983 -1.763951 -1.923732
N -3.889020 0.128243 -0.942116
C -4.783374 -0.732831 -0.075948
C -4.726075 -0.436233 1.414658
H -4.474424 -1.761833 -0.251274
H -5.805371 -0.610507 -0.449273
H -5.257290 -1.235829 1.940071
H -3.700765 -0.407866 1.786847
H -5.204703 0.509231 1.679153
C -4.256041 1.580157 -0.710301
C -3.496028 2.587382 -1.560801
H -4.064317 1.780748 0.341728
H -5.333922 1.666405 -0.883993
H -3.773941 3.589101 -1.218247
H -2.415467 2.481776 -1.447748
H -3.746902 2.522418 -2.622037
C -4.186257 -0.203752 -2.393625
C -4.025119 -1.669866 -2.771225
H -3.506165 0.402453 -2.989160
H -5.210909 0.127149 -2.593534
H -4.095477 -1.747132 -3.860487
H -3.053261 -2.066869 -2.467215
H -4.803162 -2.305027 -2.342144
B -2.341101 -0.137832 -0.619807
H -1.665291 0.216800 -1.555154
H -2.179213 -1.286646 -0.286644
C 3.691313 4.372791 -0.568726
C 2.336428 4.042537 -0.473442
C 1.924984 2.705019 -0.404576
C 2.895439 1.688790 -0.428563
C 4.247962 2.025768 -0.522123
C 4.654194 3.362224 -0.593209
H 3.991942 5.415668 -0.623634
H 1.588919 4.832495 -0.451946
H 2.593713 0.634977 -0.381693
H 5.000758 1.239326 -0.537030
H 5.709050 3.613149 -0.666090
C 0.459661 2.357009 -0.264813
C 0.122410 1.859175 1.157081
H 0.187977 1.578831 -0.985886
H -0.149147 3.237899 -0.501199
C -1.369493 1.729958 1.337037
H 0.630438 0.899045 1.339595
H 0.518741 2.584715 1.883088
H -1.939157 2.632996 1.115365
H -1.896214 0.745211 0.456922
Cl -1.816728 1.266744 3.058652

Et_3N-BH_2R (II)

B3LYP-D3BJ/BS1 G_{corr} in *t*-BuCN:
0.360302 a.u.

B3LYP-D3BJ/BS1 SCF energy in *t*-BuCN:
-668.203079 a.u.

B3LYP-D3BJ/BS2 SCF energy in *t*-BuCN:
-668.358033 a.u.

B3LYP-D3BJ/BS2 free energy in *t*-BuCN:
-667.994719 a.u.

C	5.352500	-1.163246	0.553905
C	4.095466	-1.226920	-0.050828
C	3.424037	-0.060820	-0.445333
C	4.046916	1.174457	-0.214564
C	5.303420	1.244292	0.389954
C	5.961625	0.074076	0.776874
H	5.857612	-2.079270	0.848053
H	3.626747	-2.192900	-0.223212
H	3.540351	2.088406	-0.515659
H	5.770328	2.211392	0.555745
H	6.940632	0.125949	1.244536
C	2.040463	-0.128613	-1.045861
C	0.925760	-0.033958	0.014952
H	1.924399	-1.067941	-1.600452
H	1.911084	0.686609	-1.768747
C	-0.492806	-0.097374	-0.570617
H	1.068028	-0.844051	0.744007
H	1.057148	0.900798	0.577330
H	-0.570609	-1.023173	-1.160559
H	-0.593037	0.717953	-1.300347
N	-3.178415	0.022629	0.086891
C	-3.638969	1.442567	-0.149617
C	-2.858297	2.251239	-1.175227
H	-3.577936	1.950947	0.810894
H	-4.694750	1.388479	-0.438939
H	-3.343784	3.228929	-1.261439
H	-1.827379	2.416951	-0.859327
H	-2.854667	1.799296	-2.169817
C	-3.376158	-0.751086	-1.195232
C	-3.141340	-2.253766	-1.093931
H	-2.692838	-0.324634	-1.926496
H	-4.398492	-0.555426	-1.537638
H	-3.113793	-2.661380	-2.109438
H	-2.190258	-2.487296	-0.611552
H	-3.940380	-2.768690	-0.555641
C	-4.097438	-0.578473	1.128182
C	-4.107161	0.106743	2.488223
H	-3.778734	-1.610786	1.257443
H	-5.107355	-0.583646	0.701861
H	-4.733699	-0.492876	3.156547
H	-3.107081	0.160490	2.922049
H	-4.532646	1.112493	2.458916
B	-1.603263	-0.035762	0.613222
H	-1.467542	0.961234	1.305046
H	-1.534241	-1.032154	1.317211

PhCH2CH2CH$_2^•$ (III)

B3LYP-D3BJ/BS1 G_{corr} in *t*-BuCN:
0.135137 a.u.

B3LYP-D3BJ/BS1 SCF energy in *t*-BuCN:
-349.574273 a.u.

B3LYP-D3BJ/BS2 SCF energy in *t*-BuCN:
-349.659975 a.u.

B3LYP-D3BJ/BS2 free energy in *t*-BuCN:
-349.521826 a.u.

C	2.101882	1.207124	-0.134058
C	0.748833	1.204504	0.210178
C	0.053939	-0.000019	0.388965
C	0.748853	-1.204523	0.210124
C	2.101902	-1.207105	-0.134112
C	2.783471	0.000019	-0.307927
H	2.624494	2.150867	-0.263450
H	0.223255	2.146546	0.346613
H	0.223290	-2.146580	0.346516
H	2.624530	-2.150833	-0.263547
H	3.836814	0.000034	-0.572887
C	-1.421318	-0.000038	0.707205
C	-2.294378	0.000027	-0.586007
H	-1.677931	0.882498	1.303016
H	-1.677928	-0.882636	1.302925
C	-3.754170	0.000009	-0.298496
H	-2.017143	0.882616	-1.176367
H	-2.017139	-0.882499	-1.176458
H	-4.278158	-0.928861	-0.090850
H	-4.278159	0.928854	-0.090736

$PhCH_2CH_2CHCl^{\bullet}$ (III')

B3LYP-D3BJ/BS1 G_{corr} in *t*-BuCN:
0.124516 a.u.
B3LYP-D3BJ/BS1 SCF energy in *t*-BuCN:
-809.178253 a.u.
B3LYP-D3BJ/BS2 SCF energy in *t*-BuCN:
-809.295939 a.u.
B3LYP-D3BJ/BS2 free energy in *t*-BuCN:
-809.168411 a.u.

C	-3.025222	-1.274009	-0.183415
C	-1.746356	-1.170777	0.367014
C	-1.123929	0.077028	0.508274
C	-1.812493	1.221179	0.082804
C	-3.091620	1.122773	-0.468218
C	-3.702307	-0.126391	-0.603302
H	-3.494025	-2.249196	-0.281246
H	-1.223695	-2.066030	0.694809
H	-1.341793	2.195513	0.188784
H	-3.612339	2.020891	-0.788521
H	-4.698591	-0.204650	-1.028761
C	0.278114	0.180405	1.060650
C	1.338658	0.068056	-0.054307
H	0.450436	-0.612311	1.796344
H	0.405136	1.138241	1.577380
C	2.714389	0.189929	0.492427
H	1.225146	-0.888262	-0.581177
H	1.152114	0.851904	-0.808015
H	2.968168	0.816346	1.340519
Cl	4.070237	-0.160683	-0.548334

[2]IM2a

B3LYP-D3BJ/BS1 G_{corr} in *t*-BuCN:
-0.024625 a.u.
B3LYP-D3BJ/BS1 SCF energy in *t*-BuCN:
-2902.519415 a.u.
B3LYP-D3BJ/BS2 SCF energy in *t*-BuCN:
-2902.818915 a.u.
B3LYP-D3BJ/BS2 free energy in *t*-BuCN:
-2902.840528 a.u.

P	-1.549482	0.000626	0.143992
O	-1.021896	1.278636	0.836056
O	-3.124750	0.000328	-0.045842
O	-1.167936	-0.002825	-1.387885
O	-1.022135	-1.274372	0.841738
K	0.745275	0.005290	2.341882
K	0.453479	2.133329	-1.178534
K	0.452339	-2.138927	-1.169476
Cl	2.503835	-0.001040	-0.235058

[1]IM2b

B3LYP-D3BJ/BS1 G_{corr} in *t*-BuCN:
0.201570 a.u.
B3LYP-D3BJ/BS1 SCF energy in *t*-BuCN:
-3221.098930 a.u.
B3LYP-D3BJ/BS2 SCF energy in *t*-BuCN:
-3221.488605 a.u.
B3LYP-D3BJ/BS2 free energy in *t*-BuCN:
-3221.284023 a.u.

P	2.986895	-0.014383	0.082520
O	2.478833	1.293053	0.839261
O	2.455802	0.089820	-1.419468
O	4.511726	-0.185672	0.152008
O	2.210476	-1.250421	0.742834
K	1.077445	0.108766	2.563947
K	1.195733	2.266787	-1.123272
K	1.252875	-2.100349	-1.438646
N	-2.662598	-0.584229	-0.003625
C	-3.703157	-0.592891	1.104368
C	-4.325709	0.746457	1.464079
H	-3.207034	-1.005247	1.982431
H	-4.477294	-1.298112	0.787876
H	-5.075648	0.555436	2.238619
H	-3.592338	1.444948	1.869134
H	-4.835055	1.221594	0.622965
C	-3.370618	-0.301373	-1.315276
C	-2.481720	-0.233272	-2.550967
H	-3.883850	0.649111	-1.187202
H	-4.126741	-1.083315	-1.431793
H	-3.107937	0.097331	-3.385692
H	-1.671414	0.488756	-2.438836
H	-2.061540	-1.201815	-2.833359
C	-2.101005	-1.995518	-0.076501
C	-1.127700	-2.400709	1.025846
H	-1.618106	-2.084419	-1.048606
H	-2.963389	-2.667012	-0.089752

H	-0.919261	-3.469112	0.900530
H	-0.173964	-1.867757	0.965519
H	-1.546319	-2.274265	2.027286
B	-1.448723	0.426756	0.309473
H	-1.286230	0.423937	1.498910
H	-0.488944	0.087590	-0.322305
Cl	-1.851576	2.236155	-0.198850

[1]IM2c

B3LYP-D3BJ/BS1 G_{corr} in *t*-BuCN:
0.207188 a.u.
B3LYP-D3BJ/BS1 SCF energy in *t*-BuCN:
-2761.445350 a.u.
B3LYP-D3BJ/BS2 SCF energy in *t*-BuCN:
-2761.802538 a.u.
B3LYP-D3BJ/BS2 free energy in *t*-BuCN:
-2761.592338 a.u.

P	-2.876232	0.025882	-0.010631
O	-2.388533	1.085704	1.077060
O	-2.363246	-1.402612	0.485384
O	-4.396052	0.065786	-0.229748
O	-2.062066	0.351054	-1.351198
K	-0.957727	2.461336	-0.474509
K	-1.013498	-0.529398	2.459017
K	-0.879873	-1.876929	-1.482937
N	2.844886	-0.026987	-0.138577
C	3.796866	1.149139	-0.060619
C	4.107022	1.662872	1.337354
H	3.340716	1.949155	-0.644066
H	4.717598	0.842958	-0.568041
H	4.808306	2.497242	1.234104
H	3.213038	2.033372	1.842041
H	4.583681	0.912821	1.973173
C	3.565534	-1.250442	0.386296
C	2.719438	-2.509315	0.525027
H	3.958321	-0.979868	1.365398
H	4.413414	-1.431104	-0.282321
H	3.361788	-3.296087	0.933321
H	1.884968	-2.366152	1.213923
H	2.332678	-2.877570	-0.429489
C	2.545547	-0.257638	-1.604493
C	1.562301	0.712510	-2.247998
H	2.162463	-1.274333	-1.693073
H	3.504886	-0.233689	-2.130049
H	1.523410	0.488373	-3.318936
H	0.551295	0.599877	-1.848055
H	1.874784	1.755471	-2.146502
B	1.499968	0.259742	0.704617
H	1.202909	1.422995	0.533992
H	1.728894	0.050592	1.880287
H	0.636093	-0.479462	0.295491

[2]TS1a01

B3LYP-D3BJ/BS1 G_{corr} in *t*-BuCN:
0.355029 a.u.
B3LYP-D3BJ/BS1 SCF energy in *t*-BuCN:
-3570.634228 a.u.
B3LYP-D3BJ/BS2 SCF energy in *t*-BuCN:
-3571.096962 a.u.
B3LYP-D3BJ/BS2 free energy in *t*-BuCN:
-3570.7389214 a.u.

C	-6.198126	-1.955579	-0.136291
C	-4.909709	-1.947081	0.400840
C	-3.868925	-1.260143	-0.239854
C	-4.148628	-0.582233	-1.434140
C	-5.436044	-0.589019	-1.975396
C	-6.465665	-1.275034	-1.327192
H	-6.992268	-2.494769	0.372657
H	-4.705191	-2.479290	1.326992
H	-3.349354	-0.048473	-1.941106
H	-5.633822	-0.060552	-2.903886
H	-7.467175	-1.282576	-1.747503
C	-2.488249	-1.212968	0.367890
C	-2.367229	-0.141379	1.466538
H	-1.741517	-0.995098	-0.403816
H	-2.259540	-2.190071	0.810223
C	-0.944161	0.135089	1.881896
H	-2.765194	0.802923	1.081807
H	-2.979737	-0.408415	2.334581
H	-0.211718	0.146258	1.073054
H	-0.803288	0.858701	2.674829
Cl	-0.276101	-1.560955	2.871238
N	0.000979	3.507482	-0.084325
C	1.357902	3.876210	0.472731
C	2.097069	2.733675	1.154810
H	1.188990	4.683402	1.184094

H	1.949481	4.270344	-0.360508
H	2.990806	3.153301	1.631634
H	1.470430	2.301289	1.942746
H	2.419790	1.955003	0.452604
C	0.201657	2.417133	-1.125850
C	-1.061343	1.942391	-1.827272
H	0.653912	1.550633	-0.641038
H	0.913781	2.820001	-1.855822
H	-0.776773	1.082219	-2.439391
H	-1.811687	1.601876	-1.111488
H	-1.512912	2.692699	-2.482009
C	-0.567722	4.726828	-0.792592
C	-0.770245	5.953154	0.085378
H	-1.526365	4.415334	-1.204498
H	0.106022	4.954573	-1.625912
H	-1.298674	6.708091	-0.505373
H	-1.381798	5.720857	0.961678
H	0.170329	6.396239	0.420709
B	-0.976243	3.047974	1.074390
H	-0.682641	3.472924	2.156264
H	-2.121466	2.978899	0.734768
P	2.328791	-0.909058	-0.972438
O	3.329384	0.225422	-0.521217
O	2.504207	-1.243527	-2.474730
O	2.746588	-2.195919	-0.100120
O	0.855797	-0.555895	-0.544902
K	2.611611	-0.443543	1.928187
K	4.994452	-1.734867	-1.527936
K	0.326699	-3.031768	0.039555

^{2}TS1a02

B3LYP-D3BJ/BS1 G_{corr} in *t*-BuCN:
0.355134 a.u.
B3LYP-D3BJ/BS1 SCF energy in *t*-BuCN:
-3570.634045 a.u.
B3LYP-D3BJ/BS2 SCF energy in *t*-BuCN:
-3571.103031 a.u.
B3LYP-D3BJ/BS2 free energy in *t*-BuCN:
-3570.7448855 a.u.

C	-6.091651	2.849644	0.131098
C	-4.721589	2.698166	-0.093511
C	-4.072389	1.497711	0.222090
C	-4.825840	0.448300	0.766970
C	-6.195038	0.596112	0.993512
C	-6.832592	1.798412	0.675817
H	-6.579140	3.788344	-0.116938
H	-4.147305	3.518029	-0.518341
H	-4.332315	-0.489067	1.011932
H	-6.763750	-0.225693	1.419493
H	-7.897669	1.915443	0.853610
C	-2.602602	1.311953	-0.067748
C	-2.359299	0.645572	-1.430322
H	-2.159531	0.677850	0.710424
H	-2.106368	2.289530	-0.051942
C	-0.910175	0.331010	-1.711133
H	-2.915136	-0.296150	-1.469173
H	-2.760808	1.275799	-2.231119
H	-0.263802	0.002146	-0.900540
H	-0.736209	-0.211778	-2.635388
Cl	-0.019339	2.119635	-2.160641
N	-0.430598	-3.324705	0.081045
C	0.642661	-3.874284	-0.834272
C	1.398044	-2.824742	-1.640179
H	0.154456	-4.574987	-1.510759
H	1.333242	-4.438156	-0.199101
H	2.226326	-3.327246	-2.152870
H	0.749435	-2.376475	-2.398550
H	1.794832	-2.030619	-1.000985
C	0.274669	-2.534615	1.178143
C	-0.629878	-1.862831	2.200282
H	0.878849	-1.755593	0.704571
H	0.943441	-3.243609	1.676042
H	0.022613	-1.310495	2.885574
H	-1.309112	-1.147326	1.731378
H	-1.223129	-2.557314	2.802061
C	-1.133172	-4.500940	0.742729
C	-1.920422	-5.410404	-0.189499
H	-1.810716	-4.071715	1.479706
H	-0.362792	-5.067852	1.276873
H	-2.426360	-6.162626	0.424354
H	-2.682717	-4.855627	-0.742443
H	-1.287176	-5.941675	-0.904006
B	-1.472549	-2.450194	-0.721221
H	-1.633511	-2.798402	-1.856633
H	-2.400441	-2.088954	-0.057202
P	3.089060	0.080373	0.880083
O	2.931923	1.360286	1.816818

O	3.414596	-1.199209	1.660808
O	1.711619	-0.055695	0.078019
O	4.193264	0.446107	-0.209371
K	4.903099	2.622046	0.860725
K	0.531194	1.898346	1.169739
K	2.667234	0.340790	-2.246024

²TS1a03

B3LYP-D3BJ/BS1 G_{corr} in *t*-BuCN:
0.357433 a.u.
B3LYP-D3BJ/BS1 SCF energy in *t*-BuCN:
-3570.644047 a.u.
B3LYP-D3BJ/BS2 SCF energy in *t*-BuCN:
-3571.106651 a.u.
B3LYP-D3BJ/BS2 free energy in *t*-BuCN:
-3570.7462065 a.u.

C	-4.947608	2.901049	0.099629
C	-4.255322	1.832065	0.670527
C	-2.857370	1.851484	0.797040
C	-2.174229	2.991670	0.338646
C	-2.863598	4.063522	-0.237051
C	-4.253867	4.023015	-0.361075
H	-6.030220	2.858889	0.017468
H	-4.803831	0.961383	1.022419
H	-1.093010	3.057550	0.434024
H	-2.309944	4.932056	-0.583440
H	-4.789954	4.857062	-0.804151
C	-2.137105	0.619960	1.303076
C	-0.872744	0.855205	2.146544
H	-1.872759	0.012891	0.418300
H	-2.847395	0.017519	1.881135
C	0.182606	-0.193874	1.934292
H	-0.396317	1.801807	1.870677
H	-1.116897	0.945464	3.210580
H	0.363606	-0.530620	0.907112
H	1.043795	-0.145364	2.588126
Cl	-0.616193	-2.001722	2.691725
N	3.447936	1.369073	0.133566
C	4.226781	0.430166	1.032922
C	3.678332	-0.987018	1.108687
H	4.222409	0.879546	2.025207
H	5.256898	0.415610	0.663378
H	4.216331	-1.518109	1.900095
H	2.617667	-0.967882	1.357952
H	3.824112	-1.530665	0.172188
C	3.246751	0.686393	-1.211892
C	2.876739	1.619490	-2.361836
H	2.463542	-0.067089	-1.070214
H	4.188131	0.183251	-1.454888
H	2.523672	1.003120	-3.195894
H	2.094332	2.335309	-2.093078
H	3.727783	2.198953	-2.726619
C	4.297231	2.617806	-0.075998
C	4.764011	3.317051	1.192524
H	3.689579	3.302153	-0.666553
H	5.159421	2.312567	-0.679205
H	5.276355	4.236899	0.893362
H	3.926058	3.592199	1.836850
H	5.472737	2.719108	1.769697
B	2.055271	1.743762	0.783536
H	2.128544	2.128304	1.912225
H	1.275941	2.233763	0.015910
P	-0.490340	-1.929105	-1.729117
O	-1.778678	-1.081241	-1.330449
O	-0.239931	-1.829936	-3.248059
O	0.734585	-1.269437	-0.939799
O	-0.680346	-3.393221	-1.165396
K	-2.700919	-2.744654	0.331950
K	-0.322342	0.983485	-1.885678
K	1.310587	-3.330377	0.444018

²TS1a04

B3LYP-D3BJ/BS1 G_{corr} in *t*-BuCN:
0.359465 a.u.
B3LYP-D3BJ/BS1 SCF energy in *t*-BuCN:
-3570.641209 a.u.
B3LYP-D3BJ/BS2 SCF energy in *t*-BuCN:
-3571.104275 a.u.
B3LYP-D3BJ/BS2 free energy in *t*-BuCN:
-3570.7417984 a.u.

C	-1.409784	4.642223	1.229745
C	-1.540373	3.469682	1.982521
C	-1.058985	2.241663	1.509307
C	-0.426948	2.209607	0.251061
C	-0.285997	3.383903	-0.496069
C	-0.781208	4.603691	-0.016097

H	-1.797532	5.580276	1.617984
H	-2.035309	3.507434	2.950460
H	-0.106782	1.254062	-0.163985
H	0.197308	3.345293	-1.468811
H	-0.675925	5.509206	-0.607370
C	-1.274602	0.968357	2.293923
C	0.018917	0.284097	2.798851
H	-1.805639	0.286548	1.615595
H	-1.921046	1.188380	3.150346
C	0.497640	-0.856514	1.939231
H	0.839570	1.010628	2.845209
H	-0.114967	-0.075131	3.824929
H	0.314159	-0.789275	0.860098
H	1.426506	-1.319975	2.246273
Cl	-0.755435	-2.483296	2.351204
N	3.819871	0.085653	-0.279984
C	4.363229	-1.323202	-0.206877
C	3.305952	-2.416472	-0.222040
H	4.940284	-1.387410	0.714399
H	5.046172	-1.449471	-1.052976
H	3.798485	-3.374740	-0.030696
H	2.567732	-2.244189	0.564673
H	2.813909	-2.477811	-1.196043
C	2.889779	0.178308	-1.483751
C	2.547338	1.592232	-1.930461
H	1.959208	-0.332428	-1.223213
H	3.384446	-0.358518	-2.301308
H	1.729285	1.514270	-2.653027
H	2.195695	2.204409	-1.099354
H	3.379561	2.103983	-2.420329
C	4.996364	1.024328	-0.507089
C	6.107294	0.939292	0.529766
H	4.581667	2.031068	-0.512447
H	5.388523	0.807106	-1.507083
H	6.826822	1.735628	0.314951
H	5.725013	1.093375	1.541936
H	6.647236	-0.009855	0.497297
B	3.057101	0.470654	1.054950
H	3.580865	0.053332	2.048192
H	2.535102	1.544252	1.021802
P	-1.654676	-0.837931	-1.466523
O	-0.124858	-0.790309	-1.069289
O	-2.522834	-0.141408	-0.330569
O	-1.920786	-0.086602	-2.803585
O	-2.082918	-2.364843	-1.501308
K	0.137662	-3.297645	-0.664380
K	-2.981766	1.955415	-1.653541
K	-3.485525	-2.220936	0.672706

[2]TS1a05

B3LYP-D3BJ/BS1 G_{corr} in *t*-BuCN:
0.356600 a.u.
B3LYP-D3BJ/BS1 SCF energy in *t*-BuCN:
-3570.630683 a.u.
B3LYP-D3BJ/BS2 SCF energy in *t*-BuCN:
-3571.097834 a.u.
B3LYP-D3BJ/BS2 free energy in *t*-BuCN:
-3570.7382216 a.u.

P	-1.284515	2.247034	1.010821
O	-2.305961	2.058043	2.175713
O	-1.997124	2.645963	-0.360164
O	-0.213065	3.345401	1.325222
O	-0.588650	0.834968	0.753606
K	-1.532619	-0.290296	2.825199
K	-0.686557	4.803091	-0.645303
K	-1.278285	0.583706	-1.680949
N	3.846863	-0.118508	-0.616818
C	4.898750	-0.026899	-1.711907
C	4.446471	0.692328	-2.974306
H	5.172576	-1.051631	-1.958045
H	5.772182	0.468966	-1.275132
H	5.229897	0.573893	-3.729218
H	3.525088	0.257787	-3.373354
H	4.293524	1.762997	-2.821023
C	3.440113	1.294902	-0.227227
C	2.473321	1.403062	0.939536
H	2.981929	1.728041	-1.115967
H	4.371559	1.835165	-0.025645
H	2.150484	2.444720	1.034195
H	1.532523	0.863355	0.783633
H	2.928572	1.106402	1.889570
C	4.491657	-0.768794	0.595023
C	5.024313	-2.177973	0.384242
H	3.731220	-0.783539	1.373053
H	5.295834	-0.098215	0.916573
H	5.380470	-2.544752	1.352112
H	4.243479	-2.856524	0.034334

H	5.865668	-2.217802	-0.311405
B	2.604637	-0.947768	-1.129080
H	2.883219	-1.967313	-1.686835
H	1.670531	-0.281167	-1.463126
C	-3.141940	-2.221203	-3.122718
C	-2.197150	-2.841128	-2.297939
C	-2.305611	-2.772566	-0.898737
C	-3.397152	-2.082172	-0.348174
C	-4.342148	-1.456281	-1.167567
C	-4.213041	-1.516970	-2.559438
H	-3.039725	-2.283894	-4.202563
H	-1.357085	-3.370337	-2.741234
H	-3.503264	-2.025521	0.732775
H	-5.172674	-0.918466	-0.719566
H	-4.942689	-1.028156	-3.198140
C	-1.182989	-3.264334	-0.013414
C	-0.104578	-2.163283	0.014828
H	-0.759312	-4.199645	-0.396626
H	-1.554824	-3.456752	0.996415
C	1.104317	-2.359457	0.890635
H	0.267459	-2.060017	-1.010937
H	-0.546496	-1.198073	0.283954
H	1.731650	-3.226574	0.709319
H	1.640473	-1.462987	1.163580
Cl	0.402845	-2.852412	2.818122

[2]TS1a06

B3LYP-D3BJ/BS1 G_{corr} in *t*-BuCN:
0.360434 a.u.
B3LYP-D3BJ/BS1 SCF energy in *t*-BuCN:
-3570.646119 a.u.
B3LYP-D3BJ/BS2 SCF energy in *t*-BuCN:
-3571.109055 a.u.
B3LYP-D3BJ/BS2 free energy in *t*-BuCN:
-3570.7456089 a.u.

C	-4.584939	-1.843083	-0.701944
C	-3.333798	-2.265775	-1.166270
C	-2.266606	-2.476153	-0.277489
C	-2.495235	-2.263008	1.091488
C	-3.742439	-1.842440	1.563893
C	-4.794365	-1.624537	0.665829
H	-5.395774	-1.685090	-1.407878
H	-3.188800	-2.428449	-2.229963
H	-1.680025	-2.434445	1.790192
H	-3.894835	-1.684826	2.628812
H	-5.764973	-1.296059	1.026259
C	-0.914142	-2.967773	-0.749192
C	-0.322085	-2.286006	-1.994012
H	-0.992969	-4.041095	-0.970608
H	-0.199715	-2.874981	0.073771
C	0.048952	-0.820013	-1.815778
H	0.588528	-2.827812	-2.267312
H	-1.000081	-2.401433	-2.845287
H	-0.092608	-0.366455	-0.825380
H	0.983379	-0.539897	-2.284038
Cl	-1.203060	0.304172	-2.916458
N	3.707895	-0.908559	0.184382
C	4.403337	0.087725	-0.719926
C	3.481004	0.954277	-1.567400
H	5.069063	-0.485846	-1.364475
H	5.021784	0.713961	-0.068375
H	4.100343	1.715803	-2.056745
H	2.994958	0.366031	-2.349748
H	2.719581	1.454289	-0.958835
C	3.030575	-0.122777	1.298498
C	2.109688	-0.932172	2.203474
H	2.438397	0.684118	0.850049
H	3.844719	0.322412	1.881968
H	1.930443	-0.318076	3.093938
H	1.158502	-1.115001	1.700352
H	2.534359	-1.877633	2.550802
C	4.788399	-1.766440	0.831806
C	5.501479	-2.740528	-0.094447
H	4.298472	-2.323487	1.629588
H	5.504785	-1.076299	1.290806
H	6.233630	-3.294869	0.501975
H	4.805474	-3.459209	-0.533308
H	6.044048	-2.243504	-0.902535
B	2.690292	-1.815259	-0.618757
H	3.027397	-2.017695	-1.752813
H	2.240028	-2.710097	0.039863
P	-0.125151	2.181486	0.954533
O	-0.154321	2.601614	2.456051
O	-1.137708	3.022097	0.067374
O	1.300736	2.320153	0.313400
O	-0.605130	0.659493	0.868467
K	-0.832015	0.337013	3.355351

K	0.436950	2.970823	-1.982662
K	-2.820362	1.135006	-0.332145

[2]TS1a07

B3LYP-D3BJ/BS1 G_{corr} in *t*-BuCN:
0.357861 a.u.
B3LYP-D3BJ/BS1 SCF energy in *t*-BuCN:
-3570.642221 a.u.
B3LYP-D3BJ/BS2 SCF energy in *t*-BuCN:
-3571.102273 a.u.
B3LYP-D3BJ/BS2 free energy in *t*-BuCN:
-3570.7414001 a.u.

C	-5.030698	1.514709	0.877896
C	-3.979187	0.759713	1.405339
C	-2.657036	1.244584	1.414365
C	-2.439037	2.538791	0.912666
C	-3.487034	3.297050	0.376705
C	-4.785062	2.784994	0.344334
H	-6.040204	1.111872	0.886225
H	-4.178428	-0.228421	1.814924
H	-1.446376	2.980977	0.949620
H	-3.283743	4.292201	-0.008977
H	-5.597959	3.372880	-0.070920
C	-1.543978	0.309833	1.834723
C	-0.251751	0.938054	2.380756
H	-1.292852	-0.312226	0.961389
H	-1.936282	-0.390611	2.577456
C	0.931905	0.030463	2.189892
H	-0.000205	1.859243	1.840234
H	-0.362598	1.229846	3.430772
H	0.997584	-0.411997	1.198771
H	1.845863	0.262149	2.715256
Cl	0.502402	-1.766594	3.314908
N	3.786898	0.938645	-0.261452
C	4.515310	-0.169946	0.476805
C	3.731248	-1.452996	0.713450
H	4.829960	0.248840	1.432335
H	5.410589	-0.392076	-0.112478
H	4.399444	-2.150501	1.229235
H	2.864812	-1.289346	1.350729
H	3.410212	-1.924857	-0.217276
C	3.159373	0.343838	-1.514765
C	2.796332	1.353949	-2.600422
H	2.262895	-0.202392	-1.195526
H	3.886714	-0.364643	-1.923485
H	2.137743	0.852860	-3.318491
H	2.285244	2.237648	-2.206692
H	3.666576	1.711991	-3.155141
C	4.837159	1.962000	-0.689311
C	5.732490	2.499513	0.417813
H	4.289554	2.784652	-1.147582
H	5.444127	1.478395	-1.462334
H	6.360669	3.282449	-0.018889
H	5.155086	2.945467	1.230324
H	6.397066	1.738111	0.831784
B	2.698313	1.637632	0.649012
H	3.121570	2.034449	1.692681
H	1.888977	2.278126	0.039000
P	-0.741016	-1.585470	-1.570784
O	0.354135	-0.817470	-0.701117
O	-0.060952	-2.773251	-2.292629
O	-1.829845	-2.122487	-0.551364
O	-1.434242	-0.523989	-2.519693
K	-0.267553	1.511424	-1.462466
K	0.387725	-3.213165	0.512551
K	-3.770407	-0.814541	-1.485484

[2]TS1a08

B3LYP-D3BJ/BS1 G_{corr} in *t*-BuCN:
0.355872 a.u.
B3LYP-D3BJ/BS1 SCF energy in *t*-BuCN:
-3570.626913 a.u.
B3LYP-D3BJ/BS2 SCF energy in *t*-BuCN:
-3571.091611 a.u.
B3LYP-D3BJ/BS2 free energy in *t*-BuCN:
-3570.7327274 a.u.

C	-0.160927	2.895071	1.613105
C	0.154239	1.805298	0.792941
C	-0.394138	1.699618	-0.498086
C	-1.246456	2.721242	-0.942774
C	-1.557891	3.811466	-0.126423
C	-1.020739	3.900539	1.160295
H	0.271056	2.957692	2.609423
H	0.875514	1.059417	1.128986
H	-1.680142	2.654606	-1.936998
H	-2.227764	4.584720	-0.492483

H	-1.268695	4.741903	1.801164
C	-0.041019	0.547145	-1.413686
C	0.169425	-0.814794	-0.715749
H	0.936201	0.761594	-1.869490
H	-0.793033	0.469082	-2.204355
C	-0.992337	-1.336881	0.084437
H	0.415778	-1.557636	-1.484568
H	1.046960	-0.691862	-0.071453
H	-1.352425	-0.711157	0.891250
H	-1.762498	-1.902951	-0.419026
Cl	-0.221829	-2.930401	1.267398
N	-4.699829	-0.469720	-0.124408
C	-5.382797	-1.820886	-0.189609
C	-4.511078	-3.014874	0.169513
H	-5.740167	-1.933441	-1.211889
H	-6.251209	-1.767243	0.474842
H	-5.106186	-3.919805	0.010492
H	-3.629382	-3.079662	-0.471667
H	-4.188718	-3.013912	1.212859
C	-4.219089	-0.255291	1.302035
C	-3.571565	1.092663	1.579464
H	-3.503166	-1.048893	1.504428
H	-5.089413	-0.407717	1.949279
H	-3.178550	1.068142	2.600661
H	-2.738570	1.284435	0.902940
H	-4.276325	1.924800	1.518675
C	-5.741070	0.600359	-0.424190
C	-6.400617	0.488614	-1.790714
H	-5.221559	1.554134	-0.350221
H	-6.488975	0.550256	0.374387
H	-7.011994	1.383474	-1.943032
H	-5.656377	0.448296	-2.591216
H	-7.058456	-0.379129	-1.876736
B	-3.521272	-0.363327	-1.178522
H	-3.544330	-1.197007	-2.034239
H	-3.170282	0.753257	-1.389748
P	3.874541	0.001126	-0.062000
O	5.111691	0.897132	0.209346
O	4.192103	-1.541455	-0.211680
O	2.830931	0.180920	1.125303
O	3.157907	0.498580	-1.392137
K	3.079191	2.734318	0.074649
K	2.769055	-2.250593	1.823035
K	3.136287	-1.740458	-2.525821

[2]TS1a09

B3LYP-D3BJ/BS1 G_{corr} in *t*-BuCN:
0.359929 a.u.
B3LYP-D3BJ/BS1 SCF energy in *t*-BuCN:
-3570.649442 a.u.
B3LYP-D3BJ/BS2 SCF energy in *t*-BuCN:
-3571.11201 a.u.
B3LYP-D3BJ/BS2 free energy in *t*-BuCN:
-3570.7490686 a.u.

C	1.791073	3.404507	0.388109
C	1.303151	2.842899	-0.794709
C	2.123996	2.048073	-1.607467
C	3.445833	1.820610	-1.189785
C	3.935790	2.362409	0.003016
C	3.109110	3.165784	0.795804
H	1.138768	4.028595	0.994448
H	0.274179	3.028872	-1.083462
H	4.105065	1.224649	-1.819329
H	4.962336	2.170143	0.302830
H	3.490002	3.608819	1.712619
C	1.645591	1.486727	-2.931783
C	0.195619	0.983896	-2.981763
H	2.320131	0.681060	-3.239302
H	1.744958	2.270334	-3.694946
C	-0.153305	-0.160821	-2.061702
H	-0.486981	1.802897	-2.731636
H	-0.039308	0.703488	-4.014171
H	0.153648	-0.127422	-1.007399
H	-1.133390	-0.587019	-2.222982
Cl	0.979521	-1.771463	-2.712460
N	-3.512032	0.864228	0.064758
C	-4.287067	-0.395813	-0.262166
C	-3.445791	-1.571689	-0.742258
H	-5.017754	-0.126037	-1.024426
H	-4.832853	-0.663706	0.648512
H	-4.100031	-2.450951	-0.784293
H	-3.060974	-1.396010	-1.750046
H	-2.611637	-1.782936	-0.063920
C	-2.724992	0.600151	1.341216
C	-1.736255	1.690518	1.734452
H	-2.166231	-0.336019	1.221546
H	-3.477890	0.463831	2.125910

H	-1.463036	1.498817	2.778818
H	-0.841419	1.622984	1.114239
H	-2.143164	2.704361	1.693321
C	-4.527834	1.955859	0.381096
C	-5.325813	2.480663	-0.803339
H	-3.965779	2.775738	0.826807
H	-5.196197	1.548055	1.147227
H	-6.002763	3.257263	-0.432313
H	-4.676155	2.928351	-1.559031
H	-5.937883	1.711941	-1.281847
B	-2.583935	1.306575	-1.133049
H	-3.043654	1.058575	-2.212054
H	-2.020128	2.344936	-0.940016
P	0.412948	-1.663213	1.681709
O	0.593545	-1.377906	3.203787
O	1.371772	-2.815293	1.158181
O	-1.060712	-2.059587	1.310118
O	0.826802	-0.340554	0.888633
K	1.330068	1.039203	2.931325
K	-0.396542	-3.689513	-0.516218
K	2.894899	-1.298054	-0.241689

[2]TS1b01

B3LYP-D3BJ/BS1 G_{corr} in *t*-BuCN:
0.357156 a.u.
B3LYP-D3BJ/BS1 SCF energy in *t*-BuCN:
-3570.638916 a.u.
B3LYP-D3BJ/BS2 SCF energy in *t*-BuCN:
-3571.099938 a.u.
B3LYP-D3BJ/BS2 free energy in *t*-BuCN:
-3570.7397696 a.u.

P	-0.217264	1.944116	-0.130892
O	0.205154	1.031070	-1.325656
O	-1.078572	3.149725	-0.670488
O	-1.114701	1.085909	0.857028
O	1.024756	2.503035	0.676736
K	0.853502	0.551867	2.385817
K	-2.493749	0.845217	-1.438309
K	0.420188	4.940084	0.283800
N	3.912414	-1.028517	-0.776661
C	5.396089	-0.781279	-1.013311
C	6.200007	-1.987243	-1.477573
H	5.797135	-0.423831	-0.066019
H	5.468624	0.031814	-1.744425
H	7.254328	-1.694299	-1.509636
H	6.103218	-2.826775	-0.784534
H	5.921434	-2.324070	-2.478759
C	3.296145	-1.471866	-2.088089
C	1.790694	-1.695954	-2.055195
H	3.801876	-2.395890	-2.366048
H	3.554668	-0.705746	-2.827315
H	1.493201	-2.047752	-3.050716
H	1.526301	-2.468977	-1.330450
H	1.223595	-0.785275	-1.825364
C	3.289265	0.304752	-0.401087
C	3.799670	0.942584	0.881788
H	2.209499	0.175591	-0.358936
H	3.478758	0.978052	-1.243868
H	4.838147	1.278054	0.816614
H	3.154418	1.812256	1.044123
H	3.722968	0.263615	1.736885
B	3.736459	-2.134454	0.347485
H	4.351063	-1.890422	1.345876
H	3.716324	-3.247242	-0.091971
C	-4.949501	-1.711259	-1.847013
C	-4.105121	-2.319717	-0.912909
C	-4.010049	-1.830744	0.402174
C	-4.786472	-0.715538	0.755518
C	-5.635480	-0.103667	-0.174585
C	-5.718595	-0.598118	-1.481509
H	-5.011054	-2.106281	-2.857258
H	-3.512944	-3.183252	-1.204864
H	-4.720392	-0.321900	1.766434
H	-6.228531	0.757407	0.120821
H	-6.377142	-0.126615	-2.205109
C	-3.051268	-2.457028	1.389011
C	-1.575404	-2.174509	1.021532
H	-3.213207	-3.541547	1.427269
H	-3.257427	-2.065339	2.391370
C	-0.631810	-2.749756	2.051232
H	-1.361113	-2.622634	0.043503
H	-1.419851	-1.090283	0.927539
H	-0.708013	-2.280042	3.034551
H	-0.678430	-3.835126	2.147959
Cl	1.241252	-2.415965	1.459364

[2]TS1b02

B3LYP-D3BJ/BS1 G_{corr} in *t*-BuCN:
0.357522 a.u.
B3LYP-D3BJ/BS1 SCF energy in *t*-BuCN:
-3570.642710 a.u.
B3LYP-D3BJ/BS2 SCF energy in *t*-BuCN:
-3571.107962 a.u.
B3LYP-D3BJ/BS2 free energy in *t*-BuCN:
-3570.7474285 a.u.

P	-0.375550	-2.483335	0.783817
O	1.148225	-2.332239	1.111337
O	-0.607399	-2.529253	-0.800242
O	-0.989239	-3.786311	1.387161
O	-1.123766	-1.180448	1.312102
K	0.976757	-0.081230	2.273582
K	-1.611414	-4.856771	-0.788231
K	-1.941566	-0.364584	-0.945791
N	3.931844	0.428133	-0.723391
C	5.280623	-0.274540	-0.803042
C	5.430545	-1.487839	0.103501
H	6.032761	0.469893	-0.544784
H	5.424582	-0.561420	-1.850650
H	6.456805	-1.855071	0.002908
H	5.264289	-1.231658	1.152468
H	4.756953	-2.302995	-0.169129
C	2.855103	-0.609732	-1.016726
C	1.479538	-0.074165	-1.383467
H	2.737862	-1.238933	-0.133097
H	3.238622	-1.223124	-1.838930
H	0.806461	-0.941668	-1.339722
H	1.134581	0.672798	-0.664467
H	1.447692	0.371126	-2.382617
C	3.882713	1.481503	-1.812947
C	5.006698	2.507865	-1.810326
H	2.927661	1.990544	-1.692803
H	3.870069	0.942774	-2.766190
H	4.797630	3.224578	-2.611159
H	5.055072	3.060694	-0.870423
H	5.985922	2.068128	-2.012639
B	3.770421	1.071323	0.714314
H	4.593241	1.902305	0.961788
H	3.475801	0.270655	1.554121
C	-5.161872	0.686580	0.226111
C	-4.293152	1.619098	0.803334
C	-3.636768	2.577561	0.013614
C	-3.878378	2.584981	-1.370749
C	-4.747015	1.654747	-1.952380
C	-5.388363	0.698170	-1.155468
H	-5.657878	-0.048833	0.853198
H	-4.111752	1.597652	1.874864
H	-3.378667	3.322125	-1.994586
H	-4.924407	1.677686	-3.023948
H	-6.062477	-0.024917	-1.605009
C	-2.603314	3.498272	0.619527
C	-1.201145	2.850330	0.548989
H	-2.851817	3.707238	1.665451
H	-2.586562	4.453967	0.084672
C	-0.117447	3.707282	1.155963
H	-1.238394	1.882032	1.064707
H	-0.956048	2.654050	-0.503036
H	0.044597	4.651107	0.635614
H	-0.223002	3.862187	2.229845
Cl	1.604573	2.729294	0.960492

^{2}TS1b03

B3LYP-D3BJ/BS1 G_{corr} in *t*-BuCN:
0.358051 a.u.
B3LYP-D3BJ/BS1 SCF energy in *t*-BuCN:
-3570.644505 a.u.
B3LYP-D3BJ/BS2 SCF energy in *t*-BuCN:
-3571.10608 a.u.
B3LYP-D3BJ/BS2 free energy in *t*-BuCN:
-3570.7450166 a.u.

P	0.134969	2.626070	-0.642920
O	1.550679	2.003286	-1.014814
O	0.003832	2.554819	0.944903
O	0.106664	4.112619	-1.082548
O	-0.993610	1.715887	-1.274976
K	0.660820	-0.014413	-2.265342
K	2.309887	3.904165	0.652372
K	-2.036222	1.043715	0.965437
N	3.456250	-1.399303	0.633249
C	4.933962	-1.037653	0.700938
C	5.338520	0.179710	-0.118903
H	5.483180	-1.912225	0.354193
H	5.170826	-0.878239	1.759011
H	6.427174	0.276884	-0.058829

H	5.065932	0.069209	-1.171016
H	4.900674	1.105920	0.260544
C	2.661937	-0.156626	1.015394
C	1.206060	-0.370495	1.399696
H	2.677130	0.528413	0.165492
H	3.197221	0.303257	1.853849
H	0.760367	0.635496	1.401021
H	0.681764	-0.984683	0.664068
H	1.085904	-0.836245	2.382681
C	3.184399	-2.473284	1.667801
C	4.032487	-3.733362	1.569977
H	2.132637	-2.733452	1.558763
H	3.324418	-2.003348	2.647022
H	3.682075	-4.425323	2.342703
H	3.922647	-4.227718	0.603100
H	5.093177	-3.547628	1.753732
B	3.114504	-1.899596	-0.830440
H	3.697939	-2.894111	-1.145070
H	3.013664	-1.004403	-1.618491
C	-5.419012	0.767840	-0.124681
C	-4.822296	-0.345742	-0.726087
C	-4.399172	-1.441022	0.045136
C	-4.590882	-1.395423	1.436465
C	-5.187870	-0.284244	2.042347
C	-5.601068	0.803839	1.263186
H	-5.738009	1.606018	-0.737584
H	-4.674813	-0.364124	-1.802883
H	-4.268283	-2.235853	2.046136
H	-5.332572	-0.268523	3.118933
H	-6.064456	1.667015	1.731671
C	-3.655359	-2.591188	-0.592556
C	-2.132954	-2.323271	-0.582892
H	-3.990531	-2.728575	-1.626122
H	-3.860698	-3.520831	-0.051027
C	-1.331609	-3.431411	-1.221615
H	-1.944864	-1.378478	-1.109627
H	-1.803216	-2.191992	0.455717
H	-1.407877	-4.386597	-0.702466
H	-1.507929	-3.548236	-2.290960
Cl	0.593360	-2.944372	-1.079055

[2]TS1b04

B3LYP-D3BJ/BS1 G_{corr} in *t*-BuCN:
0.358636 a.u.

B3LYP-D3BJ/BS1 SCF energy in *t*-BuCN:
-3570.646561 a.u.

B3LYP-D3BJ/BS2 SCF energy in *t*-BuCN:
-3571.108179 a.u.

B3LYP-D3BJ/BS2 free energy in *t*-BuCN:
-3570.7465309 a.u.

N	-4.634539	-0.082266	-0.252796
C	-5.864929	0.267353	-1.085259
C	-6.890874	-0.845075	-1.245548
H	-5.492073	0.564713	-2.064261
H	-6.322069	1.141803	-0.610181
H	-7.661296	-0.484649	-1.934486
H	-6.450156	-1.747408	-1.675283
H	-7.385020	-1.105066	-0.306839
C	-5.116543	-0.566703	1.099056
C	-4.034023	-0.733216	2.154524
H	-5.605779	-1.524222	0.927033
H	-5.868488	0.150694	1.441603
H	-4.494649	-1.175516	3.043063
H	-3.250364	-1.409723	1.807038
H	-3.607306	0.228085	2.454278
C	-3.828093	1.190552	-0.051103
C	-3.601941	2.044235	-1.294068
H	-2.856602	0.907872	0.351483
H	-4.354877	1.782062	0.704106
H	-2.764633	2.708950	-1.063075
H	-3.341098	1.449851	-2.174604
H	-4.472625	2.649249	-1.556496
B	-3.791292	-1.195381	-1.000212
H	-3.322637	-0.807453	-2.032556
H	-4.266663	-2.286074	-0.919131
C	6.123226	-0.557167	0.380362
C	5.032059	-1.349663	0.757813
C	4.070303	-1.754797	-0.181636
C	4.225354	-1.335473	-1.514215
C	5.307922	-0.537962	-1.895868
C	6.264752	-0.146109	-0.950243
H	6.857921	-0.260692	1.123960
H	4.924550	-1.659167	1.794282
H	3.491990	-1.633253	-2.258421
H	5.406925	-0.223303	-2.931046
H	7.109055	0.469244	-1.247339
C	2.900493	-2.618740	0.237848

C	1.518042	-2.008763	-0.098637
H	2.961738	-2.792539	1.317970
H	2.982982	-3.602729	-0.242568
C	0.422316	-2.880401	0.460265
H	1.468743	-0.985948	0.301802
H	1.411725	-1.946585	-1.188563
H	0.365381	-3.880292	0.031228
H	0.385540	-2.925152	1.550551
Cl	-1.421328	-2.106846	-0.039448
P	0.802811	1.961214	0.404174
O	-0.766260	1.745559	0.453206
O	1.268728	3.410176	0.696585
O	1.462894	0.970316	1.466140
O	1.329443	1.514785	-1.023491
K	-0.752231	0.367584	-1.799850
K	-0.671117	-0.046639	2.306671
K	3.641327	1.848163	0.196117

²TS1b05

B3LYP-D3BJ/BS1 G_{corr} in *t*-BuCN:
0.356429 a.u.
B3LYP-D3BJ/BS1 SCF energy in *t*-BuCN:
-3570.640875 a.u.
B3LYP-D3BJ/BS2 SCF energy in *t*-BuCN:
-3571.101408 a.u.
B3LYP-D3BJ/BS2 free energy in *t*-BuCN:
-3570.7419673 a.u.

N	-4.056678	-0.581171	-0.558082
C	-5.085481	-1.593021	-1.032330
C	-6.537818	-1.220944	-0.766867
H	-4.849376	-2.524345	-0.520390
H	-4.919039	-1.737935	-2.104928
H	-7.154924	-2.100280	-0.976816
H	-6.696075	-0.940799	0.278262
H	-6.892241	-0.409894	-1.407013
C	-4.390699	0.749072	-1.212425
C	-3.422264	1.894267	-0.949997
H	-5.384715	1.026392	-0.864141
H	-4.451214	0.545709	-2.286695
H	-3.663907	2.696706	-1.655604
H	-3.578880	2.308375	0.050210
H	-2.364118	1.637031	-1.078809
C	-2.692741	-1.015549	-1.092526
C	-2.336115	-2.473276	-0.837868
H	-1.939176	-0.355746	-0.660046
H	-2.720785	-0.828788	-2.172832
H	-1.278013	-2.606418	-1.090379
H	-2.456752	-2.741388	0.212661
H	-2.910035	-3.168172	-1.456322
B	-4.091549	-0.481512	1.033302
H	-4.664354	-1.386367	1.560677
H	-4.189058	0.634174	1.455083
C	5.311325	-1.030513	-0.473089
C	4.510151	-1.122328	0.672245
C	3.321894	-1.869242	0.673809
C	2.977186	-2.560502	-0.501201
C	3.774559	-2.476029	-1.647465
C	4.936292	-1.694635	-1.643689
H	6.227683	-0.446344	-0.446530
H	4.820848	-0.625451	1.590210
H	2.078576	-3.172490	-0.517747
H	3.491410	-3.024344	-2.542658
H	5.551513	-1.624519	-2.535493
C	2.430427	-1.919310	1.894809
C	1.000406	-1.407204	1.604826
H	2.882919	-1.322445	2.694920
H	2.372687	-2.951912	2.263641
C	0.125560	-1.481741	2.831873
H	1.031839	-0.376415	1.229581
H	0.558876	-2.030082	0.818220
H	0.048359	-2.472761	3.280732
H	0.332488	-0.721209	3.585924
Cl	-1.782109	-1.091008	2.238904
P	0.643521	2.170413	-0.376110
O	1.863853	2.115099	-1.386843
O	-0.195344	3.471483	-0.433679
O	-0.297560	0.928688	-0.680593
O	1.218073	1.971882	1.094650
K	3.636327	1.959535	0.440796
K	1.224006	-0.212617	-2.294848
K	-1.388505	1.864395	1.630247

²TS1b06

B3LYP-D3BJ/BS1 G_{corr} in *t*-BuCN:
0.358939 a.u.
B3LYP-D3BJ/BS1 SCF energy in *t*-BuCN:
-3570.644851 a.u.

B3LYP-D3BJ/BS2 SCF energy in *t*-BuCN:
-3571.109644 a.u.
B3LYP-D3BJ/BS2 free energy in *t*-BuCN:
-3570.7476928 a.u.

N	-4.534221	0.226493	-0.251036
C	-5.870502	0.858051	0.109443
C	-6.768278	1.198923	-1.071563
H	-5.634189	1.767765	0.658063
H	-6.376472	0.165148	0.789337
H	-7.594727	1.812002	-0.698717
H	-6.234278	1.780594	-1.828292
H	-7.201342	0.315674	-1.545966
C	-4.825477	-1.022345	-1.068852
C	-3.621296	-1.845960	-1.510783
H	-5.380293	-0.696786	-1.947133
H	-5.493264	-1.630691	-0.451259
H	-3.989737	-2.831426	-1.814404
H	-3.158312	-1.403989	-2.397990
H	-2.863276	-1.991870	-0.732390
C	-3.868467	-0.230375	1.042811
C	-3.740860	0.843672	2.115209
H	-2.886803	-0.632030	0.779806
H	-4.482480	-1.053941	1.423870
H	-3.215693	0.400722	2.970101
H	-3.179186	1.711742	1.761997
H	-4.704849	1.183890	2.499160
B	-3.649800	1.277088	-1.052731
H	-4.027736	2.402087	-0.950540
H	-3.207459	0.867903	-2.085196
C	5.596970	0.574991	-1.765309
C	4.549000	1.436156	-1.425394
C	4.247853	1.709729	-0.078978
C	5.025700	1.097759	0.917272
C	6.079720	0.238751	0.581696
C	6.367563	-0.027783	-0.761858
H	5.815769	0.377514	-2.810905
H	3.960355	1.904202	-2.210247
H	4.802583	1.296383	1.962398
H	6.671726	-0.221652	1.367667
H	7.184373	-0.693409	-1.024828
C	3.094017	2.615354	0.285231
C	1.712521	2.015222	-0.075907
H	3.201865	3.576729	-0.231061
H	3.122266	2.822299	1.360226
C	0.609851	2.954623	0.344957
H	1.667982	1.855501	-1.160650
H	1.611368	1.028620	0.398363
H	0.535035	3.117484	1.421528
H	0.604824	3.903060	-0.189892
Cl	-1.234315	2.175852	-0.142537
P	0.367454	-2.289729	0.511762
O	-1.038321	-1.538699	0.533883
O	0.275768	-3.739017	1.011461
O	0.923526	-2.196992	-0.978254
O	1.355441	-1.402245	1.405059
K	-0.386433	0.216239	2.268878
K	-0.676757	-0.400151	-1.774507
K	3.286918	-1.670454	-0.239134

[2]TS1b07
B3LYP-D3BJ/BS1 G_{corr} in *t*-BuCN:
0.357515 a.u.
B3LYP-D3BJ/BS1 SCF energy in *t*-BuCN:
-3570.633993 a.u.
B3LYP-D3BJ/BS2 SCF energy in *t*-BuCN:
-3571.102059 a.u.
B3LYP-D3BJ/BS2 free energy in *t*-BuCN:
-3570.7415320 a.u.

P	-1.592922	-2.209132	-0.663401
O	-1.831783	-3.645243	-1.224121
O	-0.261610	-2.124392	0.209000
O	-1.481802	-1.135785	-1.802513
O	-2.808030	-1.858411	0.309395
K	-4.093653	-3.979227	-0.185706
K	0.870431	-0.403321	-1.327187
K	-1.409209	-1.089425	2.262590
N	-2.744348	2.832739	-0.318849
C	-3.977212	3.705532	-0.134761
C	-3.765167	5.193083	-0.375341
H	-4.306101	3.549229	0.891303
H	-4.748543	3.312959	-0.806826
H	-4.682384	5.714851	-0.084332
H	-2.945868	5.584503	0.233626
H	-3.569878	5.431067	-1.423506
C	-2.264288	2.998713	-1.744045
C	-1.087019	2.113698	-2.125761

H -1.989677 4.046276 -1.857900
H -3.121941 2.794286 -2.393639
H -0.787294 2.375568 -3.146658
H -0.239200 2.312777 -1.462257
H -1.344017 1.048508 -2.098904
C -3.179014 1.387247 -0.137899
C -3.781066 1.041561 1.214741
H -2.324106 0.743123 -0.334325
H -3.911716 1.186572 -0.927302
H -3.895596 -0.046829 1.202300
H -3.122598 1.334010 2.038823
H -4.757888 1.501119 1.386082
B -1.620557 3.265078 0.716944
H -2.028648 3.365925 1.838303
H -0.851072 4.069916 0.275150
C 7.538014 0.771222 0.161909
C 6.444783 0.407128 0.950635
C 5.379334 -0.321670 0.405839
C 5.433535 -0.678404 -0.949063
C 6.524598 -0.316480 -1.741335
C 7.580789 0.411473 -1.187478
H 8.357543 1.332813 0.601563
H 6.415828 0.688433 2.000403
H 4.615924 -1.251596 -1.381305
H 6.552594 -0.606617 -2.788027
H 8.432130 0.691746 -1.800923
C 4.161867 -0.657782 1.232937
C 3.112325 0.473482 1.165511
H 4.442016 -0.828874 2.277868
H 3.708544 -1.584294 0.860923
C 1.812395 0.076601 1.830027
H 3.513447 1.381556 1.627621
H 2.947554 0.726581 0.109731
H 1.402082 -0.864194 1.452048
H 1.873218 0.068451 2.918718
Cl 0.414791 1.373544 1.400933

[2]TS1b08

B3LYP-D3BJ/BS1 G_{corr} in *t*-BuCN:
0.358547 a.u.
B3LYP-D3BJ/BS1 SCF energy in *t*-BuCN:
-3570.640131 a.u.
B3LYP-D3BJ/BS2 SCF energy in *t*-BuCN:
-3571.108604 a.u.
B3LYP-D3BJ/BS2 free energy in *t*-BuCN:
-3570.7470449 a.u.

N -4.030078 -1.024814 -0.331047
C -4.983525 0.114517 -0.664051
C -5.203402 0.369866 -2.147637
H -4.569484 1.005886 -0.196219
H -5.931116 -0.106914 -0.161644
H -5.812101 1.274459 -2.243802
H -4.259247 0.535638 -2.674084
H -5.740125 -0.443184 -2.641039
C -4.646716 -2.303695 -0.868479
C -3.908818 -3.584523 -0.513517
H -4.677515 -2.192146 -1.951303
H -5.674577 -2.335217 -0.494648
H -4.409363 -4.408525 -1.031954
H -2.868291 -3.553360 -0.841327
H -3.932857 -3.805108 0.556170
C -3.940495 -1.133142 1.182610
C -3.558782 0.150360 1.912462
H -3.198694 -1.904342 1.381104
H -4.914905 -1.487966 1.532457
H -3.344796 -0.112660 2.954518
H -2.677164 0.640274 1.489210
H -4.372623 0.879387 1.936505
B -2.604433 -0.772478 -0.964354
H -2.094449 0.248761 -0.610243
H -2.474675 -1.175698 -2.083080
C 5.282559 0.937790 -0.710340
C 4.369712 0.115921 -0.044632
C 4.649022 -1.252737 0.128965
C 5.849730 -1.765245 -0.378053
C 6.762333 -0.938833 -1.040359
C 6.481068 0.417954 -1.209287
H 5.053301 1.992801 -0.839815
H 3.428971 0.542775 0.314431
H 6.072578 -2.822305 -0.252934
H 7.689109 -1.356708 -1.424210
H 7.186802 1.063460 -1.724725
C 3.656514 -2.161370 0.827211
C 2.252197 -2.029709 0.208216
H 3.591007 -1.897447 1.891643
H 4.006493 -3.198331 0.778557
C 1.205136 -2.909703 0.843289

H	1.947864	-0.983414	0.319389
H	2.313656	-2.259499	-0.864133
H	1.223305	-3.957310	0.546063
H	1.130371	-2.815544	1.928494
Cl	-0.570478	-2.228445	0.166244
P	0.607024	2.498156	0.472746
O	1.380981	1.106484	0.609141
O	1.471036	3.690640	0.904360
O	0.122887	2.602807	-1.041928
O	-0.721127	2.351482	1.352824
K	-0.161823	0.076068	2.337272
K	0.660011	0.217457	-1.713750
K	-2.313737	2.964556	-0.524846

[2]TS1b09

B3LYP-D3BJ/BS1 G_{corr} in *t*-BuCN:
0.356358 a.u.
B3LYP-D3BJ/BS1 SCF energy in *t*-BuCN:
-3570.642473 a.u.
B3LYP-D3BJ/BS2 SCF energy in *t*-BuCN:
-3571.105970 a.u.
B3LYP-D3BJ/BS2 free energy in *t*-BuCN:
-3570.746600 a.u.

N	4.910986	-0.649135	0.261809
C	6.114354	0.283153	0.189240
C	6.260004	1.242180	1.361304
H	5.999339	0.852997	-0.731021
H	7.000867	-0.352487	0.094219
H	7.085900	1.923465	1.134217
H	5.357180	1.842244	1.503870
H	6.498641	0.735653	2.299123
C	5.057460	-1.507052	1.503540
C	3.969294	-2.550477	1.708399
H	5.059582	-0.820867	2.348819
H	6.041473	-1.982757	1.443951
H	4.133862	-3.014864	2.686009
H	2.974112	-2.101091	1.706159
H	3.992168	-3.343979	0.958198
C	4.959648	-1.562686	-0.951177
C	4.866823	-0.870179	-2.302353
H	4.123491	-2.249827	-0.846818
H	5.892318	-2.131234	-0.873876
H	4.851755	-1.649999	-3.070582
H	3.945439	-0.292090	-2.395138
H	5.718392	-0.219055	-2.511549
B	3.579711	0.202289	0.312889
H	3.549495	1.080946	-0.501748
H	3.187371	0.403517	1.428080
C	-5.076427	-0.915603	1.565957
C	-3.918490	-1.048247	0.786395
C	-3.902418	-1.920019	-0.315126
C	-5.063212	-2.655405	-0.603348
C	-6.214699	-2.521420	0.173093
C	-6.226804	-1.647354	1.264478
H	-5.072059	-0.233208	2.413078
H	-3.048056	-0.429206	1.018926
H	-5.061068	-3.342157	-1.446949
H	-7.100010	-3.102926	-0.069411
H	-7.120175	-1.544324	1.873976
C	-2.686820	-2.066890	-1.212296
C	-1.357374	-1.596194	-0.596937
H	-2.859045	-1.496763	-2.137018
H	-2.608705	-3.116571	-1.523034
C	-0.201516	-1.939204	-1.500690
H	-1.394268	-0.511856	-0.438007
H	-1.217053	-2.085121	0.376696
H	0.019268	-3.002992	-1.590612
H	-0.243636	-1.460786	-2.480070
Cl	1.495922	-1.179693	-0.704481
P	-1.280330	2.347480	0.206671
O	0.252466	2.581303	0.508081
O	-2.168696	3.618334	0.308587
O	-1.413237	1.739932	-1.259594
O	-1.830738	1.243815	1.208608
K	0.460005	0.395482	1.908870
K	1.070136	1.728972	-1.780618
K	-3.972513	1.781111	-0.535558

[2]TS1a'

B3LYP-D3BJ/BS1 G_{corr} in *t*-BuCN:
0.352581 a.u.
B3LYP-D3BJ/BS1 SCF energy in *t*-BuCN:
-1128.330108 a.u.
B3LYP-D3BJ/BS2 SCF energy in *t*-BuCN:
-1128.522215 a.u.
B3LYP-D3BJ/BS2 free energy in *t*-BuCN:
-1128.1666225 a.u.

C	4.274148	-2.389768	0.127909
C	3.137070	-1.657502	-0.218017
C	3.185403	-0.260119	-0.323070
C	4.402215	0.387163	-0.069039
C	5.542374	-0.340772	0.277755
C	5.481935	-1.732833	0.377426
H	4.218437	-3.472470	0.200201
H	2.198875	-2.171999	-0.411763
H	4.453498	1.470444	-0.146812
H	6.477799	0.178481	0.467314
H	6.368419	-2.300983	0.644309
C	1.940971	0.529914	-0.648056
C	1.123196	0.857752	0.611220
H	1.306535	-0.042546	-1.334945
H	2.208679	1.463897	-1.152070
C	-0.147190	1.615699	0.310550
H	0.867452	-0.074853	1.125112
H	1.732768	1.437217	1.313486
H	-0.630487	1.436994	-0.638924
H	-0.820203	1.807781	1.135059
Cl	0.456661	3.597105	-0.052062
N	-3.120213	-0.650092	-0.014039
C	-4.010979	-0.310703	1.163895
C	-3.746258	1.038550	1.814273
H	-3.858706	-1.098673	1.899522
H	-5.043689	-0.368829	0.805133
H	-4.398652	1.120289	2.689519
H	-2.712661	1.122198	2.156882
H	-3.970727	1.881630	1.157367
C	-3.324224	0.415120	-1.078495
C	-2.609941	0.172088	-2.400002
H	-2.975141	1.349833	-0.643856
H	-4.404609	0.494239	-1.240174
H	-2.740669	1.067025	-3.016344
H	-1.538360	0.013246	-2.261663
H	-3.022011	-0.672837	-2.956142
C	-3.585139	-1.980521	-0.588783
C	-3.530112	-3.151675	0.380879
H	-2.936117	-2.185122	-1.438499
H	-4.604610	-1.829974	-0.960372
H	-3.755298	-4.063685	-0.180593
H	-2.533742	-3.261587	0.818192
H	-4.262058	-3.071521	1.187624
B	-1.601372	-0.753884	0.426940
H	-1.440506	-0.948524	1.595085
H	-0.899919	-1.264749	-0.394697

²TS1b'

B3LYP-D3BJ/BS1 G_{corr} in *t*-BuCN:
0.350965 a.u.
B3LYP-D3BJ/BS1 SCF energy in *t*-BuCN:
-1128.330079 a.u.
B3LYP-D3BJ/BS2 SCF energy in *t*-BuCN:
-1128.520689 a.u.
B3LYP-D3BJ/BS2 free energy in *t*-BuCN:
-1128.166712 a.u.

C	5.332782	-1.303721	-1.379507
C	4.505432	-0.182742	-1.288519
C	4.247865	0.423108	-0.050764
C	4.838493	-0.124964	1.096542
C	5.666527	-1.245727	1.010757
C	5.916259	-1.839880	-0.228833
H	5.524144	-1.756641	-2.348433
H	4.055145	0.232043	-2.187132
H	4.648620	0.335140	2.063260
H	6.118910	-1.653179	1.910673
H	6.562407	-2.710238	-0.297718
C	3.307629	1.600823	0.052759
C	1.844705	1.148887	0.265934
H	3.365822	2.205438	-0.859575
H	3.608229	2.244144	0.887634
C	0.898169	2.319677	0.381380
H	1.543533	0.508022	-0.571371
H	1.789122	0.534934	1.172543
H	1.082410	2.947567	1.254049
H	0.838632	2.925698	-0.523592
Cl	-0.954496	1.643081	0.641216
N	-3.693393	-0.511921	-0.096182
C	-4.293067	0.091429	-1.349147
C	-3.391689	1.058054	-2.102913
H	-5.194417	0.613766	-1.032845
H	-4.588202	-0.742354	-1.994827
H	-3.987328	1.521131	-2.896180
H	-3.015741	1.847226	-1.449034
H	-2.540785	0.563311	-2.576633
C	-2.433730	-1.261689	-0.492258

C	-1.729742	-2.003822	0.633520
H	-1.755707	-0.517381	-0.901918
H	-2.719770	-1.955203	-1.290724
H	-0.798376	-2.412327	0.228195
H	-1.471579	-1.332162	1.454016
H	-2.311715	-2.841075	1.025744
C	-4.683599	-1.527927	0.454208
C	-6.049381	-0.965242	0.819806
H	-4.218760	-1.952002	1.342449
H	-4.780872	-2.320477	-0.296006
H	-6.615556	-1.756520	1.321059
H	-5.962674	-0.122187	1.510571
H	-6.626240	-0.648487	-0.052079
B	-3.406173	0.607739	0.998847
H	-4.080012	1.587499	0.860463
H	-3.218120	0.149102	2.087969

[2]TS1c'

B3LYP-D3BJ/BS1 G_{corr} in *t*-BuCN:
0.351263 a.u.
B3LYP-D3BJ/BS1 SCF energy in *t*-BuCN:
-1128.320114 a.u.
B3LYP-D3BJ/BS2 SCF energy in *t*-BuCN:
-1128.509481 a.u.
B3LYP-D3BJ/BS2 free energy in *t*-BuCN:
-1128.155206 a.u.

N	2.996672	-0.756846	0.096260
C	4.163806	0.009612	-0.486312
C	4.003037	1.521433	-0.507270
H	4.286454	-0.350713	-1.506229
H	5.051778	-0.275295	0.088304
H	4.850584	1.941314	-1.058567
H	3.085637	1.822492	-1.016130
H	4.009787	1.965410	0.490861
C	2.804928	-0.297713	1.527165
C	1.713326	-1.016655	2.305752
H	2.570216	0.763506	1.478451
H	3.771123	-0.411637	2.031261
H	1.604916	-0.512795	3.271485
H	0.751391	-0.969415	1.792914
H	1.952923	-2.063125	2.507675
C	3.372170	-2.226012	0.124619
C	3.684270	-2.841567	-1.231744
H	2.526135	-2.747490	0.568693
H	4.230396	-2.325357	0.798603
H	3.809469	-3.919731	-1.090576
H	2.866694	-2.686356	-1.940030
H	4.608169	-2.458315	-1.670783
B	1.660110	-0.532375	-0.777065
H	0.896655	-1.451686	-0.609155
H	1.934724	-0.290827	-1.926590
C	-5.624174	-0.075245	0.511444
C	-4.327315	0.270448	0.896422
C	-3.213849	-0.308954	0.272625
C	-3.430786	-1.243247	-0.749633
C	-4.725644	-1.592215	-1.138016
C	-5.827677	-1.008535	-0.508113
H	-6.475230	0.381112	1.009391
H	-4.173817	0.995230	1.692314
H	-2.575269	-1.700749	-1.240466
H	-4.874011	-2.321578	-1.929544
H	-6.835899	-1.280922	-0.806653
C	-1.811060	0.096661	0.656950
C	-1.322008	1.310853	-0.159109
H	-1.128568	-0.742265	0.493669
H	-1.773581	0.345959	1.723774
C	0.094699	1.678945	0.208257
H	-1.374846	1.073795	-1.229728
H	-2.006752	2.153050	0.012126
H	0.269364	1.800215	1.278644
H	0.941657	0.649997	-0.255469
Cl	0.623548	3.229948	-0.593233

[2]IM2a'

B3LYP-D3BJ/BS1 G_{corr} in *t*-BuCN:
0.354825 a.u.
B3LYP-D3BJ/BS1 SCF energy in *t*-BuCN:
-1128.382940 a.u.
B3LYP-D3BJ/BS2 SCF energy in *t*-BuCN:
-1128.572972 a.u.
B3LYP-D3BJ/BS2 free energy in *t*-BuCN:
-1128.215135 a.u.

C	-5.502860	-0.879735	-1.051605
C	-4.260922	-1.303816	-0.574404
C	-3.557400	-0.549990	0.375471
C	-4.130841	0.644886	0.833859

C	-5.371972	1.073774	0.360138
C	-6.063204	0.311981	-0.585548
H	-6.033794	-1.480660	-1.784882
H	-3.829996	-2.233038	-0.939559
H	-3.597804	1.240943	1.570774
H	-5.800773	2.000744	0.730950
H	-7.030256	0.642715	-0.953383
C	-2.189321	-0.980179	0.847864
C	-1.056113	-0.366262	0.004522
H	-2.111674	-2.073728	0.810925
H	-2.049952	-0.685125	1.895296
C	0.343670	-0.785814	0.475589
H	-1.199416	-0.655079	-1.045210
H	-1.145745	0.726302	0.034250
H	0.457705	-0.518928	1.532859
H	0.397158	-1.886943	0.448072
Cl	0.972766	2.869124	-0.321220
N	3.040279	-0.418663	-0.017530
C	3.533071	0.614169	0.982395
C	2.792653	0.688300	2.308361
H	3.461119	1.580222	0.488235
H	4.591583	0.390937	1.149051
H	3.311285	1.429325	2.925253
H	1.764055	1.027518	2.181290
H	2.793744	-0.254729	2.859389
C	3.218093	-1.793687	0.603267
C	2.960244	-2.965209	-0.335978
H	2.538825	-1.842647	1.451367
H	4.242253	-1.833029	0.986730
H	2.937686	-3.878379	0.266610
H	1.999958	-2.880875	-0.850104
H	3.746371	-3.083652	-1.084637
C	3.946251	-0.340380	-1.233973
C	3.996660	1.011617	-1.930461
H	3.581125	-1.090417	-1.932745
H	4.945962	-0.637701	-0.901041
H	4.569408	0.885359	-2.854625
H	3.000828	1.370252	-2.198473
H	4.496981	1.779349	-1.336529
B	1.500350	-0.193510	-0.456712
H	1.309605	1.187171	-0.415906
H	1.384978	-0.380746	-1.643933

Et_3NBH_2Cl

B3LYP-D3BJ/BS1 G_{corr} in *t*-BuCN:
0.198058 a.u.
B3LYP-D3BJ/BS1 SCF energy in *t*-BuCN:
-778.805691 a.u.
B3LYP-D3BJ/BS2 SCF energy in *t*-BuCN:
-778.912130 a.u.
B3LYP-D3BJ/BS2 free energy in *t*-BuCN:
-778.711060 a.u.

N	0.493436	0.008114	0.168920
C	1.871269	-0.561111	-0.127427
C	2.680710	0.161222	-1.196325
H	1.715675	-1.592332	-0.437079
H	2.415376	-0.566180	0.822300
H	3.617122	-0.391195	-1.324326
H	2.167859	0.177245	-2.159729
H	2.941254	1.185003	-0.919142
C	0.689466	1.419204	0.687229
C	-0.568304	2.173095	1.092503
H	1.191037	1.968823	-0.106956
H	1.375815	1.344872	1.537142
H	-0.257004	3.167969	1.427555
H	-1.257893	2.296210	0.256582
H	-1.102846	1.700525	1.919613
C	-0.111625	-0.822247	1.281862
C	-0.291986	-2.302679	0.976038
H	-1.080938	-0.382330	1.502948
H	0.534354	-0.686647	2.155091
H	-0.839424	-2.748306	1.812736
H	-0.879980	-2.461560	0.070189
H	0.654925	-2.839019	0.882833
B	-0.361533	-0.026510	-1.215107
H	-0.094997	-1.050861	-1.784165
H	-0.089343	0.965626	-1.834658
Cl	-2.251444	-0.024529	-0.894772

Et_3NBH_3

B3LYP-D3BJ/BS1 G_{corr} in *t*-BuCN:
0.205966 a.u.
B3LYP-D3BJ/BS1 SCF energy in *t*-BuCN:
-319.154047 a.u.
B3LYP-D3BJ/BS2 SCF energy in *t*-BuCN:
-319.227050 a.u.
B3LYP-D3BJ/BS2 free energy in *t*-BuCN:

-319.018072 a.u.

N	-0.000060	-0.000051	0.054614
C	-0.872808	-1.109312	-0.487916
C	-2.336141	-1.066455	-0.070307
H	-0.433776	-2.042395	-0.138969
H	-0.788064	-1.079252	-1.580152
H	-2.822523	-1.961585	-0.471193
H	-2.447425	-1.080132	1.015608
H	-2.869467	-0.199551	-0.467306
C	-0.523827	1.310344	-0.488905
C	0.244417	2.556024	-0.070141
H	-1.551788	1.396799	-0.141404
H	-0.539031	1.221998	-1.581159
H	-0.286816	3.424992	-0.471703
H	0.286237	2.658828	1.015880
H	1.262586	2.584425	-0.465282
C	1.396726	-0.201656	-0.488534
C	2.092080	-1.489543	-0.069854
H	1.985101	0.645887	-0.141215
H	1.327535	-0.144882	-1.580819
H	3.110125	-1.463286	-0.471666
H	2.160665	-1.577356	1.016139
H	1.608047	-2.385544	-0.465350
B	-0.000373	0.000572	1.686433
H	0.015384	-1.156023	2.056525
H	-1.010275	0.565059	2.055301
H	0.993078	0.593108	2.056246

[1]IM3

B3LYP-D3BJ/BS1 G_{corr} in *t*-BuCN:
0.360454 a.u.
B3LYP-D3BJ/BS1 SCF energy in *t*-BuCN:
-3110.503182 a.u.
B3LYP-D3BJ/BS2 SCF energy in *t*-BuCN:
-3110.928440 a.u.
B3LYP-D3BJ/BS2 free energy in *t*-BuCN:
-3110.564974 a.u.

C	-3.137044	3.343522	1.401915
C	-1.755243	3.131601	1.441411
C	-0.942815	3.438562	0.335352
C	-1.557529	3.955169	-0.817445
C	-2.940377	4.173519	-0.862258
C	-3.736136	3.869021	0.249091
H	-3.745167	3.105149	2.270181
H	-1.297229	2.730562	2.342109
H	-0.945002	4.198008	-1.682197
H	-3.393521	4.584678	-1.760136
H	-4.807850	4.042808	0.219435
C	0.547134	3.196672	0.382419
C	0.953677	1.718759	0.211871
H	0.941457	3.554411	1.341920
H	1.034295	3.788168	-0.401448
C	2.477868	1.520315	0.248725
H	0.471823	1.132390	1.008071
H	0.545131	1.343264	-0.737309
H	2.844453	1.954579	1.190584
H	2.911366	2.143109	-0.544577
N	4.514248	-0.331211	-0.004536
C	4.989839	-0.281891	-1.439438
C	4.780611	1.029181	-2.182180
H	4.454596	-1.068457	-1.967915
H	6.054046	-0.542646	-1.426790
H	5.221213	0.914664	-3.177988
H	3.721225	1.256299	-2.310728
H	5.270376	1.880037	-1.703248
C	5.320998	0.666750	0.793723
C	5.110369	0.614101	2.302143
H	5.055308	1.653891	0.421697
H	6.375775	0.490614	0.556887
H	5.598217	1.489616	2.741947
H	4.052196	0.649040	2.569302
H	5.555953	-0.273390	2.757245
C	4.838941	-1.710736	0.529384
C	4.251554	-2.883287	-0.246300
H	4.468046	-1.736623	1.551807
H	5.931138	-1.795853	0.557979
H	4.407650	-3.789517	0.347220
H	3.178713	-2.772835	-0.414692
H	4.732569	-3.036803	-1.214758
B	2.892603	-0.041393	0.113535
H	2.408351	-0.547372	-0.887588
H	2.530578	-0.662119	1.105573
P	-2.353167	-2.069979	-0.706076
O	-3.870575	-1.966622	-1.087041
O	-1.424895	-1.356018	-1.751094
O	-2.190255	-1.255934	0.675785

O	-1.906022	-3.539599	-0.403266
K	-3.936478	-3.114767	1.407108
K	-2.966677	0.712793	-0.918421
K	0.354835	-1.853432	0.234317

[1]TS2

B3LYP-D3BJ/BS1 G_{corr} in *t*-BuCN:
0.358899 a.u.
B3LYP-D3BJ/BS1 SCF energy in *t*-BuCN:
-3110.468220 a.u.
B3LYP-D3BJ/BS2 SCF energy in *t*-BuCN:
-3110.894787 a.u.
B3LYP-D3BJ/BS2 free energy in *t*-BuCN:
-3110.532876 a.u.

C	3.756633	-3.407965	1.337599
C	2.489037	-3.533125	0.758530
C	2.245017	-3.085290	-0.551407
C	3.312716	-2.524944	-1.273182
C	4.581901	-2.398284	-0.698829
C	4.806856	-2.832143	0.612766
H	3.923923	-3.758451	2.352375
H	1.673986	-3.969483	1.331200
H	3.139127	-2.166550	-2.284756
H	5.391674	-1.956290	-1.272550
H	5.790723	-2.731251	1.061466
C	0.839344	-3.046325	-1.101457
C	0.150465	-1.735488	-0.671984
H	0.263556	-3.906388	-0.738035
H	0.861010	-3.103908	-2.196173
C	-1.271957	-1.528005	-1.197985
H	0.132476	-1.708627	0.428689
H	0.735350	-0.875859	-1.012511
H	-1.885124	-2.394747	-0.921378
H	-1.242996	-1.531164	-2.297466
N	-3.936505	-0.951732	0.345786
C	-5.015587	-1.128921	-0.649137
C	-4.708618	-2.122507	-1.766044
H	-5.199877	-0.157999	-1.110895
H	-5.949079	-1.425708	-0.137363
H	-5.543387	-2.120627	-2.474987
H	-3.804077	-1.838738	-2.308964
H	-4.589005	-3.148352	-1.407498
C	-3.704040	-2.219258	1.068566
C	-2.608911	-2.165284	2.130879
H	-3.430765	-2.976696	0.332218
H	-4.648801	-2.553948	1.533890
H	-2.426230	-3.179312	2.501934
H	-1.673865	-1.780476	1.717857
H	-2.884697	-1.547441	2.989857
C	-4.289866	0.109265	1.315719
C	-4.765160	1.425706	0.697890
H	-3.398366	0.304937	1.915121
H	-5.068774	-0.258271	2.006690
H	-4.707149	2.219354	1.451143
H	-4.158116	1.705380	-0.167304
H	-5.802716	1.378570	0.357355
B	-1.939602	-0.179816	-0.663835
H	-2.624458	0.502633	-1.377667
H	-1.663251	0.233450	0.425574
P	0.978949	2.113145	-0.727952
O	2.472077	1.700843	-0.991384
O	-0.076459	1.208828	-1.438326
O	0.783547	2.019046	0.864431
O	0.731008	3.625806	-1.090287
K	2.670248	4.031948	0.588764
K	2.405550	0.003810	0.993879
K	-1.645805	2.876305	0.116995

[1]IM4

B3LYP-D3BJ/BS1 G_{corr} in *t*-BuCN:
0.163933 a.u.
B3LYP-D3BJ/BS1 SCF energy in *t*-BuCN:
-2818.049188 a.u.
B3LYP-D3BJ/BS2 SCF energy in *t*-BuCN:
-2818.401742 a.u.
B3LYP-D3BJ/BS2 free energy in *t*-BuCN:
-2818.234797 a.u.

C	-4.222870	-0.597345	1.618652
C	-3.734852	0.580211	1.042195
C	-3.507236	0.669240	-0.342239
C	-3.793096	-0.455784	-1.135355
C	-4.282852	-1.635887	-0.564667
C	-4.493913	-1.713638	0.817496
H	-4.391272	-0.644290	2.691085
H	-3.514428	1.438469	1.672235
H	-3.617012	-0.407102	-2.207212

H -4.498237 -2.492877 -1.196944
H -4.873436 -2.628482 1.263122
C -2.813290 1.872434 -0.934460
C -1.286001 1.694015 -0.805567
H -3.127670 2.787591 -0.417907
H -3.086480 1.981613 -1.991133
C -0.393549 2.745703 -1.465889
H -1.026094 1.606532 0.256338
H -1.022677 0.724460 -1.247781
H -0.669580 3.752972 -1.108081
H -0.614118 2.750586 -2.548298
B 1.198990 2.440639 -1.188484
H 1.494094 2.837902 -0.035276
H 1.905362 3.101847 -1.978543
P 1.783273 -0.100016 -0.221058
O 0.942016 0.233531 1.048344
O 1.377277 -1.487164 -0.804933
O 1.489082 0.985330 -1.375362
O 3.307466 -0.048959 0.116449
K 2.807477 1.755733 1.963881
K -0.864434 -1.531757 0.581763
K 3.769416 -2.430700 -0.711449

Et_3N

B3LYP-D3BJ/BS1 G_{corr} in *t*-BuCN:
0.171731 a.u.
B3LYP-D3BJ/BS1 SCF energy in *t*-BuCN:
-292.467272 a.u.
B3LYP-D3BJ/BS2 SCF energy in *t*-BuCN:
-292.538838 a.u.
B3LYP-D3BJ/BS2 free energy in *t*-BuCN:
-292.364095 a.u.

N 0.000074 0.000402 0.004105
C -1.394535 0.148787 0.441325
C -2.372550 -0.636072 -0.430485
H -1.656909 1.209115 0.385218
H -1.511724 -0.142981 1.502256
H -3.401535 -0.470779 -0.092629
H -2.294806 -0.312954 -1.473913
H -2.184225 -1.714089 -0.396811
C 0.568047 -1.282106 0.440667
C 1.737875 -1.736202 -0.429906
H -0.219139 -2.039118 0.381977
H 0.877461 -1.239607 1.502215
H 2.107385 -2.711076 -0.093264
H 1.420926 -1.827965 -1.474098
H 2.578375 -1.035543 -0.393471
C 0.825237 1.133684 0.442588
C 0.636082 2.371921 -0.431065
H 1.874774 0.830888 0.389106
H 0.628712 1.381791 1.502889
H 1.290445 3.182363 -0.091520
H 0.881651 2.142093 -1.473193
H -0.392835 2.744971 -0.402242

$I^•$

B3LYP-D3BJ/BS1 G_{corr} in *t*-BuCN:
-0.017503 a.u.
B3LYP-D3BJ/BS1 SCF energy in *t*-BuCN:
-11.397779 a.u.
B3LYP-D3BJ/BS2 SCF energy in *t*-BuCN:
-11.397779 a.u.
B3LYP-D3BJ/BS2 free energy in *t*-BuCN:
-11.412270 a.u.

I 0.000000 0.000000 0.000000

I^-

B3LYP-D3BJ/BS1 G_{corr} in *t*-BuCN:
-0.016848 a.u.
B3LYP-D3BJ/BS1 SCF energy in *t*-BuCN:
-11.605310 a.u.
B3LYP-D3BJ/BS2 SCF energy in *t*-BuCN:
-11.605310 a.u.
B3LYP-D3BJ/BS2 free energy in *t*-BuCN:
-11.619146 a.u.

I 0.000000 0.000000 0.000000

^{2}IM5

B3LYP-D3BJ/BS1 G_{corr} in *t*-BuCN:
0.157303 a.u.
B3LYP-D3BJ/BS1 SCF energy in *t*-BuCN:
-2817.881536 a.u.
B3LYP-D3BJ/BS2 SCF energy in *t*-BuCN:
-2818.222691 a.u.
B3LYP-D3BJ/BS2 free energy in *t*-BuCN:

-2818.062376 a.u.

C	-4.072748	-0.819328	1.647516
C	-3.640162	0.419941	1.161316
C	-3.507788	0.646856	-0.219126
C	-3.818747	-0.401064	-1.102915
C	-4.250730	-1.641982	-0.621820
C	-4.376436	-1.856870	0.756939
H	-4.173942	-0.974272	2.718012
H	-3.399535	1.219876	1.856871
H	-3.717966	-0.241512	-2.173466
H	-4.490415	-2.438510	-1.320456
H	-4.714458	-2.818338	1.131941
C	-2.936193	1.944720	-0.737019
C	-1.397149	1.803131	-0.933142
H	-3.136511	2.754500	-0.028035
H	-3.401472	2.213260	-1.691038
C	-0.745265	3.056875	-1.396593
H	-0.950363	1.487966	0.016041
H	-1.215392	1.000028	-1.656858
H	-0.684694	3.903573	-0.716465
H	-0.733034	3.294983	-2.456760
B	1.604700	2.516055	-1.175721
H	1.652545	2.932213	-0.044976
H	1.798506	3.260560	-2.097802
P	1.780268	-0.018422	-0.254402
O	0.832799	0.354798	0.905523
O	1.370913	-1.315528	-0.975613
O	1.638910	1.178633	-1.433572
O	3.265041	0.008750	0.165362
K	2.739206	1.378192	2.354668
K	-0.845118	-1.620535	0.418255
K	3.792714	-2.316876	-0.902759

[2]TS3

B3LYP-D3BJ/BS1 G_{corr} in *t*-BuCN:
0.158747 a.u.
B3LYP-D3BJ/BS1 SCF energy in *t*-BuCN:
-2817.879322 a.u.
B3LYP-D3BJ/BS2 SCF energy in *t*-BuCN:
-2818.221128 a.u.
B3LYP-D3BJ/BS2 free energy in *t*-BuCN:
-2818.059369 a.u.

C	-3.267427	-1.458037	1.581198
C	-2.809895	-0.150373	1.388198
C	-3.036781	0.528667	0.180613
C	-3.752680	-0.137578	-0.829819
C	-4.209081	-1.447760	-0.645451
C	-3.963221	-2.116722	0.560382
H	-3.074684	-1.964317	2.523068
H	-2.254056	0.347620	2.177536
H	-3.948198	0.368009	-1.771460
H	-4.756850	-1.944774	-1.441263
H	-4.315485	-3.133950	0.704028
C	-2.476592	1.915704	-0.044265
C	-1.599446	2.010472	-1.305260
H	-1.873759	2.198672	0.824702
H	-3.294031	2.644844	-0.119384
C	-0.873338	3.302627	-1.436856
H	-0.866145	1.192789	-1.260296
H	-2.204053	1.830589	-2.205958
H	-0.935825	4.061929	-0.662427
H	-0.488392	3.610439	-2.403615
B	1.619664	2.729103	-0.734191
H	1.333255	2.911438	0.422299
H	1.909988	3.661172	-1.430123
P	1.717080	0.083132	-0.283353
O	0.348206	0.124992	0.421516
O	1.844989	-1.075941	-1.286834
O	1.807321	1.483674	-1.233697
O	2.898723	0.184817	0.702157
K	1.290245	1.043056	2.645312
K	-0.662143	-1.919022	-0.844184
K	4.195613	-1.813471	-0.377624

[1]IM6

B3LYP-D3BJ/BS1 G_{corr} in *t*-BuCN:
-0.001685 a.u.
B3LYP-D3BJ/BS1 SCF energy in *t*-BuCN:
-2468.279284 a.u.
B3LYP-D3BJ/BS2 SCF energy in *t*-BuCN:
-2468.542535 a.u.
B3LYP-D3BJ/BS2 free energy in *t*-BuCN:
-2468.541208 a.u.

B	1.746679	0.000738	2.496948
H	2.663300	0.000047	1.714958

H	1.963902	0.001144	3.677038
P	0.021727	0.000323	0.445228
O	0.621980	1.289970	-0.144146
O	0.630109	-1.285154	-0.144927
O	0.465059	0.001309	2.093317
O	-1.515032	-0.004574	0.474029
K	-1.710182	2.442048	-0.469029
K	2.598618	0.008698	-1.311314
K	-1.693879	-2.451911	-0.471307

[1]PTH

B3LYP-D3BJ/BS1 G_{corr} in *t*-BuCN:
0.215769 a.u.
B3LYP-D3BJ/BS1 SCF energy in *t*-BuCN:
-1146.793890 a.u.
B3LYP-D3BJ/BS2 SCF energy in *t*-BuCN:
-1147.002077 a.u.
B3LYP-D3BJ/BS2 free energy in *t*-BuCN:
-1146.783296 a.u.

S	-2.683159	0.000620	0.917754
N	0.245095	-0.000126	0.141336
C	-0.415783	1.233994	-0.072049
C	-0.416416	-1.233916	-0.072103
C	-1.797713	1.354499	0.174434
C	-1.798415	-1.353697	0.174386
C	1.683759	-0.000414	0.166676
C	0.276172	2.371676	-0.516849
C	0.274919	-2.371985	-0.516903
C	-2.465605	2.556617	-0.063064
C	-2.466953	-2.555452	-0.063133
C	2.328635	-0.000704	1.404188
C	2.427411	-0.000300	-1.018173
C	-0.389930	3.581814	-0.713544
C	-0.391838	-3.581761	-0.713615
C	-1.763697	3.681376	-0.498746
C	-1.765657	-3.680583	-0.498832
C	3.723850	-0.000945	1.459093
C	3.821410	-0.000543	-0.959060
C	4.470261	-0.000876	0.278792
H	1.340775	2.314273	-0.702212
H	1.339552	-2.315187	-0.702241
H	-3.534716	2.612940	0.119198
H	-3.536092	-2.611208	0.119135

H	1.731528	-0.000751	2.310532
H	1.910863	-0.000023	-1.972644
H	0.176315	4.445797	-1.047966
H	0.173943	-4.446049	-1.048036
H	-2.285658	4.618665	-0.662173
H	-2.288125	-4.617588	-0.662270
H	4.225364	-0.001186	2.422039
H	4.399443	-0.000466	-1.878117
H	5.555235	-0.001061	0.322348

[2]PTH$^{+\bullet}$

B3LYP-D3BJ/BS1 G_{corr} in *t*-BuCN:
0.216050 a.u.
B3LYP-D3BJ/BS1 SCF energy in *t*-BuCN:
-1146.624769 a.u.
B3LYP-D3BJ/BS2 SCF energy in *t*-BuCN:
-1146.824113 a.u.
B3LYP-D3BJ/BS2 free energy in *t*-BuCN:
-1146.605051 a.u.

S	2.914563	0.000051	-0.000115
N	-0.222022	-0.000008	0.000047
C	0.415703	-1.236749	-0.000067
C	0.415657	1.236756	-0.000027
C	1.831006	-1.362390	-0.000167
C	1.830955	1.362451	-0.000120
C	-1.675323	-0.000028	0.000191
C	-0.357084	-2.422219	-0.000084
C	-0.357177	2.422194	-0.000006
C	2.433189	-2.632293	-0.000290
C	2.433088	2.632377	-0.000195
C	-2.349751	-0.000042	1.219172
C	-2.349985	-0.000031	-1.218662
C	0.252183	-3.660930	-0.000206
C	0.252041	3.660929	-0.000082
C	1.654108	-3.774255	-0.000313
C	1.653962	3.774309	-0.000179
C	-3.744960	-0.000062	1.212366
C	-3.745192	-0.000050	-1.211590
C	-4.440263	-0.000066	0.000456
H	-1.435367	-2.354656	-0.000003
H	-1.435458	2.354586	0.000067
H	3.516009	-2.706187	-0.000369
H	3.515905	2.706314	-0.000269

H	-1.788174	-0.000038	2.147210
H	-1.788583	-0.000019	-2.146806
H	-0.363948	-4.553477	-0.000220
H	-0.364125	4.553451	-0.000068
H	2.124975	-4.750972	-0.000411
H	2.124790	4.751044	-0.000240
H	-4.285827	-0.000074	2.153066
H	-4.286240	-0.000054	-2.152186
H	-5.525742	-0.000082	0.000559

$K_3PO_4^{+\bullet}$

B3LYP-D3BJ/BS1 G_{corr} in *t*-BuCN:
-0.025045 a.u.
B3LYP-D3BJ/BS1 SCF energy in *t*-BuCN:
-2442.113078 a.u.
B3LYP-D3BJ/BS2 SCF energy in *t*-BuCN:
-2442.371362 a.u.
B3LYP-D3BJ/BS2 free energy in *t*-BuCN:
-2442.3933948 a.u.

P	0.028314	0.002110	0.010703
O	0.840624	1.302500	0.025326
O	-1.103348	-0.023482	1.131181
O	-1.088100	-0.008327	-1.125643
O	0.880775	-1.272479	0.006208
K	3.168071	0.047622	-0.008096
K	-1.540042	2.572030	-0.008626
K	-1.452466	-2.620564	-0.007337

K_3PO_4_H^+

B3LYP-D3BJ/BS1 G_{corr} in *t*-BuCN:
-0.011717 a.u.
B3LYP-D3BJ/BS1 SCF energy in *t*-BuCN:
-2442.786159 a.u.
B3LYP-D3BJ/BS2 SCF energy in *t*-BuCN:
-2443.052475 a.u.
B3LYP-D3BJ/BS2 free energy in *t*-BuCN:
-2443.061180 a.u.

P	0.000359	-0.029876	0.743655
O	-1.280184	0.745669	0.342083
O	0.001615	-0.193290	2.396764
O	-0.000596	-1.483933	0.223646
O	1.281076	0.744698	0.340722
K	0.000661	2.766846	-0.688111
K	-2.425138	-1.349407	-0.717945
K	2.423100	-1.351374	-0.719181
H	0.005479	0.687758	2.798959

^{2}IM0

B3LYP-D3BJ/BS1 G_{corr} in *t*-BuCN:
0.206234 a.u.
B3LYP-D3BJ/BS1 SCF energy in *t*-BuCN:
-2761.301031 a.u.
B3LYP-D3BJ/BS2 SCF energy in *t*-BuCN:
-2761.630509 a.u.
B3LYP-D3BJ/BS2 free energy in *t*-BuCN:
-2761.421263 a.u.

P	2.710289	0.011183	0.031082
O	2.255848	-0.824749	1.286998
O	2.315383	1.497221	0.174278
O	4.270778	-0.235228	0.190942
O	2.140405	-0.598497	-1.272390
K	0.985171	-2.666841	-0.140887
K	0.918898	1.216421	2.388715
K	0.919103	1.601107	-2.023599
N	-2.762407	-0.030373	-0.115929
C	-3.604721	-1.242264	0.233681
C	-3.790567	-1.514712	1.718800
H	-3.117295	-2.099783	-0.229696
H	-4.574230	-1.099656	-0.254309
H	-4.430906	-2.397490	1.814017
H	-2.842443	-1.729871	2.214966
H	-4.284179	-0.694575	2.245722
C	-3.562043	1.207351	0.239072
C	-2.832516	2.536465	0.101818
H	-3.876834	1.081398	1.273924
H	-4.456117	1.195233	-0.392480
H	-3.530107	3.326223	0.398444
H	-1.962085	2.596101	0.758071
H	-2.517735	2.752856	-0.922592
C	-2.562118	-0.039637	-1.615841
C	-1.577565	-1.071906	-2.152313
H	-2.229261	0.960559	-1.893818
H	-3.547849	-0.186998	-2.067581
H	-1.562673	-0.987899	-3.243456
H	-0.561501	-0.895981	-1.790477

H	-1.865294	-2.098930	-1.911649
B	-1.358146	-0.058801	0.665924
H	-0.957935	-1.202432	0.668830
H	-1.549014	0.318458	1.805230
H	-0.583701	0.676863	0.092245

^{2}TS0

B3LYP-D3BJ/BS1 G_{corr} in *t*-BuCN:
0.204768 a.u.
B3LYP-D3BJ/BS1 SCF energy in *t*-BuCN:
-2761.281171 a.u.
B3LYP-D3BJ/BS2 SCF energy in *t*-BuCN:
-2761.612633 a.u.
B3LYP-D3BJ/BS2 free energy in *t*-BuCN:
-2761.404853 a.u.

P	1.891956	-0.035802	0.286175
O	0.809616	0.591641	1.380559
O	1.466013	0.466058	-1.124020
O	3.182952	0.630636	0.782828
O	1.815622	-1.582702	0.379455
K	0.411627	-1.822121	2.531282
K	1.967369	2.903936	0.046239
K	1.768659	-1.876435	-2.220675
N	-2.503227	0.226651	-0.338418
C	-3.914715	-0.337403	-0.360627
C	-4.835368	0.127131	0.758259
H	-3.816850	-1.420837	-0.314033
H	-4.332501	-0.076939	-1.338301
H	-5.814341	-0.333116	0.590215
H	-4.477391	-0.191470	1.739228
H	-4.980448	1.209943	0.773821
C	-2.587781	1.688784	-0.727826
C	-1.291204	2.473907	-0.582965
H	-3.354250	2.138606	-0.098341
H	-2.940845	1.712565	-1.763169
H	-1.451096	3.466146	-1.017755
H	-1.031062	2.595275	0.471276
H	-0.460841	1.988988	-1.101997
C	-1.705606	-0.486109	-1.413853
C	-1.375328	-1.948737	-1.131338
H	-0.768091	0.060662	-1.524606
H	-2.287936	-0.393897	-2.335543
H	-1.092118	-2.417065	-2.082341
H	-0.543390	-2.039500	-0.429281
H	-2.218388	-2.529469	-0.752711
B	-1.850669	0.048133	1.101565
H	-1.964029	-1.101424	1.466087
H	-2.269560	0.872779	1.872519
H	-0.539339	0.315846	1.032863

Cs_2CO_3

B3LYP-D3BJ/BS1 G_{corr} in *t*-BuCN:
-0.022376 a.u.
B3LYP-D3BJ/BS1 SCF energy in *t*-BuCN:
-304.241817 a.u.
B3LYP-D3BJ/BS2 SCF energy in *t*-BuCN:
-304.382510 a.u.
B3LYP-D3BJ/BS2 free energy in *t*-BuCN:
-304.401874 a.u.

O	1.131182	1.961734	0.031269
O	-1.124876	1.959823	-0.068467
O	-0.001224	0.010922	0.128653
C	0.001477	1.323990	0.030642
Cs	2.932900	-0.358667	-0.011107
Cs	-2.933800	-0.357765	-0.005538

$Cs_2CO_3^{+\bullet}$

B3LYP-D3BJ/BS1 G_{corr} in *t*-BuCN:
-0.028889 a.u.
B3LYP-D3BJ/BS1 SCF energy in *t*-BuCN:
-304.128612 a.u.
B3LYP-D3BJ/BS2 SCF energy in *t*-BuCN:
-304.233163 a.u.
B3LYP-D3BJ/BS2 free energy in *t*-BuCN:
-304.259040 a.u.

O	1.098087	2.095180	-0.012425
O	-1.090825	2.090481	-0.072982
O	0.000275	0.187367	0.237067
C	0.002227	1.445209	0.053547
Cs	3.175560	-0.397474	-0.015468
Cs	-3.176900	-0.396262	-0.012434

Cs_2CO_3_H^+

B3LYP-D3BJ/BS1 G_{corr} in *t*-BuCN:
-0.013137 a.u.

B3LYP-D3BJ/BS1 SCF energy in *t*-BuCN:
-304.781859 a.u.
B3LYP-D3BJ/BS2 SCF energy in *t*-BuCN:
-304.892052 a.u.
B3LYP-D3BJ/BS2 free energy in *t*-BuCN:
-304.902177 a.u.

H	-1.184719	2.768234	-0.934614
O	1.076398	1.965351	0.862494
O	-0.595574	3.000249	-0.201161
O	-0.093962	0.869911	-0.743996
C	0.193090	1.859407	-0.008374
Cs	2.625588	-0.559693	-0.019283
Cs	-2.681383	-0.542285	0.049213

^{2}IM0'

B3LYP-D3BJ/BS1 G_{corr} in *t*-BuCN:
0.198364 a.u.
B3LYP-D3BJ/BS1 SCF energy in *t*-BuCN:
-623.299865 a.u.
B3LYP-D3BJ/BS2 SCF energy in *t*-BuCN:
-623.471767 a.u.
B3LYP-D3BJ/BS2 free energy in *t*-BuCN:
-623.270391 a.u.

N	2.335240	-2.128226	0.037346
C	2.506940	-3.611799	-0.200067
C	3.626442	-4.010199	-1.151430
H	1.559611	-3.970482	-0.600203
H	2.657849	-4.075273	0.781032
H	3.614689	-5.101504	-1.239116
H	3.479702	-3.589935	-2.147922
H	4.617869	-3.722730	-0.792836
C	3.545271	-1.640143	0.801578
C	3.622538	-0.137177	1.031740
H	4.420680	-1.954957	0.235542
H	3.550210	-2.173569	1.758276
H	4.508057	0.060721	1.644691
H	3.741370	0.402553	0.090202
H	2.756468	0.262308	1.564815
C	1.131379	-1.942112	0.934642
C	-0.219749	-2.277277	0.319982
H	1.123763	-0.897793	1.240252
H	1.304345	-2.554131	1.826767
H	-0.975372	-2.061834	1.080444
H	-0.425836	-1.652121	-0.550596
H	-0.318059	-3.326984	0.032159
B	2.154241	-1.328295	-1.365394
H	1.447361	-2.000780	-2.088676
H	3.256089	-1.169397	-1.850596
H	1.628336	-0.264046	-1.115196
O	-1.780584	0.148623	2.233237
O	0.152376	1.175827	2.088396
O	-1.299376	1.307115	0.415087
C	-0.975381	0.884522	1.567901
Cs	-3.695400	-0.646258	-0.408942
Cs	1.421419	2.731378	-0.518755

^{2}TS0'

B3LYP-D3BJ/BS1 G_{corr} in *t*-BuCN:
0.199165 a.u.
B3LYP-D3BJ/BS1 SCF energy in *t*-BuCN:
-623.272529 a.u.
B3LYP-D3BJ/BS2 SCF energy in *t*-BuCN:
-623.449038 a.u.
B3LYP-D3BJ/BS2 free energy in *t*-BuCN:
-623.246861 a.u.

N	2.457710	-1.947157	-0.134896
C	3.089026	-2.452406	-1.417250
C	4.512218	-1.977295	-1.674339
H	2.446773	-2.114576	-2.228569
H	3.050237	-3.546496	-1.380306
H	4.811695	-2.340003	-2.662697
H	4.580051	-0.886867	-1.681625
H	5.229005	-2.369009	-0.949172
C	3.302339	-2.430392	1.025742
C	2.732270	-2.143823	2.408282
H	4.268359	-1.939629	0.921347
H	3.448525	-3.507028	0.888596
H	3.518234	-2.344263	3.143297
H	2.412864	-1.105377	2.513318
H	1.882870	-2.786681	2.650816
C	1.081043	-2.571711	-0.007137
C	0.092950	-2.228706	-1.113821
H	0.678004	-2.219829	0.940157
H	1.236842	-3.654186	0.054473
H	-0.842772	-2.749414	-0.880089

H -0.118878 -1.157695 -1.134929
H 0.414241 -2.570897 -2.100715
B 2.378325 -0.345260 -0.159113
H 2.019528 0.043019 -1.242873
H 3.399635 0.141568 0.263262
H 1.393762 0.017915 0.728088
O -1.687829 0.027154 2.006164
O 0.486037 0.209268 1.701440
O -0.926179 0.713191 -0.009987
C -0.779333 0.322232 1.187218
Cs -3.615277 -0.761821 -0.305123
Cs 1.068298 3.011668 -0.206434

²IM1''

B3LYP-D3BJ/BS1 G_{corr} in *t*-BuCN:
0.352651 a.u.
B3LYP-D3BJ/BS1 SCF energy in *t*-BuCN:
-1432.630329 a.u.
B3LYP-D3BJ/BS2 SCF energy in *t*-BuCN:
-1432.934695 a.u.
B3LYP-D3BJ/BS2 free energy in *t*-BuCN:
-1432.579032 a.u.

N -4.477352 -0.089573 0.477187
C -4.259796 -1.446983 -0.157139
C -2.894518 -1.647815 -0.801660
H -5.042687 -1.561783 -0.905892
H -4.425345 -2.193771 0.627521
H -2.925900 -2.597470 -1.349470
H -2.680273 -0.853088 -1.521470
H -2.072565 -1.695183 -0.079738
C -3.403414 0.102332 1.532765
C -3.494628 1.379183 2.353516
H -2.433697 0.091594 1.041348
H -3.455270 -0.771943 2.192019
H -2.563307 1.444252 2.923075
H -3.558014 2.265519 1.717619
H -4.332828 1.390951 3.055249
C -5.819408 -0.108432 1.187645
C -7.023362 -0.395120 0.302318
H -5.932157 0.874548 1.643352
H -5.748543 -0.851526 1.990355
H -7.927037 -0.254574 0.903974
H -7.069082 0.294874 -0.544450
H -7.037213 -1.419464 -0.077631
B -4.436945 1.056967 -0.629267
H -4.808139 0.682586 -1.710632
H -4.803670 2.123912 -0.212757
C 5.156055 3.464555 0.179114
C 4.690081 2.509050 1.088217
C 3.583797 1.707155 0.781523
C 2.948085 1.873111 -0.462106
C 3.415765 2.826393 -1.368913
C 4.520413 3.626429 -1.053509
H 6.014604 4.079991 0.434603
H 5.189453 2.386543 2.046685
H 2.075343 1.247420 -0.679287
H 2.915573 2.951070 -2.327108
H 4.881165 4.367697 -1.761422
C 3.072093 0.654396 1.737868
C 3.419006 -0.765947 1.243353
H 1.980264 0.741900 1.817375
H 3.506469 0.810057 2.732835
C 2.748256 -1.812270 2.111602
H 3.053257 -0.871514 0.218326
H 4.506760 -0.903763 1.237858
H 3.046570 -1.745103 3.159184
H 1.658503 -1.788145 1.986067
Cl 3.250006 -3.517076 1.588360
O -0.268195 0.673816 1.420107
O 0.456685 -0.074239 -0.579313
O -0.134207 -1.544933 1.025157
C 0.019943 -0.312684 0.630310
Cs -0.964000 2.624219 -0.707725
Cs 0.886898 -2.905499 -1.410651

²TS1a''

B3LYP-D3BJ/BS1 G_{corr} in *t*-BuCN:
0.351414 a.u.
B3LYP-D3BJ/BS1 SCF energy in *t*-BuCN:
-1432.606080 a.u.
B3LYP-D3BJ/BS2 SCF energy in *t*-BuCN:
-1432.922453 a.u.
B3LYP-D3BJ/BS2 free energy in *t*-BuCN:
-1432.568027 a.u.

C -6.392100 -1.553091 1.360519
C -5.006397 -1.631660 1.205398

C	-4.290252	-0.587222	0.605557
C	-4.995348	0.542262	0.165649
C	-6.380369	0.625078	0.318178
C	-7.083796	-0.423528	0.916912
H	-6.931015	-2.373968	1.825437
H	-4.470943	-2.512336	1.552139
H	-4.450365	1.360159	-0.299616
H	-6.910810	1.506300	-0.031907
H	-8.161806	-0.361576	1.034502
C	-2.787903	-0.649461	0.480377
C	-2.077653	0.023531	1.664826
H	-2.472902	-0.147460	-0.442112
H	-2.462131	-1.692669	0.416458
C	-0.575142	0.047113	1.521122
H	-2.437762	1.052134	1.763196
H	-2.342213	-0.485552	2.598054
H	-0.154246	0.139263	0.527532
H	-0.026284	0.596193	2.277086
Cl	0.035420	-1.886453	1.989655
N	0.126609	3.422209	-0.481861
C	1.329329	3.983161	0.245302
C	2.157353	2.949697	0.996602
H	0.950665	4.733234	0.939225
H	1.942005	4.490966	-0.507177
H	1.558404	2.452893	1.765616
H	2.588565	2.195163	0.328670
H	2.973770	3.479922	1.500889
C	0.633602	2.475615	-1.563789
C	-0.425568	1.717321	-2.345305
H	1.327831	1.763252	-1.115496
H	1.228362	3.095092	-2.244591
H	0.148058	1.016186	-2.959583
H	-1.075588	1.136523	-1.685770
H	-1.052794	2.354023	-2.976715
C	-0.585104	4.568203	-1.185602
C	-1.185803	5.627867	-0.273622
H	-1.374634	4.110224	-1.780481
H	0.142184	5.016851	-1.870697
H	-1.730909	6.341053	-0.900305
H	-1.892965	5.193275	0.437745
H	-0.430192	6.188551	0.281704
B	-0.871258	2.723982	0.520011
H	-0.819798	3.140132	1.641048
H	-1.917053	2.436163	0.019423
O	3.203230	0.655150	-1.018074
O	2.002521	-0.222247	-2.723311
O	2.756484	-1.554266	-1.070139
C	2.661804	-0.373809	-1.614846
Cs	3.450615	-0.612952	1.668269
Cs	-0.042530	-2.238052	-1.879849

[2]TS1b''

B3LYP-D3BJ/BS1 G_{corr} in *t*-BuCN:
0.355162 a.u.
B3LYP-D3BJ/BS1 SCF energy in *t*-BuCN:
-1432.605843 a.u.
B3LYP-D3BJ/BS2 SCF energy in *t*-BuCN:
-1432.922532 a.u.
B3LYP-D3BJ/BS2 free energy in *t*-BuCN:
-1432.564358 a.u.

N	-2.683018	-2.259694	0.911397
C	-3.496297	-1.154359	1.578558
C	-4.873115	-0.923213	0.969788
H	-2.930862	-0.216981	1.514696
H	-3.595531	-1.436974	2.633471
H	-5.268376	0.005808	1.391405
H	-4.817979	-0.801364	-0.116098
H	-5.587290	-1.721246	1.189963
C	-3.489631	-3.538461	0.961491
C	-2.789867	-4.773890	0.411752
H	-4.392213	-3.350967	0.381958
H	-3.780709	-3.692871	2.005954
H	-3.526969	-5.580974	0.348579
H	-2.391548	-4.593101	-0.589270
H	-1.975971	-5.121201	1.052261
C	-1.428669	-2.464502	1.740765
C	-0.464022	-1.291153	1.779908
H	-0.919169	-3.325328	1.312918
H	-1.767372	-2.728118	2.748986
H	0.383225	-1.594111	2.408291
H	-0.077019	-1.096775	0.778961
H	-0.860985	-0.354220	2.184393
B	-2.351306	-1.857119	-0.587202
H	-1.936313	-0.742966	-0.685856
H	-3.126600	-2.297298	-1.389269
C	5.909963	0.142929	1.211138
C	5.326812	-0.972026	0.604271

C	4.982731	-0.954156	-0.755975
C	5.238663	0.210639	-1.494590
C	5.822057	1.328328	-0.892069
C	6.158074	1.298942	0.464585
H	6.175543	0.107710	2.264188
H	5.135143	-1.868351	1.189381
H	4.978396	0.238979	-2.550024
H	6.018375	2.219356	-1.482173
H	6.616469	2.164863	0.933548
C	4.267482	-2.127267	-1.384702
C	2.740811	-2.010101	-1.178737
H	4.620266	-3.065062	-0.940695
H	4.486739	-2.169757	-2.457426
C	1.960235	-3.148157	-1.791986
H	2.533004	-1.964145	-0.102898
H	2.398721	-1.060801	-1.608920
H	2.022607	-3.191398	-2.880032
H	2.179591	-4.121395	-1.351102
Cl	0.043687	-2.827603	-1.393853
O	-2.689698	1.859828	1.444974
O	-0.609815	1.847306	2.326135
O	-0.918367	2.431804	0.169204
C	-1.406122	2.054027	1.321327
Cs	-3.373000	1.917182	-1.429247
Cs	1.849186	1.452898	0.688718

[2]TS1c''

B3LYP-D3BJ/BS1 G_{corr} in *t*-BuCN:
0.346631 a.u.
B3LYP-D3BJ/BS1 SCF energy in *t*-BuCN:
-1432.593427 a.u.
B3LYP-D3BJ/BS2 SCF energy in *t*-BuCN:
-1432.908307 a.u.
B3LYP-D3BJ/BS2 free energy in *t*-BuCN:
-1432.558664 a.u.

N	3.328843	1.949194	0.253391
C	4.577360	1.116828	0.446599
C	4.843303	0.080604	-0.634724
H	4.461150	0.610647	1.403482
H	5.418372	1.813897	0.527089
H	5.707917	-0.514444	-0.322968
H	3.994836	-0.593652	-0.764052
H	5.083660	0.524936	-1.603264
C	3.481828	2.727906	-1.036936
C	2.335405	3.668875	-1.376746
H	3.583778	1.988869	-1.828973
H	4.422951	3.283722	-0.963758
H	2.526536	4.080443	-2.372899
H	1.378031	3.146329	-1.407780
H	2.255248	4.510701	-0.684936
C	3.250453	2.946228	1.393399
C	3.128405	2.338680	2.783828
H	2.375527	3.563223	1.196392
H	4.140835	3.581587	1.328164
H	2.970842	3.156845	3.493928
H	2.273376	1.661579	2.851882
H	4.027082	1.801650	3.095881
B	2.010196	1.021906	0.227732
H	1.040334	1.682792	0.512611
H	2.179945	0.033219	0.901479
C	-4.603328	1.826987	-3.037887
C	-3.211398	1.919623	-3.146653
C	-2.377286	1.270347	-2.227082
C	-2.962799	0.517424	-1.196355
C	-4.351080	0.425746	-1.088056
C	-5.179077	1.079844	-2.006806
H	-5.235361	2.336456	-3.760783
H	-2.770818	2.501333	-3.953497
H	-2.330206	-0.011300	-0.476364
H	-4.753233	-0.169367	-0.272411
H	-6.260566	1.007186	-1.924662
C	-0.869532	1.372680	-2.318023
C	-0.179163	-0.003736	-2.398336
H	-0.490033	1.897890	-1.432919
H	-0.590502	1.979677	-3.187613
C	1.319836	0.158302	-2.452518
H	-0.458690	-0.596414	-1.515579
H	-0.545473	-0.530895	-3.291522
H	1.685768	0.890997	-3.174024
H	1.762436	0.570894	-1.152451
Cl	2.169863	-1.414235	-2.843943
O	-3.135613	-1.329765	1.546746
O	-2.002196	-3.173928	0.882201
O	-1.078276	-1.128757	0.653045
C	-2.091174	-1.897142	1.031034
Cs	-1.683213	1.198824	2.363008
Cs	0.937102	-3.219847	0.239716

[2]IM2a''

B3LYP-D3BJ/BS1 G_{corr} in *t*-BuCN:
-0.033265 a.u.
B3LYP-D3BJ/BS1 SCF energy in *t*-BuCN:
-764.504945 a.u.
B3LYP-D3BJ/BS2 SCF energy in *t*-BuCN:
-764.653744 a.u.
B3LYP-D3BJ/BS2 free energy in *t*-BuCN:
-764.683997 a.u.

O	1.098022	2.640012	0.657565
O	-1.097814	2.640181	0.657489
O	0.000049	1.614923	-0.967203
C	0.000098	2.302308	0.104404
Cs	2.526765	-0.224256	-0.097731
Cs	-2.526816	-0.224219	-0.097718
Cl	0.000009	-2.606389	0.431789

[1]IM2b''

B3LYP-D3BJ/BS1 G_{corr} in *t*-BuCN:
0.197793 a.u.
B3LYP-D3BJ/BS1 SCF energy in *t*-BuCN:
-1083.073924 a.u.
B3LYP-D3BJ/BS2 SCF energy in *t*-BuCN:
-1083.306815 a.u.
B3LYP-D3BJ/BS2 free energy in *t*-BuCN:
-1083.106010 a.u.

N	-1.249368	-2.278747	0.395762
C	-0.197561	-3.084124	1.154862
C	0.630384	-2.322384	2.182565
H	0.450281	-3.525475	0.398931
H	-0.739441	-3.896435	1.649558
H	1.463225	-2.977200	2.467339
H	1.047355	-1.359921	1.856769
H	0.055524	-2.124965	3.091392
C	-1.957048	-1.387854	1.408107
C	-3.287998	-0.793954	0.961227
H	-1.257044	-0.582623	1.667300
H	-2.132512	-2.009024	2.292390
H	-3.600679	-0.081255	1.731926
H	-3.212635	-0.260621	0.013418
H	-4.079739	-1.541965	0.868293
C	-2.241796	-3.260256	-0.184500
C	-1.655153	-4.304707	-1.123869
H	-2.985700	-2.675232	-0.720831
H	-2.734641	-3.744184	0.664797
H	-2.480493	-4.922704	-1.491754
H	-1.169356	-3.845910	-1.987245
H	-0.941616	-4.968234	-0.630159
B	-0.464629	-1.430614	-0.736623
H	0.304948	-2.166883	-1.294952
H	0.035942	-0.480950	-0.221808
Cl	-1.660079	-0.768125	-2.108393
O	-0.191087	1.288050	2.086765
O	1.941702	0.532653	2.055018
O	1.232285	1.968503	0.472299
C	0.996093	1.269635	1.550687
Cs	-1.579422	2.496027	-0.246353
Cs	3.100911	-0.126677	-0.593775

[1]IM2c''

B3LYP-D3BJ/BS1 G_{corr} in *t*-BuCN:
0.206511 a.u.
B3LYP-D3BJ/BS1 SCF energy in *t*-BuCN:
-623.413948 a.u.
B3LYP-D3BJ/BS2 SCF energy in *t*-BuCN:
-623.619190 a.u.
B3LYP-D3BJ/BS2 free energy in *t*-BuCN:
-623.409667 a.u.

N	-2.457371	1.621846	0.118181
C	-2.602776	2.776909	1.082878
C	-2.084051	4.123596	0.597737
H	-2.057246	2.494615	1.981611
H	-3.666253	2.848499	1.337153
H	-2.232751	4.845962	1.406915
H	-1.017060	4.087697	0.370278
H	-2.617281	4.500817	-0.278256
C	-3.318352	1.911798	-1.089908
C	-3.296588	0.859300	-2.190554
H	-2.965618	2.856510	-1.500663
H	-4.341612	2.059169	-0.726862
H	-3.931966	1.218495	-3.006541
H	-2.290529	0.709433	-2.586691
H	-3.694177	-0.106127	-1.868003
C	-3.011930	0.384995	0.793479

C -2.293992 -0.092244 2.049818
H -2.985250 -0.406759 0.045990
H -4.063790 0.599749 1.015189
H -2.853054 -0.961947 2.417115
H -1.258139 -0.420350 1.887525
H -2.313118 0.650762 2.852080
B -0.906906 1.415868 -0.307530
H -0.800592 0.315892 -0.802524
H -0.231614 1.511620 0.691709
H -0.626547 2.276063 -1.121235
O 0.583023 -1.623006 2.016458
O 2.225507 -0.068300 2.030289
O 1.957154 -1.411844 0.238413
C 1.596584 -1.033679 1.441451
Cs -0.609469 -2.748977 -0.434587
Cs 2.748085 1.362228 -0.507142

$[Cs_2CO_3]_2$

B3LYP-D3BJ/BS1 G_{corr} in *t*-BuCN:
-0.022583 a.u.
B3LYP-D3BJ/BS1 SCF energy in *t*-BuCN:
-608.545225 a.u.
B3LYP-D3BJ/BS2 SCF energy in *t*-BuCN:
-608.801124 a.u.
B3LYP-D3BJ/BS2 free energy in *t*-BuCN:
-608.820695 a.u.

O -2.210272 -1.127731 1.927756
O -3.415833 0.001498 0.388416
O -2.208980 1.130105 1.927202
C -2.632697 0.001287 1.429265
Cs 0.601588 -0.000542 2.221237
Cs -1.772156 2.265810 -0.883400
O 1.148196 1.124746 -0.672781
O -0.155538 -0.000552 -2.136791
O 1.147254 -1.127344 -0.673003
C 0.714894 -0.001069 -1.178599
Cs 3.983107 -0.000560 -0.592322
Cs -1.774935 -2.264837 -0.883521

^{2}IM1'''

B3LYP-D3BJ/BS1 G_{corr} in *t*-BuCN:
0.352470 a.u.
B3LYP-D3BJ/BS1 SCF energy in *t*-BuCN:
-1736.920307 a.u.
B3LYP-D3BJ/BS2 SCF energy in *t*-BuCN:
-1737.356655 a.u.
B3LYP-D3BJ/BS2 free energy in *t*-BuCN:
-1737.001173 a.u.

N -5.477305 -0.271079 -0.067225
C -6.741463 -0.982870 0.388485
C -7.406373 -0.391708 1.622407
H -6.450082 -2.011801 0.593413
H -7.435572 -0.981946 -0.459696
H -8.220824 -1.060361 1.918163
H -6.705190 -0.321573 2.458103
H -7.839379 0.594718 1.440589
C -5.826613 1.176784 -0.343475
C -4.714999 1.996657 -0.984273
H -6.092913 1.613295 0.617829
H -6.717179 1.176819 -0.980800
H -5.006325 3.051340 -0.951851
H -3.773682 1.884530 -0.440608
H -4.545859 1.733541 -2.030379
C -5.037473 -0.900360 -1.375899
C -4.790481 -2.402165 -1.342566
H -4.120202 -0.388628 -1.661691
H -5.808666 -0.663548 -2.117276
H -4.370302 -2.690105 -2.311882
H -4.073956 -2.681727 -0.567373
H -5.706766 -2.978830 -1.198348
B -4.333384 -0.366880 1.038825
H -4.090356 -1.494415 1.376114
H -4.396335 0.510938 1.858943
C 2.673118 4.075627 1.920167
C 2.328992 3.439573 0.724736
C 2.288143 4.171983 -0.475412
C 2.596030 5.538226 -0.446740
C 2.942197 6.170775 0.751467
C 2.982714 5.440325 1.940688
H 2.699839 3.501427 2.843681
H 2.053111 2.378299 0.714763
H 2.566294 6.111983 -1.370150
H 3.179804 7.231101 0.753396
H 3.252816 5.928014 2.873332
C 1.904354 3.494471 -1.774316
C 0.477490 2.913086 -1.739438

H	2.609806	2.682072	-1.990457
H	1.992237	4.211392	-2.598758
C	0.142728	2.266376	-3.069300
H	0.413712	2.164409	-0.943557
H	-0.235971	3.719811	-1.534089
H	0.219801	2.957086	-3.909946
H	0.751644	1.382537	-3.266239
Cl	-1.600311	1.674829	-3.094735
O	0.789738	-3.842137	-1.115266
O	2.986404	-3.323812	-1.103858
O	1.576576	-2.226536	-2.484152
C	1.788958	-3.148112	-1.586021
Cs	-0.951907	-1.350430	-0.973918
Cs	3.525128	-0.326721	-1.062431
O	1.020543	0.654667	0.434366
O	2.401390	-0.471350	1.817445
O	0.167432	-0.806846	1.915009
C	1.206221	-0.210124	1.410776
Cs	-1.310836	1.941573	1.914410
Cs	1.840268	-3.501943	1.746224

[2]TS1a'''

B3LYP-D3BJ/BS1 G_{corr} in *t*-BuCN:
0.356762 a.u.
B3LYP-D3BJ/BS1 SCF energy in *t*-BuCN:
-1736.917177 a.u.
B3LYP-D3BJ/BS2 SCF energy in *t*-BuCN:
-1737.352233 a.u.
B3LYP-D3BJ/BS2 free energy in *t*-BuCN:
-1736.992459 a.u.

C	-4.636193	-2.794078	2.189840
C	-5.167170	-1.533525	1.914296
C	-4.578058	-0.372178	2.437829
C	-3.445596	-0.511183	3.252282
C	-2.908876	-1.772300	3.531236
C	-3.501922	-2.918572	2.998159
H	-5.107138	-3.680474	1.774075
H	-6.049025	-1.445820	1.285196
H	-2.979306	0.378754	3.667536
H	-2.027503	-1.856816	4.161464
H	-3.086841	-3.899218	3.211880
C	-5.138482	0.994943	2.113645
C	-4.876664	1.446763	0.663486
H	-4.713832	1.732957	2.799658
H	-6.223247	0.994178	2.270066
C	-3.424904	1.477121	0.258818
H	-5.396545	0.768986	-0.022232
H	-5.312139	2.439927	0.509768
H	-2.813328	0.629925	0.539757
H	-3.202382	1.877744	-0.721061
Cl	-2.530812	2.956216	1.432074
N	-2.728386	-1.397735	-2.422627
C	-2.244328	-0.731375	-3.693372
C	-1.596269	0.632198	-3.501949
H	-3.115697	-0.639071	-4.340251
H	-1.540751	-1.426880	-4.163830
H	-1.391582	1.042888	-4.497552
H	-2.282581	1.318711	-2.996926
H	-0.653684	0.589279	-2.944226
C	-1.521305	-1.624625	-1.525234
C	-1.793864	-2.256926	-0.169503
H	-1.020486	-0.665285	-1.392128
H	-0.853729	-2.267432	-2.107869
H	-0.828269	-2.363978	0.342568
H	-2.448357	-1.633364	0.442163
H	-2.242485	-3.251077	-0.241841
C	-3.282975	-2.766304	-2.791766
C	-4.484004	-2.749911	-3.725753
H	-3.559932	-3.243039	-1.852999
H	-2.456448	-3.332357	-3.235301
H	-4.854625	-3.775979	-3.814516
H	-5.293376	-2.132093	-3.327297
H	-4.237075	-2.400714	-4.730984
B	-3.857265	-0.552025	-1.711517
H	-4.427824	0.217105	-2.429104
H	-4.431797	-1.159994	-0.862809
O	2.491719	-1.284456	2.634519
O	3.475102	-2.488302	0.999958
O	1.232162	-2.547782	1.247171
C	2.403179	-2.124539	1.642437
Cs	0.140510	0.347469	1.721705
Cs	2.147792	-2.217352	-1.700022
O	0.789598	0.631462	-1.374309
O	3.032327	0.677543	-1.647849
O	2.046244	1.993266	-0.099434
C	1.970796	1.107850	-1.052326
Cs	-0.288675	3.511146	-1.390722

Cs	4.523672	0.405969	1.051118

[2]TS1b'''

B3LYP-D3BJ/BS1 G_{corr} in *t*-BuCN:
0.355149 a.u.
B3LYP-D3BJ/BS1 SCF energy in *t*-BuCN:
-1736.912418 a.u.
B3LYP-D3BJ/BS2 SCF energy in *t*-BuCN:
-1737.348645 a.u.
B3LYP-D3BJ/BS2 free energy in *t*-BuCN:
-1736.990484 a.u.

N	-5.747848	-1.000436	-0.187784
C	-6.489450	-2.313221	0.022210
C	-6.140894	-3.049489	1.307417
H	-6.242470	-2.940350	-0.832561
H	-7.559879	-2.084015	-0.014435
H	-6.640484	-4.022850	1.282743
H	-5.064568	-3.224641	1.389374
H	-6.482562	-2.525536	2.203053
C	-6.060772	-0.093302	0.985044
C	-5.557473	1.337266	0.847265
H	-5.603542	-0.558969	1.856573
H	-7.147094	-0.105355	1.118691
H	-5.634057	1.820209	1.826691
H	-4.515725	1.367854	0.523346
H	-6.151749	1.923652	0.142527
C	-6.292389	-0.351334	-1.448107
C	-6.140973	-1.165524	-2.724114
H	-5.753568	0.586950	-1.559902
H	-7.347923	-0.130249	-1.255292
H	-6.501692	-0.548553	-3.553318
H	-5.095959	-1.413775	-2.919671
H	-6.730855	-2.084850	-2.718604
B	-4.188159	-1.274754	-0.296034
H	-3.917081	-2.171667	-1.041297
H	-3.611786	-1.160559	0.748501
C	1.857854	4.627305	1.536280
C	1.315544	3.854762	0.506041
C	0.802703	4.477466	-0.647372
C	0.840832	5.875743	-0.732295
C	1.384559	6.645306	0.300750
C	1.896788	6.022678	1.440719
H	2.254346	4.135393	2.421841
H	1.281917	2.762074	0.592528
H	0.441876	6.366943	-1.616868
H	1.405472	7.728339	0.213799
H	2.320115	6.616835	2.245920
C	0.208365	3.652931	-1.772063
C	-0.899958	2.696205	-1.284027
H	0.995745	3.052527	-2.248577
H	-0.182944	4.324230	-2.545422
C	-1.571695	1.959820	-2.415373
H	-0.446927	1.969221	-0.602673
H	-1.650670	3.272329	-0.727488
H	-2.190938	2.577129	-3.067110
H	-0.896763	1.330054	-2.997299
Cl	-2.855732	0.642177	-1.610433
O	2.236500	-3.440974	-1.356365
O	4.077191	-2.136493	-1.431756
O	2.254744	-1.589160	-2.648203
C	2.865998	-2.401951	-1.831719
Cs	-0.298640	-1.806492	-0.959110
Cs	3.422409	0.851621	-1.226360
O	0.887946	0.756432	0.516814
O	2.692282	0.136542	1.719385
O	0.749096	-1.005968	1.905648
C	1.454675	-0.036151	1.401422
Cs	-1.655442	0.968240	2.163589
Cs	3.302011	-2.860581	1.452978

[2]TS1c'''

B3LYP-D3BJ/BS1 G_{corr} in *t*-BuCN:
0.352716 a.u.
B3LYP-D3BJ/BS1 SCF energy in *t*-BuCN:
-1736.906855 a.u.
B3LYP-D3BJ/BS2 SCF energy in *t*-BuCN:
-1737.335378 a.u.
B3LYP-D3BJ/BS2 free energy in *t*-BuCN:
-1736.979650 a.u.

N	-2.218437	-3.977189	-0.038796
C	-1.081758	-4.179138	0.948923
C	-1.353645	-3.660898	2.354998
H	-0.197482	-3.685366	0.533201
H	-0.893156	-5.257708	0.990583
H	-0.410047	-3.680992	2.911186
H	-1.728589	-2.635432	2.348363

H -2.065512 -4.277388 2.909761
C -3.481785 -4.553980 0.559968
C -4.746180 -4.423753 -0.278233
H -3.638085 -4.032373 1.502709
H -3.276526 -5.606971 0.783962
H -5.570254 -4.856461 0.298496
H -4.988922 -3.378905 -0.481209
H -4.694618 -4.962280 -1.227457
C -1.870532 -4.778811 -1.281432
C -0.711636 -4.229851 -2.107825
H -2.769088 -4.813229 -1.895794
H -1.657821 -5.797242 -0.939227
H -0.384587 -5.020919 -2.792738
H -1.044701 -3.386555 -2.718680
H 0.146792 -3.899035 -1.509351
B -2.396035 -2.430186 -0.424511
H -3.100777 -2.332768 -1.401700
H -1.321033 -1.901122 -0.510336
C -4.285563 3.974827 -3.035474
C -4.507476 2.784202 -2.336572
C -3.442551 2.069631 -1.771353
C -2.136841 2.573797 -1.914804
C -1.919625 3.765960 -2.610922
C -2.987890 4.470742 -3.176458
H -5.125557 4.511610 -3.468219
H -5.520659 2.403342 -2.230176
H -1.291586 2.052050 -1.449710
H -0.905573 4.146767 -2.711728
H -2.810619 5.395227 -3.719247
C -3.695349 0.778348 -1.021628
C -3.178015 0.813376 0.430971
H -3.209620 -0.052225 -1.544145
H -4.770622 0.563834 -1.025709
C -3.618887 -0.406014 1.204095
H -2.083380 0.878437 0.420423
H -3.560189 1.724059 0.918017
H -4.698415 -0.568056 1.233654
H -3.056321 -1.580241 0.581102
Cl -3.064939 -0.310217 2.950946
O 3.318389 -2.043372 0.863963
O 3.291139 -2.009086 -1.392574
O 1.500876 -2.773217 -0.255020
C 2.720995 -2.295519 -0.262405
Cs 0.820421 -0.748659 2.026552
Cs 0.738615 -0.689762 -2.493305
O 0.064858 1.278776 -0.187822
O 1.907173 1.910462 -1.327650
O 1.874365 2.006644 0.931055
C 1.293686 1.749943 -0.205136
Cs -0.704747 3.420629 1.870564
Cs 4.465784 0.592993 -0.233155

[2]IM2a'''

B3LYP-D3BJ/BS1 G_{corr} in *t*-BuCN:
-0.031861 a.u.
B3LYP-D3BJ/BS1 SCF energy in *t*-BuCN:
-1068.806608 a.u.
B3LYP-D3BJ/BS2 SCF energy in *t*-BuCN:
-1069.073055 a.u.
B3LYP-D3BJ/BS2 free energy in *t*-BuCN:
-1069.101904 a.u.

Cl 4.465574 -0.017076 1.854285
O -2.088175 -1.136354 2.163290
O -3.524223 -0.004043 0.899271
O -2.085712 1.087118 2.196519
C -2.576444 -0.017921 1.764312
Cs 0.915793 -0.033379 2.079362
Cs -2.211107 2.486339 -0.660042
O 0.610717 1.124675 -0.883098
O -0.960287 0.024197 -2.042587
O 0.608180 -1.110482 -0.912628
C 0.085403 0.012921 -1.291752
Cs 3.490729 0.017250 -1.533024
Cs -2.221825 -2.462220 -0.717648

[1]IM2b'''

B3LYP-D3BJ/BS1 G_{corr} in *t*-BuCN:
0.196956 a.u.
B3LYP-D3BJ/BS1 SCF energy in *t*-BuCN:
-1387.371879 a.u.
B3LYP-D3BJ/BS2 SCF energy in *t*-BuCN:
-1387.728574 a.u.
B3LYP-D3BJ/BS2 free energy in *t*-BuCN:
-1387.528606 a.u.

N -4.185916 -1.437585 -0.503736
C -4.917705 -1.628732 -1.822127

C	-4.317970	-0.920443	-3.030968
H	-5.931372	-1.264880	-1.667145
H	-4.962948	-2.707996	-1.998773
H	-4.980092	-1.112224	-3.881360
H	-4.262660	0.160490	-2.888545
H	-3.327086	-1.292893	-3.300782
C	-2.819401	-2.079234	-0.649027
C	-1.847270	-1.914273	0.507821
H	-2.383958	-1.647327	-1.546286
H	-2.996704	-3.140106	-0.852853
H	-0.906055	-2.381157	0.190874
H	-1.624178	-0.868132	0.729868
H	-2.196368	-2.403929	1.421398
C	-4.943922	-2.206064	0.559417
C	-6.375657	-1.753065	0.809366
H	-4.364553	-2.111810	1.475295
H	-4.926693	-3.255935	0.251544
H	-6.753771	-2.313656	1.670263
H	-6.432145	-0.690689	1.052146
H	-7.039825	-1.960972	-0.032575
B	-4.092591	0.156312	-0.220026
H	-5.118119	0.660559	-0.591438
H	-3.113306	0.573568	-0.779477
Cl	-3.921681	0.550604	1.649445
O	1.142621	-2.652186	-0.845750
O	3.277530	-2.363137	-0.165318
O	2.601931	-1.519864	-2.145239
C	2.348039	-2.198552	-1.060790
Cs	0.071104	0.074866	-1.931477
Cs	4.200739	0.564339	-0.566896
O	1.411765	2.143954	-0.097620
O	2.317081	1.166323	1.730699
O	0.144111	0.832334	1.221794
C	1.298239	1.391331	0.964819
Cs	-1.539796	3.168923	0.547724
Cs	1.450614	-1.759526	2.170177

[1]IM2c'''

B3LYP-D3BJ/BS1 G_{corr} in *t*-BuCN:
0.207087 a.u.
B3LYP-D3BJ/BS1 SCF energy in *t*-BuCN:
-927.727232 a.u.
B3LYP-D3BJ/BS2 SCF energy in *t*-BuCN:
-928.047156 a.u.
B3LYP-D3BJ/BS2 free energy in *t*-BuCN:
-927.837058 a.u.

N	3.987104	1.291480	0.122660
C	4.225257	1.077801	-1.354889
C	3.498534	-0.105576	-1.980395
H	5.298863	0.945976	-1.483089
H	3.938639	2.009863	-1.855324
H	3.680769	-0.068664	-3.060874
H	3.912186	-1.048041	-1.612400
H	2.415702	-0.104021	-1.809211
C	2.540250	1.710610	0.291029
C	2.109091	2.082942	1.703505
H	1.924504	0.886259	-0.074178
H	2.393715	2.571253	-0.373034
H	1.018375	2.211112	1.700083
H	2.362705	1.298888	2.421970
H	2.557003	3.017620	2.052772
C	4.858495	2.439907	0.574336
C	6.361815	2.214263	0.489134
H	4.590444	2.636311	1.611050
H	4.570262	3.313923	-0.021211
H	6.855268	3.096219	0.910387
H	6.673612	1.344398	1.070516
H	6.721692	2.098065	-0.535966
B	4.306846	-0.055586	0.976092
H	4.498170	0.268377	2.131837
H	5.298446	-0.575875	0.502600
H	3.345789	-0.792580	0.886733
O	-2.872860	0.832021	2.125868
O	-3.098951	2.271316	0.403691
O	-1.214393	2.289097	1.646580
C	-2.406416	1.812346	1.405216
Cs	-0.257079	-0.638990	2.188113
Cs	-0.679847	2.398086	-1.413219
O	0.500072	-0.467669	-0.902377
O	-1.385285	-0.483872	-2.146578
O	-1.092692	-1.999594	-0.495260
C	-0.668161	-0.989363	-1.200227
Cs	1.742430	-3.231647	-0.610701
Cs	-3.920058	-0.579440	-0.397346

13. References

1. Fan, X., Lei, T., Chen, B., Tung, C.-H. & Wu. L.-Z. Photocatalytic C-C Bond Activation of Oxime Ester for Acyl Radical Generation and Application. *Org. Lett.*, **2019**, *21*, 4153– 4158.
2. Liu, Q.; Zhang, B.-B.; Sheng, H.; Qiao, S.; Wang, Z.-X.; Chen, X.-Y. Visible-Light-Induced Photoreduction of Carborane Phosphonium Salts: Efficient Synthesis of Carborane-Oxindole-Pharmaceutical Hybrids. *Angew. Chem. Int. Ed.* **2023**, *62*, e202305088.
3. Ichitsuka, T.; Fujita, T.; Ichikawa, J. Nickel-Catalyzed Allylic C(sp3)–F Bond Activation of Trifluoromethyl Groups via β-Fluorine Elimination: Synthesis of Difluoro-1,4-dienes. *ACS Catal.* **2015**, *5*, 5947-5950.
4. Pankajakshan, S., Xu, Y.-H., Cheng, J.-K., Low, M.-T. & Loh, T.-P. Palladium-Catalyzed Direct C-H Arylation of Enamides with Simple Arenes. *Angew. Chem. Int. Ed.*, **2012**, *51*, 5701–5705.
5. Boche, G. et al. Crystal and Electronic Structure of Stable Nitrenium Ions. A Comparison with Structurally Related Carbenes. *J. Am. Chem. Soc.*, **2017**, *118*, 4925–4930
6. Pogoreltsev, A., Tulchinsky, Y., Fridman, N. & Gandelman, M. Nitrogen Lewis Acids. *J. Am. Chem. Soc.*, **2017**, *139*, 4062–4067
7. Helle, N.; Kurreck, H.; Bock, M.; Kieslich, W. Endor studies of cation radicals from pharmacologically active phenothiazines. *Magn. Reson. Chem.* **2005**, *23*, 964-970.
8. Haire, L. D.; Krygsman, P. H.; Janzen, E. G.; Oehler, U. M. Correlation of radical structure with EPR spin adduct parameters: utility of the proton, carbon-13, and nitrogen-14 hyperfine splitting constants of aminoxyl adducts of PBN-nitronyl-13C for three-parameter scatter plots. *J. Org. Chem* **1988**, *53*, 4535-4542.
9. Muralirajan, K.; Kancherla, R.; Gimnkhan, A.; Rueping, M. Unactivated Alkyl Chloride Reactivity in Excited-State Palladium Catalysis. *Org. Lett.* **2021**, *23*, 6905-6910.
10. Gao, M.-Y.; Gosmini, C. Cobalt-Catalyzed Reductive Cross-Coupling To Construct Csp3–Csp3 Bonds via C_{sp^3}–S and C_{sp^3}–X Bonds Activation. *Org. Lett.* **2023**, *25*, 7689-7693.

11. Supranovich, V. I.; Levin, V. V.; Kokorekin, V. A.; Dilman, A. D. Generation of Alkyl Radicals from Thiols via Zinc Thiolates: Application for the Synthesis of gem-Difluorostyrenes. *Adv. Synth. Catal.* **2021**, *363*, 2888-2892.

12. Li, X.; Jiao, Y.; Han, L.; Sun, J.; Zhang, X. Defluorinative alkylation of 1-trifluoromethyl alkenes with alkyl radicals derived from visible light-induced deoxygenation of xanthate salts: synthesis of gem-difluoroalkenes. *Org. Biomol. Chem.* **2023**, *21*, 3330-3334.

13. Wang, Y.; Fan, S.; Tang, X. Nucleophilic Organocatalyst for Photochemical Carbon Radical Generation via SN2 Substitution. *Org. Lett.* **2024**, *26*, 4002-4007.

14. Hou, H.; Ou, W.; Su, C. Photochemical C(sp3)–H Activation for Diversity-Oriented Synthesis of 3-Functionalized Oxindoles. *J. Org. Chem* **2024**, *89*, 4120-4127.

15. Zhang, Z.; Zhu, Q.; Pyle, D.; Zhou, X.; Dong, G. Methyl Ketones as Alkyl Halide Surrogates: A Deacylative Halogenation Approach for Strategic Functional Group Conversions. *J. Am. Chem. Soc.* **2023**, *145*, 21096-21103.

16. Zhou, P.; Li, Y.; Xu, T. Molybdenum-Catalyzed Cross-Coupling of Benzyl Alcohols: Direct C–OH Bond Transformation via [2 + 2]-Type Addition and Elimination. *Org. Lett.* **2022**, *24*, 4218-4223.

17. Duan, H.; Meng, L.; Bao, D.; Zhang, H.; Li, Y.; Lei, A. Zinc Chloride Enhanced Arylations of Secondary Benzyl Trifluoroacetates in the Presence of β-Hydrogen Atoms. *Angew. Chem. Int. Ed.* **2010**, *49*, 6387-6390.

18. Frisch, M. J.; Trucks, G. W.; Schlegel, H. B.; Scuseria, G. E.; Robb, M. A.; Cheeseman, J. R.; Scalmani, G.; Barone, V.; Petersson, G. A.; Nakatsuji, H.; Li, X.; Caricato, M.; Marenich, A. V.; Bloino, J.; Janesko, B. G.; Gomperts, R.; Mennucci, B.; Hratchian, H. P.; Ortiz, J. V.; Izmaylov, A. F.; Sonnenberg, J. L.; Williams-Young, D.; Ding, F.; Lipparini, F.; Egidi, F.; Goings, J.; Peng, B.; Petrone, A.; Henderson, T.; Ranasinghe, D.; Zakrzewski, V. G.; Gao, J.; Rega, N.; Zheng, G.; Liang, W.; Hada, M.; Ehara, M.; Toyota, K.; Fukuda, R.; Hasegawa, J.; Ishida, M.; Nakajima, T.; Honda, Y.; Kitao, O.; Nakai, H.; Vreven, T.; Throssell, K.; Montgomery, J. A.; Peralta, Jr., J. E.; Ogliaro, F.; Bearpark, M. J.; Heyd, J. J.; Brothers, E. N.; Kudin, K. N.; Staroverov, V. N.; Keith, T. A.; Kobayashi, R.; Normand, J.; Raghavachari, K.; Rendell, A. P.; Burant, J. C.; Iyengar, S. S.; Tomasi, J.; Cossi, M.; Millam, J. M.; Klene, M.; Adamo, C.; Cammi, R.; Ochterski, J. W.; Martin, R. L.; Morokuma, K.;

Farkas, O.; Foresman, J. B.; Fox, D. J. Gaussian 16, Revision A.03; Gaussian, Inc., Wallingford CT, 2016.

19. Stephens, P. J.; Devlin, F. J.; Chabalowski, C. F.; Frisch, M. J. Ab Initio Calculation of Vibrational Absorption and Circular Dichroism Spectra Using Density Functional Force Fields. *J. Phys. Chem.* **1994**, *98,* 11623–11627.
20. a) Grimme, S.; Antony, J.; Ehrlich, S.; Krieg, H. A consistent and accurate ab initio parametrization of density functional dispersion correction (DFT-D) for the 94 elements H-Pu. *J. Chem. Phys.* **2010**, *132*, 154104; b) Grimme, S.; Ehrlich, S.; Goerigk, L. Effect of the Damping Function in Dispersion Corrected Density Functional Theory. *J. Comput. Chem.* **2011**, *32*, 1456–1465.
21. Marenich, A. V.; Cramer, C. J.; Truhlar, D. G. Universal Solvation Model Based on Solute Electron Density and on a Continuum Model of the Solvent Defined by the Bulk Dielectric Constant and Atomic Surface Tensions. *J. Phys. Chem. B* **2009**, *113*, 6378-6396.
22. Abboud, J.-L. M.; Notario, R. Critical Compilation of Scales of Solvent Parameters. Part I. Pure, Non-hydrogen Bond Donor Solvents. *Pure Appl. Chem.* **1999**, *71*, 645-718.
23. a) Dolg, M.; Wedig, U.; Stoll, H.; Preuss, H. Energy-Adjusted Ab Initio Pseudopotentials for the First Row Transition Elements. *J. Chem. Phys.* **1987**, *86*, 866-872. b) Andrae, D.; Häußermann, U.; Dolg, M.; Stoll, H.; Preuß, H. Energy-Adjusted Ab Initio Pseudopotentials for the Second and Third Row Transition Elements. *Theor. Chim. Acta.* **1990**, *77*, 123-141.
24. a) Hratchian, H. P.; Schlegel, H. B. Accurate Reaction Paths Using a Hessian Based Predictor–Corrector Integrator. *J. Chem. Phys.* **2004**, *120*, 9918-9924; b) Hratchian, H. P.; Schlegel, H. B. Using Hessian Updating to Increase the Efficiency of a Hessian Based Predictor-Corrector Reaction Path Following Method. *J. Chem. Theory Comput.* **2005**, *1*, 61-69.
25. a) Grimme, S.; Bannwarth, C.; Shushkov, P. A Robust and Accurate Tight-Binding Quantum Chemical Method for Structures, Vibrational Frequencies, and Noncovalent Interactions of Large Molecular Systems Parametrized for All spd-Block Elements (Z = 1–86). *J. Chem. Theory Comput.* **2017**, *13*, 1989-2009; b) Bannwarth, C.; Ehlert, S.; Grimme, S. GFN2-xTB—An Accurate and Broadly Parametrized Self-Consistent Tight-Binding Quantum Chemical Method with Multipole Electrostatics and Density-Dependent Dispersion Contributions. *J. Chem.*

Theory Comput. **2019**, *15*, 1652-1671; c) Bannwarth, C.; Caldeweyher, E.; Ehlert, S.; Hansen, A.; Pracht, P.; Seibert, J.; Spicher, S.; Grimme, S. Extended Tight-Binding Quantum Chemistry Methods. *WIREs Comput. Mol. Sci.* **2020**, *11*, e1493.

26. Legault, C. Y., CYLview, 1.0b; Université de Sherbrooke, **2009**
